# Supplementary material for: Membrane protein-regulated networks across human cancers
Source: Nat Commun. 2019 Jul 16;10:3131. doi: 10.1038/s41467-019-10920-8 (PMC6635409; doi:10.1038/s41467-019-10920-8)
Supplement: Supplementary file 1 — Supplementary Information [file 41467_2019_10920_MOESM1_ESM.pdf]

Membrane protein-regulated networks across human cancers

Supplementary Information

Chun-Yu Lin[#], Chia-Hwa Lee[#], Yi-Hsuan Chuang, Jung-Yu Lee, Yi-Yuan Chiu, Yan-Hwa Wu Lee, Yuh-Jyh Jong, Jenn-Kang Hwang, Sing-Han Huang, Li-Ching Chen, Chih-Hsiung Wu, Shih-Hsin Tu, Yuan-Soon Ho, and Jinn-Moon Yang

[#]These authors contributed equally to this work.

Correspondence and requests for materials should be addressed to J.M.Y. (email: moon@faculty.nctu.edu.tw) or to Y.S.H (email: hoyuansn@tmu.edu.tw)

Supplementary Note 1: Evaluation and comparison of prediction accuracies for our reported PPI- and direct PPI-based SIMs, the STRING database, the FpClass method, and the generalized interolog mapping method.

In this study, we compared our SIM with the STRING database[1], the FpClass method[2], and the generalized interolog mapping method[3] using prediction accuracies (**Fig. 2a**, **Supplementary Fig. 4**, and **Supplementary Table 4**) and their features (**Supplementary Table 3**). The STRING database (v. 10.0) contained both known and predicted PPIs assessed by confidence scores. To obtain STRING PPI data of MPs, we first downloaded human protein network data (9606.protein.links.v10.txt) from the STRING database and then filtered by considering both proteins of each PPI recorded in the UniProt complete proteome database. Among 8,472,740 STRING PPIs (scores ranging from 0.15 to 0.99), we assembled 770,140 STRING PPIs for 2,594 MPs (**Supplementary Fig. 44a**). To avoid bias in evaluating and comparing the predictive power of any methods, we used the same criterion to curate the standard positive (including reported PPIs and direct PPIs derived from five public databases) and negative sets (protein pairs with $RSS_{BP} < 0.4$ or $RSS_{CC} < 0.4$) for each method; notably, for our SIM and generalized interologs, all positive cases of each MP were excluded in advance of the PPI templates being selected. Among 18,827 reported PPIs and 2,049 direct PPIs of 2,594 MPs in five public databases, we observed that the overlaps of these PPIs between the STRING database and the public databases were 57% (10,774/18,827) and 76% (1,549/2,049), respectively (**Supplementary Fig. 44a**). To assess the performance of STRING, we needed a group of known interactions as positives for 2,594 MPs, and all elements in this set could be observed among 770,140 STRING PPIs. Here, 10,774 reported PPIs and 1,549 direct PPIs were considered as the total numbers of positive sets for evaluating the predictive power of STRING. Finally, we utilized the precision, recall, F_2 measures, and ROC curves to compare the predictive performances of MP-interacting proteins by using the positive and negative sets across a broad range of thresholds. For example, the recall of STRING was 0.39 (4,234/10,774) when the combined score was set to 0.7 (**Supplementary Fig. 44b**). Similar procedures

were used in assessing the performances of the other methods.

FpClass is a data mining-based method for proteome-wide PPI prediction. First, we generated a total of 182,998,918 paired protein sets between 2,549 MPs and 70,548 proteins of the complete human proteome from UniProt and then calculated their probabilities (ranging from 0 to 1) using the FpClass scoring method. Based on the suggested threshold (probability > 0.47), 43,417 predicted PPIs of MPs were assembled. The generalized interolog mapping method was extended from the interolog mapping[4] that assumed a conserved PPI between a pair of proteins that have interacting homologs in other organisms. For the generalized interolog mapping method, we identified 3,157,311 and 1,313,834 predicted PPIs for MPs using the reported PPIs and direct PPIs as PPI templates when the joint E value was set to 10^{-70}, respectively.

The prediction accuracy shows that the S_{SIM} approach achieved AUCs of 0.924 and 0.932 in positive sets of MP reported PPIs and MP direct PPIs, respectively, outperforming the FpClass (AUCs of 0.811 and 0.929), STRING database (AUCs of 0.824 and 0.921), and generalized interologs (AUCs of 0.793 and 0.835) (**Fig. 2a**, **Supplementary Fig. 4b,c**, and **Supplementary Table 4**). Moreover, reported PPI-based ($S_{SIM} \geq 3.6$) and direct PPI-based ($S_{SIM} \geq 3.7$) SIMs were more accurate for predicting PPIs of different MP types (i.e., enzymes, transporters, miscellaneous, and unclassified) than STRING (high confidences with score > 0.7), FpClass (probability > 0.47) and generalized interologs (joint E value < 10^{-70}) regardless of whether positive sets of all MP PPIs and MP direct PPIs were used. Please note that the STRING database, the FpClass method, and the generalized interolog mapping method included the predictions of both MP and non-MP PPIs; conversely, our method emphasized the characteristics of MPs and was used only to predict MP PPIs. Therefore, the better performance of our method compared with the others was only for MP PPI prediction.

In comparison to the STRING, FpClass, and generalized interologs, our S_{SIM} approach has several improved features for predicting MP PPIs even though the other sources considered similar features (**Supplementary Table 3**). For example, the cytoplasmic region of an integral MP plays a key

role in conveying signals into cells by interacting with other proteins, including direct binding and phosphorylation in intracellular signaling pathways. In contrast to FpClass, which considers all domains of each protein, our SIM sequentially used the potential MP interacting regions (i.e., cytoplasmic regions) to select similar templates by searching known PPIs based on S_{irs} and S_{qul}. According to our previous work[5], S_{rank} was able to avoid the influence of the protein family size and protein length to improve the PPI prediction accuracy of the homology-based approach (e.g., generalized interologs). In summary, SIM achieved the best prediction accuracy for predicting MP PPIs even without regarding gene co-expression, pathway and functional annotations, genomic context associations, post-translational modification, and physicochemical properties.

Supplementary Note 2: Characteristics and performance analysis of reported PPI- and direct PPI-based SIMs and CaMPNets.

Using the positive sets, i.e., 18,827 MP reported PPIs (or 2,049 MP direct PPIs) in humans derived from the reported PPI set (or direct PPI set), and the negative set, we compared the PPI prediction accuracies of reported PPI-based and direct PPI-based SIMs. The result shows that these two S_{SIM} approaches using the positive set of MP direct PPIs achieved similar AUCs (0.944 and 0.932), lightly outperforming the reported PPI-based S_{SIM} approach using MP reported PPIs as the positive set (AUC = 0.924, **Supplementary Fig. 4b**). Moreover, both S_{SIM} approaches also had greater AUCs than six individual or combined scoring methods (**Supplementary Fig. 4a**).

The similar performances between the reported PPI- and direct PPI-based S_{SIM} approaches can be explained by the observation that 84% of other PPIs were annotated by only one interaction type and were given a penalty for evaluating the x_j value of our S_{qul} scoring method, compared with 19% of the direct PPIs (**Supplementary Fig. 5a**). We further examined the predicted PPI numbers for reported PPI-based and direct PPI-based S_{SIM} approaches and observed that (1) the predicted PPI number for the reported PPI-based S_{SIM} approach was higher (4.9-fold, 130,952/26,948) than that for the direct PPI-based S_{SIM} approach; furthermore, (2) the reported PPI-based S_{SIM} approach could additionally identify the interacting proteins of 880 (34%) MPs in comparison to the direct PPI-based S_{SIM} approach (**Supplementary Fig. 5b**). For example, the interacting proteins ERBB2/EGFR for CHRNA9 could be identified only by using the reported PPI-based S_{SIM} approach and were experimentally validated by IP and FRET assays in this work.

Additionally, we identified reported PPI- and direct PPI-based MPP community-regulated pathways to obtain two global maps of the CaMPNet patterns between 994 overlapping MPP communities and 65 cancer-related pathways across 15 cancers. These two heat maps of meta-*z*-scores for reported PPI- and direct PPI-based community-regulated pathways display high positive correlation ($R = 0.632$, $P < 2.2 \times 10^{-16}$, Pearson test, **Supplementary Fig. 18c** and **Supplementary**

Data 3 and **4**). We observed that the top-sized clusters for 1,862 reported PPI-based MPP communities and 1,009 direct PPI-based MPP communities across these cancer-related pathways presented similar associations between communities and pathways in 15 cancers (**Fig. 3c** and **Supplementary Fig. 20**). Considering the above results, we simultaneously present both reported PPI- and direct PPI-based SIMs to generate both resources of reported PPI- and direct PPI-based CaMPNets.

Supplementary Note 3: Biological function and performance analysis of our SIM.

In contrast to STRING (high confidence with scores > 0.7), FpClass (probability > 0.47), and generalized interologs (joint E value < 10^{-70}), our analysis suggested that the precision of the developed method ($S_{SIM} \geq 3.6$; **Supplementary Fig. 6a**) was higher when the numbers of true-positive PPIs ranged from 4,000 to 5,000 (**Supplementary Fig. 6b**). To characterize the biological functions of our predicted PPIs, we first evaluated the joint relative specificity similarities (*RSS*)[5,6] for Gene Ontology (GO) cellular components (CCs) and biological processes (BPs) of the predicted PPIs, as two interacting proteins are often located in adjacent cellular locations and are involved in similar cellular processes. The S_{SIM} values of the PPI candidates are highly correlated with their respective joint *RSS* scores (R = 0.93, $P = 1 \times 10^{-13}$, Pearson test), and the top 1% of the PPI candidates with $S_{SIM} \geq 3.6$ represent high functional similarities (mean ≥ 0.56; **Supplementary Fig. 7a**). We further considered a PPI candidate to be an essential PPI candidate when both of their genes were annotated as being essential in the Database of Essential Genes[7]. When comparing correlations of the percentage of essential PPI candidates between S_{SIM} values and STRING combined scores, our method (R = 0.86, $P = 4 \times 10^{-9}$, Pearson correlation, *t*-test) outperforms STRING (R = 0.19, P = 0.36, Pearson correlation, *t*-test; **Supplementary Fig. 7b**).

Next, we investigated relationships between the numbers of interacting proteins and the annotated Kyoto Encyclopedia of Genes and Genomes (KEGG) pathways for MPs to examine whether the predicted PPIs could uncover new involved processes for MPs. Interestingly, our predicted PPIs (R = 0.83) illuminated more undiscovered regulated pathways than STRING (R = 0.50) and generalized interologs (R = 0.39), excluding the FpClass (R = 0.84), and were even better than the reported PPI data (R = 0.77; **Supplementary Fig. 7c**). Moreover, more than 50% of MPs in five groups could be annotated with at least one new interacting protein ($S_{SIM} \geq 3.6$) and one novel pathway annotation, especially for the transporter group. In addition, to quantify the association and dissociation of MPs with interacting protein candidates in time and space, we collected and assessed correlations between

their expression profile in 7,208 gene expression data sets (≥3 samples) from the Gene Expression Omnibus database (GEO; **Supplementary Fig. 7d**). Similar to the reported PPIs, the predicted PPIs are co-expressed frequently, implying that predicted PPIs are often simultaneously active or inactive in time and space (**Supplementary Fig. 7e**).

Supplementary Note 4: Investigation of functional connectivity and genomic alteration between MPs and binding partners using the experimental data of the loss-of-function screens from the Project Achilles data and the mutation and copy-number alteration (CNA) data of TCGA from the cBioPortal database.

To investigate the functional connectivity between the MPs and their predicted binding partners, we first downloaded the experimental data (ceresgeneeffects.csv) of the loss-of-function screens from the Project Achilles data[8] (version: Achilles Avana Public 17Q4 v2, https://depmap.org/portal/download/all/), which contain the gene-knockout effect profiles of 17,670 genes from 341 cancer cell lines. In these profiles, each gene was assigned a CERES score[8] (also called dependency score) to reflect genetic dependency in each cell line. We selected for analysis cancer cell lines that contained more than five samples, were classified as carcinomas, and were one of the 15 cancer types used in CaMPNets. Finally, 155 cancer cell lines representing nine cancer types were chosen from the Project Achilles data, including 19 bladder urothelial carcinoma (BLCA), 26 breast invasive carcinoma (BRCA), 25 colon adenocarcinoma (COAD), 8 head and neck squamous cell carcinoma (HNSC), 12 uterine corpus endometrial carcinoma (UCEC), 16 kidney cancer (containing kidney renal clear cell carcinoma (KIRC) and kidney renal papillary cell carcinoma (KIRP)), 12 liver hepatocellular carcinoma (LIHC), 26 lung adenocarcinoma (LUAD), and 11 lung squamous cell carcinoma (LUSC) cell lines.

Next, we asked whether MPs and their binding partners exhibited mutual exclusivity or co-occurrence of loss-of-function effects across the 155 cell lines (**Supplementary Fig. 8a)**. By using the odds ratio and Fisher's exact test[9-11], we determined whether mutual exclusivity or co-occurrence was exhibited by each PPI of four sets, including 68,059 and 14,011 MP predicted PPIs identified by reported PPI- and direct PPI-based SIMs, respectively, and 16,334 MP reported PPIs and 1,851 MP direct PPIs as the positive controls. Here, we only analyzed PPIs/protein pairs in which each gene had a CERES score. Each gene with CERES scores ≤ -0.2, -0.4, -0.6, -0.8, or -1.0 (suggested threshold in

Project Achilles Data Portal) is respectively considered to have a significant gene-knockout effect in each cell line. The genes of each PPI/protein pair must both either pass or not pass the given thresholds to be considered a co-occurrence of loss-of-function effects in a cell line.

To further examine the statistical significance of mutual exclusivity and co-occurrence of the loss-of-function effects for MPs and their binding partners (i.e., predicted and positive sets), we generated 1,000 random sets by randomly selecting 68,059 (or 14,011) pairs from all possible combinational pairs, i.e., 2,594 MPs × 8,497 (or 3,115) binding partners, and then determined their mutual exclusivity and co-occurrence in each cancer type or in the group of 155 cell lines. Our results show that the percentages of PPIs having significant mutual exclusivity (or co-occurrence) for predicted and positive sets in the 155 cancer cell lines and nine cancer types were significantly higher than those of random chance (empirical P value < 0.05; **Supplementary Fig. 8b**), except for a few cases (percentage = 0) when the thresholds were set to -0.8 or -1.0 in LIHC and UCEC. We found that loss-of-function effects between the MPs and their binding partners were more likely to be a co-occurrence than a mutual exclusivity; moreover, the direct PPI-related sets and positive PPI-related sets usually presented higher percentages than the corresponding reported PPI-related sets and predicted PPI-related sets, respectively. However, the percentages of MP PPIs with co-occurrence or mutual exclusivity for reported and direct PPI-related sets in the nine cancers or in the 155 cell lines were very low (≤8.29%), especially when the threshold of CERES score was set to -1.0. In comparison to the cell line numbers (8-26) of nine cancer types, we also observed that the median values of cell line numbers for the co-occurrence MP PPIs (P < 0.05, Fisher's exact test), whose both genes have significant gene-knockout effects, ranged from 1 to 8, indicating that the co-occurrence MP PPIs were not frequently shared by multiple cell lines (**Supplementary Fig. 8c)**. Taken together, these results suggest that the two genes of the positive/predicted MP PPIs tend to exhibit co-occurrence of loss-of-function effects in the nine cancer types but only to a limited extent.

To link the predicted PPIs with the development of cancer, we first downloaded mutation and copy-number alteration (CNA) data of TCGA from the cBioPortal[12] database (https://www.cbioportal.org/datasets). In these data, the mutation data are obtained from whole exome sequencing, and the levels of copy number are derived from the copy-number analysis algorithm, GISTIC[13]. For CNAs in each gene, we considered deep deletions and amplifications as biologically relevant. Additionally, 10 types of mutations (missense, nonsense, nonstop, frameshift insertion or deletion, in-frame insertion or deletion, splice region or site, and translation start site) were also considered as biologically relevant for individual genes. For 15 cancer types used in this study, we collected a total of 6,354 samples that contain CNA and/or mutation data, including 408 BLCA, 1,070 BRCA, 36 CHOL, 592 COAD/READ (samples of two types were merged as one set in cBioPortal), 517 HNSC, 65 KICH, 509 KIRC, 283 KIRP, 367 LIHC, 511 LUAD, 487 LUSC, 489 PRAD, 497 THCA, and 523 UCEC samples.

By using the odds ratio and Fisher's exact test, we determined whether mutual exclusivity or co-occurrence of genomic alterations[9-11] was exhibited by each PPI of four sets, including 78,569 and 16,269 MP predicted PPIs identified by reported PPI- and direct PPI-based SIMs, respectively, and 17,203 MP reported PPIs and 1,851 MP direct PPIs as the positive controls. Here, we only analyzed PPIs/protein pairs whose two genes could be mapped from UniProt accession number to the Entrez Gene ID used in TCGA data. To further examine the statistical significance of the functional connectivity between MPs and their binding partners (i.e., predicted and positive sets), we generated 1,000 random sets by randomly selecting 78,569 (or 16,269) pairs from all possible combinational pairs, i.e., 2,594 MPs × 10,284 (or 3,985) binding partners, and then determined whether they exhibited mutual exclusivity or co-occurrence of genomic alterations for each cancer.

Our results show that the percentages of MP PPIs with significant co-occurrence/mutual exclusivity ($P < 0.05$, Fisher's exact test) for our predicted/positive-related sets in 14 cancer types were significantly higher than those of random chance (empirical P value < 0.05; **Supplementary Fig. 9a**);

moreover, the direct PPI-related sets and positive PPI-related sets usually presented higher percentages than the corresponding reported PPI-related sets and predicted PPI-related sets, respectively. In comparison with mutual exclusivity, our results demonstrate that the genomic alterations between the MPs and their binding partners were more likely to be a co-occurrence. Notably, the reported and direct PPI-related sets in most cancers show limited co-occurrence or mutual exclusivity (≤20%), except for UCEC and COAD/READ. In comparison to other cancers, we found that 36% (61/169) and 12% (21/169) of patients with ≥ 2,000 genes that have CNAs or mutations belong to UCEC and COAD/READ, whose sample sizes are 8% (523) and 9% (592) of 6,354 samples, respectively (**Supplementary Fig. 9b,c**). This observation may explain why gene pairs of MP PPIs in these two cancer types have a relatively high chance occurring as either a co-occurrence or a mutual exclusivity. Similar to (but slightly better than) our observations on the loss-of-function screens from the Project Achilles data, these results suggest that the two genes of the MP positive/predicted PPIs tend to exhibit co-occurrence of genomic alterations in most of the 15 cancer types but to a limited extent.

Supplementary Note 5: Analysis of MP-specific PPI network properties.

To investigate the MP-specific PPI network properties, we performed two analyses: (1) an analysis of the hub properties of MPs and other proteins (called non-MPs) in the PPI networks and (2) an analysis of the topological properties and functional enrichment of the MP-focused and non-MP subnetworks. In the first analysis, an *in silico* approach[14,15] that mimics the effect of specifically removing (attacking) hubs in the given network on the characteristic path length, that is, the average shortest path length between node pairs, of the main component of the network was used. Here, the proteins with degrees within the top 25% of all proteins are considered hubs[16] of these human networks. Consistent with previous studies[14,15,17], continuously removing all hubs, beginning from the most connected hubs, had a significantly more pernicious effect on the network integrity than attacking random proteins (**Supplementary Fig. 10**). The effects on the characteristic path length of reported PPI-based (**Supplementary Fig. 10a**) and direct PPI-based (**Supplementary Fig. 10b**) networks on gradual hub (or random node) removal are similar to the high-confidence and filtered high-confidence networks in yeast proposed by Bertin *et al*[17]. We further observed that MP (blue) and non-MP (red) hubs had distinct effects on the overall topology of reported PPI- and direct PPI-based networks when removed from the network. In comparison to the removal of non-MP hubs presenting properties such as party/date hubs, the removal of MP hubs is more like attacking random proteins. This observation can be explained by the fact that the MPs are often located in the periphery but not at the center of biological networks, reflecting the removal of MP hubs having limited effects on the network integrity.

Second, we further derived the MP-focused subnetwork, which extended one layer of PPIs and proteins in 2,594 MPs, and the non-MP subnetwork, which excluded all MPs and their PPIs, from the whole network. These networks/subnetworks possessed scale-free network characteristics with degree exponent (γ) values ranging between 1.42 and 1.78 (**Supplementary Fig. 11a**). Additionally, functional enrichment analyses of the KEGG pathways and cellular components of gene ontology for all proteins of these networks/subnetworks were performed using a hypergeometric test (*P* value <

0.05; **Supplementary Fig. 11b,c**). The results indicate that the MP-focused subnetworks included proteins that were involved in the pathways relevant to cell survival, cell adhesion, and immune response (e.g., PI3K-Akt signaling, focal adhesion, and cytokine-cytokine receptor interaction) and were primarily located in the plasma membrane, cytosol, and endosome. In contrast, proteins of the non-MP subnetworks mainly participated in proteolysis- and central dogma-related pathways and were found in the nucleus and cytosol. Taken together, these results suggest that MPs, which are mainly located in the periphery but not at the center in the cellular interactome, exert limited effects on network integrity and play roles in cell communication and immune responses on the cell surface.

Supplementary Note 6: Characteristics of MPP communities.

Among 1,996 reported PPI-based and 1,105 direct PPI-based MPP communities with at least one binding partner, we observed that most MPP communities (85%-95%) contained at least 50% of non-MP proteins (**Supplementary Fig. 12**). Please note that 1,862 reported PPI-based MPP communities (or 1,009 direct PPI-based MPP communities) could be associated with at least one cancer-related pathway in 15 cancers. To evaluate the overlap between the binding partners identified for different MPs, we used the Jaccard similarity coefficient (JSC) to measure the overlap between any two MPP communities (i.e., an MP with its binding partners) and select the largest overlapping community for each MPP community. Approximately 35% of reported PPI-based communities and 48% of direct PPI-based communities had JSC values of at least 0.6 (**Supplementary Fig. 13a**), and we found that 79% (or 86%) of MPs in these reported PPI-based (or direct PPI-based) communities and their corresponding MPs belonged to the same MP families in comparison to those (58% for reported PPI-based or 69% for direct PPI-based) of other communities with JSC values < 0.6 (**Supplementary Fig. 13b**). Similar to our observations (**Fig. 2b,c** and **Supplementary Fig. 15**), these data imply that MPs in a family often share their interacting proteins to functionally compensate for each other in the regulation of specific cancer-regulated pathways.

Supplementary Note 7: CaMPNets for pan-cancer analysis.

To investigate the roles of CaMPNets in cancer-wide landscape and cancer hallmarks, we built the CaMPNet-based networks using identified MPP community-regulated pathways. For the hubs of the pan-cancer network, we observed that the lymphocyte homing receptor CD44 community, involved in 50 cancer-related pathways, had the highest mean meta-*z*-score, which might provide clues as to why CD44 is considered a cancer stem cell marker in several malignancies of hematopoietic and epithelial origin and how CD44 can regulate focal adhesion kinase to promote cell migration[18]. Almost half of the community hubs belonged to receptors, especially kinase receptors (14%) and G protein-coupled receptors (GPCR) (12%), and similar results for direct PPI-based communities were also observed (Supplementary Fig. 19b). For instance, we show that the dysregulation of epidermal growth factor receptor (EGFR, kinase) plays an important role in the initiation, progression, and metastasis of various epithelial tumors based on its community, which engages 58 pathways in the pan-cancer network (**Fig. 3b**).

To obtain a global map of the CaMPNets patterns, we clustered the enrichment-associated meta-*z*-scores of MPP communities across all 65 cancer-related pathways based on hierarchical clustering using the average agglomeration method with correlations as the distance metric (Supplementary Data 3 and 4). Among the four largest clusters with > 100 reported PPI-based MPP communities, the cluster with 135 communities was broadly linked to the most pathways relevant to cancer hallmarks. For example, the MAPK (pathway number 58), ErbB (59), and TGF-beta (27) signaling pathways are utilized for sustaining proliferative signaling, the VEGF signaling pathway (54) is used to induce angiogenesis, and the oxidative phosphorylation (16) and central carbon metabolism (57) pathways are involved in deregulating cellular energetics[19] (**Fig. 3c** and **Supplementary Fig. 21a**). By contrast, the other three top-sized clusters were relatively specific to certain cancer-related pathways that separately contribute to avoiding immune destruction (**Fig. 3d**, 279 communities), activating invasion and metastasis (**Supplementary Fig. 21b**, 119 communities), and evading growth suppressors and

virus-induced tumor development (**Supplementary Fig. 21c**, 105 communities). The involvement profiles of the top 10 MPP communities in the largest cluster (279 communities) suggest that these communities, such as receptor tyrosine-protein kinase erbB-2/3/4 (ERBB2/3/4) and ephrin type-A receptor 1 (EPHA1), play a major role in immune and inflammation-related pathways, in COAD, kidney chromophobe (KICH), KIRC, LUAD, LUSC, and READ (**Fig. 3d** and **Supplementary Fig. 22a**). The major tissues or organs hosting these six cancers are the digestive system, detoxification system, and respiratory system, which are usually exposed to or metabolize external substances (e.g., food, air, and viruses), and these findings could support the accuracy of our CaMPNets and reflect tissue-specific behaviors of cancers.

For further exploring tumor homogeneity and heterogeneity, we analyzed the involvement of MPP community-regulated pathways in 15 cancers. Communities of the 3rd-ranked cluster (119 communities), such as integrin alpha-V (ITGAV) and cadherin-6/7/8 (CDH6/7/8), relevant to metastasis (e.g., adherens junction and focal adhesion) were found in almost 15 cancer types (homogeneity; **Supplementary Fig. 21b**); by contrast, MPP community-regulated pathways of the largest cluster are mainly discovered in six specific cancers (heterogeneity; **Fig. 3d**). Similarly, the cell cycle (pathway number 21) and p53 (22) signaling pathways regulated by MPP communities in the 4th-ranked cluster (105 communities) were also widely implicated in most cancer types (**Supplementary Fig. 21c**). Moreover, these communities in the 4th-ranked cluster and the other regulated pathways (e.g., viral carcinogenesis) suggested the existence of specific relationships between different viruses and cancer types, thus providing possible routes of access for developing targeted therapies for virus-induced cancers. For example, the hepatitis B virus-related pathway (pathway number 19) in liver cancer and the Epstein-Barr virus (EBV)-related pathway (37) in renal cancers (e.g., KIRC) were involved in relatively more MPP communities (**Supplementary Fig. 22b**). This suggests that CaMPNet resource could provide clues to identify common or specific therapeutic targets among different cancer types.

Supplementary Note 8: Second cancer relationships between 15 cancer types.

Second cancers represent the fourth or fifth most common cancers in the United States, especially for young adults[20], but the mechanisms and causal relationships between first and second cancers remain unclear. To examine the predictive ability of the appearance of secondary tumors, we first curated 49 positive cases of second cancer relationships from the second cancer list of the American Cancer Society, Inc.[21] and used the other 42 relationships as the negative cases, except for LIHC that was not recorded in the list (**Supplementary Fig. 23a**). Next, we presented 14 approaches to suggest possible relationships for 15 cancer types as follows. Based on reported PPI- and direct PPI-based CaMPNets in human cancers, we examined similarities between the profiles (1,862/1,009 communities × 65 pathways) of two cancers via Pearson's *r* correlation and the Jaccard index. Using the gene expression profiles of The Cancer Genome Atlas (TCGA) RNA sequencing (RNA-seq) (**Supplementary Table 2**) and microarray (**Supplementary Table 5**) data sets in cancers, the similarities between the profiles of all genes, 2,594 MP genes, or differentially expressed genes (DEGs) for two cancers were also measured based on Pearson's *r* correlation and the Jaccard index. In the pathway enrichment-based approaches, we used the Jaccard index to evaluate the overlaps between significantly enriched KEGG pathways (P value ≤ 0.05, hypergeometric test) of two cancers based on the pathway enrichment for (1) DEGs selected from TCGA RNA-seq and microarray data sets and for (2) cancer-related genes obtained from DisGeNET[22]. Additionally, the overlaps between the cancer-related genes of two cancers in the DisGeNET database were assessed using the Jaccard index.

The analysis suggested that the $F_{0.5}$ scores of the CaMPNets were higher than those of the other 10 approaches (**Supplementary Fig. 23b**). For example, the best of three approaches with corresponding thresholds were as follows (in order): reported PPI-based CaMPNets using Pearson's $r \geq 0.4$ and using a Jaccard index ≥ 0.15 (**Supplementary Fig. 23c**) and the pathway enrichment-based approach for differentially expressed genes of microarray data sets using a Jaccard index ≥ 0.15. Moreover, we further summarized the 15 cancer type relationships by considering the votes of six and

four different approaches using the correlation value and the Jaccard index, and we observed that these suggested relationships were similar to those of our CaMPNets (**Supplementary Fig. 23d**). In contrast to the voting strategy (suggested by >50% of methods) via the correlation value ($F_{0.5}$ score is 0.64) or the Jaccard index (0.65), our CaMPNets often performed better (~0.66). Taken together, these results suggest that our CaMPNets not only provide some clues but also have utility for further investigating the appearance of secondary tumors.

Supplementary Note 9: The prognostic landscape of MPP community-regulated pathways.

We described some of the most significant community-regulated pathways associated with adverse or favorable outcomes as follows. The combined scores of genes in the APP and CDH3 communities, involving four (e.g., p53 signaling) and two (e.g., microRNAs in cancer) pathways, respectively, were most frequently associated with adverse risks (**Fig. 4b**, right), and these two MPs were linked to the promotion of tumor cell proliferation, migration, and invasion[23-25]. The combined score of genes in the KL community, which contains the *KL* gene encoding Klotho that functions as a tumor suppressor on the cell surface coupled with FGF–FGFR[26] in certain pathways (e.g., Hedgehog signaling), was most frequently associated with favorable outcomes (**Fig. 4b**, left). Surprisingly, the combined scores of genes in the ERBB2 and ERBB3 community-regulated ErbB signaling pathways also represented favorable prognostic associations. This result is reminiscent of some drugs (~15) that are approved for the ERBB family[27], such as trastuzumab against ERBB2 in BRCA[28], and may thus offer better treatment options for cancer patients expressing high levels of ERBB family members.

Considering 65 cancer-related pathways (or 1,862 MPP communities) across cancers for a specific MPP community (or pathway), we further examined whether a meta-analysis (i.e., global meta-*z*-score; **Supplementary Fig. 17c,d**) could determine which communities (or pathways) are associated with biological functions required for long-term survival in cancer patients. In agreement with our findings for the relationship between global meta-*z*-scores for the involvement and prognostic significance of MPP communities and cancer-related pathways, the SLC16A7 and CD40 communities were the most adverse and favorable prognostic communities, respectively; and the focal adhesion and ErbB signaling pathways were the most adverse and favorable prognostic pathways, respectively (**Supplementary Fig. 26**). Several lines of evidence support our finding that *CD40*, encoding a member of the tumor necrosis factor receptor superfamily that is considered a target for cancer therapy (e.g., CD40 agonist), mediates immune activation and regulates tumor apoptosis[29], and the focal adhesion pathway directly activates invasion and metastasis[19].

Among the top ten frequently favorable prognostic MPP communities (**Fig. 4e**) and cancer-related pathways (**Fig. 4f**), ephrin receptor A4 and A7 (EPHA4/7), a tumor suppressor in melanoma[30] and follicular lymphoma[31], was the 1st-ranked and 3rd-ranked frequent favorable prognostic communities across 15 cancers, respectively (**Fig. 4e**, bottom). The endometrial cancer-related pathway was the third most frequent favorable prognostic pathway, and this finding can be explained by the observation that more than 75% of women diagnosed with endometrial cancer are in stages I or II because symptoms frequently appear at early stages, and the 5-year overall survival ranges from 74% to 91%[32] (**Fig. 4f**, bottom).

Supplementary Note 10: Selection of representative MP-interacting proteins.

To select representative interacting protein candidates for each MP in a specific cancer type for experimental validation, we first clustered these candidates based on their biological similarity score (*BS*). We evaluated the BLASTP sequence similarity for any two candidates (X and Y) and further assessed their co-expression (i.e., Pearson's *r*) using the gene expression profiles of tumor samples in TCGA RNA-seq data. Then, we computed the RSS_{BP} and RSS_{CC} scores of GO terms for any pair of candidates to examine whether they participate in similar functional processes (BP) and cellular compartments (CC). For each candidate pair (X-Y), *BS* is given by

$$BS_{X-Y} = r_{X-Y} + \frac{\max(nE_{X\to Y}, nE_{Y\to X}) + RSS_{BP} + RSS_{CC}}{3} \tag{1}$$

where r_{X-Y} is their gene co-expression using Pearson's *r*, and $nE_{X\to Y}$ and $nE_{Y\to X}$ are the normalized *E* values calculated by the *E* values when aligning X to Y and Y to X divided by the *E* values when aligning X to X and Y to Y, respectively. A high *BS* value implies that two candidates with similar sequences are often co-expressed in a specific cancer and regulate similar pathway(s) at the same or adjacent cellular location(s). Finally, we selected at least one candidate in each cluster using the following criteria (**Supplementary Fig. 27a,b**): (1) differentially expressed gene in certain cancer types (e.g., breast cancer); (2) high S_{SIM}; (3) for each type of *S* score, including S_{jss}, S_{irs}, S_{es}, and S_{topo}, its score should be greater than the mean of all the candidates; and (4) a commercially available antibody.

Supplementary Note 11: CHRNA9 community-regulated hepatitis B pathway in liver hepatocellular carcinoma.

To examine our CHRNA9 community-regulated hepatitis B pathway in LIHC and understand the mechanisms underlying the roles of CHRNA9 in the hepatitis B virus (HBV)-regulated pathway and LIHC, we first transfected Hep3B cells that contained an integrated HBV genome with a CRISPR/Cas9 system targeting the CHRNA9 gene locus (i.e., CHRNA9 knockdown) and observed that Hep3B-transfected CHRNA9 knockdown 1 (KD1) and 2 (KD2) viruses significantly reduced CHRNA9 protein expression (**Supplementary Fig. 28a**). Then, we performed microarray analyses of these Hep3B cells (**Supplementary Fig. 28b**). Based on the CaMPNets resource, CHRNA9 was found to interact with tyrosine-protein kinase HCK (HCK), ubiquitin D (UBD), and serine/threonine-protein kinase PLK1 (PLK1), and their genes are co-expressed (orange dashed line) with numerous differentially expressed genes (DEGs), such as Toll-like receptor 2 (*TLR2*) and matrix metalloproteinase-9 (*MMP9*), in the LIHC hepatitis B pathway. Our results show that 12 genes (indicated by an asterisk, e.g., proto-oncogene c-Fos (*FOS*) and early growth response protein 3 (*EGR3*)) among 44 differentially expressed genes (DEGs) in the hepatitis B pathway had opposite regulatory effects between the CHRNA9 knockdown samples and the HBV-infected tissue samples of LIHC in TCGA (**Supplementary Fig. 28b**).

Next, we proposed a model to study the influence of knocking down CHRNA9 in the LIHC hepatitis B pathway (**Supplementary Fig. 28c**). Consistent with previous reports[33-35] showing that the activation of nAChRs suppresses proinflammatory functions, the genes Toll-like receptor 4 (*TLR4*), myeloid differentiation primary response protein MyD88 (*MYD88*), and tumor necrosis factor (*TNF*) were overexpressed to promote the inflammatory and immune responses[36-38] when CHRNA9 was knocked down. Moreover, the analysis also demonstrated that CHRNA9 knockdown might induce the overexpression of downstream genes, such as tumor necrosis factor ligand superfamily member 6 (*FASLG*) and caspase-8 (*CASP8*), and this overexpression could trigger apoptosis of the virus-infected

cell[39]. Finally, we found that the upregulation of several downstream genes was relevant to the invasion and metastasis of hepatocellular carcinoma. For example, interleukin-8 (*CXCL8*) promotes cancer metastasis when it is overexpressed[40-42], and cyclic AMP-dependent transcription factor ATF-4 (*ATF4*) is an important transcription factor associated with cancer progression and metastasis[43,44].

To confirm the proposed model, we further measured the gene expression of these candidates in CHRNA9 knockdown Hep3B cells using a real-time quantitative polymerase chain reaction (Q-PCR) assay. Q-PCR analysis showed that the hepatitis B pathway genes (*MYD88*, *TNF*, *FOS*, *CREB3L2*, *ATF*, *FASLG*, and *TLR4*) were dramatically enhanced in CHRNA9 KD1 and KD2 Hep3B cells (**Supplementary Fig. 28d**). In addition, CHRNA9 knockdown Hep3B cells also displayed a significant increase in the gene and protein expression of HBsAg as determined by Q-PCR and enzyme-linked immunosorbent assay (ELISA) measurements (**Supplementary Fig. 28e,f**). In summary, these observations suggest that CHRNA9 plays functional roles in the hepatitis B pathway in LIHC, especially in inflammatory-, apoptosis-, and metastasis-related processes, and CaMPNets can be used to explore the uncovered relationship between MPs and cancer-related pathways.

Supplementary Note 12: Validation of interacting candidates of CHRNA9 in cancers.

Among 18 potential interacting candidates of CHRNA9, most of the proteins (≥12) were associated with CHRNA9 in breast (BT474 and MDA-MB-231), lung (A549), bladder (RT4) and pancreatic (MIA PaCa-2) cancer cell lines as determined by IP analyses of CHRNA9 (or 18 interacting partners) in a reciprocal fashion[45], whereas six candidates (FYN, CSNK1D, ERBB4, YWHAB, APP, and ABCB1) were weakly detected (or non-detected) in more than two cell lines. In the orthogonal validation via FRET analysis, six candidates (SRC, SFN, ERBB2, ERBB3, YWHAG, and CSNK1D) were strongly associated with CHRNA9, eight candidates (COPS6, YWHAH, EGFR, HCK, APP, YWHAB, INSR, and ERBB4) exhibited a mild association, and four candidates (ABCB1, PLK1, ATXN1, and FYN) were weakly associated (or non-associated) with CHRNA9 in BT474 cells (Fig. 6a and Supplementary Fig. 31a,c,e,f; details in Methods). Consistently, MDA-MB-231 cells exhibited a similar protein interaction profile according to FRET analysis **(Supplementary Fig. 31b,d-f)**.

Supplementary Note 13: Validation of CHRNA9/ERBB2 complex in breast cancer.

Based on a time-dependent nicotine treatment (10 μM), the CHRNA9/ERBB2 complex began to disassociate in less than 3 minutes and dramatically disassociated by 10 minutes (**Supplementary Fig. 33b**). The activated form of the CHRNA9/ERBB2 (Y1248) complex was almost completely disassociated with nicotine treatment by 10 minutes, indicating that nicotine may affect the cross-talk between the CHRNA9 and ERBB2 signaling pathways through complex formation in breast cancer cells. To confirm this hypothesis, we first measured the phosphorylated forms of both ERBB2 and EGFR in a nicotine-dependent manner, revealing that nicotine quickly activated ERBB2 and EGFR in BT474 and MDA-MB-231 cells (**Supplementary Fig. 33c,d**). The downstream mediator AKT (protein kinase B), implicated in cell proliferation and metastasis, was also activated and reached the maximum level after 15 and 30 minutes of nicotine stimulation in BT474 and MDA-MB-231 cells, respectively (**Supplementary Fig. 33e,f**).

To further investigate the association between CHRNA9 and ERBB2 *ex vitro*, we constructed CHRNA9/CFP and ERBB2/YFP fusion proteins to evaluate complex formation using FRET and fluorescence-lifetime imaging microscopy (FLIM) experiments in a low ERBB2 expression breast cancer cell (MDA-MB-231), for observing CHRNA9/ERBB2 interaction. Fluorescent fusion protein carried MDA-MB-231 cells demonstrated strong FRET efficiency (**Supplementary Fig. 33g**, red) on the cell membrane, whereas its cytosol showed weak FRET efficiency (blue). After 30 minutes of exposure to nicotine, the FRET efficiency on the cell membrane completely vanished.

Supplementary Note 14: Investigation of the relationships between nicotine/bupropion and CHRNA9/ERBB2 in breast cancer cells

To further investigate the relationships between nicotine/bupropion and CHRNA9/ERBB2, we selectively knocked down the CHRNA9 and ERBB2 genes of using the CRISPR/Cas9 system. CHRNA9 and ERBB2 in both BT474 and MDA-MB-231 cells were dramatically knocked down in CHRNA9 KD1 and ERBB2 KD2 clones (**Supplementary Figs. 38a,b** and **39a,b)**. Next*,* we found that bupropion pretreatment could attenuate nicotine-induced ERBB2 phosphorylation in BT474 scramble (SC) cells, whereas CHRNA9 KD1 (or ERBB2 KD2) had weak (or no) response to either nicotine or bupropion exposure (**Supplementary Fig. 38c**). In addition, bupropion treatment significantly attenuated nicotine treatment-induced cancer migration (**Supplementary Figs. 38d** and **39c)** and invasion (**Supplementary Figs. 38e** and **39d**) in both BT474 and MDA-MB-231 SC cells, whereas CHRNA9 KD1 and ERBB2 KD2 showed weak and no changes in their migration and invasion abilities to nicotine and bupropion exposure.

Supplementary Note 15: Microarray analysis of bupropion and/or nicotine treatment in an animal model.

To further examine our CaMPNets and understand the mechanisms underlying bupropion's anti-metastasis ability, we performed a microarray analysis of xenograft mouse mammary tumors treated with bupropion or nicotine (see Methods). Pathway enrichment analysis revealed that genes up-regulated in the tumors of mice treated with nicotine compared to the control were significantly associated (P value ≤ 0.05, hypergeometric test) with several metastasis-related pathways (blue box), such as tight junctions, ECM-receptor interactions, and focal adhesion, but those in mice treated with bupropion were not significantly associated with these pathways (**Supplementary Fig. 40**). Moreover, six genes (indicated by an asterisk, e.g., collagen alpha-2(I) chain (*COL1A2*), laminin subunit alpha-4 (*LAMA4*), and tyrosine-protein kinase receptor FLT4 (*FLT4*)) among 24 DEGs in the focal adhesion pathway had opposite regulation effects between the treatment with only bupropion and the BRCA tissue samples in TCGA (**Supplementary Fig. 41a**). We then proposed a model to illustrate how bupropion and nicotine modulate the focal adhesion pathway as well as parts of the pathways of ECM-receptor interaction and cytokine-cytokine receptor interaction by inhibiting CHRNA9 to affect EGFR, ERBB2, FYN, SRC and their upstream/downstream signaling cascades (**Supplementary Fig. 41b**). In comparison with the up-regulation of downstream genes, such as *ROCK1*, *MYLK2*, *MYLPF*, and *VASP*, with only nicotine treatment, these genes were down-regulated when treated with bupropion. Activation or overexpression of *RHOA*, *ROCK1*, or *MYLK* in their signaling pathways have been indicated that these genes perform major functions in tumor cell migration *in vitro* as well as in invasion and progression *in vivo*[46,47]. Differential *MYLPF* expression also participates in contractile forces necessary for cell migration processes[48], and increased *VASP* expression in the Ena/VASP family enhances tumor migration and invasion[49,50]. Similarly, numerous downstream genes (e.g., *MYH1/2/4* and *MYLPF*) of *CHRNA9* in the tight junction also exhibit opposite regulations when treated with only nicotine versus other treatments with bupropion (**Supplementary Fig. 42**). *MYH1* and

MYH4 expression are known to be functionally involved in epithelial-to-mesenchymal differentiation[51]. To confirm the proposed model for bupropion as an inhibitor of CHRNA9, sodium dodecyl sulfate polyacrylamide gel electrophoresis (SDS-PAGE) in MDA-MB-231 cells was used to examine whether bupropion suppresses nicotine-induced carcinogenic signaling pathways. The result illustrates that nicotine exposure enhanced cell motility-related proteins, such as VASP, ROCK1, MYLK2, and MYLPF, while pretreatment with bupropion effectively attenuated the nicotine stimulation effect (**Supplementary Fig. 41c**). We next introduced CRISPR/Cas9 targeting the *CHRNA9* gene locus into MDA-MB-231 cells to confirm the above finding. Nicotine addition enhanced the expression of cell motility-related proteins in control and scramble targeting MDA-MB-231 cells, whereas these proteins were not affected in MDA-MB-231 cells in which CHRNA9 was knocked down (CHRNA9 KD1; **Supplementary Fig. 41d**). This observation suggests that cancer cell migration and invasion through these metastasis-related pathways, such as focal adhesion, ECM-receptor interaction, cytokine-cytokine receptor interaction, adherens junction, and tight junction, triggered by nicotine may be mediated by interactions between CHRNA9 and its interacting proteins in breast cancer cells.

Supplementary Note 16: Advantages for CaMPNets.

Integrating CaMPNets with a meta-*z* approach across numerous malignancies provides pan-cancer analysis for revealing which MPs and regulated pathways are specific and which are common tumor hallmarks. For instance, the cell cycle regulation of many kinds of MPP communities across human cancers, including SELL, CDH1/3, SLC16A7, and SLC12A9, demonstrates that the most essential characteristic of cancer cells is sustained proliferation[19] (**Fig. 2d**). In addition to the findings of the well-known EGFR and CD44 communities, we also discovered communities of APP, CUB domain-containing protein 1 (encoded by *CDCP1*), and N-methyl D-aspartate receptor subtype 2D (a product of *GRIN2D*) in 15 cancers to be broadly implicated in more than 50 cancer-related pathways, especially in invasion and metastasis (**Fig. 3b**).

Our resource also offers clues for observing how changes in cancer-related pathways or MPP communities reflect clinical outcomes, such as alterations in prognostic associations. For example, the ErbB signaling pathway, involved in proliferation and cell motility, is expected to have the association with adverse outcomes[28], but we observed that this pathway regulated by MPP communities, especially the ERBB2 and ERBB3 communities, is most frequently associated with favorable outcomes across multiple cancers. There are several factors may explain this observation. ERBB2 is the target of a therapeutic monoclonal antibody, trastuzumab, which extends survival in patients with metastatic breast cancer[52]. Recent evidences have also indicated that ERBB2 might be considered a predictive marker due to its response to adjuvant chemotherapy and endocrine therapy[28,53,54]. Clearly, the prognostic significance of ERBB2 must be sufficiently validated by well-controlled and well-designed studies with a sufficient follow-up time.

Our observations may explain why multi-target therapeutics are effective and overcome adaptive resistance to cancer therapy[55] since MPs belonging to the same family often display complementary functions toward each other in mediating certain pathways in distinct human cancers. For example, regorafenib is an approved multi-targeted kinase inhibitor (e.g., vascular endothelial growth factor

receptor 1/2/3) as a treatment for metastatic colorectal cancer and advanced gastrointestinal stromal tumors[27,56]. By using CaMPNets resource and Homopharma approach[57], we also found that the VEGF signaling pathway is regulated by CHRNA9 in BRCA and by CHRNA7 in LUAD and COAD, as well as both nAChRs have similar ligand binding environments, implying that the repurposed role of the drug bupropion could have potential for interfering with interaction(s) between CHRNA7 and ErbB proteins and blocking metastasis in LUAD and COAD. Smoking-induced tumor progression, especially that of nicotine and tobacco-specific nitrosamine 4-(methylnitrosamino)-1-(3-pyridyl)-1-butanone (NNK) via CHRNA7, has been sufficiently investigated[58,59]. As CHRNA7 is often associated with lung cancer[59], our previous studies[33,60] have shown that CHRNA9 expression plays important roles in breast cancer progression. This study suggests that bupropion could have utility in smoking-related metastatic cancer patients with high nAChR expression.

Supplementary Note 17: Limitations, challenges, and perspectives for CaMPNets.

CaMPNets have several limitations, challenges, and perspectives. First, note that the reported PPI set includes a wide range of interactions: direct physical binding of two proteins, co-existence of proteins in a stable complex, genetic interaction, etc. We collected data from five databases and did not determine whether any reported PPI has been validated, is well-established, or reflects the current consensus of the community. Therefore, the interactions reported in these databases should not be presumed to indicate direct and physical binding. The associated PubMed identifier(s) and PSI-MI term(s) are provided on the website as supporting evidence for a reported PPI, allowing the users to assess the confidence of the reported PPI. The direct PPI set was collected and curated via relatively rigorous criteria to offer reliable PPI templates. However, the predicted PPIs identified by reported PPI- or direct PPI-based SIM still need to be experimentally validated. To investigate MP PPIs and their regulated networks in 15 cancers, we hope that the data produced by our SIM and CaMPNets will provide the scientific community with clues that will reduce the time and cost of experiments. Second, one potential limitation of CaMPNets is that our approach may miss gene sets that belong to the same pathway but are potentially not sufficiently co-expressed, as the co-expression may be less evident in the case signaling pathways that are often hierarchical in nature. While it is optimal to directly evaluate the co-expression of protein pairs and the epigenetic changes (e.g., phosphorylation and dephosphorylation) using proteomic compared with genomic data, this information remains restricted to only a few cancer proteomic resources. As proteomic data become increasingly available, such as the NCI Clinical Proteomic Tumor Analysis Consortium (CPTAC)[61,62], CaMPNets can be expected to provide more robust data for future interactome investigations associated with cancers. For example, we found that the genes involved in the CHRNA9 community are co-expressed with several genes of neighboring proteins in the adhesion junction pathway but not co-expressed with the genes of most proteins that convey signals via phosphorylation and dephosphorylation (**Fig. 5b**). Integration of genomic and proteomic data may solve this problem. Third, the MPs in the plasma membrane, such as

receptor tyrosine kinases, integrins, and cytokine receptors, often play key roles in the formation, progression, and metastasis of different tumors; therefore, we chose to focus on plasma MP to start with the establishment and analysis of CaMPNets. We believe that our approach is a general strategy for identifying interactions of other MPs and further constructing disease-associated networks via corresponding genomic data. Forth, another CaMPNets challenge is to consider interactions between MPs and extracellular proteins for elucidating tumor microenvironment responses. Within the tumor microenvironment, stromal cell types are genetically stable, unlike tumor cells[63]. However, separating tumor and stromal cells in tumor tissues to reflect signaling transduction between complicated tissue environments and tumor cells is challenging. The unabated progress in single-cell sequencing, and next-generation sequencing technologies[64,65] will allow this issue to be addressed as well as revolutionize our model to reconstruct close-to-real CaMPNets.

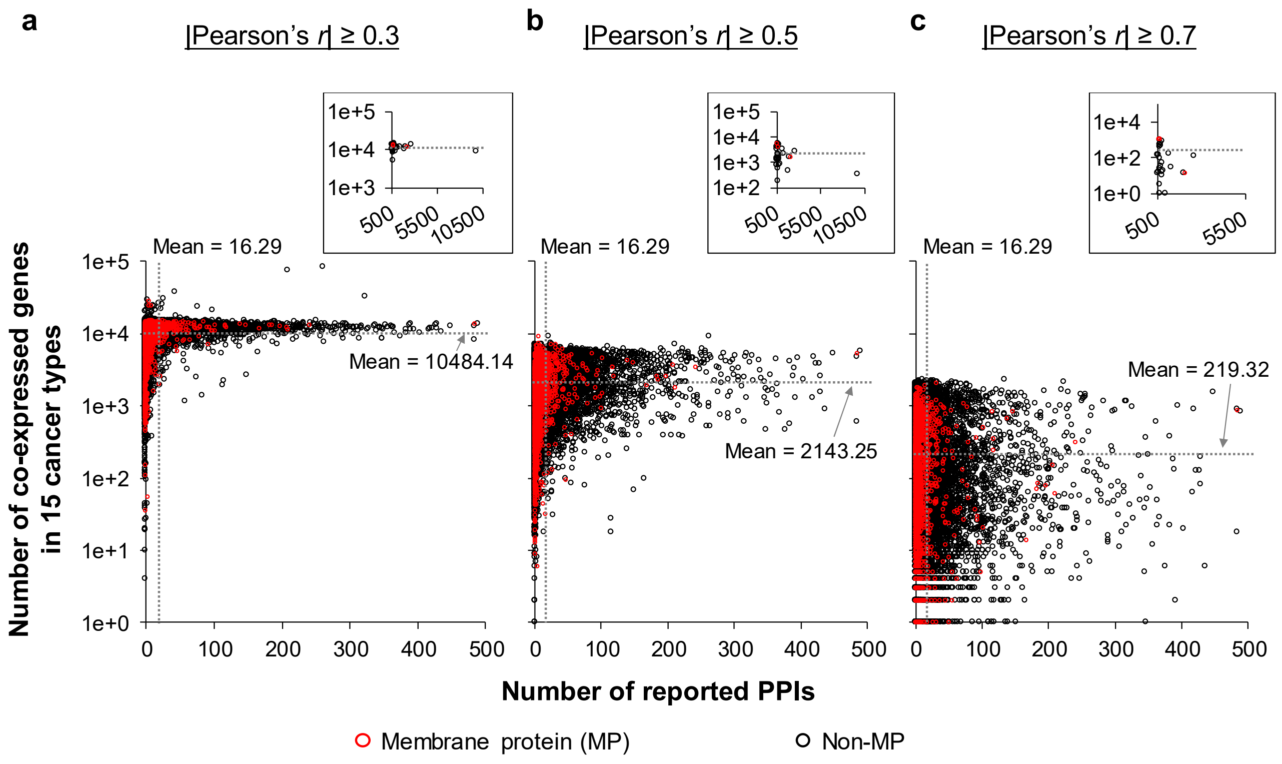

|Pearson's r| ≥ 0.3

Number of co-expressed pairs > mean

	MPs	Non-MPs
Number of reported PPIs > mean	196	3321
Others	954	8633

P value = 9 × 10^{-17}

|Pearson's r| ≥ 0.5

Number of co-expressed pairs > mean

	MPs	Non-MPs
Number of reported PPIs > mean	134	2327
Others	679	5652

P value = 3 × 10^{-16}

|Pearson's r| ≥ 0.7

Number of co-expressed pairs > mean

	MPs	Non-MPs
Number of reported PPIs > mean	102	1362
Others	688	3594

P value = 9 × 10^{-21}

Supplementary Figure 1. Distribution of the numbers of reported protein-protein interactions (PPIs) and co-expressed gene pairs for membrane proteins (MPs) and other proteins based on the human reported PPI network and gene expression profiles of tumor samples from 15 cancer types in The Cancer Genome Atlas (TCGA) RNA sequencing (RNA-seq) data.

For each gene of MPs (red) and other proteins (black), we assessed and combined all the co-expressed gene pairs with (**a**) |Pearson's *r*| values ≥ 0.3, (**b**) 0.5, or (**c**) 0.7 using gene expression profiles in 15 cancer types. Among proteins with numbers of co-expressed pairs higher than the mean, the percentages of MPs with the numbers of reported PPIs (>mean) are significantly lower than those of other proteins ($P < 3 \times 10^{-16}$, Fisher's exact test).

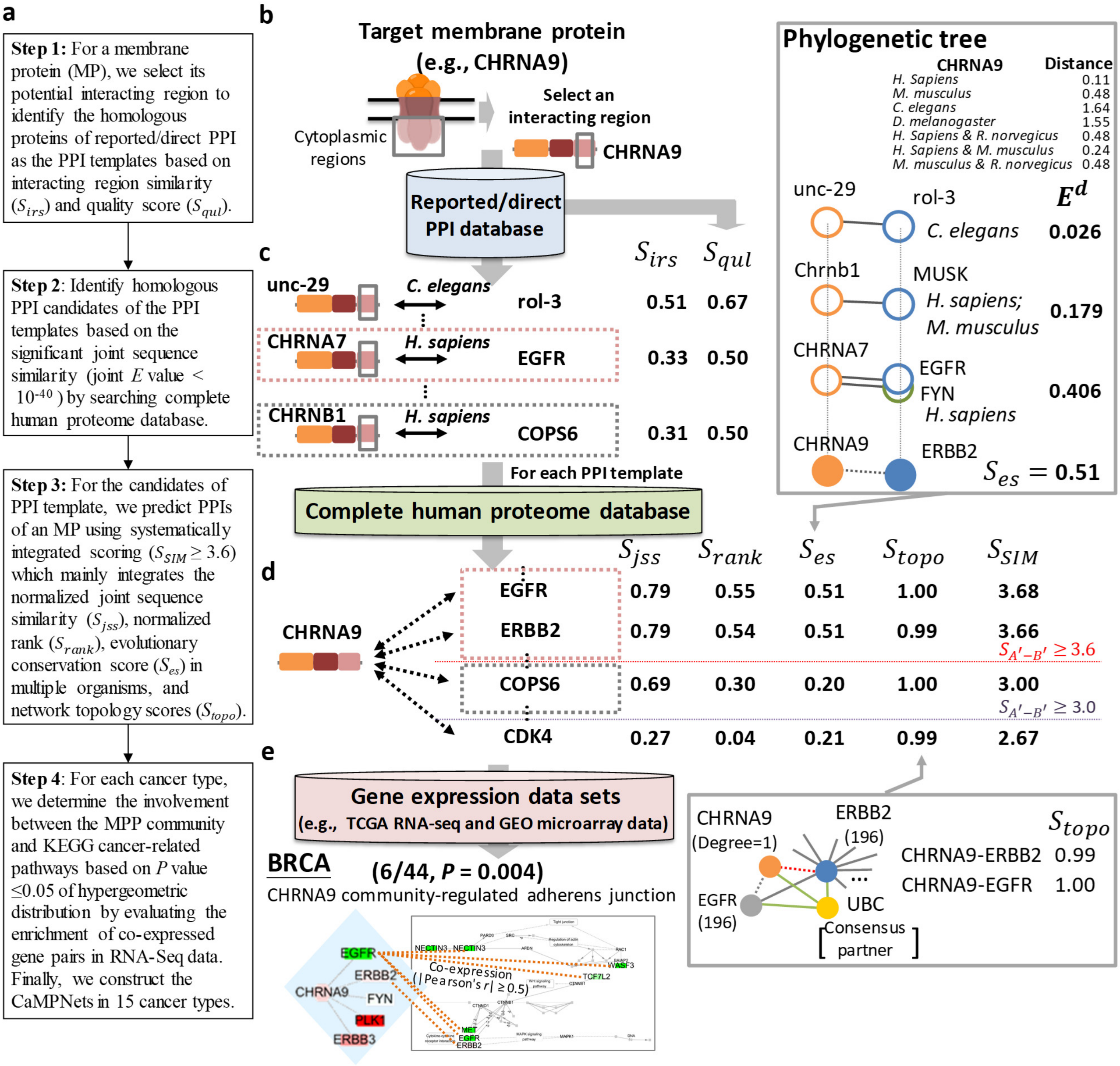

Supplementary Figure 2. Schematic diagram of predicting proteins that interact with 2,594 MPs in the plasma membrane.

(**a**) The main procedure. (**b**) Selection of a possible interacting region (i.e., cytoplasmic region) for an MP to infer its interacting proteins. (**c**) PPI templates of an MP derived from a reported PPI set using the interacting region similarity score (S_{irs}) and template quality score (S_{qul}). (**d**) Predicted PPI candidates of the templates determined by searching the complete human proteome database. PPI candidates are scored using the integrated scoring method (S_{SIM}), including the S_{irs} and S_{qul} for the PPI template, the normalized joint sequence similarity (S_{jss}), the normalized rank (S_{rank}), the

evolutionarily conserved score (S_{es}) on multiple organisms, and the network topology score (S_{topo}) in the PPI network of the target organism (*H. sapiens* is shown here). Here, we give an example to descript the S_{es}. The distances from the source organisms, *H. sapiens*, *M. musculus*, and *C. elegans*, to the target organism, *H. sapiens*, are 0.105, 0.479, and 1.641, respectively. If the number of source organisms containing at least one PPI template used to infer the PPI candidate A'-B' is 7, such as for *H. sapiens*, *C. elegans*, and the *H. sapiens* and *M. musculus* hybrid, the normalized evolutionary distances of CHRNA9-ERBB2 are 0.409, 0.026, and 0.179, respectively. The predicted PPIs with S_{SIM} ≥ 3.6 and ≥ 3.0 are considered PPI candidates of the queried MP with high and median confidences, respectively. (**e**) Community-regulated pathways between the MP and its interacting proteins (called the MPP community) and Kyoto Encyclopedia of Genes and Genomes (KEGG) cancer-related pathways (e.g., adherens junction) in a cancer type determined by enrichment *P* value ≤ 0.05 using hypergeometric distribution based on the probabilities of co-expressed gene pairs. Gene pairs with Pearson's *r* values ≥ 0.5 or ≤ −0.5 calculated by the gene expression profiles of tumor samples in TCGA RNA-seq data or Gene Expression Omnibus (GEO) microarray data are considered co-expressed gene pairs. These MPP community-regulated pathways were used to construct Cancer Membrane Protein-regulated Networks (CaMPNets) in 15 cancer types.

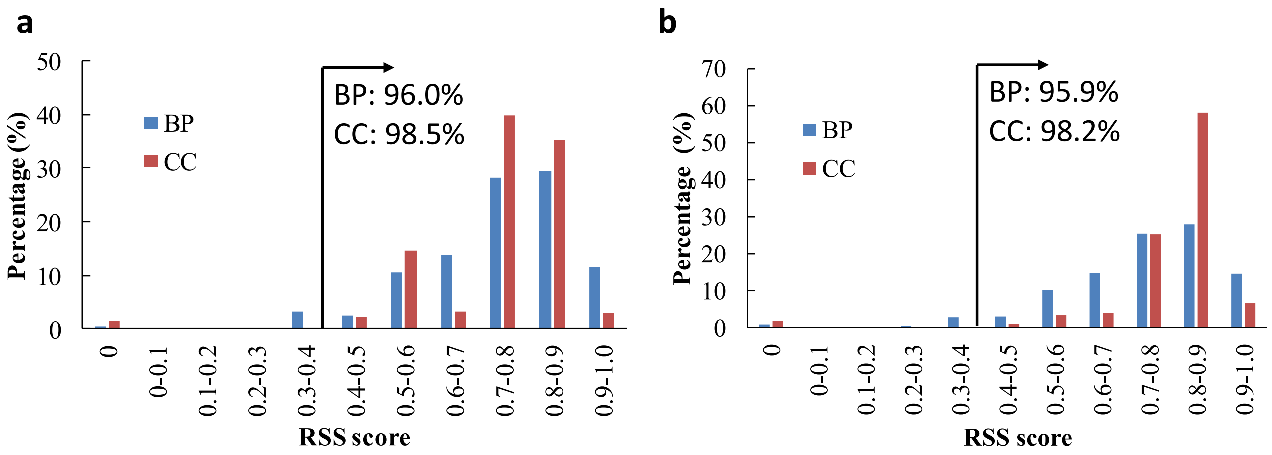

Supplementary Figure 3. Relative Specificity Similarity (*RSS*) score distributions of biological processes (BPs) and cellular components (CCs) for (a) 18,827 human PPIs of 2,594 MPs and (b) 179,248 human PPIs in the reported PPI database. More than 95% of human PPIs (or PPIs of MPs) in the reported PPI set have RSS_{BP} (blue) ≥ 0.4 and RSS_{CC} (red) ≥ 0.4. In this study, a protein pair with $RSS_{BP} < 0.4$ or $RSS_{CC} < 0.4$ were considered as a negative case.

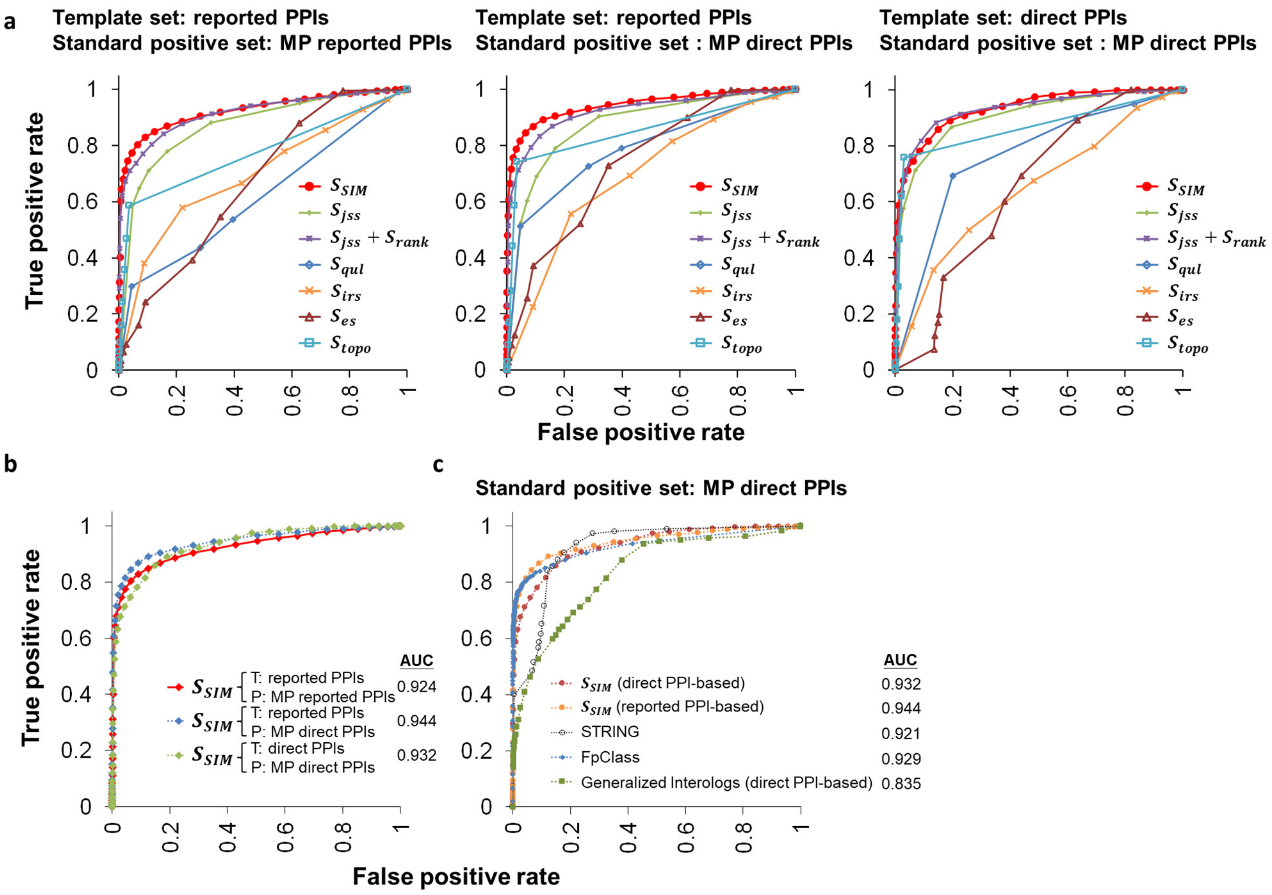

Supplementary Figure 4. Receiver operator characteristic (ROC) curves of the prediction accuracies of our S_{SIM} method, respective scoring methods, the STRING database, the FpClass method, and the generalized interolog mapping method, using reported PPIs or direct PPIs as templates (T) and MP reported PPIs or MP direct PPIs as standard positives (P).

(**a**) Comparison of the prediction accuracies between two S_{SIM} methods and six respective scoring methods using MP reported PPIs or MP direct PPIs as positive cases. (**b**) Comparison of the prediction accuracies between the reported PPI-based S_{SIM} approach using all MP reported PPIs as positive cases (red), the reported PPI-based S_{SIM} approach using MP direct PPIs as positive cases (blue), and the direct PPI-based S_{SIM} approach using MP direct PPIs as positive cases (green). The average area under the receiver operating characteristic curve (AUC) is shown for each method. (**c**) Comparison of the prediction accuracies between the reported PPI- and direct PPI-based S_{SIM} methods, the STRING

database (black), the FpClass method (blue), and the direct PPI-based generalized interolog mapping method (green) using MP direct PPIs as standard positives.

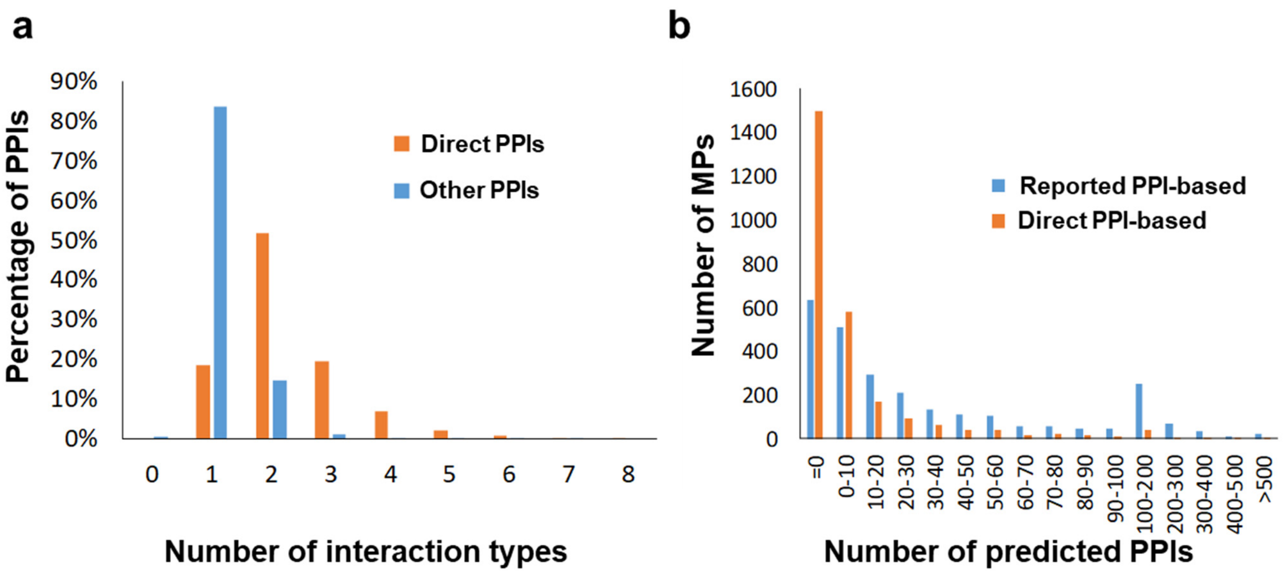

Supplementary Figure 5. Characteristics of the reported PPIs, consisting of direct PPIs and other PPIs, and the predicting MP PPIs, identified by reported PPI- and direct PPI-based SIMs. (**a**) Distributions of interaction type numbers for 176,087 direct PPIs (orange) and 573,000 other PPIs (blue). The identifier number and name of the interaction element were collected and processed using the standard 'interaction type' vocabulary implemented in the PSI MI[66]. (**b**) Relationships between the numbers of MPs and the numbers of predicted PPIs identified by reported PPI-based (blue) and direct PPI-based (orange) SIMs.

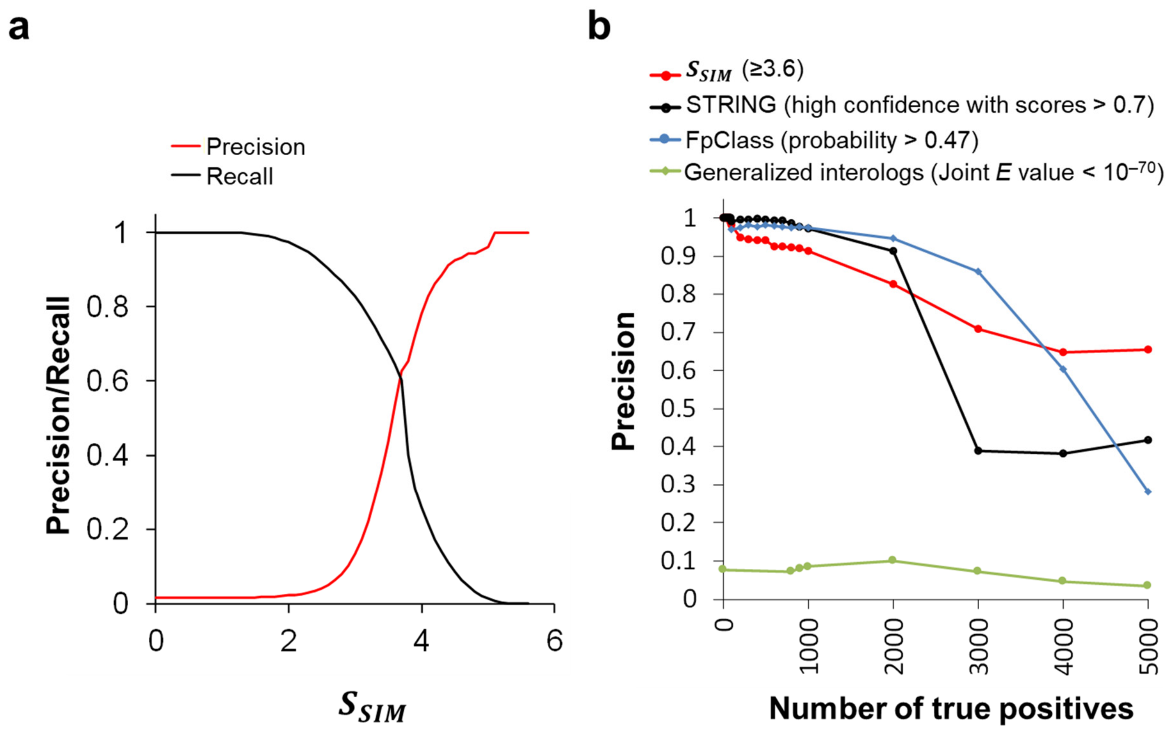

Supplementary Figure 6. Performance analysis for the reported PPI-based SIM strategy.

(**a**) Relationships between recall (black) and precision (red) with S_{SIM} values for 2,594 MPs. Precision and recall are defined as TP / (TP+FP) and TP / (TP+FN), where TP, FP, and FN are the numbers of true-positive, false-positive, and false-negative cases, respectively. We observed the highest F_2 score of 0.62 when S_{SIM} was set to 3.6. (**b**) Relationships between the numbers of true-positives and the precision of our scoring method ($S_{SIM} \geq 3.6$; red), STRING (high confidence > 0.7; black), and FpClass (probability > 0.47; blue), and generalized interologs (joint E value < 10^{-70}; green). Our analysis also suggested that our method ($S_{SIM} \geq 3.6$) accomplishes the highest precision, with its number of true-positive PPIs ranging from 4,000 to 5,000.

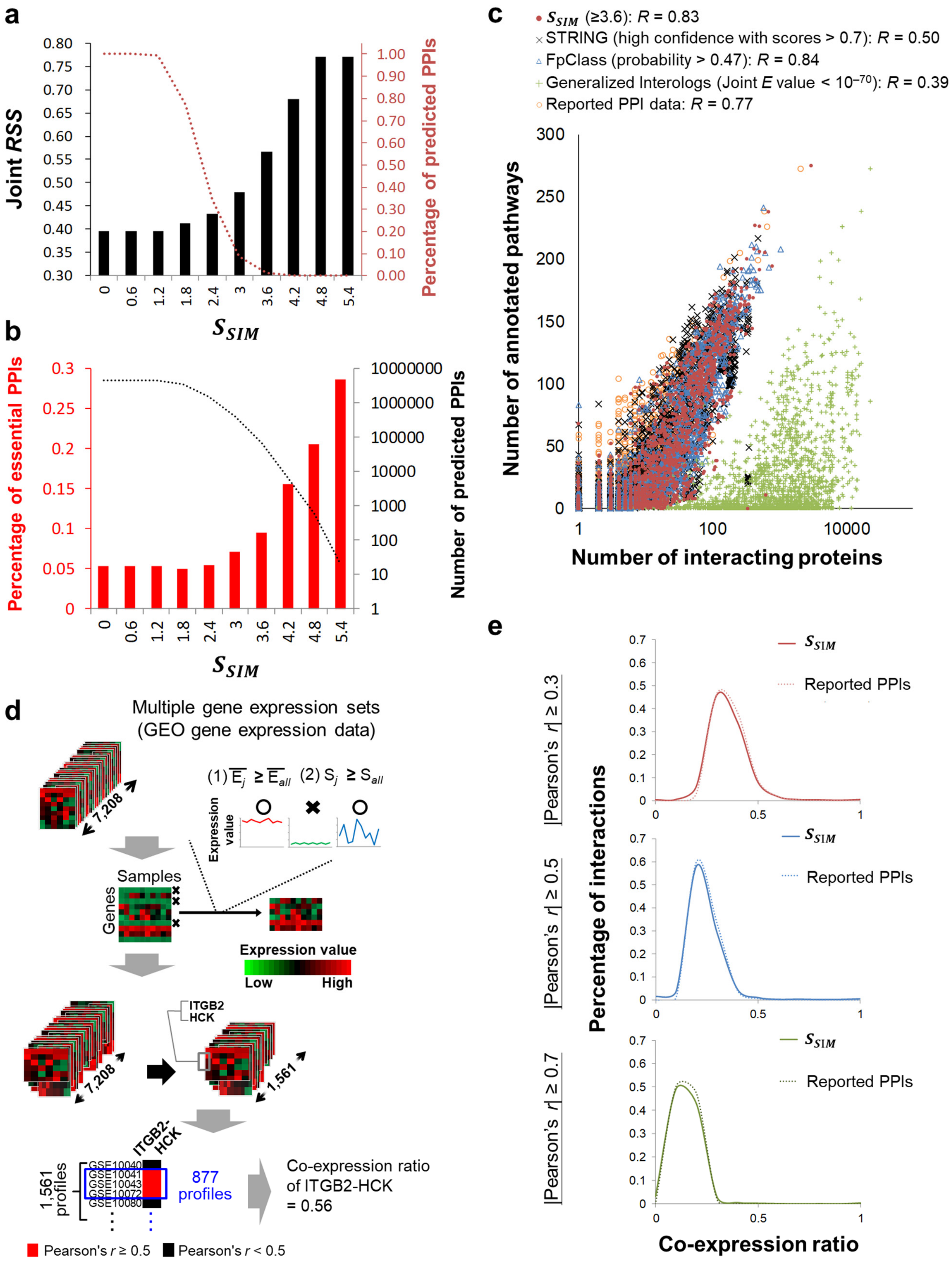

a
Joint RSS
Percentage of predicted PPIs
S_SIM
b
Percentage of essential PPIs
Number of predicted PPIs
S_SIM
c
S_SIM (≥3.6): R = 0.83
STRING (high confidence with scores > 0.7): R = 0.50
FpClass (probability > 0.47): R = 0.84
Generalized Interologs (Joint E value < 10^−70): R = 0.39
Reported PPI data: R = 0.77
Number of annotated pathways
Number of interacting proteins
d
Multiple gene expression sets
(GEO gene expression data)
7,208
(1) E_j ≥ E_all (2) S_j ≥ S_all
Expression value
Samples
Genes
Expression value
Low
High
ITGB2
HCK
7,208
1,561
ITGB2-HCK
1,561 profiles
GSE10040
GSE10041
GSE10043
GSE10072
GSE10080
877 profiles
Co-expression ratio of ITGB2-HCK = 0.56
Pearson's r ≥ 0.5
Pearson's r < 0.5
e
S_SIM
Reported PPIs
|Pearson's r| ≥ 0.3
|Pearson's r| ≥ 0.5
|Pearson's r| ≥ 0.7
Percentage of interactions
Co-expression ratio

Supplementary Figure 7. Biological function analysis for the systematically integrated method (SIM) strategy.

(**a**) Relationships between S_{SIM} values and joint RSS scores as well as percentages of PPI candidates in the MP set. (**b**) Relationships between S_{SIM} values scores against percentages of essential PPIs or numbers of PPI candidates in the MP set. (**c**) Relationships between the numbers of interacting proteins and annotated KEGG pathways for reported PPIs and predicted PPIs identified by our method, STRING (high confidence > 0.7; black), FpClass (probability > 0.47; blue), and generalized interologs (joint E value < 10^{-70}; green). (**d**) Characteristics of co-expression for reported PPIs and predicted PPIs with $S_{SIM} \geq 3.6$ in the MP set. Gene co-expression of PPIs using 7,208 human gene sets from GEO. Here, we quantified the associations and dissociations of MPs with interacting protein candidates in time and space by assessing correlations between their expression profiles in 7,208 gene expression data sets (≥3 samples) derived from GEO. To avoid the influence of genes with low expression and variance, we selected the gene j in a gene expression set based on the following criteria: 1) average expression ($\overline{E_j}$) ≥ mean expression of all genes ($\overline{E_{all}}$) in the gene expression set, and 2) standard deviation of expression (S_j) ≥ standard deviation of expression values for all genes (S_{all}) in the gene expression set. For each protein pair (e.g., an MP and its interacting protein candidate), we collected expression profiles containing the gene expression values of the two proteins and then calculated Pearson's r values for this pair to construct a correlation matrix. We assume that an MP interacts with another protein to perform biological functions in a cell if Pearson's $r \geq h$ is high (here, h was set to 0.3, 0.5, or 0.7). For a protein pair p (containing genes i and j), the co-expression ratio (CE) at the threshold h is defined as $CE_p^h = \frac{N_p}{N}$, where N is the total number of the 7,208 expression profiles with these two genes, and N_p is the number of expression profiles containing high co-expression of genes i and j with Pearson's r values ≥ h. For example, the CE of ITGB2–HCK is 0.56, reflecting high co-expression (Pearson's $r \geq 0.5$) in 877 of 1,561 gene expression sets when h = 0.5. (**e**) Distributions of

co-expression of reported PPIs and predicted PPIs with $S_{SIM} \geq 3.6$ based on Pearson's r thresholds of ≥ 0.3 (red), ≥ 0.5 (blue) and ≥ 0.7 (green).

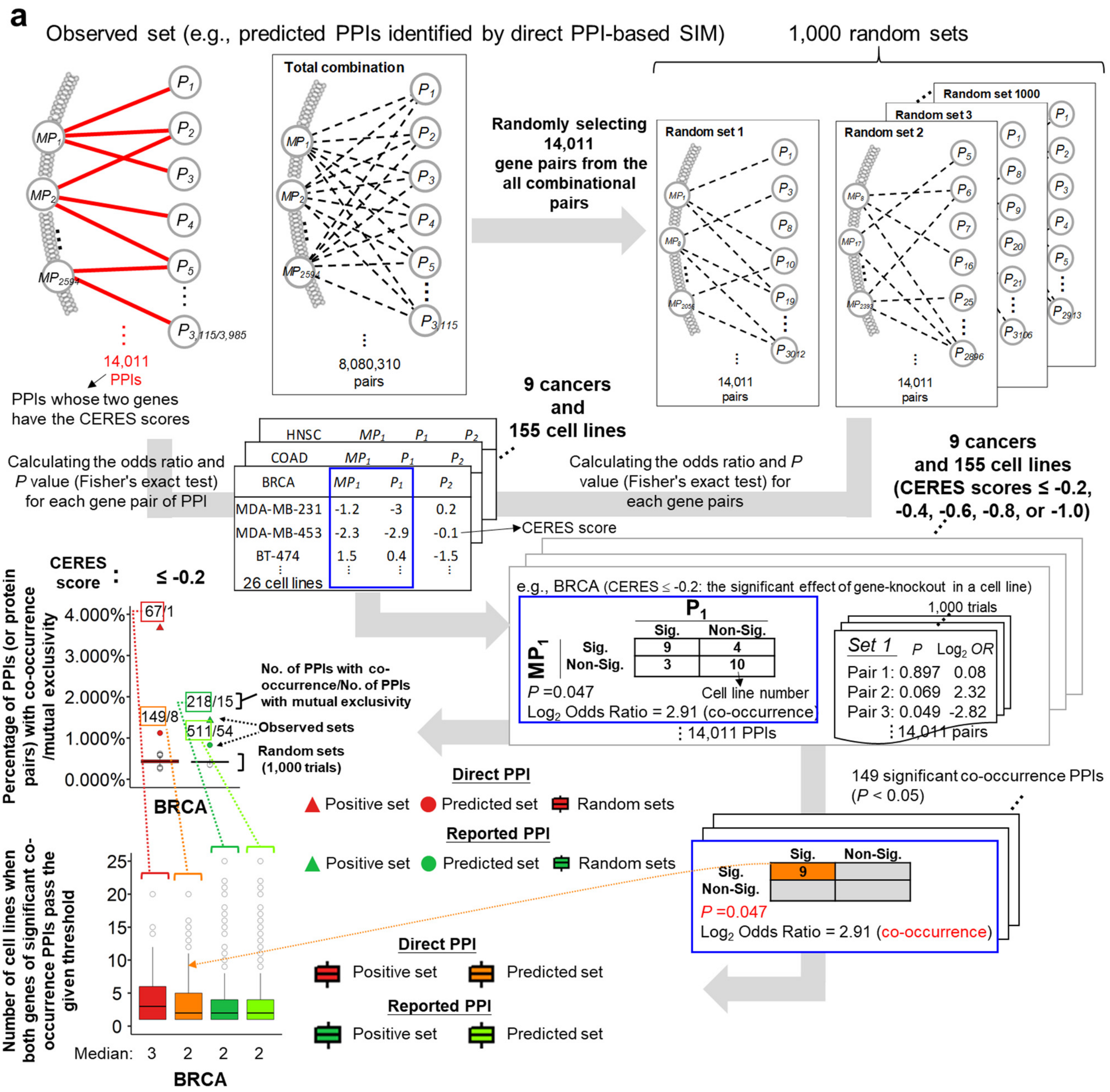
a
Observed set (e.g., predicted PPIs identified by direct PPI-based SIM)
1,000 random sets
Total combination
8,080,310 pairs
Randomly selecting 14,011 gene pairs from the all combinational pairs
Random set 1
Random set 2
Random set 3
Random set 1000
14,011 pairs
14,011 PPIs
PPIs whose two genes have the CERES scores
9 cancers and 155 cell lines
Calculating the odds ratio and P value (Fisher's exact test) for each gene pair of PPI
Calculating the odds ratio and P value (Fisher's exact test) for each gene pairs
9 cancers and 155 cell lines (CERES scores ≤ -0.2, -0.4, -0.6, -0.8, or -1.0)
HNSC
COAD
BRCA
MDA-MB-231
MDA-MB-453
BT-474
26 cell lines
CERES score
e.g., BRCA (CERES ≤ -0.2: the significant effect of gene-knockout in a cell line)
Sig.
Non-Sig.
Cell line number
P =0.047
Log2 Odds Ratio = 2.91 (co-occurrence)
1,000 trials
Set 1 P Log2 OR
Pair 1: 0.897 0.08
Pair 2: 0.069 2.32
Pair 3: 0.049 -2.82
14,011 pairs
CERES score : ≤ -0.2
Percentage of PPIs (or protein pairs) with co-occurrence /mutual exclusivity
No. of PPIs with co-occurrence/No. of PPIs with mutual exclusivity
Observed sets
Random sets (1,000 trials)
BRCA
Direct PPI
Positive set
Predicted set
Random sets
Reported PPI
149 significant co-occurrence PPIs (P < 0.05)
Number of cell lines when both genes of significant co-occurrence PPIs pass the given threshold
Median: 3 2 2 2

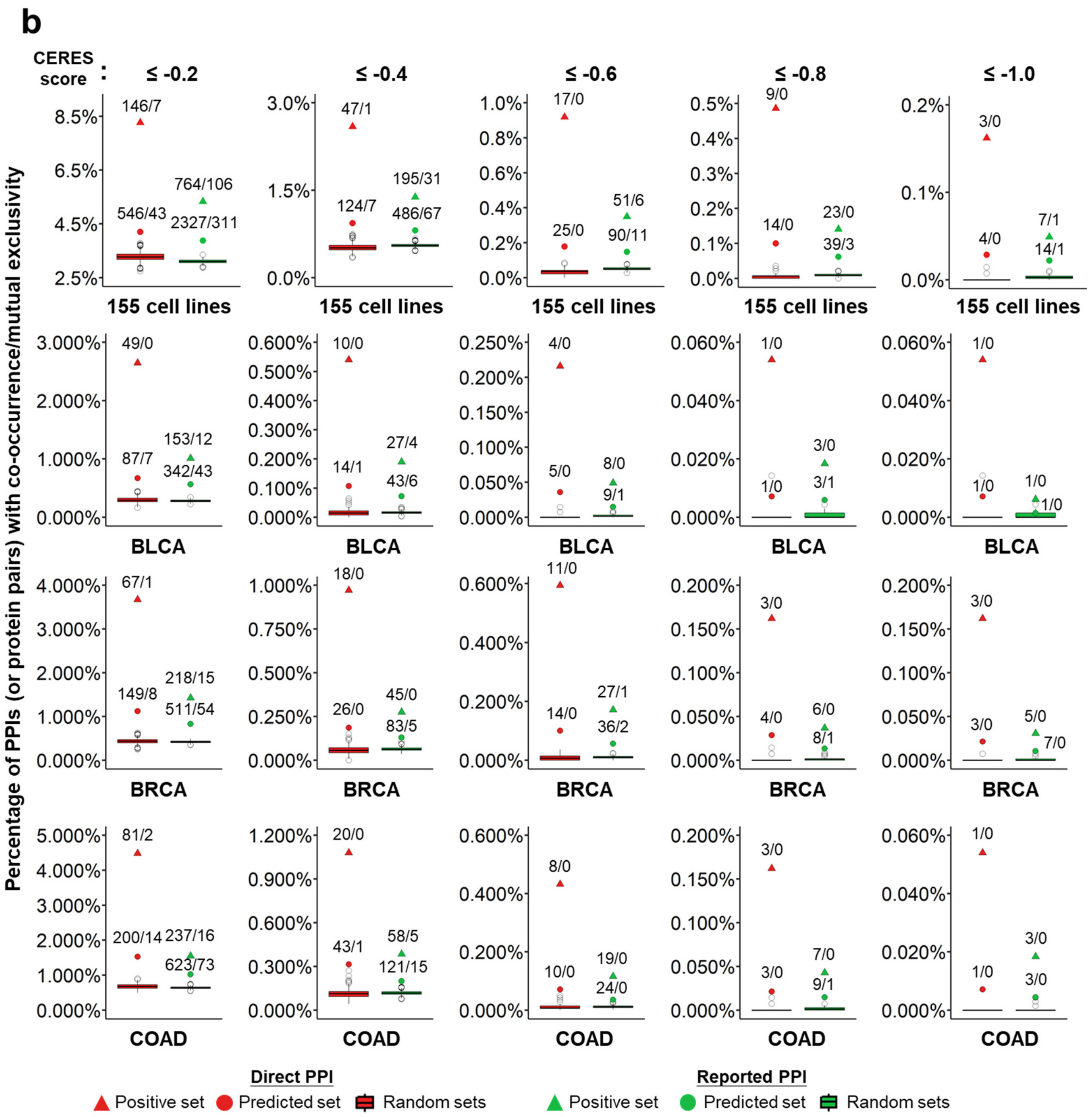
b
CERES score :
≤ -0.2
≤ -0.4
≤ -0.6
≤ -0.8
≤ -1.0
Percentage of PPIs (or protein pairs) with co-occurrence/mutual exclusivity
155 cell lines
BLCA
BRCA
COAD
Direct PPI
Positive set
Predicted set
Random sets
Reported PPI
Positive set
Predicted set
Random sets

b (continued)

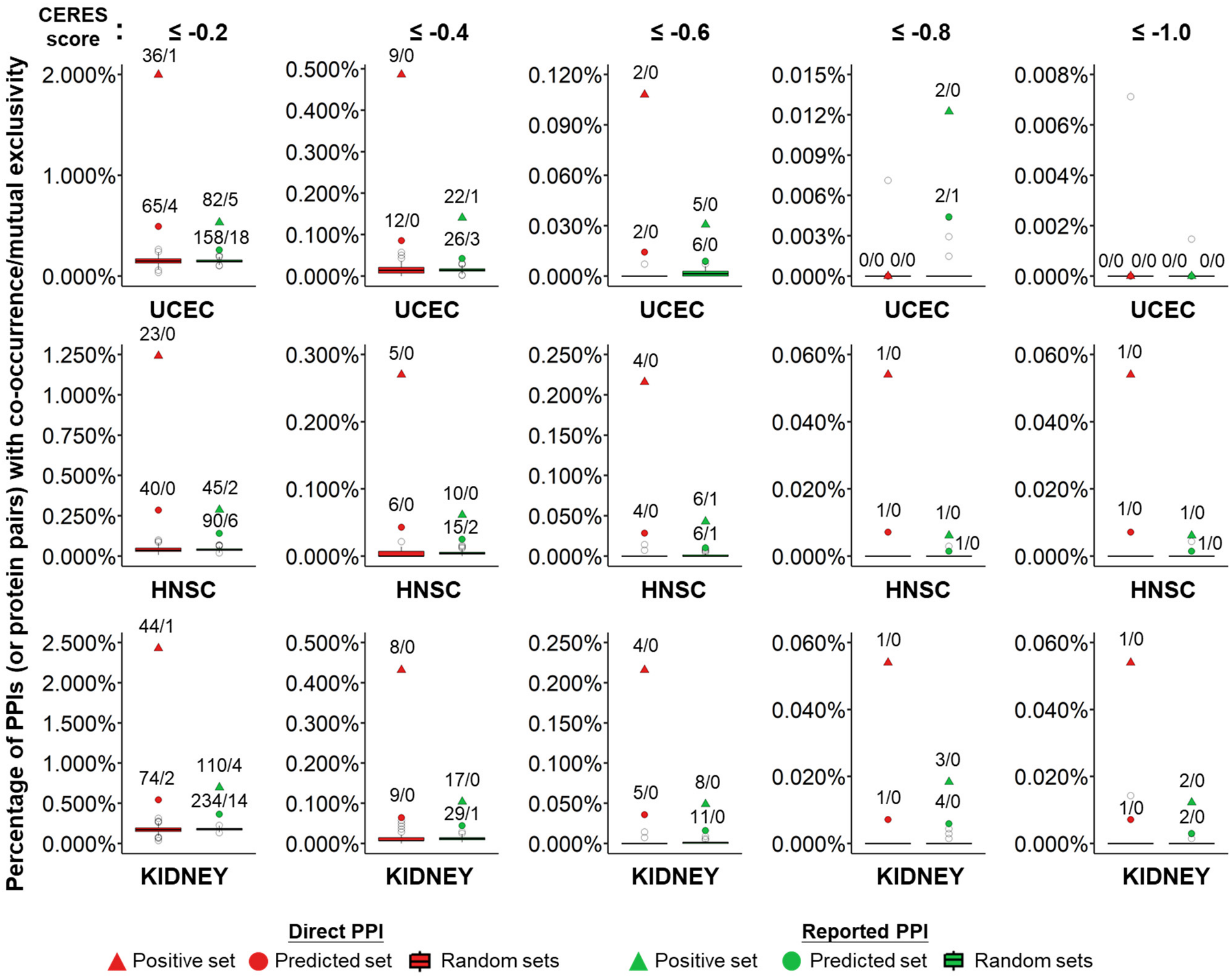
CERES score :
≤ -0.2
≤ -0.4
≤ -0.6
≤ -0.8
≤ -1.0
Percentage of PPIs (or protein pairs) with co-occurrence/mutual exclusivity
UCEC
HNSC
KIDNEY
Direct PPI
Positive set
Predicted set
Random sets
Reported PPI
Positive set
Predicted set
Random sets

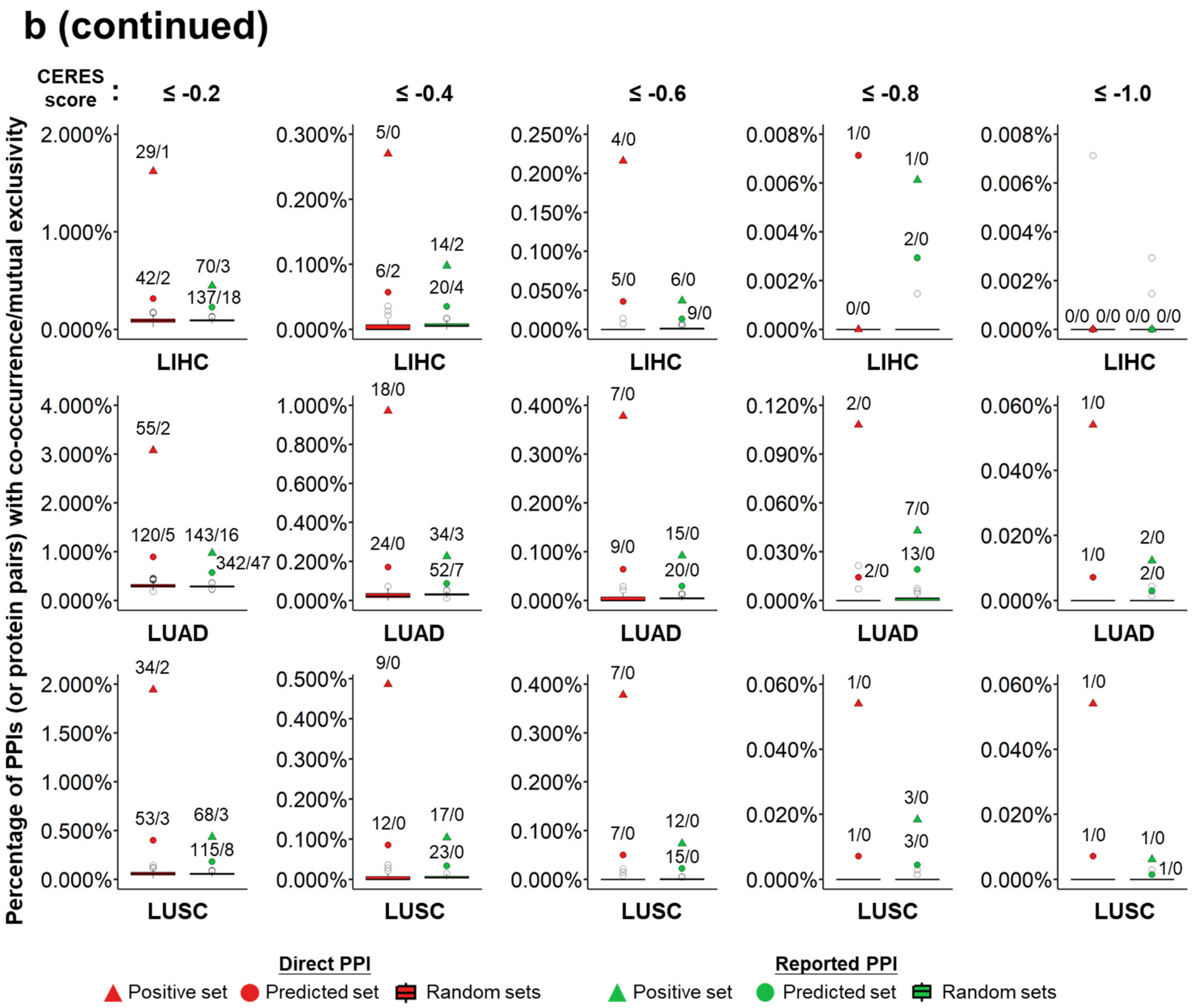
b (continued)
CERES score :
≤ -0.2
≤ -0.4
≤ -0.6
≤ -0.8
≤ -1.0
Percentage of PPIs (or protein pairs) with co-occurrence/mutual exclusivity
LIHC
LUAD
LUSC
Direct PPI
Positive set
Predicted set
Random sets
Reported PPI
Positive set
Predicted set
Random sets

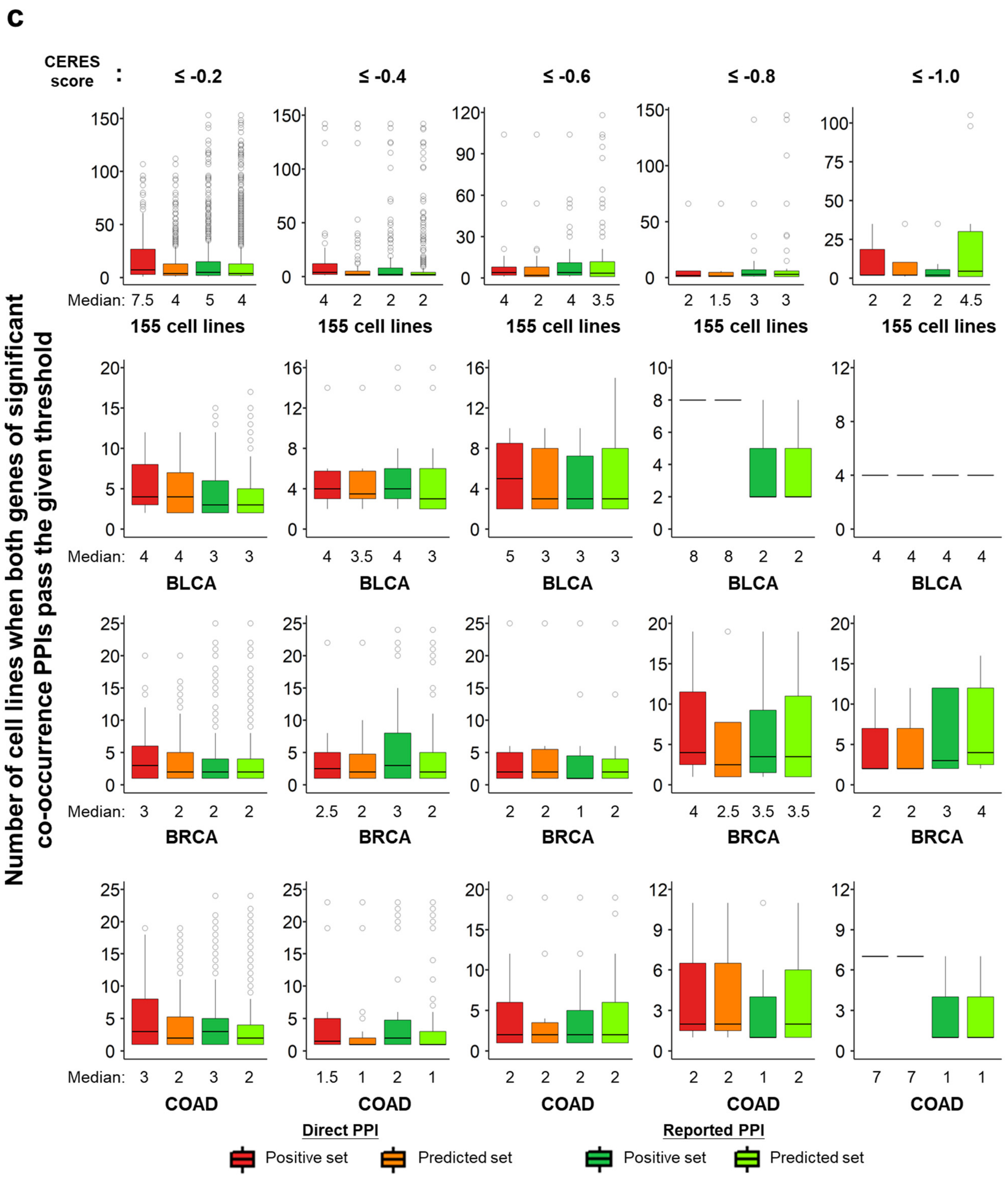
c
CERES score :
≤ -0.2
≤ -0.4
≤ -0.6
≤ -0.8
≤ -1.0
Number of cell lines when both genes of significant co-occurrence PPIs pass the given threshold
Median: 7.5 4 5 4
4 2 2 2
4 2 4 3.5
2 1.5 3 3
2 2 2 4.5
155 cell lines
Median: 4 4 3 3
4 3.5 4 3
5 3 3 3
8 8 2 2
4 4 4 4
BLCA
Median: 3 2 2 2
2.5 2 3 2
2 2 1 2
4 2.5 3.5 3.5
2 2 3 4
BRCA
Median: 3 2 3 2
1.5 1 2 1
2 2 2 2
2 2 1 2
7 7 1 1
COAD
Direct PPI
Positive set
Predicted set
Reported PPI
Positive set
Predicted set

c (continued)

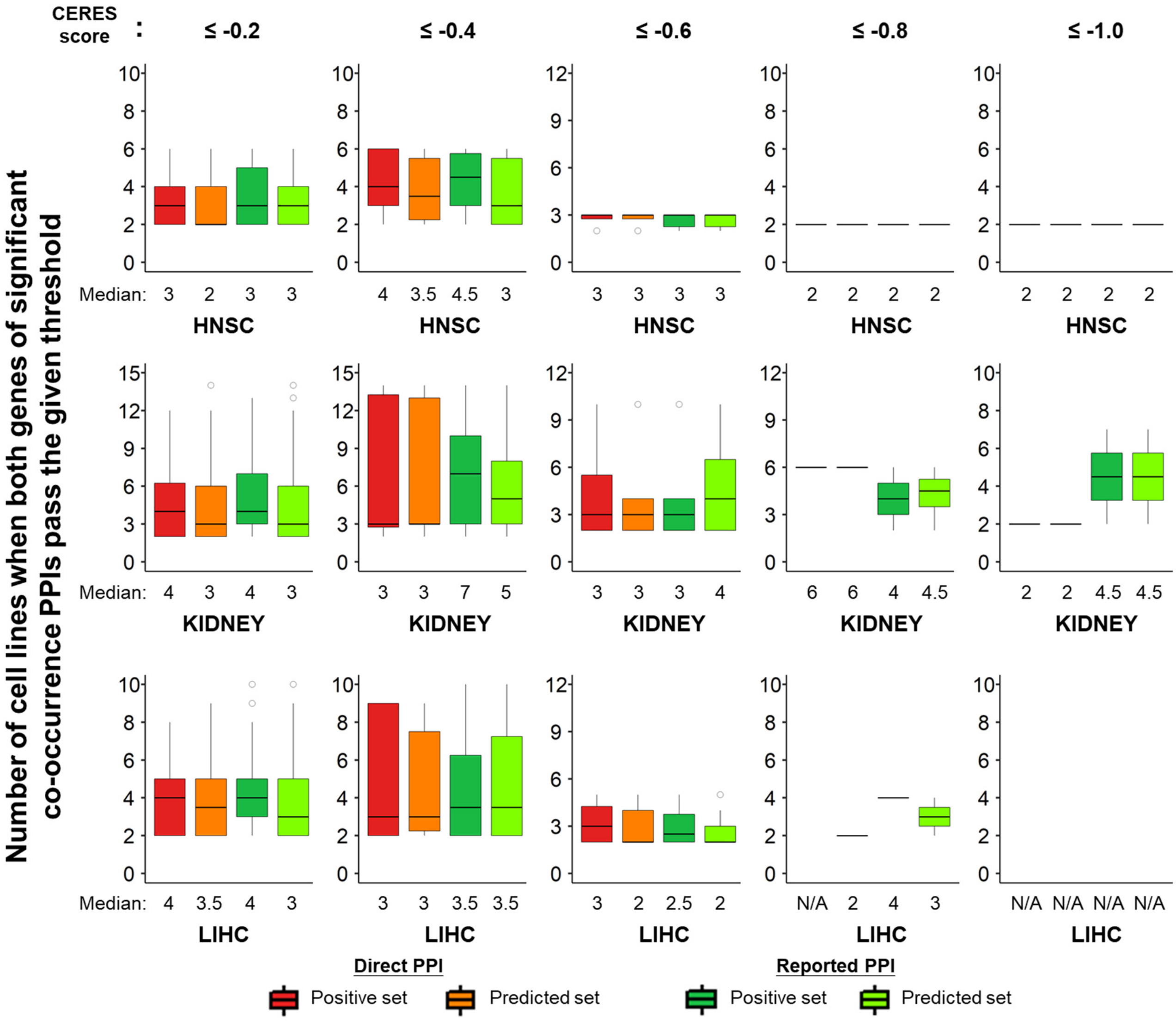
CERES score :
≤ -0.2
≤ -0.4
≤ -0.6
≤ -0.8
≤ -1.0
Number of cell lines when both genes of significant co-occurrence PPIs pass the given threshold
Median: 3 2 3 3
HNSC
4 3.5 4.5 3
3 3 3 3
2 2 2 2
2 2 2 2
Median: 4 3 4 3
KIDNEY
3 3 7 5
3 3 3 4
6 6 4 4.5
2 2 4.5 4.5
Median: 4 3.5 4 3
LIHC
3 3 3.5 3.5
3 2 2.5 2
N/A 2 4 3
N/A N/A N/A N/A
Direct PPI
Positive set
Predicted set
Reported PPI
Positive set
Predicted set

c (continued)

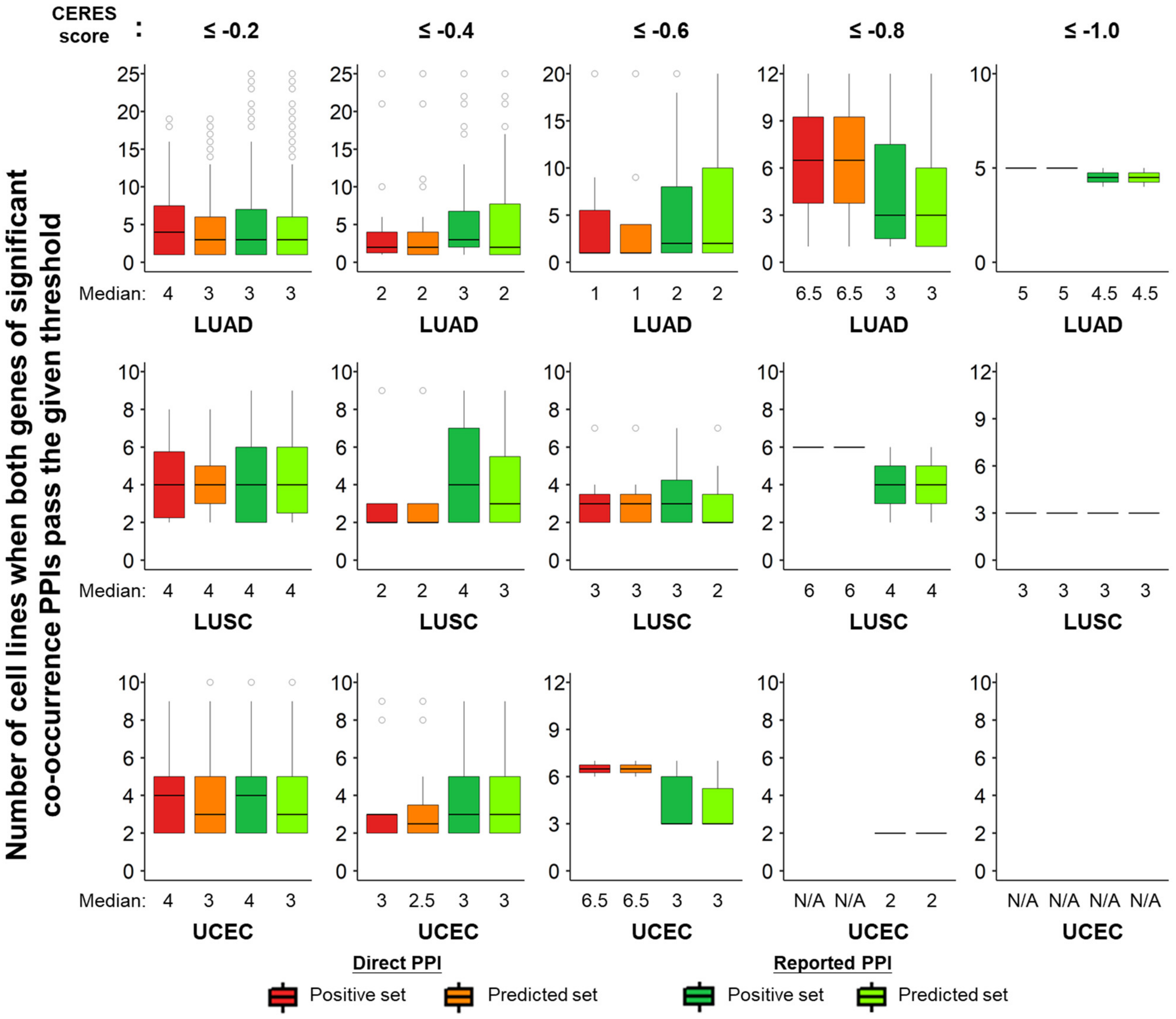

Supplementary Figure 8. Analysis of functional connectivity between the MPs and their positive/predicted binding partners using the experimental data of the loss-of-function screens from the Project Achilles data.

(**a**) Schematic diagram depicting the identification of mutual exclusivity or co-occurrence of loss-of-function effects between the MPs and their positive/predicted binding partners using the Project Achilles data in nine cancer types and 155 cell lines. Here, only PPIs/protein pairs whose two genes have CERES scores were analyzed. For each positive/predicted MP PPI set, we randomly sampled 14,011 (or 68,059 for reported PPI sets) gene pairs from a total of 8,080,310 (or 22,041,218 for reported PPI sets) combinational pairs in 1,000 trials and then calculated the odds ratio and *P* value

(Fisher's exact test) between two gene-knockout effect profiles across numerous cell lines in each cancer type to evaluate the empirical *P* value in comparison to the observed set. (**b**) Significance of percentage of PPIs (or protein pairs) with co-occurrence/mutual exclusivity in nine cancer types and the group of 155 cancer cell lines across five CERES score thresholds for 14,011 (red circle) and 68,059 (green circle) MP predicted PPIs using direct PPI- and reported PPI-based SIMs, respectively, and for 1,851 MP direct PPIs (red triangle) and 16,334 MP recorded PPIs (green triangle) as positive control sets. The boxplots show the percentages of gene pairs in the corresponding random sets. The numbers that are shown near the circles and triangles indicate the number of PPIs with significant co-occurrence and mutual exclusivity, respectively. (**c**) Distribution of cell line numbers for significant co-occurrence PPIs ($P < 0.05$, Fisher's exact test) whose two genes have CERES scores ≤ -0.2, -0.4, -0.6, -0.8, or -1.0, which indicates that they have significant knockout effects. The median values of cell line numbers are shown below each plot, and N/A represents no significant co-occurrence PPI. The box represents the interquartile range (IQR) and the horizontal line in the box is the median. The whiskers denote the lowest and highest values within 1.5 times IQR from the first and third quartiles, respectively. The external circles represent outliers.

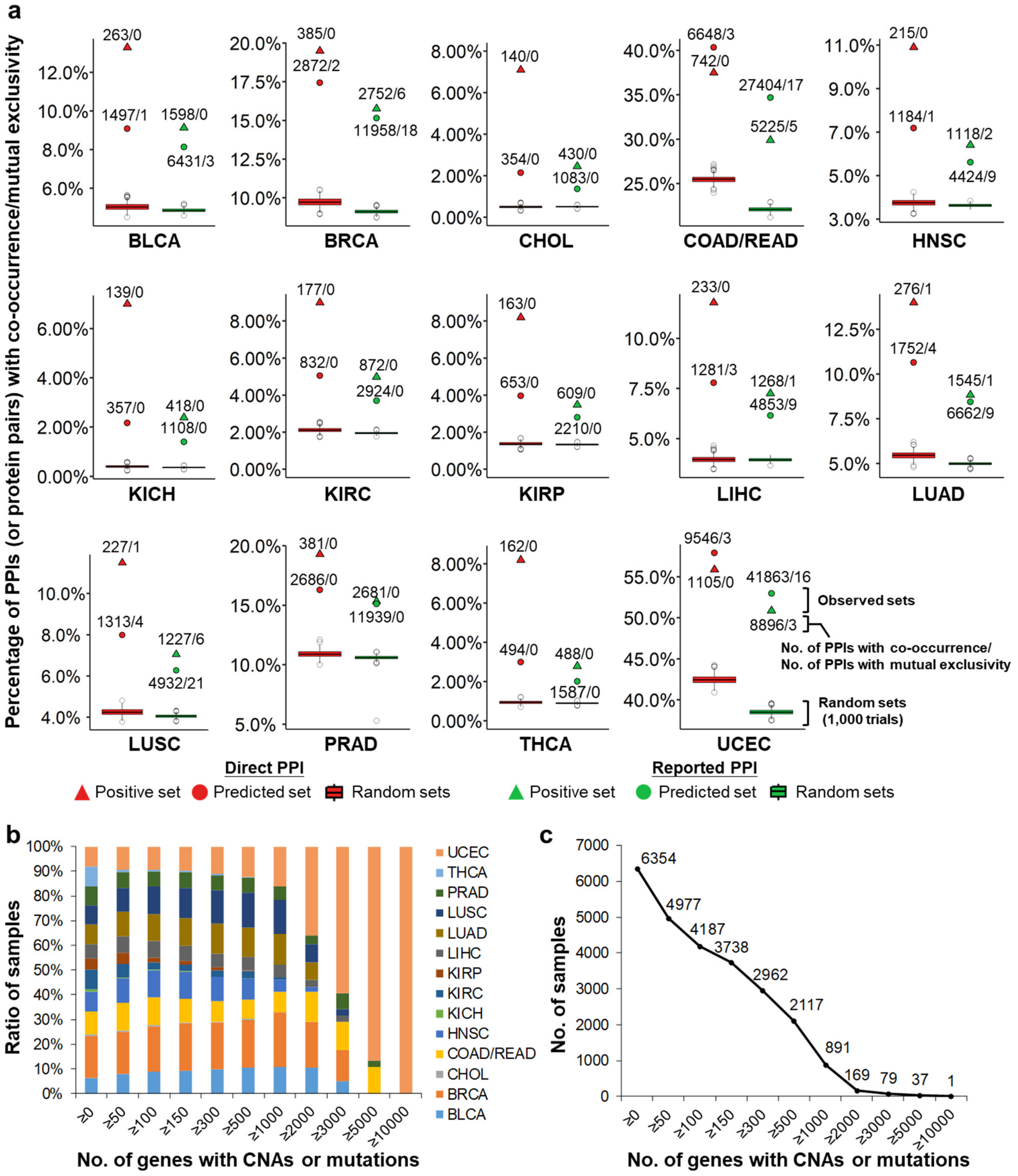
a
Percentage of PPIs (or protein pairs) with co-occurrence/mutual exclusivity
BLCA
BRCA
CHOL
COAD/READ
HNSC
KICH
KIRC
KIRP
LIHC
LUAD
LUSC
PRAD
THCA
UCEC
Observed sets
No. of PPIs with co-occurrence/
No. of PPIs with mutual exclusivity
Random sets
(1,000 trials)
Direct PPI
Positive set
Predicted set
Random sets
Reported PPI
Positive set
Predicted set
Random sets
b
Ratio of samples
UCEC
THCA
PRAD
LUSC
LUAD
LIHC
KIRP
KIRC
KICH
HNSC
COAD/READ
CHOL
BRCA
BLCA
No. of genes with CNAs or mutations
c
No. of samples
6354
4977
4187
3738
2962
2117
891
169
79
37
1
No. of genes with CNAs or mutations

Supplementary Figure 9. Analysis of co-occurrence and mutual exclusivity of genomic alterations between the MPs and their positive/predicted binding partners using the copy-number alteration (CNA) and mutation data from the TCGA.

(a) Significance of percentages of PPIs with co-occurrence and mutual exclusivity of genomic alterations in 14 cancer types for 16,269 (red circle) and 78,569 (green circle) MP predicted PPIs using direct PPI- and reported PPI-based SIMs, respectively, and for 1,851 MP direct PPIs (red triangle) and 17,203 MP reported PPIs (green triangle) as positive control sets. Here, only PPIs/protein pairs whose two genes could be mapped from UniProt accession number to the Entrez Gene ID used in TCGA data were analyzed. The boxplots show the percentages of gene pairs in the corresponding random sets. The box represents the IQR and the horizontal line in the box is the median. The whiskers denote the lowest and highest values within 1.5 times IQR from the first and third quartiles, respectively. The external circles represent outliers. The numbers that are shown near the circles and triangles indicate the number of PPIs with significant co-occurrence and mutual exclusivity, respectively. (**b**) Relationships between the number of genes with CNAs or mutations and the ratio of samples in 14 different cancer types. (**c**) Relationships between the number of genes with CNAs or mutations and the number of samples.

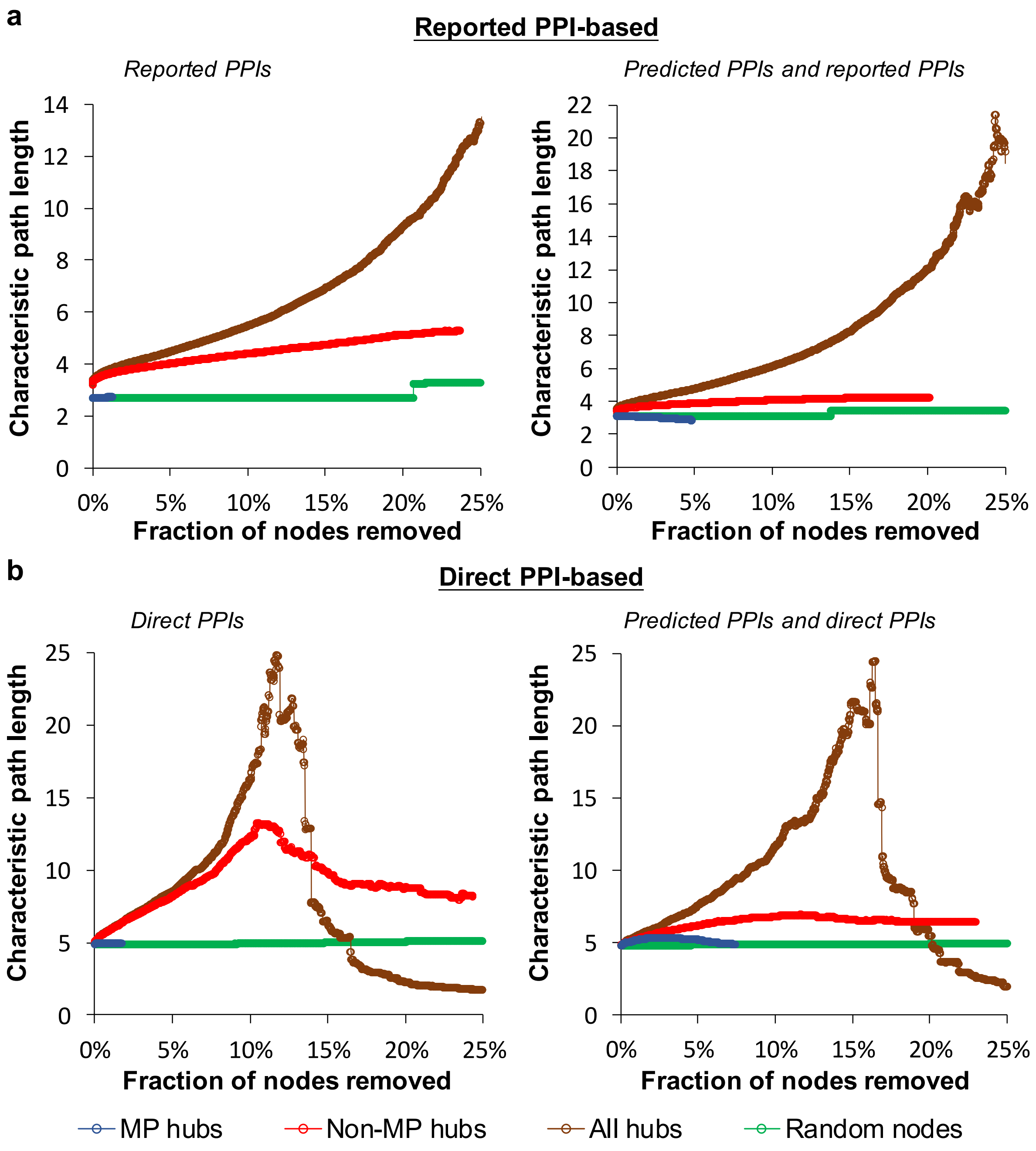

Supplementary Figure 10. Effect on the characteristic path length of the networks upon gradual node removal for the (a) reported PPI and (b) direct PPI networks without (left) or with (right) predicted PPIs.

Attacks against all hubs (brown curve), MP hubs (blue curve), non-MP hubs (red curve), and random nodes (green curve) are shown. The proteins with degrees within the top 25% of all proteins are considered hubs[16] of these human networks. Here, the degrees of hubs in the reported PPI network

(179,249 PPIs and 16,385 proteins) containing only reported PPIs, the reported PPI network-related (291,515 PPIs and 25,907 proteins) containing both reported PPI-based predicted PPIs and reported PPIs, the direct PPI network (14,549 PPIs and 6,326 proteins) containing only direct physical PPIs, and the direct PPI-related network (39,396 PPIs and 11,122 proteins) containing both direct PPI-based predicted PPIs and direct physical PPIs were ≥19, ≥17, ≥5, and ≥6, respectively.

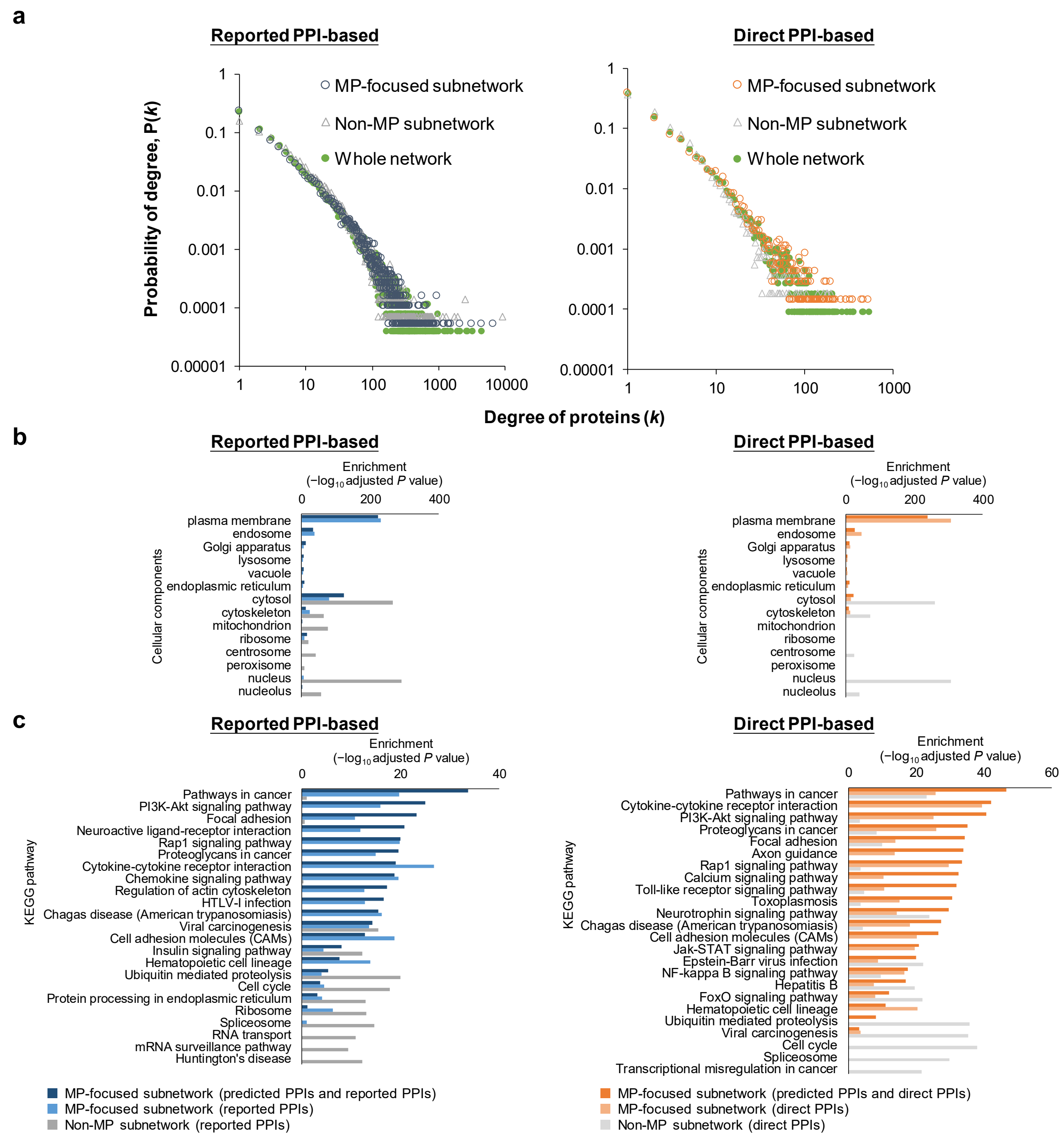

Supplementary Figure 11. Topology and functional enrichment analysis of MP-focused subnetworks and non-MP subnetworks.

(**a**) Degree distributions of the MP-focused PPI subnetworks, the non-MP PPI subnetworks, and the whole human PPI networks illustrating scale-free topology. In these subnetworks and networks, all of the degree exponent (γ) values ranged between 1.42 and 1.78. The human PPI networks include reported PPIs and predicted PPIs of MPs, identified by the reported PPI-based SIM (left) or the direct

PPI-based SIM (right). The MP-focused subnetworks, containing 239,112 PPIs (reported PPI-based) and 29,444 PPIs (direct PPI-based), were constructed by extending one layer of PPIs and proteins in 2,594 MPs from the whole human PPI network. The non-MP subnetwork (only derived from reported/direct PPI data), consisting of 160,715 reported PPIs and 12,626 direct PPIs, comprised the whole human PPI network excluding all MPs and their PPIs. (**b**) Cellular component and (**c**) KEGG pathway enrichment analyses of all proteins of the MP-focused subnetworks, established by reported/direct PPIs with or without predicted PPIs of MPs, and the non-MP subnetworks. The *P* values were calculated based on the hypergeometric test with Bonferroni correction.

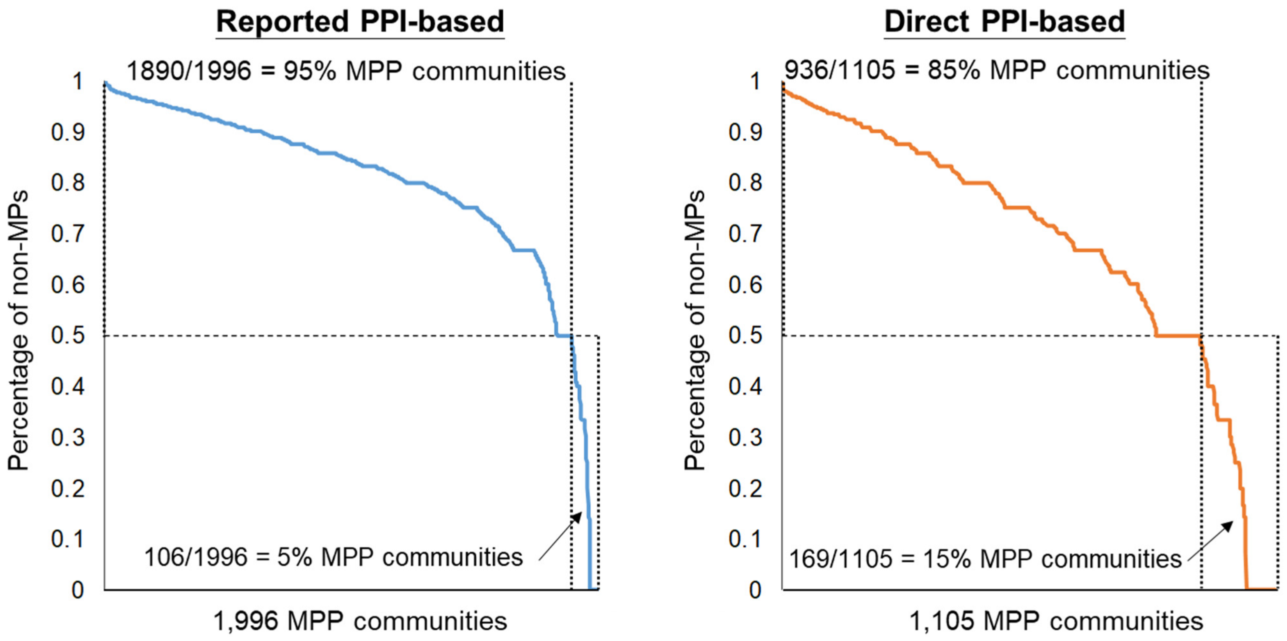

Supplementary Figure 12. Percentages of MP and non-MPs for 1,996 reported PPI-based and 1,105 direct PPI-based MPP communities.

The orders of MPP communities are sorted by the percentage of non-MP proteins.

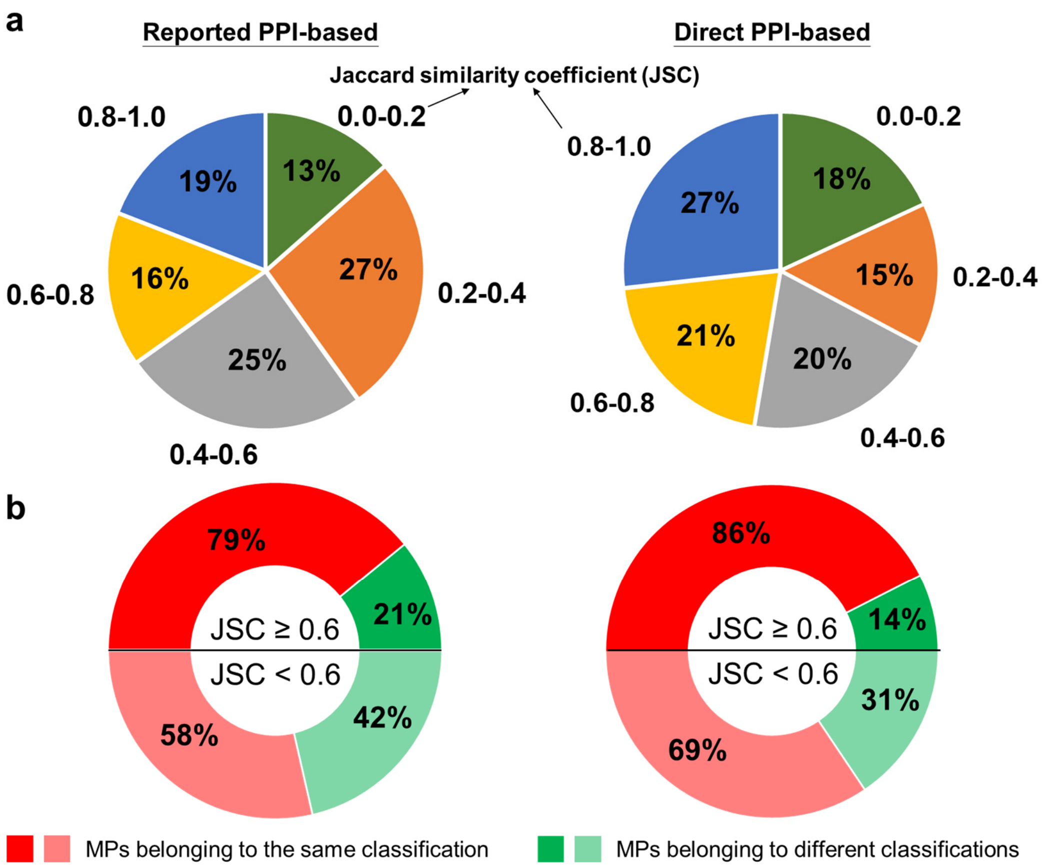

Supplementary Figure 13. Overlap analysis of the binding partners identified for different MPs by using reported PPI- and direct PPI-based SIM methods.

(**a**) Overlaps, evaluated by the Jaccard index, between the binding partners identified for any two MPs among 1,996 (reported PPI-based method) and 1,105 MPs (direct PPI-based method) with at least one binding partner. For each MPP community (i.e., an MP with its binding partners), we calculated the Jaccard similarity coefficient (JSC) to evaluate the overlap between it and the other MPP communities and select the largest overlapping community. The Jaccard similarity coefficient $\mathrm{J}(A,B)$ is given as $\mathrm{J}(A,B) = |A \cap B|/|A \cup B|$, where $A \cap B$ is the number of common binding partners (intersection set) in the MPP communities A and B, and $A \cup B$ is the number of union binding partners in the MPP communities A and B. (**b**) Pie charts of the percentages of MPs belonging to the same (red) or different (green) classifications (**Supplementary Data 1**) when their communities had the highest JSC ≥ 0.6 or < 0.6. Note that MPs belonging to the classification 'Unclassified' were excluded.

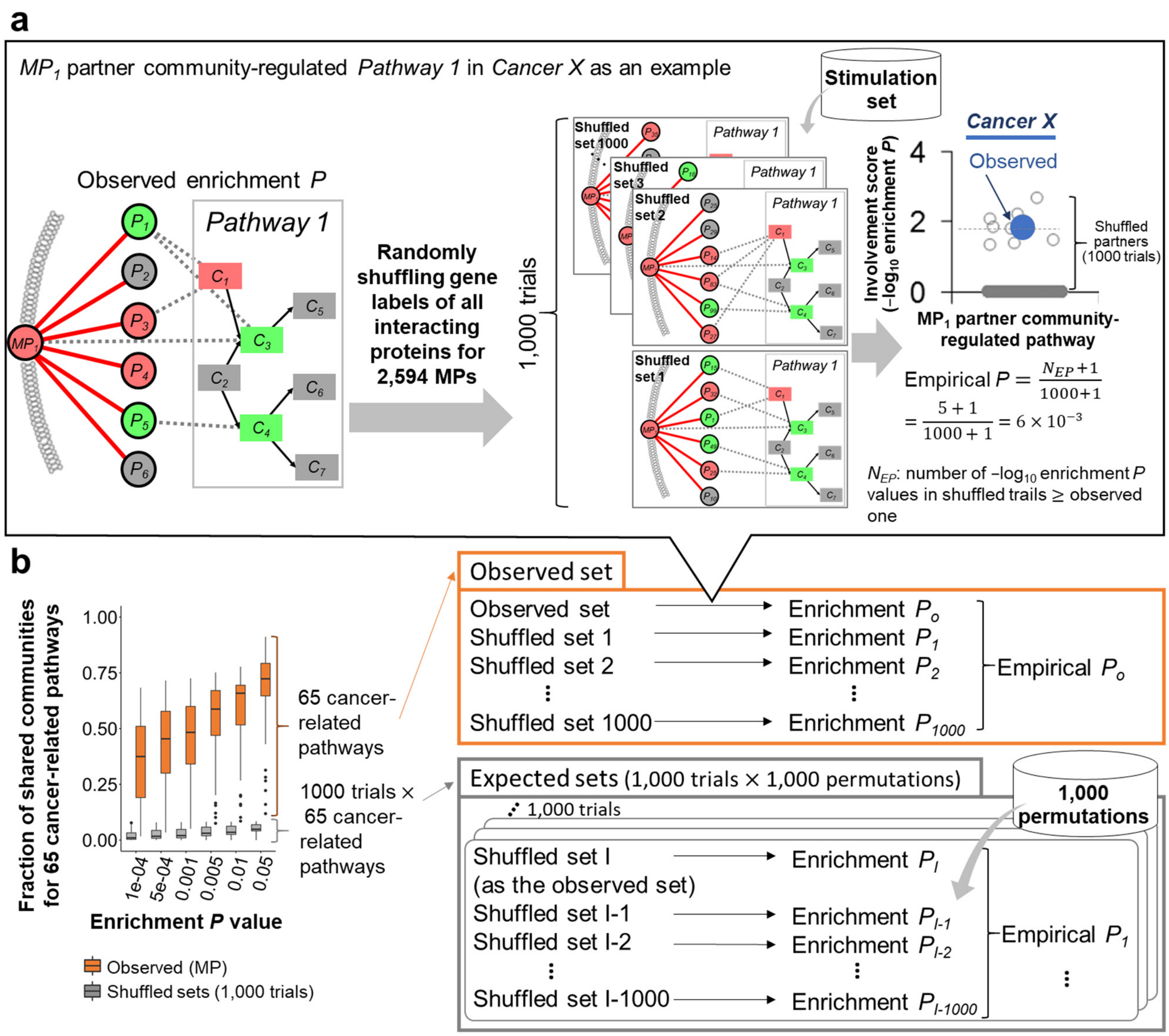

Supplementary Figure 14. Schematic diagram of empirical *P* value calculation and Monte Carlo simulation set preparation.

(**a**) Empirical *P* value calculation using an MPP community (*MP1*) and its regulated pathway (*Pathway 1*) in a target cancer (*Cancer X*) as an example. For each MPP community regulating a specific pathway (observed set), we randomly shuffled gene labels of all proteins interacting with 2,594 MPs in 1,000 trials and then calculated the enrichment *P* values for the 1,000 shuffled sets to evaluate the empirical *P* value. (**b**) Monte Carlo simulation set preparation for evaluating the significance of shared community-regulated pathways for 1,862 MPs and 197 MP families among 15 cancers. For each shuffled MPP community-regulated pathway (e.g., shuffled set I), we first evaluated its enrichment *P*

value, and the empirical *P* value was determined by 1,000 Monte Carlo permutations. Among 65 cancer-related pathways, the observed fractions of MPP communities were compared to the expected fractions of 1,000 trials (total of 1,000,000 permutations).

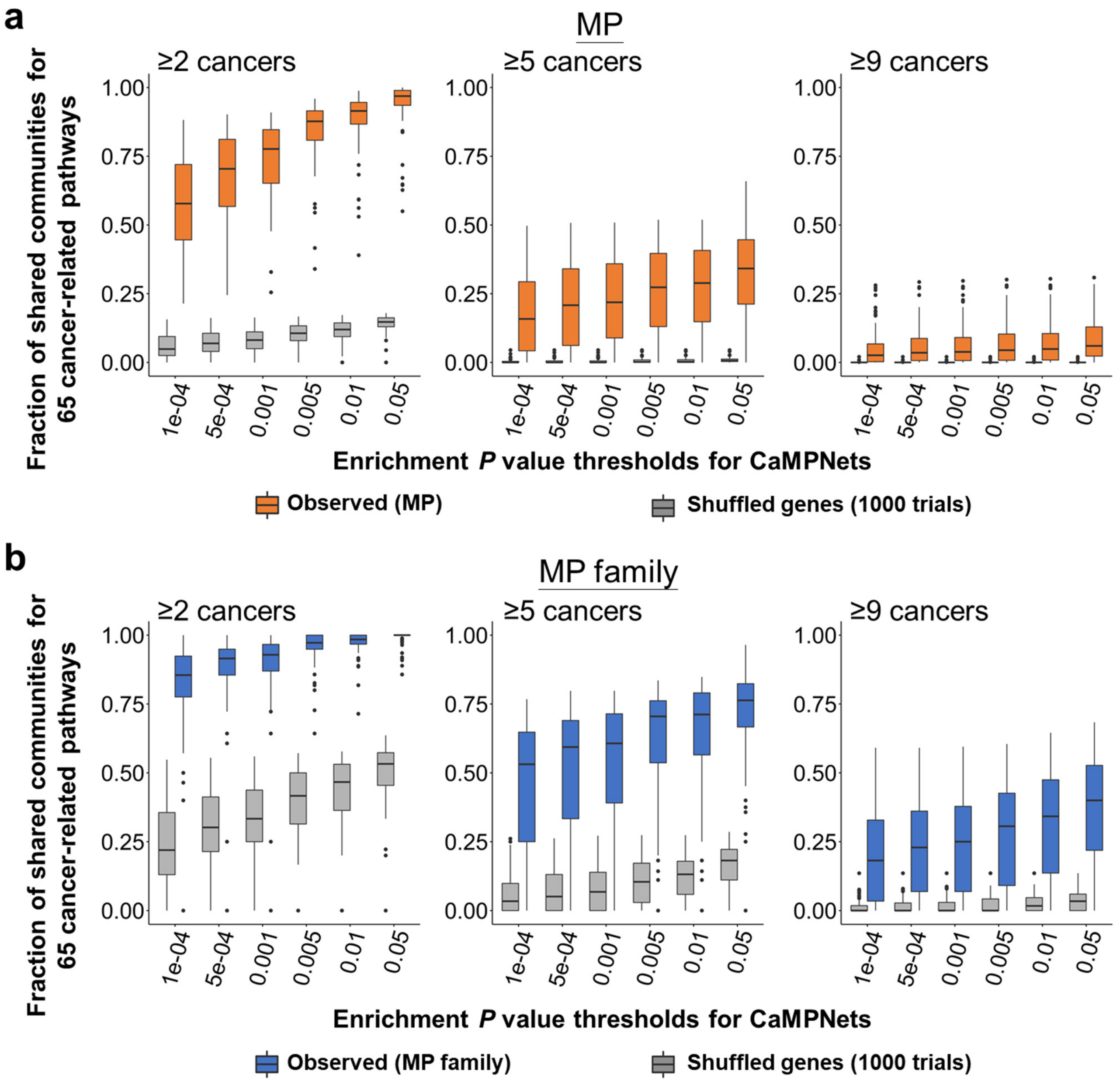

Supplementary Figure 15. Significance of shared community-regulated pathways in CaMPNets for (a) 1,862 MPs and (b) 197 MP families among 15 cancers.

Similar analyses are shown in **Figure 2b,c** compared to randomly shuffled gene labels for each gene of all proteins interacting with MPs in CaMPNets across different thresholds of enrichment *P* values (details in Methods). We then calculated the median fraction of shuffled communities for 65 cancer-related pathways (box) shared by at least 2, 5 or 9 cancers and repeated the analysis 1,000 times (**Supplementary Fig. 14b**). Based on the MP classification obtained from Almén *et al.*[67], the cancer types associated with community-regulated pathways (or shuffled pathways) for MPs of the same

family are united. Approximately 97% and 100% of communities for MPs (orange) and MP families (blue), respectively, are involved in certain cancer-related pathways (filtered for enrichment P values ≤ 0.05 and empirical P values ≤ 0.05) across ≥ 2 cancer types. Notably, significantly more shared community-regulated pathways on MPs (or MP families) were found than was expected by random chance (gray; $P < 1 \times 10^{-9}$, Wilcoxon signed-rank test) filtered at six thresholds of enrichment P values. The box represents the IQR and the horizontal line in the box is the median. The whiskers denote the lowest and highest values within 1.5 times IQR from the first and third quartiles, respectively. The black dots represent outliers.

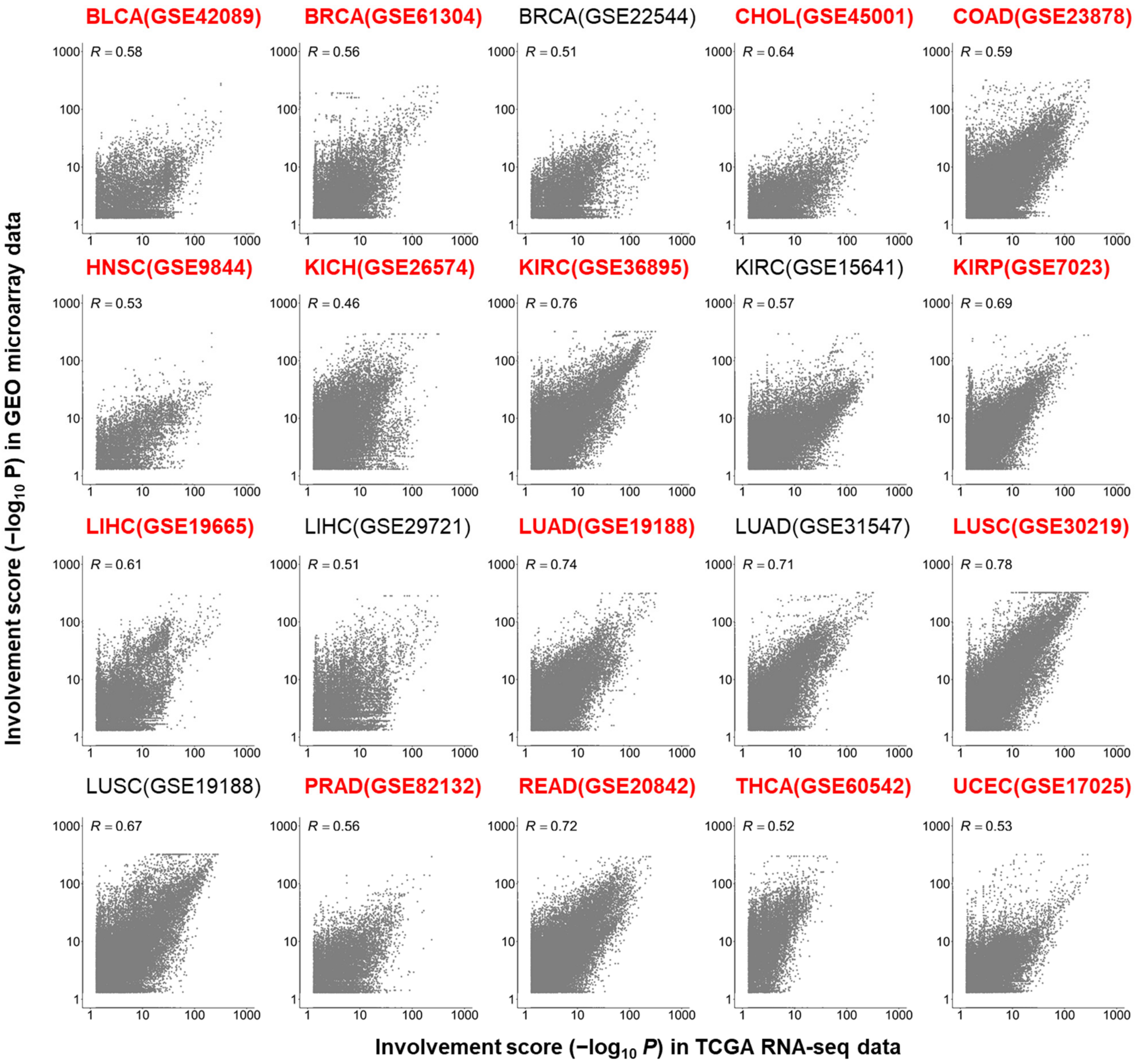

Supplementary Figure 16. Comparison of involvement scores for community-regulated pathways between TCGA RNA-seq and microarray (total of 19 sets) data sets in 15 individual cancer types.

Pearson's *r* values shown in the upper left corner of each plot represent the correlation. All correlations (*R*) are larger than 0.46, and the respective *P* values are less than 2.2×10^{-16} (Pearson correlation, *t*-test). To further validate the involvement scores of community-regulated pathways across 15 cancers by using different gene expression resources, the enrichment *P* values were transformed into *z*-scores

and utilized to calculate meta-*z*-scores (Stouffer's *Z*-transform method (unweighted); **Fig. 2d**) via selecting independent microarray sets with higher correlation (labeled red) in 15 cancers.

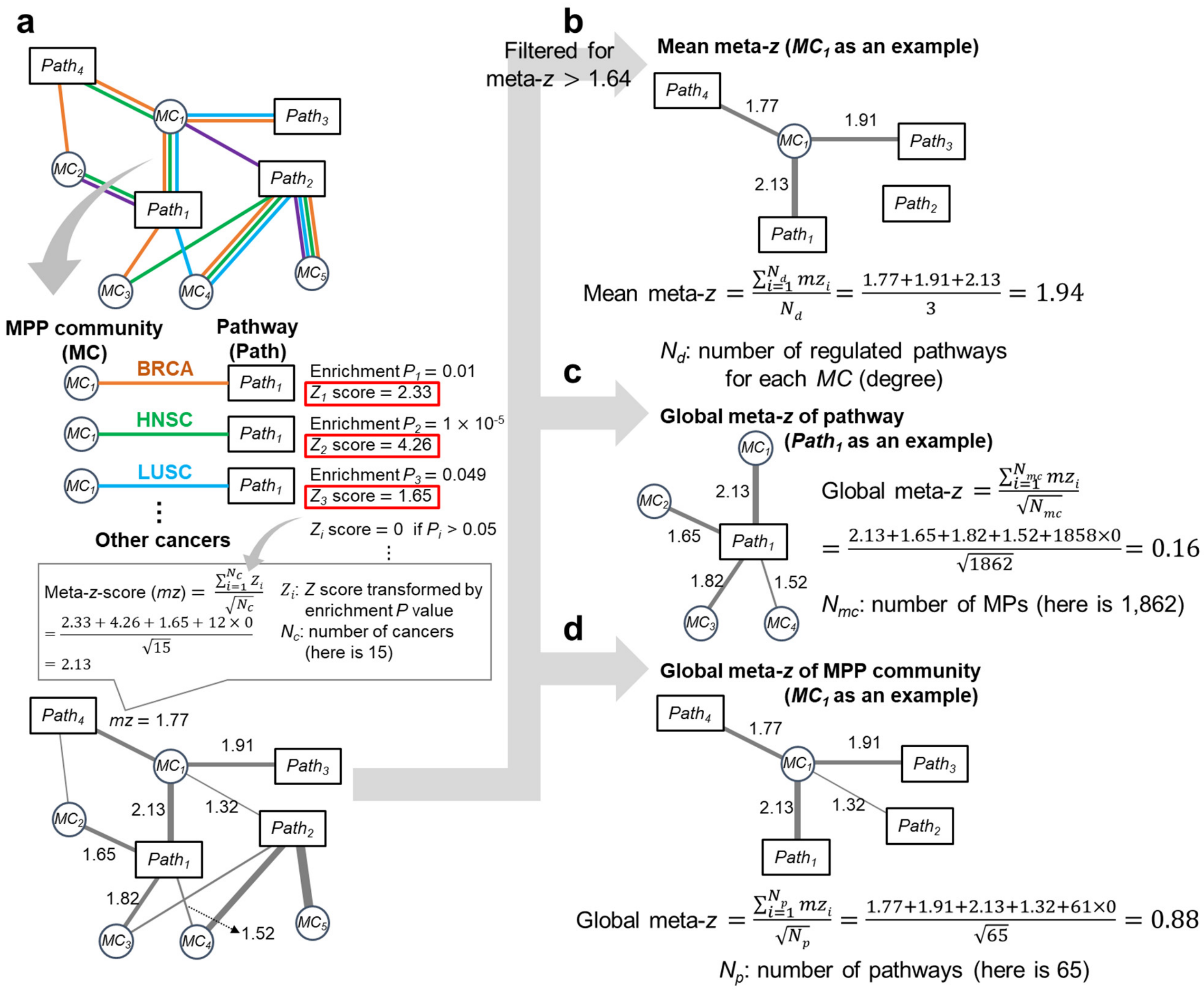

Supplementary Figure 17. Schematic diagram of calculating the (a) meta-*z*-score, (b) mean meta-*z*-score, and global meta-*z*-scores of (c) pathways and (d) MPP communities.

Enrichment *P* values for each community-regulated pathway of CaMPNets across 15 cancer types were transformed into *z*-scores and further combined into a meta-*z*-score using Stouffer's unweighted *Z*-transform test. For calculating the mean meta-*z* score of each community in the pan-cancer network of CaMPNets (filtered at meta-*z* > 1.64), we summed the meta-*z*-scores of the target community and its regulated pathways and then divided the sum by the number of regulated pathways. Based on the pan-cancer network of CaMPNets, global meta-*z*-scores of each pathway (or each community) were computed by considering the meta-*z*-scores of all 1,862 communities (or 65 pathways).

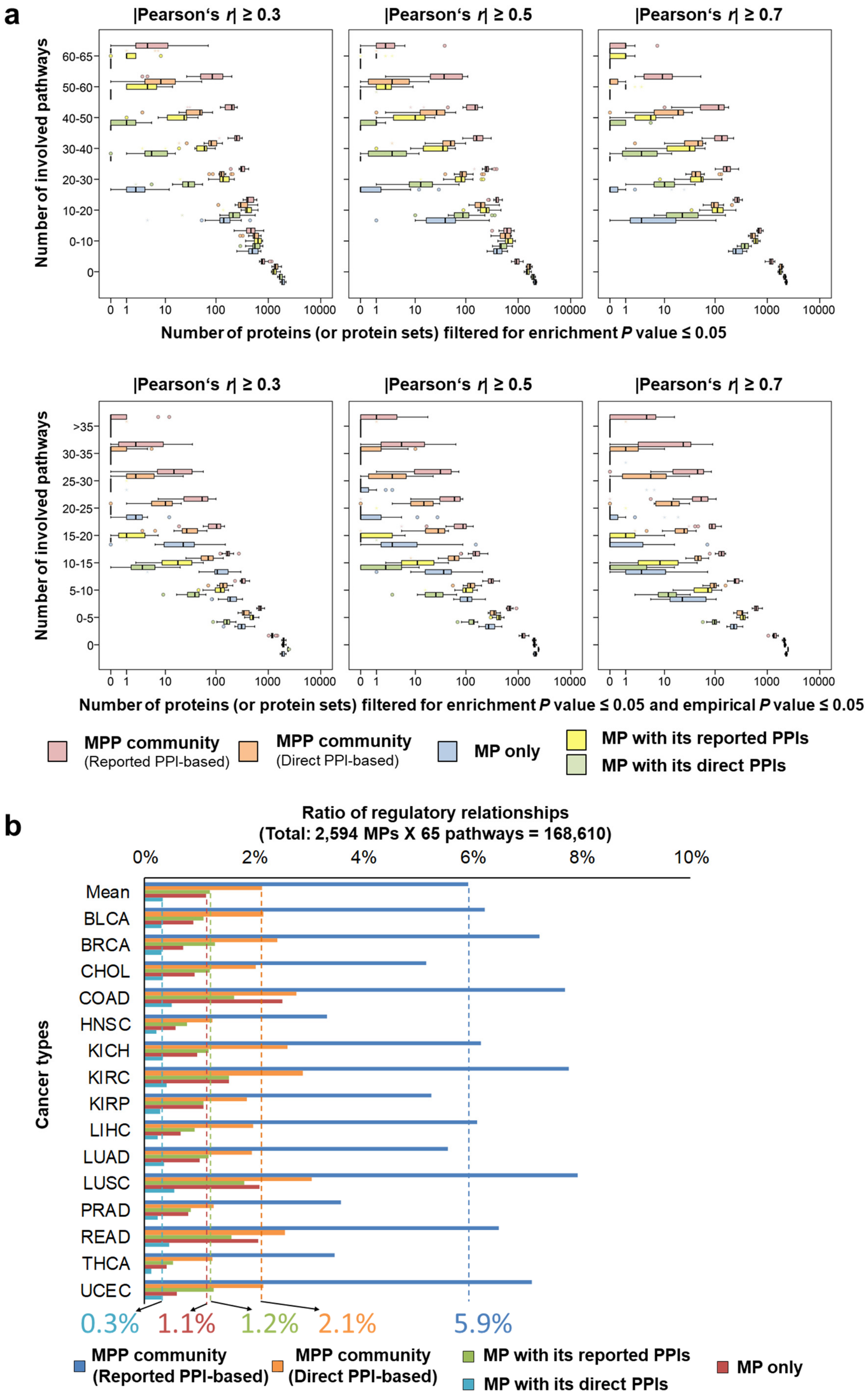

a
|Pearson's r| ≥ 0.3
|Pearson's r| ≥ 0.5
|Pearson's r| ≥ 0.7
Number of involved pathways
60-65
50-60
40-50
30-40
20-30
10-20
0-10
0
0 1 10 100 1000 10000
Number of proteins (or protein sets) filtered for enrichment P value ≤ 0.05
>35
30-35
25-30
20-25
15-20
10-15
5-10
0-5
Number of proteins (or protein sets) filtered for enrichment P value ≤ 0.05 and empirical P value ≤ 0.05
MPP community (Reported PPI-based)
MPP community (Direct PPI-based)
MP only
MP with its reported PPIs
MP with its direct PPIs
b
Ratio of regulatory relationships
(Total: 2,594 MPs X 65 pathways = 168,610)
0% 2% 4% 6% 8% 10%
Cancer types
Mean
BLCA
BRCA
CHOL
COAD
HNSC
KICH
KIRC
KIRP
LIHC
LUAD
LUSC
PRAD
READ
THCA
UCEC
0.3% 1.1% 1.2% 2.1% 5.9%
MPP community (Reported PPI-based)
MPP community (Direct PPI-based)
MP with its reported PPIs
MP with its direct PPIs
MP only

c

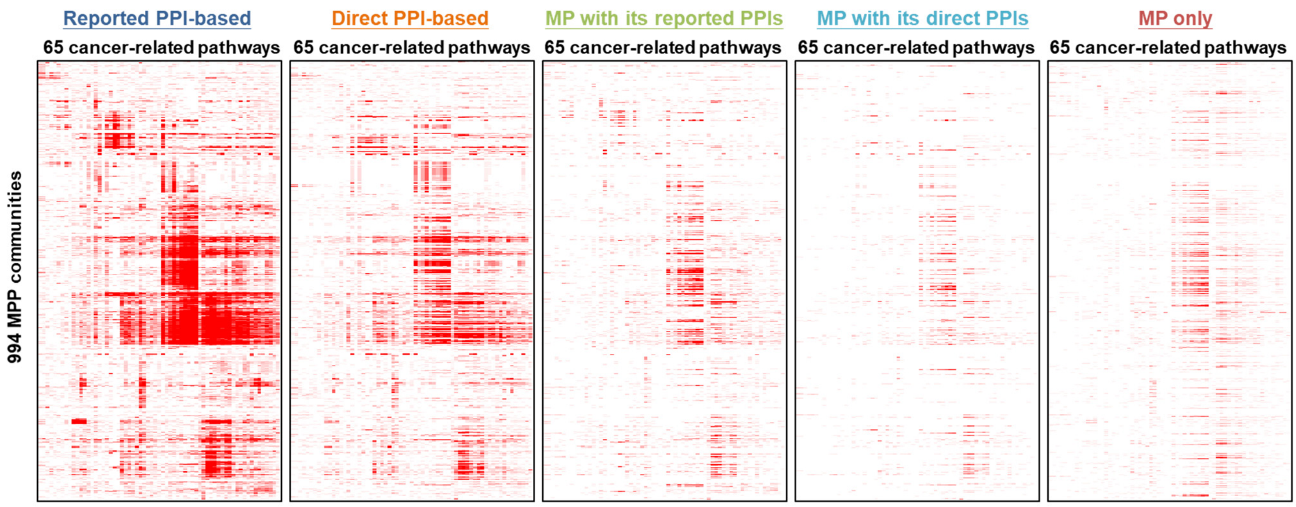

**Pearson's *r***	Reported PPI-based	Direct PPI-based	MP with its reported PPIs	MP with its direct PPIs	MP only
Reported PPI-based		0.632	0.520	0.304	0.325
Direct PPI-based	0.632		0.350	0.353	0.269
MP with its reported PPIs	0.520	0.350		0.536	0.338
MP with its direct PPIs	0.304	0.353	0.536		0.284
MP only	0.325	0.269	0.338	0.284	

Supplementary Figure 18. Comparison of pathways regulated by the MPs themselves, MPs with reported/direct PPIs, and MPs with predicted PPIs and reported/direct PPIs (i.e., reported PPI-/direct PPI-based MPP communities) in 15 cancer types.

(**a**) Distributions (boxplot) of the numbers of involved cancer-related pathways in 15 cancer types against the numbers of proteins (or protein sets) containing only MP itself, MP with reported/direct PPIs, and MP with predicted PPIs and reported/direct PPIs when co-expressed gene pair criteria are set to |Pearson's r| ≥ 0.3, ≥ 0.5, and ≥ 0.7. The involvements (enrichment P values ≤ 0.05) of cancer-related pathways for gene sets considering and not considering empirical P values ≤ 0.05 are shown in top and bottom, respectively. For example, the reported PPI-based MPP communities in BRCA achieved the highest annotation rate, as 49% (1,267/2,594) of the reported PPI-based MPP communities were annotated in at least one cancer-related pathway based on enrichment P values ≤ 0.05 and empirical P values ≤ 0.05 using co-expressed gene pairs with |Pearson's r| ≥ 0.5, while only 17% of MPs by themselves and 22% of MPs with interacting proteins derived from reported PPIs were annotated. The box represents the IQR and the line in the box is the median. The whiskers denote the lowest and highest values within 1.5 times IQR from the first and third quartiles, respectively. Circles

and asterisks represent the outliers and extreme outliers, respectively. (**b**) Ratios of the regulatory relationships between 2,594 MPs (or interacting protein sets of MPs) and 65 cancer-related pathways in distinct cancers. The dashed lines indicate the mean ratios across 15 cancers. We identified and compared the regulation between MPs and the pathways in 15 distinct cancers by considering only the MP itself, the MP with reported/direct PPIs, and the reported PPI-based (or direct PPI-based) MPP community, including the MP and its reported (or direct) PPIs and predicted PPIs. Among the 168,610 possible regulatory relationships between 2,594 MPs and 65 pathways in 15 cancers, the mean ratio (5.9%, 10,007 relationships) of regulatory relationships across 15 cancers for reported PPI-based MPP communities was greater than those for direct PPI-based MPP communities (2.1%, 3,622 relationships), MPs with reported PPIs (1.2%, 2,003 relationships), MPs with direct PPIs (0.3%, 543 relationships), and only MPs themselves (1.1%, 1,881 relationships). For example, CHRNA9 may play a role in the regulation of adherens junction via interacting with EGFR and ERBB2, which were identified by our reported-PPI based S_{SIM} approach (**Fig. 5b**). However, this regulatory relationship cannot be observed by considering only MPs with reported/direct PPIs and only MPs themselves. (**c**) Heat maps of meta-*z*-scores for five types of MPP community- (or MP-) regulated pathways between 994 overlapping MPP communities (or MPs) and 65 cancer-related pathways in 15 cancers. We constructed and compared the maps of CaMPNets for these five types of MPP community- and MP-regulated pathways. The result indicates that the heat maps of meta-*z*-scores between reported PPI-based and direct PPI-based communities displayed a higher positive correlation (R = 0.632) than any other two heat maps ($R \leq 0.536$).

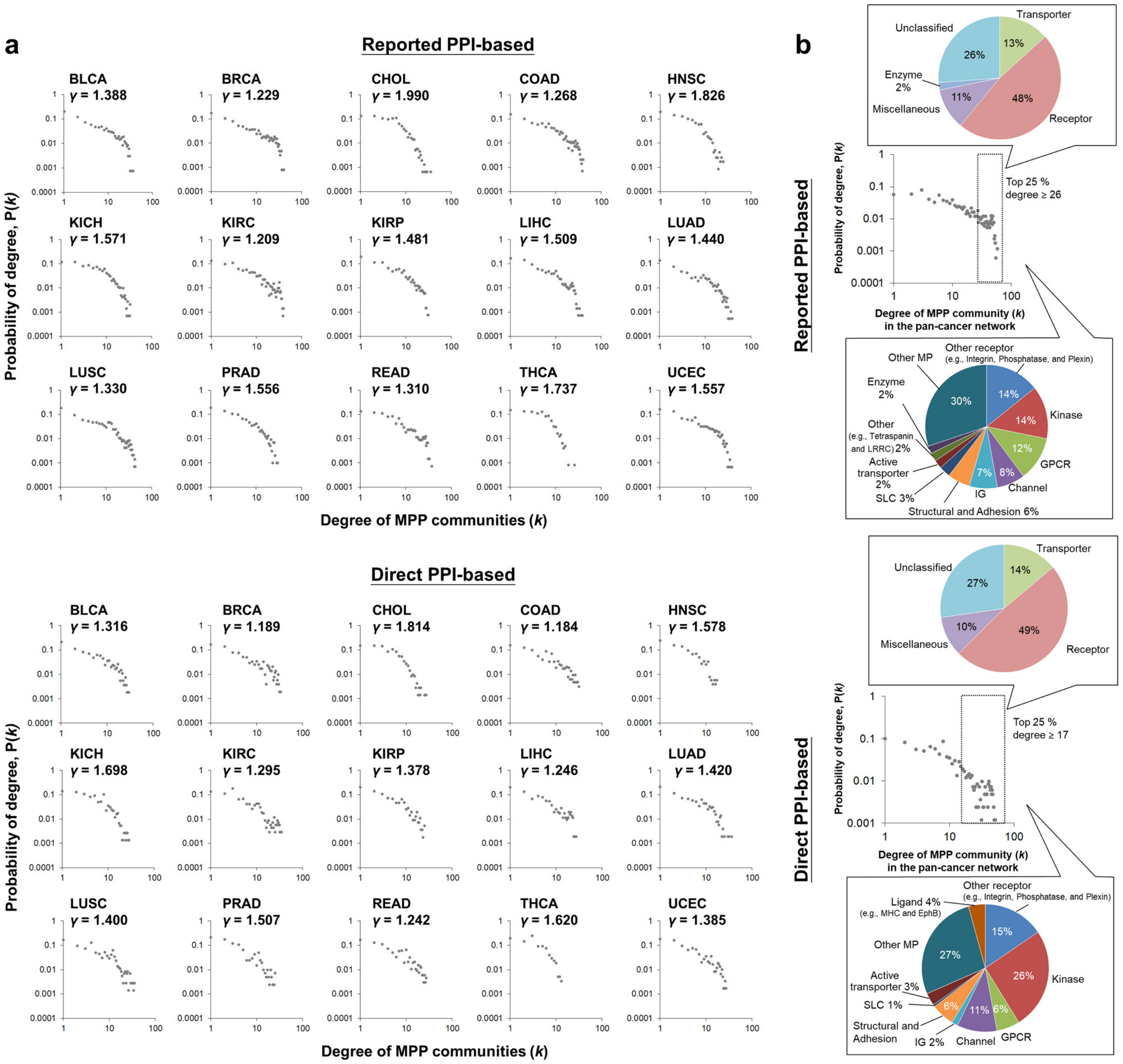

Supplementary Figure 19. Characteristics of CaMPNet-based networks.

(**a**) Degree distributions of the networks constructed by 1,862 reported PPI-based CaMPNets (top) and 1,009 direct PPI-based CaMPNets (bottom) in different cancer types illustrating scale-free topology. These networks were evaluated based on the scale-free network characteristic, described as P(k) ~ $k^{-\gamma}$, where γ is the degree exponent in which the probability of a node (MPP community) with k links (number of community-regulated pathways) decreases as the node degree increases along a log–log plot. The degree exponent γ values ranged between 1.184 and 1.990 in these networks. (**b**) Pie charts of 405 reported-PPI-based MPP communities (top) and 187 direct-PPI-based MPP communities (bottom), which were considered hubs in the network if their degree was ≥ 26 and ≥ 17 (degrees within

the top 25% of all communities), respectively, in the five groups (top subpanel) and in the top 10 families (bottom subpanel) containing the most members.

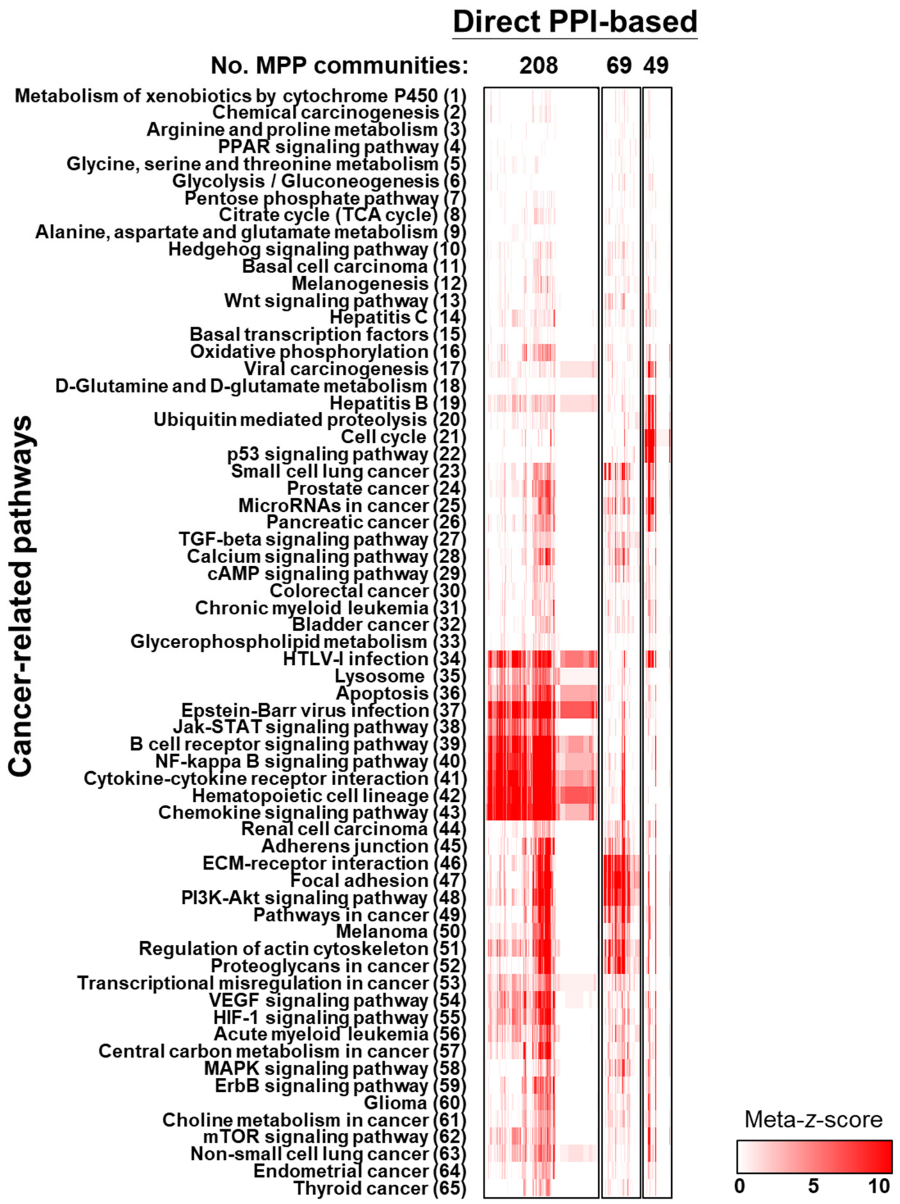

Supplementary Figure 20. Heat map and hierarchical clustering of meta-*z*-scores for community-regulated pathways between direct PPI-based MPP communities and 65 cancer-related pathways in 15 cancers.

The top corresponding clusters, ranked by cluster size, for direct PPI-based community-regulated pathways are shown, and details of the clusters are provided in **Supplementary Data 4**.

a

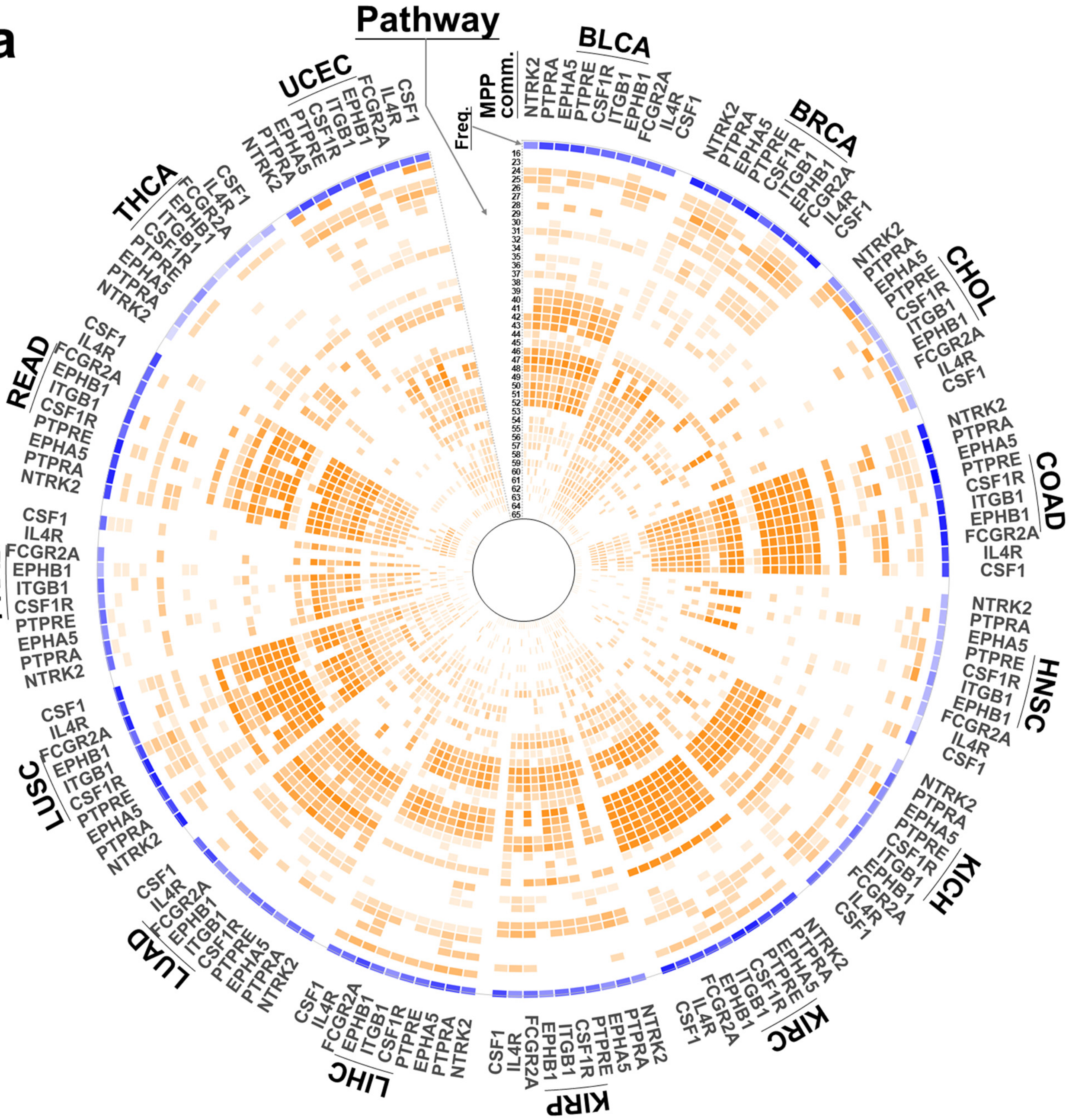
Pathway
Freq.
MPP comm.
BLCA
BRCA
CHOL
COAD
HNSC
KICH
KIRC
KIRP
LIHC
LUAD
LUSC
PRAD
READ
THCA
UCEC
NTRK2
PTPRA
EPHA5
PTPRE
CSF1R
ITGB1
EPHB1
FCGR2A
IL4R
CSF1

b

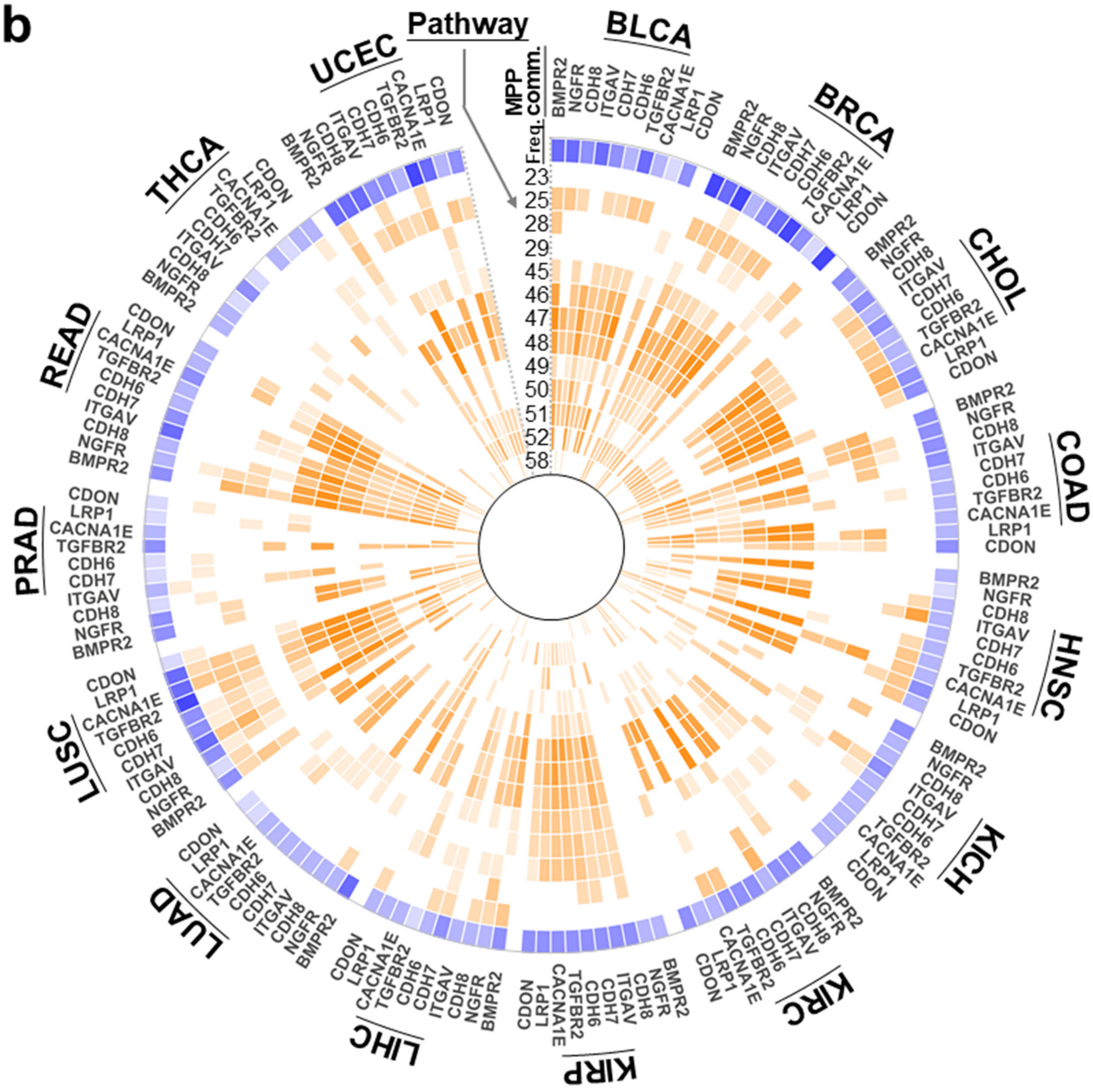

Pathway
MPP comm.
Freq.
23
25
28
29
45
46
47
48
49
50
51
52
58
BLCA
BRCA
CHOL
COAD
HNSC
KICH
KIRC
KIRP
LIHC
LUAD
LUSC
PRAD
READ
THCA
UCEC
BMPR2
NGFR
CDH8
ITGAV
CDH7
CDH6
TGFBR2
CACNA1E
LRP1
CDON

c

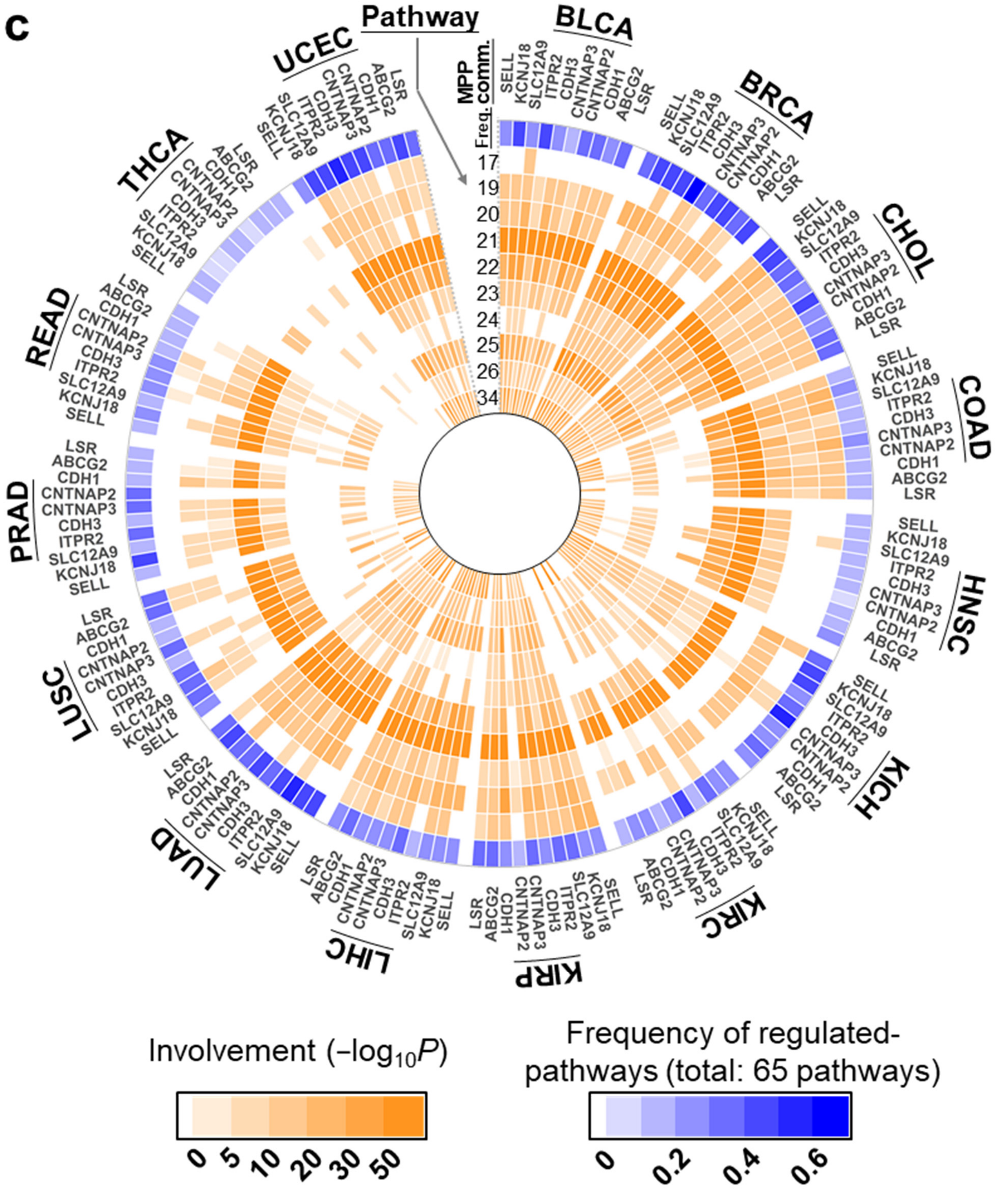

Pathway
MPP
Freq. comm.
17
19
20
21
22
23
24
25
26
34
BLCA
BRCA
CHOL
COAD
HNSC
KICH
KIRC
KIRP
LIHC
LUAD
LUSC
PRAD
READ
THCA
UCEC
SELL
KCNJ18
SLC12A9
ITPR2
CDH3
CNTNAP3
CNTNAP2
CDH1
ABCG2
LSR
Involvement (−log10P)
0 5 10 20 30 50
Frequency of regulated-pathways (total: 65 pathways)
0 0.2 0.4 0.6

Supplementary Figure 21. Polar histograms showing the involvement scores of the top 10 reported PPI-based MPP communities with high frequencies of regulated pathways in 15 human cancers for the (a) 2nd-, (b) 3rd-, and (c) 4th-ranked clusters according to group size.

The label outlines surrounding the polar histogram indicate the cancer type. The most external circle displays the frequencies of regulated pathways for MPP communities from high (blue) to zero (white). The regulated pathways for the (**a**) 2nd- (135 communities), (**b**) 3rd- (119 communities), and (**c**) 4th-ranked (105 communities) clusters are selected and visualized by frequencies of at least 0.50. The involvement scores (i.e., $-\log_{10}$ enrichment *P* value) of community-regulated pathways in the CaMPNets are shown in the internal circles from high (orange) to low (light orange) and zero (white). Of note, the number symbols refer to the pathways listed in **Figure 3c**. For example, the cell cycle pathway is No. 21.

a

Frequency of MPP communities (0 – 0.15 – ≥0.3)

	LUSC	COAD	KICH	KIRC	LUAD	READ	LIHC	KIRP	UCEC	BLCA	BRCA	THCA	CHOL	PRAD	HNSC
NF-kappa B signaling pathway (40)	0.18	0.18	0.22	0.19	0.20	0.14	0.20	0.18	0.19	0.13	0.05	0.13	0.09	0.07	0.03
Regulation of actin cytoskeleton (51)	0.20	0.24	0.09	0.19	0.10	0.22	0.04	0.14	0.15	0.20	0.15	0.04	0.16	0.11	0.10
Hematopoietic cell lineage (42)	0.19	0.18	0.26	0.21	0.19	0.14	0.19	0.16	0.06	0.17	0.02	0.16	0.07	0.05	0.05
B cell receptor signaling pathway (39)	0.19	0.18	0.25	0.19	0.11	0.15	0.12	0.16	0.17	0.14	0.11	0.04	0.10	0.04	0.06
Chemokine signaling pathway (43)	0.17	0.19	0.24	0.21	0.20	0.16	0.17	0.16	0.07	0.14	0.03	0.17	0.06	0.01	0.04
VEGF signaling pathway (54)	0.19	0.21	0.05	0.11	0.18	0.15	0.18	0.07	0.14	0.09	0.24	0.05	0.14	0.07	0.07
Cytokine-cytokine receptor interaction (41)	0.18	0.18	0.21	0.21	0.20	0.13	0.18	0.13	0.10	0.13	0.04	0.14	0.05	0.01	0.04
PI3K-Akt signaling pathway (48)	0.18	0.18	0.16	0.13	0.05	0.15	0.14	0.13	0.12	0.19	0.12	0.05	0.13	0.12	0.06
Calcium signaling pathway (28)	0.19	0.20	0.06	0.08	0.08	0.19	0.18	0.12	0.12	0.08	0.21	0.06	0.06	0.12	0.10
Apoptosis (36)	0.17	0.08	0.24	0.11	0.19	0.14	0.21	0.07	0.06	0.09	0.08	0.05	0.05	0.00	0.08
MAPK signaling pathway (58)	0.14	0.11	0.12	0.16	0.09	0.14	0.07	0.08	0.15	0.06	0.17	0.03	0.06	0.10	0.07
Jak-STAT signaling pathway (38)	0.16	0.16	0.13	0.19	0.18	0.07	0.04	0.06	0.09	0.06	0.06	0.05	0.04	0.04	0.04
Renal cell carcinoma (44)	0.13	0.09	0.06	0.03	0.10	0.06	0.05	0.10	0.09	0.06	0.13	0.10	0.05	0.09	0.02
Lysosome (35)	0.13	0.02	0.03	0.06	0.16	0.07	0.03	0.16	0.05	0.10	0.06	0.07	0.03	0.03	0.01
Ubiquitin mediated proteolysis (20)	0.07	0.07	0.05	0.03	0.06	0.06	0.09	0.04	0.13	0.05	0.05	0.04	0.08	0.03	0.05

b

	KIRC	COAD	LUSC	KICH	READ	LIHC	LUAD	UCEC	BLCA	KIRP	BRCA	CHOL	THCA	HNSC	PRAD
NF-kappa B signaling pathway (40)	0.19	0.18	0.18	0.22	0.14	0.20	0.20	0.19	0.13	0.18	0.05	0.09	0.13	0.03	0.07
Regulation of actin cytoskeleton (51)	0.19	0.24	0.20	0.09	0.22	0.04	0.10	0.15	0.20	0.14	0.15	0.16	0.04	0.10	0.11
B cell receptor signaling pathway (39)	0.19	0.18	0.19	0.25	0.15	0.12	0.11	0.17	0.14	0.16	0.11	0.10	0.04	0.06	0.04
Chemokine signaling pathway (43)	0.21	0.19	0.17	0.24	0.16	0.17	0.20	0.07	0.14	0.16	0.03	0.06	0.17	0.04	0.01
Epstein-Barr virus infection (37)	0.28	0.17	0.19	0.11	0.14	0.08	0.19	0.13	0.12	0.16	0.12	0.06	0.18	0.07	0.00
PI3K-Akt signaling pathway (48)	0.13	0.18	0.18	0.16	0.15	0.14	0.05	0.12	0.19	0.13	0.12	0.13	0.05	0.06	0.12
Calcium signaling pathway (28)	0.08	0.20	0.19	0.06	0.19	0.18	0.08	0.12	0.08	0.12	0.21	0.06	0.06	0.10	0.12
HTLV-I infection (34)	0.26	0.24	0.17	0.15	0.12	0.06	0.17	0.08	0.07	0.06	0.07	0.06	0.03	0.07	0.03
Apoptosis (36)	0.11	0.08	0.17	0.24	0.14	0.21	0.19	0.06	0.09	0.07	0.08	0.05	0.05	0.08	0.00
ECM-receptor interaction (46)	0.09	0.17	0.13	0.10	0.13	0.14	0.03	0.10	0.18	0.13	0.09	0.14	0.04	0.06	0.07
MAPK signaling pathway (58)	0.16	0.11	0.14	0.12	0.14	0.07	0.09	0.15	0.06	0.08	0.17	0.06	0.03	0.07	0.10
Jak-STAT signaling pathway (38)	0.19	0.16	0.16	0.13	0.07	0.04	0.18	0.09	0.06	0.06	0.06	0.04	0.05	0.04	0.04
Hepatitis B (19)	0.04	0.14	0.15	0.09	0.05	0.19	0.06	0.06	0.06	0.04	0.09	0.05	0.02	0.06	0.03
Wnt signaling pathway (13)	0.09	0.12	0.05	0.02	0.07	0.10	0.05	0.08	0.09	0.06	0.05	0.12	0.02	0.02	0.08
TGF-beta signaling pathway (27)	0.12	0.04	0.09	0.05	0.08	0.13	0.03	0.11	0.11	0.03	0.06	0.08	0.05	0.04	0.01
Lysosome (35)	0.06	0.02	0.13	0.03	0.07	0.03	0.16	0.05	0.10	0.16	0.06	0.03	0.07	0.01	0.03
Hepatitis C (14)	0.19	0.02	0.06	0.08	0.01	0.13	0.02	0.07	0.06	0.03	0.08	0.08	0.05	0.06	0.02
Ubiquitin mediated proteolysis (20)	0.03	0.07	0.07	0.05	0.06	0.09	0.06	0.13	0.05	0.04	0.05	0.08	0.04	0.05	0.03
Viral carcinogenesis (17)	0.16	0.10	0.05	0.06	0.06	0.04	0.04	0.04	0.02	0.12	0.03	0.08	0.03	0.03	0.02
p53 signaling pathway (22)	0.03	0.10	0.08	0.05	0.07	0.04	0.06	0.06	0.05	0.03	0.06	0.05	0.01	0.06	0.03
Cell cycle (21)	0.02	0.09	0.06	0.05	0.06	0.04	0.06	0.06	0.05	0.03	0.05	0.05	0.02	0.05	0.02
PPAR signaling pathway (4)	0.03	0.02	0.02	0.01	0.03	0.07	0.01	0.01	0.02	0.06	0.04	0.03	0.01	0.01	0.01
Basal transcription factors (15)	0.03	0.03	0.00	0.12	0.01	0.00	0.00	0.00	0.00	0.01	0.04	0.06	0.00	0.00	0.00

Supplementary Figure 22. Frequency of reported PPI-based MPP communities involved in cancer-related pathways relevant to (a) immune response and (b) viral infection.

The frequency of MPP communities for a pathway is given by the number of involved communities divided by 1,862 communities. We derived 15 immune-related pathways and 23 viral infection-related pathways according to their connections with pathways (red) in the ‘Immune system’ and ‘Infectious diseases: Viral’ categories. The orders of the cancers and pathways are sorted by the sum of the community frequencies of all the selected pathways and cancer types, respectively.

a

TCGA abbreviation	Tissue	Secondary cancers after the target cancer (among 15 selected cancer types)
BLCA	Bladder	BLCA[a,b,c], LUAD[b,c], LUSC[b,c], KIRP[b,c], KIRC[b], KICH, HNSC[b,c], PRAD[b,c]
BRCA	Breast	BRCA[a,b,c], UCEC[b,c], COAD, THCA
COAD	Colon	COAD[a,b,c], READ[b,c], KIRC[b,c], KIRP[b,c], KICH[b,c], UCEC[b,c], CHOL
READ	Rectal	READ[a,b,c], COAD[b,c], LUSC[b,c], LUAD[b,c], KIRC[b,c], KIRP[b,c], KICH[b,c]
KICH, KIRP, KIRC	Kidney	KIRP[a,b,c], KIRC[a,b,c], KICH[a,b,c], BLCA[b,c], PRAD, THCA
LUAD, LUSC	Lung	LUAD[a,b,c], LUSC[a,b,c], COAD[b,c], READ[b,c], BLCA[b,c], KIRC[b,c], KIRP[b,c], KICH[b,c], THCA, HNSC[c]
PRAD	Prostate	BLCA[b,c], THCA
THCA	Thyroid	KIRP, KIRC[b], KICH, BRCA, PRAD
UCEC	Uterus	BRCA[b,c], BLCA[b,c], COAD[b,c], READ
HNSC	Head and Neck	HNSC[a,b,c], CHOL[b,c], LUAD[,c], LUSC, COAD, READ, THCA

[a] This is different from the first cancer coming back.
[b] The Pearson's r between all PPI-based CaMPNet profiles of two cancers is higher than 0.4.
[c] The Pearson's r between direct PPI-based CaMPNet profiles of two cancers is higher than 0.4.

b

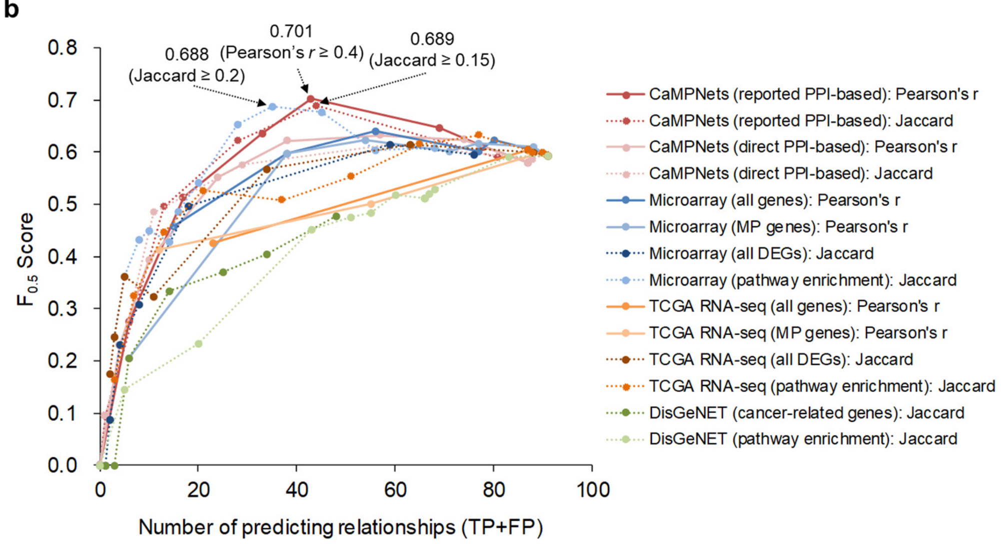

c

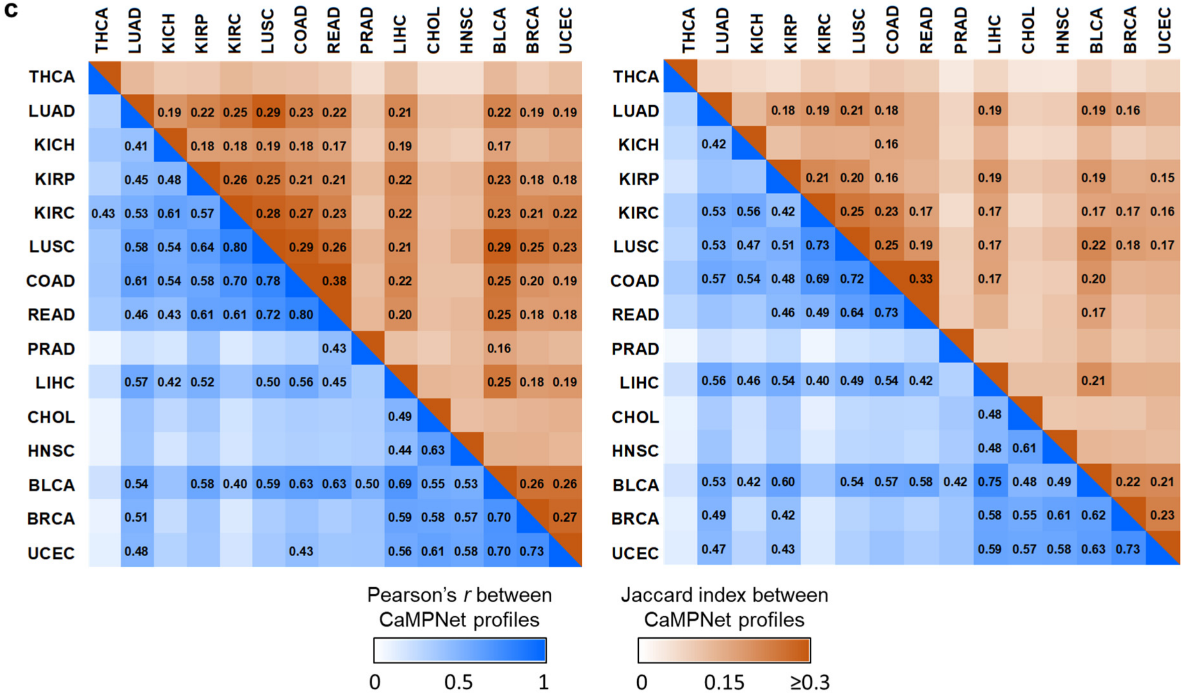

d

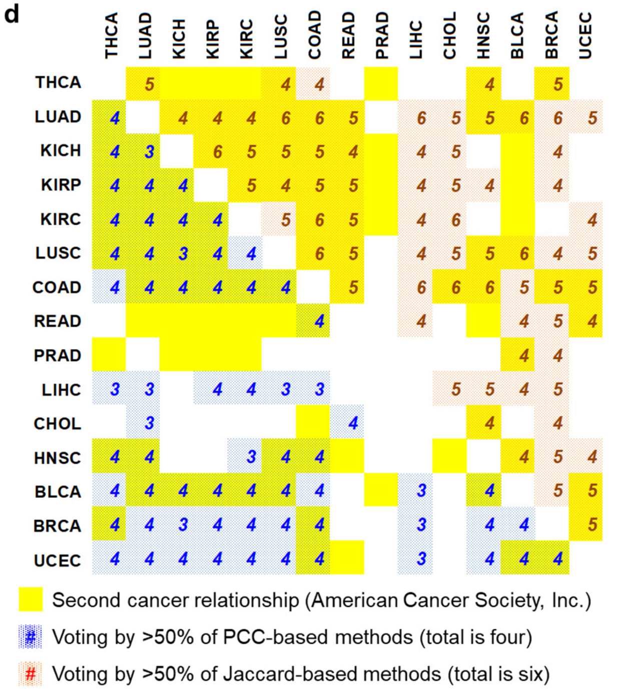

Supplementary Figure 23. Second cancer relationships of the 15 cancer types using CaMPNet profiles, gene expression data sets of microarrays assembled from the GEO database, gene expression data sets assembled from TCGA RNAseq data sets, and cancer-related gene sets in the DisGeNet database.

(**a**) Second cancer list provided by the American Cancer Society, Inc[21]. In comparison to recurrence, which is when the cancer comes back after treatment, a second cancer is defined as when a cancer survivor subsequently develops a new and unrelated cancer. (**b**) Distribution between the $F_{0.5}$ scores and numbers of predicted relationships for 15 cancer types suggested by 14 approaches using the correlation value or Jaccard index at different thresholds. Here, the $F_{0.5}$ score, for which precision is weighted higher than recall, is used to avoid the selection of approaches with certain thresholds, suggesting almost all possible relationships. (**c**) Heat map of the correlations (blue; lower triangle) and Jaccard indexes (orange; upper triangle) of reported PPI- (left) and direct PPI-based (right) CaMPNet profiles for 15 cancer types (**Supplementary Data 3** and **4**). The Jaccard similarity coefficient $\mathrm{J}(A, B)$ is given as $\mathrm{J}(A, B) = |A \cap B| / |A \cup B|$, where $A \cap B$ is the number of common community-regulated pathways (intersection set) in cancers A and B, and $A \cup B$ is the number of union pathways in cancers A and B. The values indicate pairs of cancers with a Pearson's $r \geq 0.4$ or a Jaccard index ≥ 0.15 as determined by the highest $F_{0.5}$ score using the second cancer list[21]. Notably, two cancers with highly similar CaMPNet profiles were often observed to be second cancers for each other. (**d**) Relationships of the 15 cancer types suggested by the votes of six and four different approaches using the correlation value (blue grids; lower triangle) and the Jaccard index (orange grids; lower triangle), respectively. The values indicate the number of approaches that suggested the pairs of cancers. The yellow boxes show the second cancer relationships recorded in the second cancer list[21]. PCC: Pearson correlation coefficient.

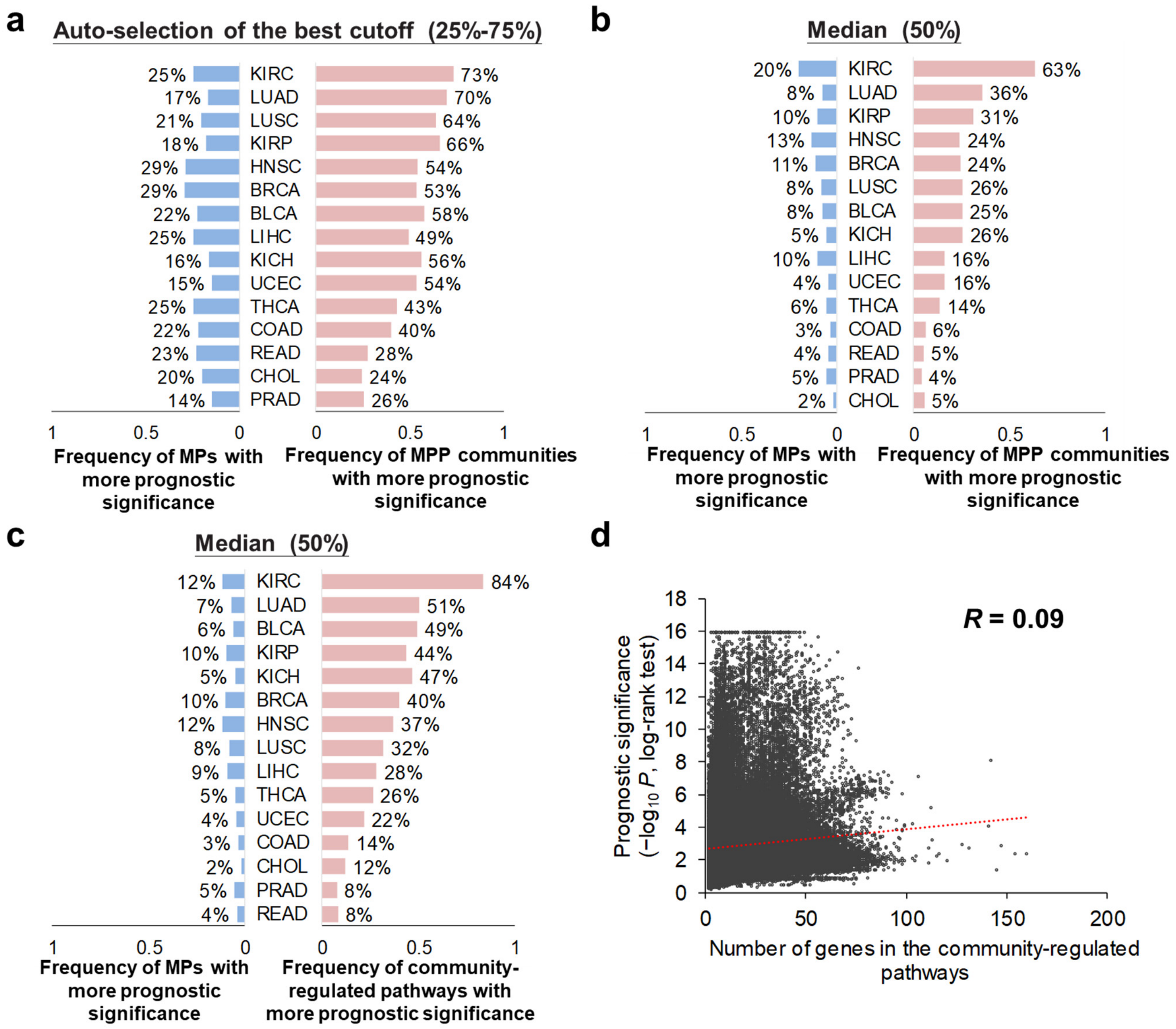

Supplementary Figure 24. Prognostic significance among MPs by themselves, reported PPI-based MPP communities, and reported PPI-based MPP community-regulated pathways across human cancers.

The frequencies of MPP communities with more significant prognostic associations in 10-year overall survival are higher than those of MPs by themselves in 15 cancers regardless of whether the patients were stratified by the (**a**) auto-select best cutoff (25-75%) or (**b**) median (50%) for high or low combined scores. Similarly, members of community-regulated pathways showed a more significant prognostic association with 10-year overall survival for patients split by the (**c**) median or auto-select best cutoff (**Fig. 4a**) scores than MPs by themselves regardless of the cancer type. Cancers are ranked

by the sum of frequencies for significant prognostic outcomes ($P < 0.05$, log-rank test). (**d**) Scatter plot of gene numbers in community-regulated pathways versus the prognostic significance ($-\log_{10} P$) of 10-year overall survival assessed by a log-rank test. The distributions of gene numbers show no correlation ($R = 0.09$) with their prognostic significance.

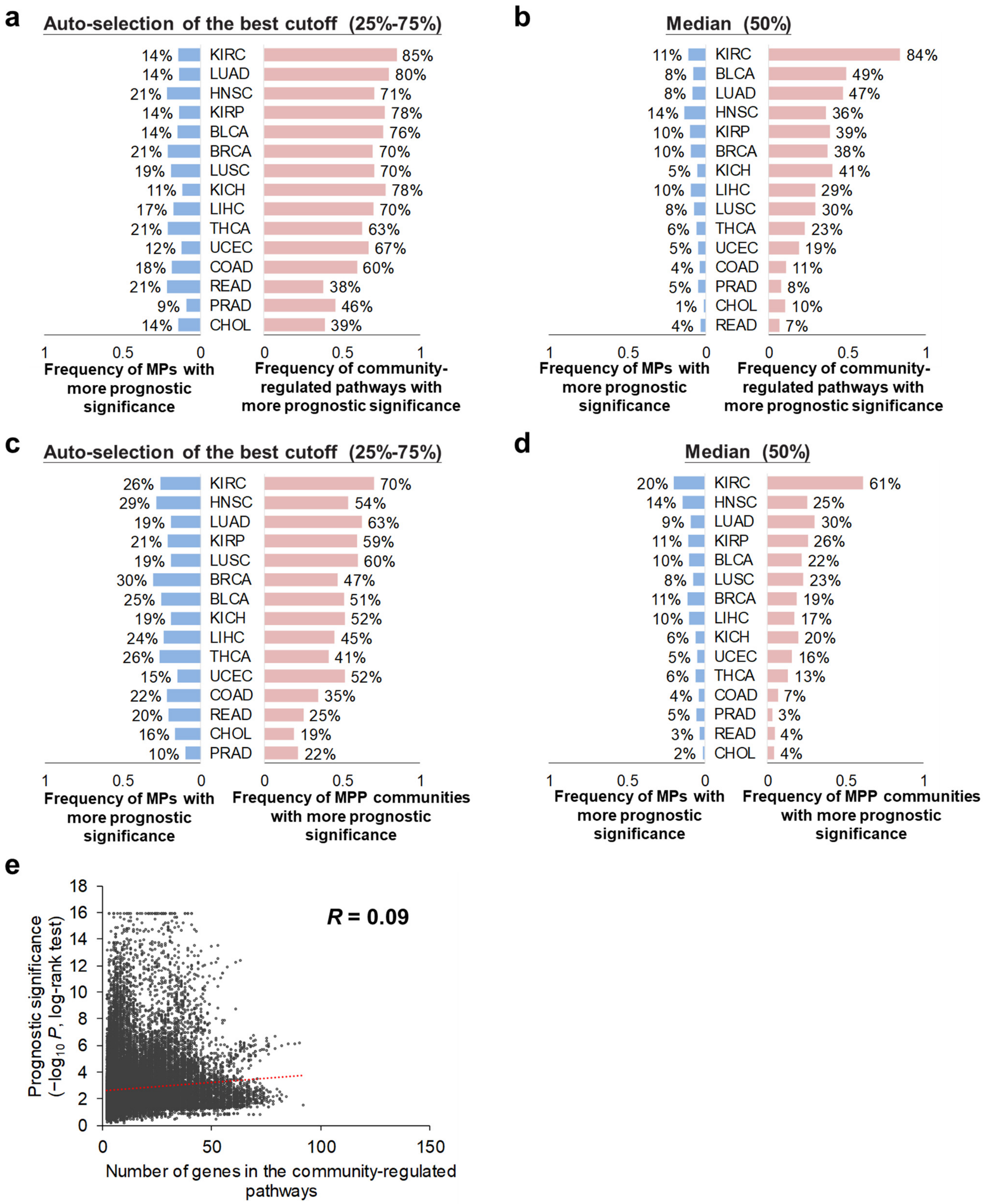
a
Auto-selection of the best cutoff (25%-75%)
14% KIRC 85%
14% LUAD 80%
21% HNSC 71%
14% KIRP 78%
14% BLCA 76%
21% BRCA 70%
19% LUSC 70%
11% KICH 78%
17% LIHC 70%
21% THCA 63%
12% UCEC 67%
18% COAD 60%
21% READ 38%
9% PRAD 46%
14% CHOL 39%
1 0.5 0
0 0.5 1
Frequency of MPs with more prognostic significance
Frequency of community-regulated pathways with more prognostic significance
b
Median (50%)
11% KIRC 84%
8% BLCA 49%
8% LUAD 47%
14% HNSC 36%
10% KIRP 39%
10% BRCA 38%
5% KICH 41%
10% LIHC 29%
8% LUSC 30%
6% THCA 23%
5% UCEC 19%
4% COAD 11%
5% PRAD 8%
1% CHOL 10%
4% READ 7%
1 0.5 0
0 0.5 1
Frequency of MPs with more prognostic significance
Frequency of community-regulated pathways with more prognostic significance
c
Auto-selection of the best cutoff (25%-75%)
26% KIRC 70%
29% HNSC 54%
19% LUAD 63%
21% KIRP 59%
19% LUSC 60%
30% BRCA 47%
25% BLCA 51%
19% KICH 52%
24% LIHC 45%
26% THCA 41%
15% UCEC 52%
22% COAD 35%
20% READ 25%
16% CHOL 19%
10% PRAD 22%
1 0.5 0
0 0.5 1
Frequency of MPs with more prognostic significance
Frequency of MPP communities with more prognostic significance
d
Median (50%)
20% KIRC 61%
14% HNSC 25%
9% LUAD 30%
11% KIRP 26%
10% BLCA 22%
8% LUSC 23%
11% BRCA 19%
10% LIHC 17%
6% KICH 20%
5% UCEC 16%
6% THCA 13%
4% COAD 7%
5% PRAD 3%
3% READ 4%
2% CHOL 4%
1 0.5 0
0 0.5 1
Frequency of MPs with more prognostic significance
Frequency of MPP communities with more prognostic significance
e
R = 0.09
Prognostic significance (−log10 P, log-rank test)
0 2 4 6 8 10 12 14 16 18
0 50 100 150
Number of genes in the community-regulated pathways

Supplementary Figure 25. Prognostic significance among MPs by themselves, direct PPI-based MPP communities, and direct PPI-based MPP community-regulated pathways across human cancers.

The frequencies of direct PPI-based MPP communities with more significant prognostic associations in 10-year overall survival are higher than those of MPs by themselves in 15 cancers regardless of whether the patients were stratified by the (**a**) auto-select best cutoff (25-75%) or (**b**) median (50%) for high or low combined scores. Similarly, members of direct PPI-based community-regulated pathways showed a more significant prognostic association with 10-year overall survival for patients split by the (**c**) auto-select best cutoff or (**d**) median scores than MPs by themselves regardless of the cancer type. Cancers are ranked by the sum of frequencies for significant prognostic outcomes ($P < 0.05$, log-rank test). (**e**) Scatter plot of gene numbers in direct PPI-based community-regulated pathways versus the prognostic significance ($-\log_{10} P$) of 10-year overall survival assessed by a log-rank test. The distributions of gene numbers show no correlation ($R = 0.09$) with their prognostic significance.

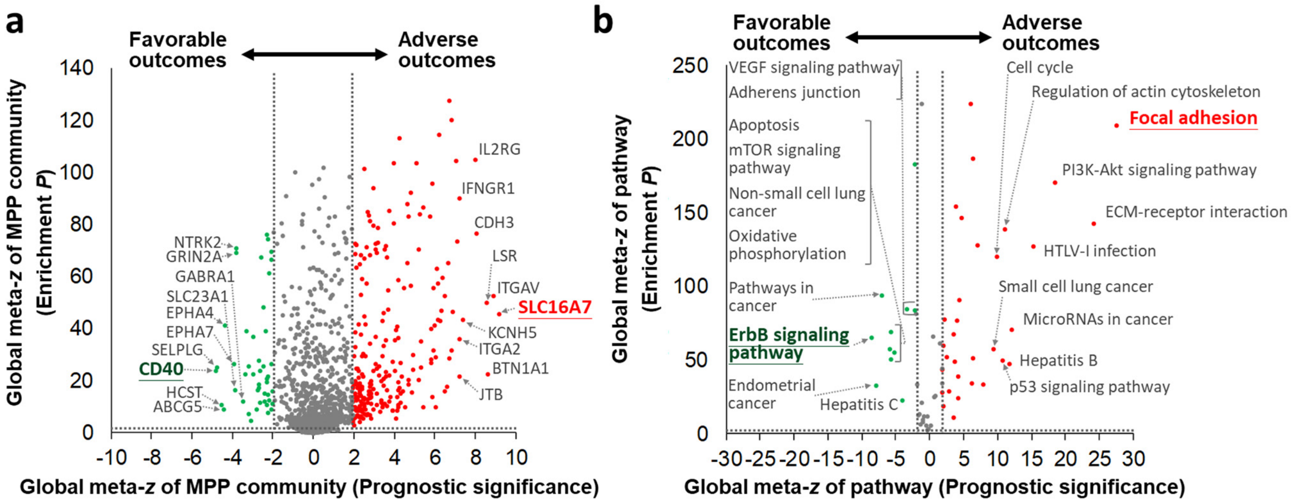

Supplementary Figure 26. Volcano plots presenting the global meta-*z*-scores of adverse (red) and favorable (green) prognostic associations versus global meta-*z*-score of involvement for (a) 1,862 MPP communities and (b) 65 cancer-related pathways across 15 cancers.

Gray dots represent non-enriched MPs or pathways (global meta-z < 1.64, or nominal one-sided P > 0.05) and insignificant prognostic MPs or pathways (|global meta-z| < 1.96, or nominal two-sided P > 0.05). The ten highest ranking adverse and favorable MPs (or pathways) were labeled and separately ranked by the absolute value of global meta-z-scores for prognostic significance.

a

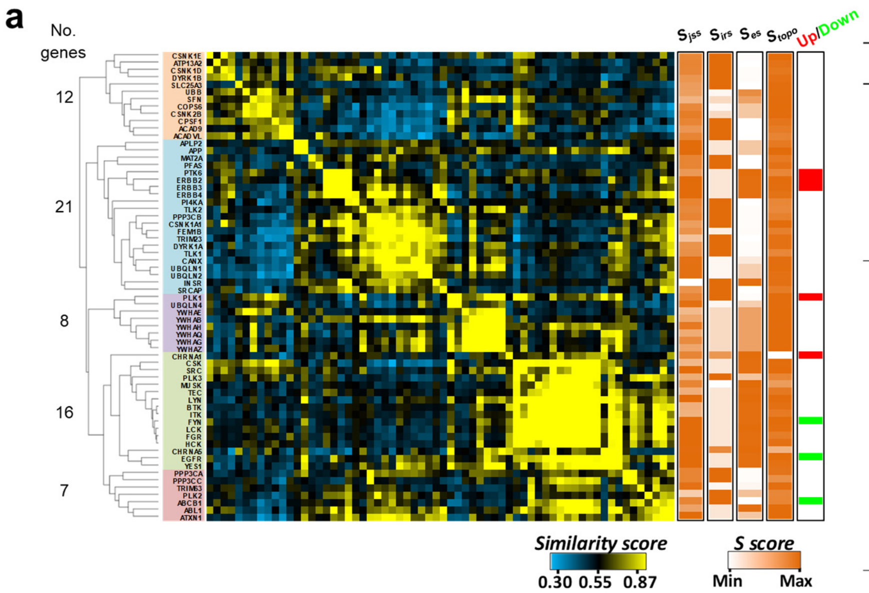

b

Sub-group	GN	S_{SIM}
1	CSNK1E	3.26
	ATP13A2	3.15
	CSNK1D	3.19
	DYRK1B	3.31
	SLC25A3	3.25
	UBB	3.10
	SFN	3.25
	COPS6	3.00
	CSNK2B	3.21
	CPSF1	3.24
	ACAD9	3.18
	ACADVL	3.26
2	APLP2	3.50
	APP	3.61
	MAT2A	3.13
	PFAS	3.17
	PTK6	3.15
	ERBB2	3.66
	ERBB3	3.65
	ERBB4	3.62
	PI4KA	3.13
	TLK2	3.23
	PPP3CB	3.12
	CSNK1A1	3.07
	FEM1B	3.04
	TRIM23	3.23
	DYRK1A	3.33
	TLK1	3.19
	CANX	3.02
	UBQLN1	3.42
	UBQLN2	3.40
	INSR	3.00
	SRCAP	3.35
3	PLK1	3.60
	UBQLN4	3.42
	YWHAE	3.22
	YWHAB	3.35
	YWHAH	3.67
	YWHAQ	3.32
	YWHAG	3.55
	YWHAZ	3.35
4	CHRNA1	3.00
	CSK	3.08
	SRC	3.65
	PLK3	3.36
	MUSK	3.27
	TEC	3.08
	LYN	3.65
	BTK	3.14
	ITK	3.05
	FYN	3.66
	LCK	3.64
	FGR	3.53
	HCK	3.62
	CHRNA5	3.20
	EGFR	3.68
	YES1	3.61
5	PPP3CA	3.20
	PPP3CC	3.00
	TRIM63	3.10
	PLK2	3.18
	ABCB1	3.11
	ABL1	3.24
	ATXN1	3.75

c

18 representative candidates interacting with CHRNA9 (Gene name)	Number of references (reporting interactions of nAChRs)	References
ABCB1	0	
APP	1	Wang *et al.*[40]
ATXN1	0	
COPS6	0	
CSNK1D	0	
EGFR	1	Jaldety *et al.*[41]
ERBB2	0	
ERBB3	0	
ERBB4	0	
FYN	3	Allen *et al.*[42]<br>Swope *et al.*[43]<br>Kumar *et al.*[44]
HCK	0	
INSR	0	
PLK1	0	
SFN	0	
SRC	3	Kumar *et al.*[44]<br>Charpantier *et al.*[45]<br>Wang *et al.*[46]
YWHAB	0	
YWHAG	0	
YWHAH	0	

d

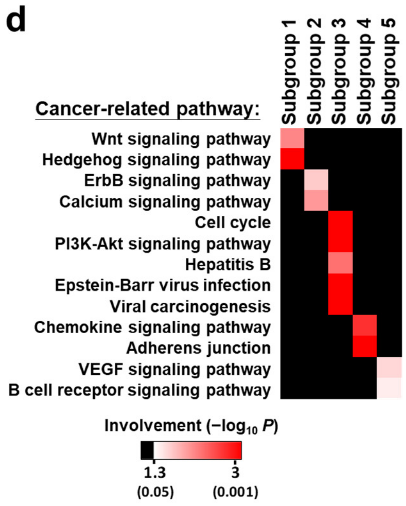

Supplementary Figure 27. Analysis and validation of CHRNA9 interacting protein candidates.

(**a**) Heat map of similarities among CHRNA9 interacting proteins. The similarity scores, ranging from 0 (blue; dissimilar) to 1 (yellow; similar), were evaluated by co-expression analyses of expression profiles in Breast Invasive Carcinoma (BRCA) tumor samples, sequence similarities, and *RSS* scores of GO terms for any two proteins potentially interacting with CHRNA9 (details in **Supplementary Note 10**). Here, the predicted results of the CHRNA9-UBC template are not shown because the number of polyubiquitin-C (UBC) interacting proteins (9,783) was far greater than that of other proteins in the reported PPI data. To select representative interacting proteins, we first used two-way hierarchical clustering to cluster 64 CHRNA9 interacting proteins with $S_{SIM} \geq 3.0$ into five subgroups. We then

selected at least one candidate in each subgroup (total of 18 candidates; e.g., ERBB2, FYN, and COPS6) to further verify by immunoprecipitation (IP) based on the several criteria (details in **Supplementary Note 10**). The color schemes on the right show the four scores (orange: high; white: low). (**b**) All 64 candidates in the five subgroups with $S_{SIM} \geq 3.0$ (medium confidence), containing 14 candidates with $S_{SIM} \geq 3.6$ (high confidence, red). (**c**) Interactions of nAChRs reported by previous works. Among 18 representative candidate partners of CHRNA9, four have been proposed to bind to nAChRs. For example, the human 42-amino acid β-amyloid peptide ($A\beta_{1-42}$) derived from amyloid precursor protein (APP) maintains the tight association characteristic with CHRNA7 in PC12 cells[68]. A previous study demonstrated that neuronal acetylcholine receptor subunit α7 (CHRNA7) may interact with epidermal growth factor receptor (EGFR) to promote EGFR localization to the sperm head and activate EGFR to promote fertilization[69]. Tyrosine-protein kinase Fyn (FYN) was suggested to be associated with nAChRs[70,71]; however, Kumar *et al.* indicated that FYN interacts with neuronal acetylcholine receptor subunit β4 (CHRNB4) but not CHRNA7 or CHRNA9 in the human sperm[72]. Proto-oncogene tyrosine-protein kinase Src (SRC) was known to directly bind to CHRNA7[72,73] and was associated with neuronal α3β4α5 acetylcholine receptors[74]. Although there was no study indicating that 18 selected candidates interact with CHRNA9, the findings for four binding proteins of nAChRs provide their predictable associations with CHRNA9. (**d**) Functional enrichment of the KEGG cancer-related pathways (adjusted P value ≤ 0.05) for each subgroup using a Bonferroni-corrected hypergeometric test. In comparison with CHRNA9, which was annotated in only the neuroactive ligand-receptor interaction pathway according to KEGG database[75], these interacting protein candidates in the five subgroups could be distinctively connected to 13 pathways. For example, ERBB2 and ERBB3 of subgroup 2 were associated with the ErbB and calcium signaling pathways.

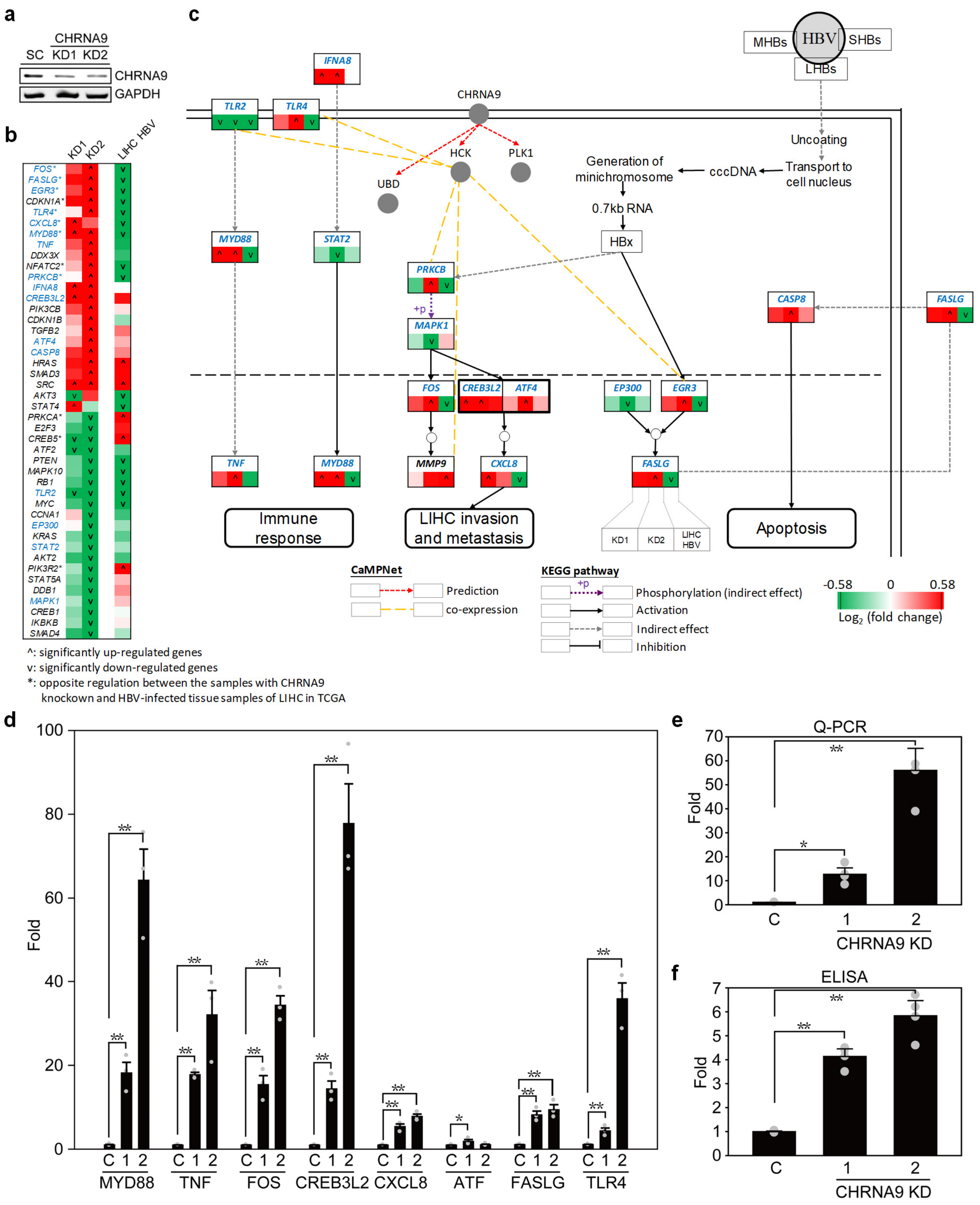

a
CHRNA9
SC KD1 KD2
CHRNA9
GAPDH
b
KD1 KD2 LIHC HBV
^: significantly up-regulated genes
v: significantly down-regulated genes
*: opposite regulation between the samples with CHRNA9 knockown and HBV-infected tissue samples of LIHC in TCGA
c
HBV
MHBs
SHBs
LHBs
Uncoating
Transport to cell nucleus
cccDNA
Generation of minichromosome
0.7kb RNA
HBx
CHRNA9
HCK
PLK1
UBD
Immune response
LIHC invasion and metastasis
Apoptosis
CaMPNet
Prediction
co-expression
KEGG pathway
Phosphorylation (indirect effect)
Activation
Indirect effect
Inhibition
Log2 (fold change)
d
Fold
MYD88 TNF FOS CREB3L2 CXCL8 ATF FASLG TLR4
e
Q-PCR
Fold
CHRNA9 KD
f
ELISA
Fold
CHRNA9 KD

Supplementary Figure 28. Effects of CHRNA9 knockdown on the hepatitis B pathway of liver hepatocellular carcinoma (LIHC).

(**a**) Hep3B cells were transfected with scramble (SC), CHRNA9 knockdown 1 (KD1) and CHRNA9 KD2 CRISPR/Cas9 virus for 48 hours and selected with puromycin for another 48 hours. The cells were immunoblotted with a CHRNA9 antibody to determine the protein knockdown efficiency. GAPDH served as the internal control. (**b**) Differential expression profiles of the hepatitis B pathway, which contained 44 genes with significant up-/downregulation in at least one test condition for Hep3B cell lines with CHRNA9 KD1 or KD2. For each gene, the fold change values, displayed in the color scheme (red: upregulated; green: downregulated; white: no change), are listed in order as (from left to right) the control (no treatment) versus CHRNA9 KD1 and CHRNA9 KD2. The fold changes of these genes in TCGA LIHC RNA-seq data derived from HBV-infected tissue samples are shown in the rightmost column. (**c**) Proposed model for the regulation of immune response, apoptosis, LIHC invasion and metastasis in the hepatitis B pathway via the knockdown of CHRNA9. Note that this proposed model displays only a part of the CHRNA9 community-regulated hepatitis B pathway in LIHC. HBV: hepatitis B virus; HBx: hepatitis B virus X protein; LHBs: large hepatitis B surface protein; MHBs: middle hepatitis B surface protein; SHBs: small hepatitis B surface protein. The cells were collected to measure the gene expression of (**d**) selected upregulated genes in the hepatitis B pathway and (**e**) the gene expression of HBsAg via Q-PCR analysis. (**f**) The supernatants of starvation medium in Hep3B cell lines with CHRNA9 KD1 and KD2 were collected and measured the amount of HBsAg by enzyme-linked immunosorbent assay (ELISA). The OD values of HBsAg were normalized with cell numbers. The results are expressed as the fold of the starvation control. The error bars indicate the mean ± standard error. Data were analyzed with Student's t-tests; all *P* values were two-sided. *P* values less than 0.05 and 0.01 are indicated by an asterisk and double asterisk, respectively. C: control; 1: CHRNA9 KD1; 2: CHRNA9 KD2.

a

BRCA

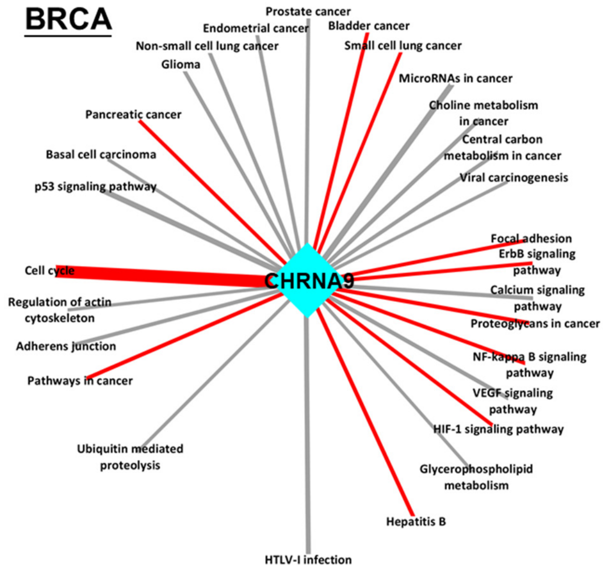

	Enriched	Non-enriched
Number of pathways in CaMPNets	11	17
Number of pathways not in CaMPNets	55	209

P value = 3×10^{-2}

LUAD

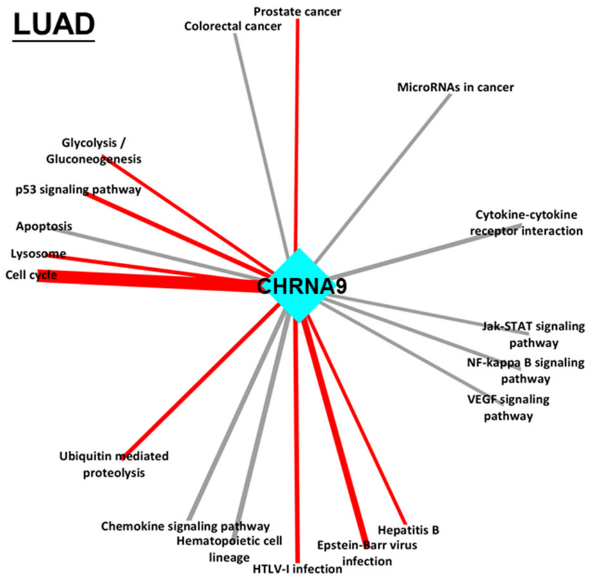

	Enriched	Non-enriched
Number of pathways in CaMPNets	9	9
Number of pathways not in CaMPNets	61	213

P value = 2×10^{-2}

b

BRCA

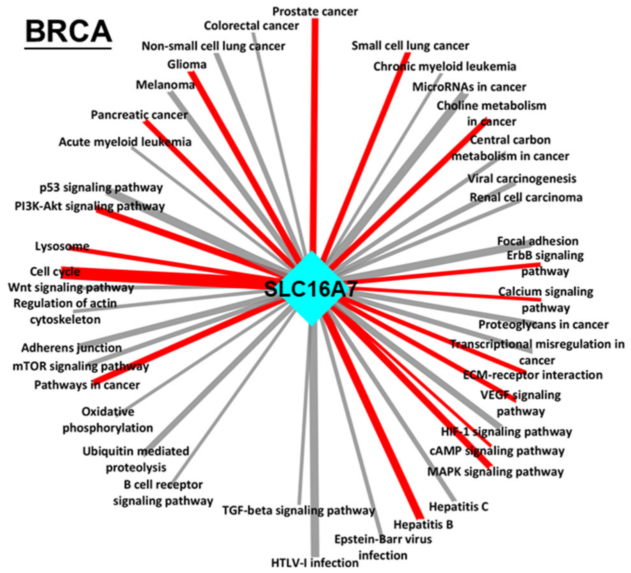

	Enriched	Non-enriched
Number of pathways in CaMPNets	16	25
Number of pathways not in CaMPNets	62	189

P value = 6×10^{-2}

LUAD

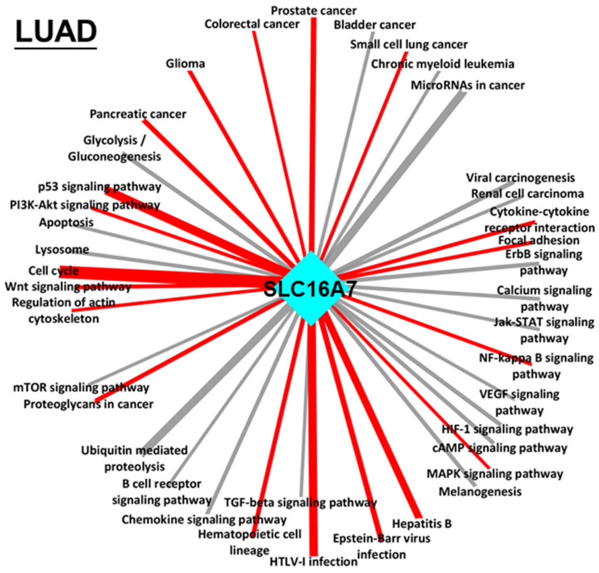

	Enriched	Non-enriched
Number of pathways in CaMPNets	19	20
Number of pathways not in CaMPNets	62	191

P value = 3×10^{-3}

MPP community-regulated pathway (filtered at enrichment $P \leq 0.05$)

Enriched pathway

Involvement ($-\log_{10}$ Enrichment *P*)

low High

Supplementary Figure 29. Enrichment of differentially expressed genes involved in the pathways of CaMPNets and other KEGG pathways in MDA-MB-231 cells and A549 cells subjected to CHRNA9 or SLC16A7 knockdown.

(**a**) CHRNA9 and (**b**) SLC16A7 CaMPNets in BRCA (left) and Lung Adenocarcinoma (LUAD; right) showing significantly enriched pathways (red lines, $P < 0.05$) via pathway enrichment analysis as calculated by hypergeometric distribution. To additionally validate the CaMPNet in a selective and systematic way, we performed microarray analysis to examine the effects of CHRNA9 and SLC16A7 knockdown in MDA-MB-231 and A549 cell lines treated with CRISPR/Cas9 targeting the CHRNA9 and SLC16A7 gene loci, respectively. In the pathway enrichment analysis, all differentially expressed genes (DEGs), upregulated DEGs, and downregulated DEGs were separately evaluated to elucidate all the possible regulations between the control cells and CHRNA9/SLC16A7 knockdown cells. Among 292 KEGG pathways, our results show that the percentages (≥39%) of enriched pathways in CHRNA9 and SLC16A7 CaMPNets in BRCA and LUAD, filtered at enrichment $P \leq 0.05$, were significantly higher than those (≤25%) not in CaMPNets ($P < 3 \times 10^{-2}$, Fisher's exact test) except for the SLC16A7 CaMPNet in BRCA (39% for CaMPNet versus 25% for the others, $P = 6 \times 10^{-2}$). For example, genes of the cell cycle pathway strongly associated with CHRNA9 and SLC16A7 communities in BRCA and LUAD were significantly altered in the MDA-MB-231 and A549 cells in which CHRNA9 and SLC16A7 were knocked down compared to those in the control. Differentially expressed genes, including upregulated and downregulated DEGs, were defined as those with at least a 1.5-fold expression change in the MDA-MB-231 and A549 cell lines. Gene expression in the control (no knockdown) cell line was compared with that in the CHRNA9 or SLC16A7 knockdown cell lines. The solid lines denote regulated pathways in CaMPNets filtered at an enrichment $P \leq 0.05$. The thickness of the lines is proportional to the significance of involvement ($-\log_{10}$ enrichment).

a

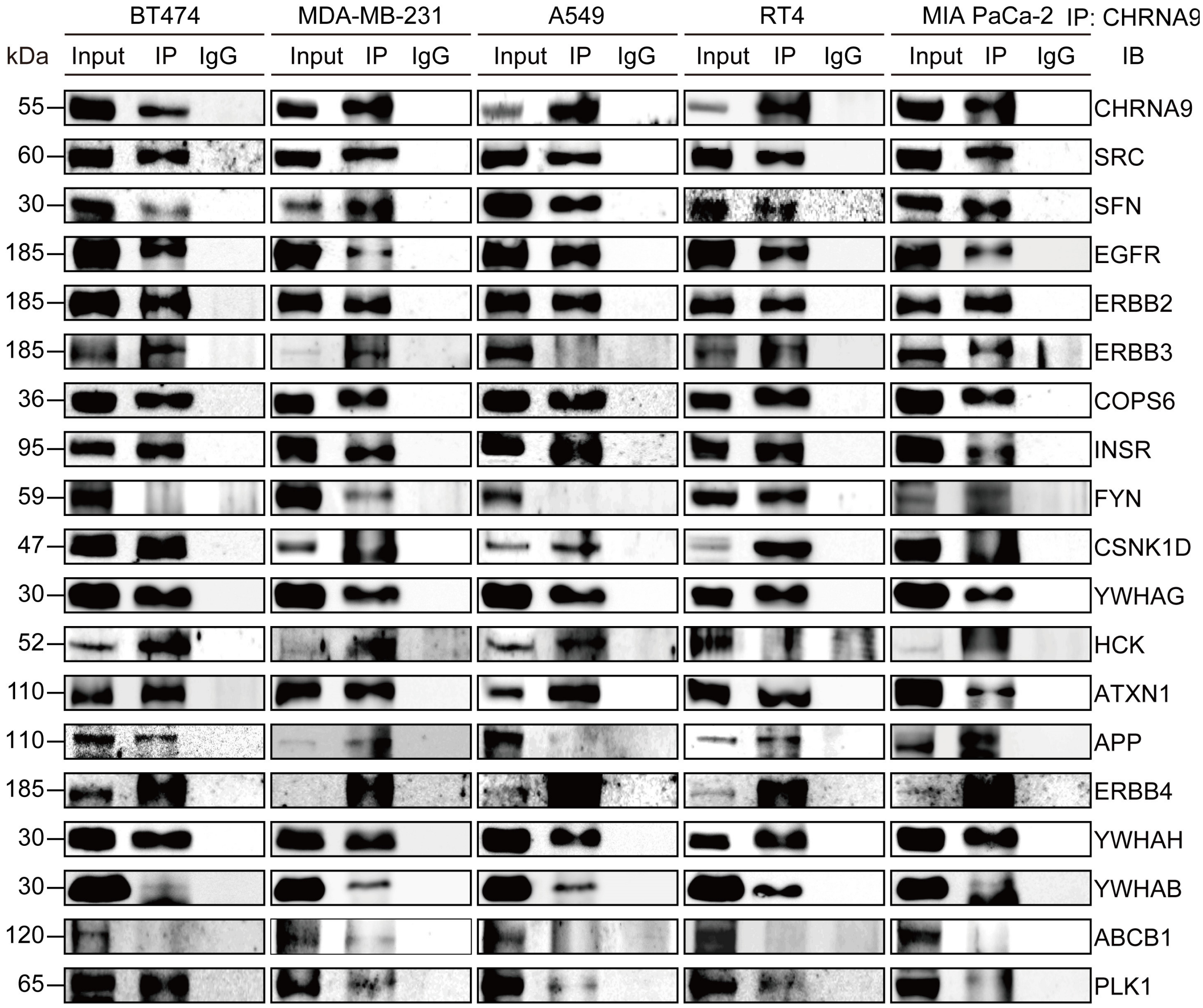
BT474
MDA-MB-231
A549
RT4
MIA PaCa-2
IP: CHRNA9
kDa
Input IP IgG
IB
55
60
30
185
185
185
36
95
59
47
30
52
110
110
185
30
30
120
65
CHRNA9
SRC
SFN
EGFR
ERBB2
ERBB3
COPS6
INSR
FYN
CSNK1D
YWHAG
HCK
ATXN1
APP
ERBB4
YWHAH
YWHAB
ABCB1
PLK1

b

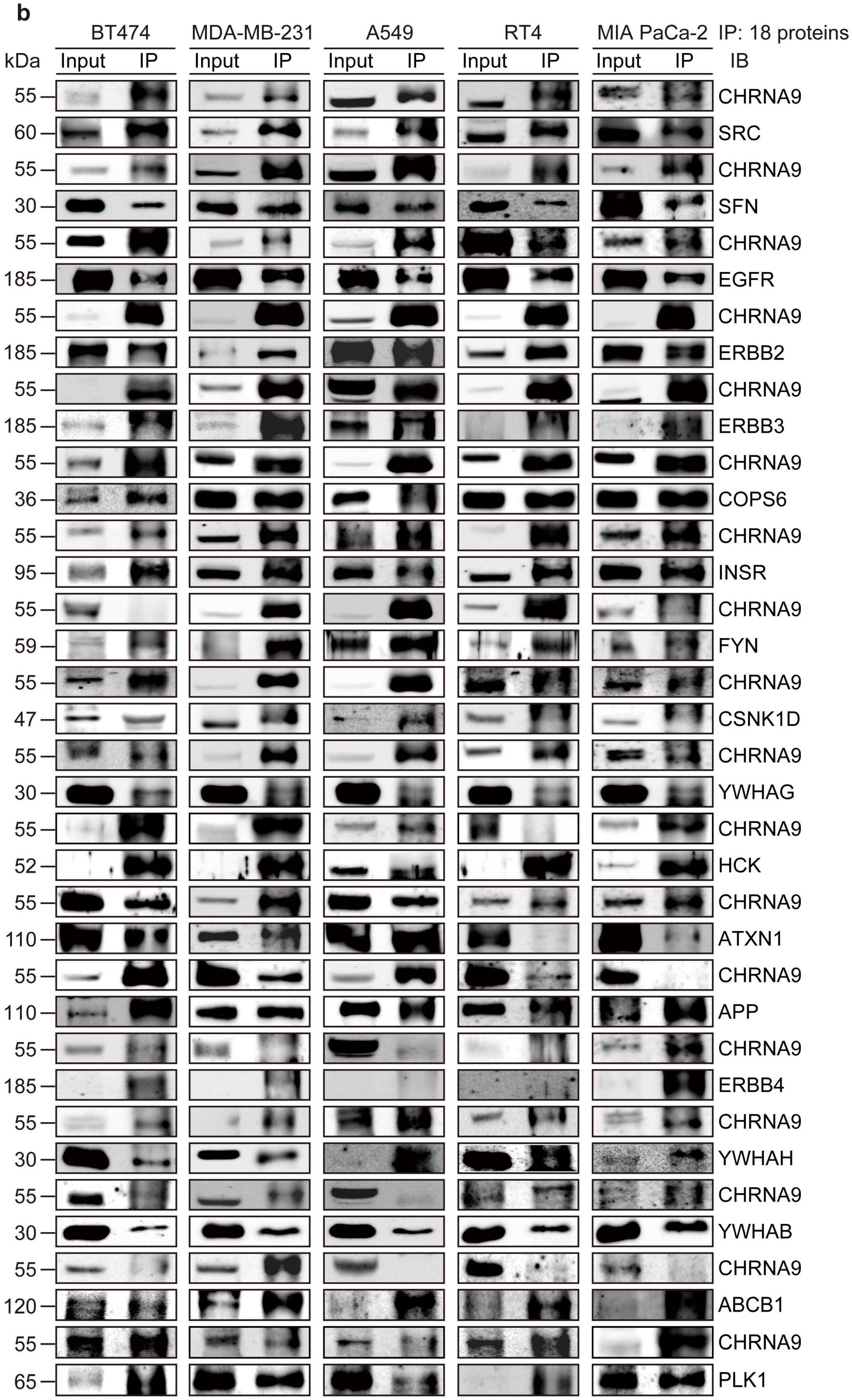

BT474
MDA-MB-231
A549
RT4
MIA PaCa-2
IP: 18 proteins
kDa
Input IP
IB
55 CHRNA9
60 SRC
55 CHRNA9
30 SFN
55 CHRNA9
185 EGFR
55 CHRNA9
185 ERBB2
55 CHRNA9
185 ERBB3
55 CHRNA9
36 COPS6
55 CHRNA9
95 INSR
55 CHRNA9
59 FYN
55 CHRNA9
47 CSNK1D
55 CHRNA9
30 YWHAG
55 CHRNA9
52 HCK
55 CHRNA9
110 ATXN1
55 CHRNA9
110 APP
55 CHRNA9
185 ERBB4
55 CHRNA9
30 YWHAH
55 CHRNA9
30 YWHAB
55 CHRNA9
120 ABCB1
55 CHRNA9
65 PLK1

c

	BT474		MDA-MB-231		A549		RT4		MIA PaCa-2	
IP / IB	CHRNA9	18 proteins	CHRNA9	18 proteins	CHRNA9	18 proteins	CHRNA9	18 proteins	CHRNA9	18 proteins
CHRNA9	5.91*	N/A	17.54*	N/A	27.62*	N/A	63.79*	N/A	12.42*	N/A
SRC	9.36*	55.83*	11.58*	25.87*	22.00*	28.92**	8.67*	73.31*	23.58*	35.37**
SFN	4.62*	51.29*	17.33*	27.57*	20.21*	42.81*	22.10**	69.80*	19.32*	40.64*
EGFR	12.25**	24.25*	4.11*	36.14*	9.48*	47.54*	7.42*	6.92*	5.23*	14.48*
ERBB2	9.55*	240.47*	9.37*	347.49*	9.22*	63.21*	8.87*	535.52*	12.07*	727.27*
ERBB3	11.16*	1762.8*	40.07*	51.38*	3.83*	27.35**	13.40*	311.68*	8.86*	173.50**
COPS6	10.10*	35.07*	11.33*	21.15*	10.62*	261.76*	14.70*	32.88*	7.22*	23.12*
INSR	13.02*	29.92*	9.61*	50.87**	16.09*	32.10**	14.33*	95.93*	6.98*	42.50**
FYN	2.48*	1.73*	4.24*	M.A.*	1.17*	M.A.*	17.43*	107.58*	M.A.*	2.82*
CSNK1D	11.74*	21.95*	M.A.*	M.A.*	20.72*	591.70*	45.38*	11.36**	M.A.*	10.75*
YWHAG	9.26*	13.16*	7.13*	124.89*	7.70*	30.98*	12.83*	29.50*	4.85*	11.61*
HCK	31.08*	42.85*	27.11*	43.35*	14.47*	18.94*	2.43*	2.36*	41.19*	36.98*
ATXN1	30.52**	17.48*	9.87*	33.86*	25.10*	18.94**	10.23*	17.91*	4.41*	15.74*
APP	16.77**	95.25*	21.53*	4.47*	2.49*	44.05*	17.38*	5.19*	M.A.*	0.43*
ERBB4	20.38*	22.70*	174.56*	2.65*	28.58*	1.86*	48.27*	M.A.*	31.50*	33.28*
YWHAH	10.32*	35.81*	9.31*	64.58*	7.44*	11.63*	16.50*	24.23*	9.49*	31.97*
YWHAB	2.70*	4.03*	3.53*	12.83*	3.44*	0.89*	3.98*	8.80*	2.75*	16.53*
ABCB1	2.50*	2.52*	3.05*	25.06*	2.63*	1.61*	1.47*	1.64*	1.02*	2.44*
PLK1	10.43*	11.37*	6.64*	7.59*	3.21*	7.41*	6.77*	12.37**	2.87*	27.94*

Supplementary Figure 30. Co-immunoprecipitation of CHRNA9 and 18 representative binding partners.

Cell protein lysates from breast (BT474 and MDA-MB-231), lung (A549), bladder (RT4) and pancreatic (MIA PaCa-2) cancer cells were collected and incubated with IgG beads and (**a**) CHRNA9 antibody or (**b**) 18 candidate protein antibodies overnight. The protein/antibody/bead mixtures were washed and immunoblotted with a light chain specific secondary antibody, followed by a CHRNA9 antibody or interacting protein antibody hybridization (**Supplementary Table 7**). IgG immunoprecipitation served as a negative control. Each protein was identified by the input control and its molecular weight. IB: immunoblot. Source data are provided as a Source Data file. (**c**) To define positive interactions, the band intensities of the immunoprecipitated proteins and their input loading

controls on blots were measured using ImageJ software and the IP ratio was calculated (*IPR*, %; details in Methods). The IP bands that can be identified at a minimum level are approximately 3% (e.g., ABCB1 in MDA-MB-231 cells and PLK1 in A549 cells); therefore, we used the IP ratio as the threshold to determine positive interactions. A candidate that passes the threshold of both reciprocal IP assays is considered a positive interaction. The negative interactions are colored blue. Here, we found that the results of the IP analysis are similar to those of the FRET efficiency analysis. For each input loading control, 20 μg and 50 μg of protein are indicated by an asterisk and a double asterisk, respectively. M.A.: misalignment.

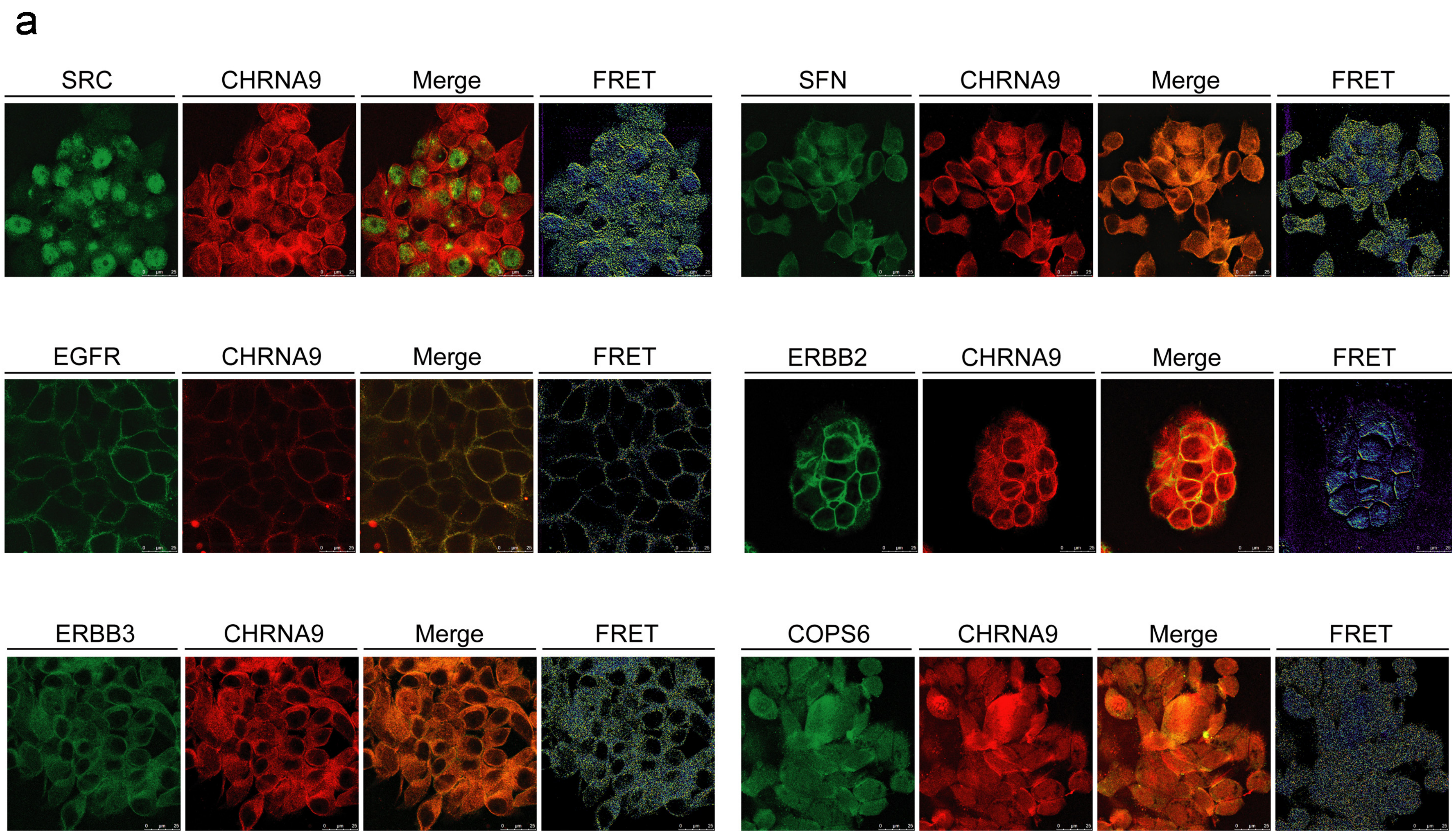
a
SRC
CHRNA9
Merge
FRET
SFN
CHRNA9
Merge
FRET
EGFR
CHRNA9
Merge
FRET
ERBB2
CHRNA9
Merge
FRET
ERBB3
CHRNA9
Merge
FRET
COPS6
CHRNA9
Merge
FRET

a (continued)

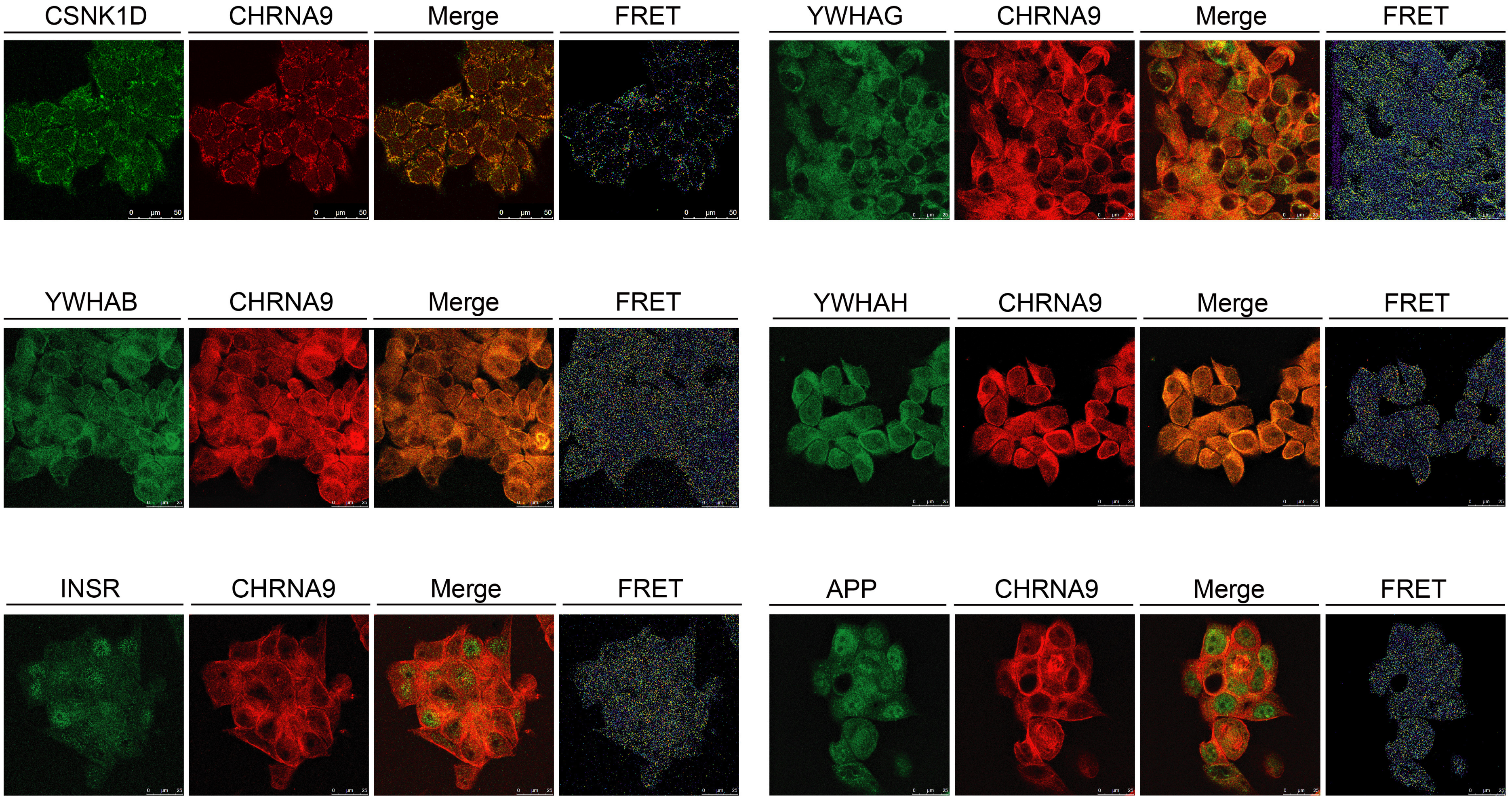
CSNK1D
CHRNA9
Merge
FRET
YWHAG
CHRNA9
Merge
FRET
YWHAB
CHRNA9
Merge
FRET
YWHAH
CHRNA9
Merge
FRET
INSR
CHRNA9
Merge
FRET
APP
CHRNA9
Merge
FRET

a (continued)

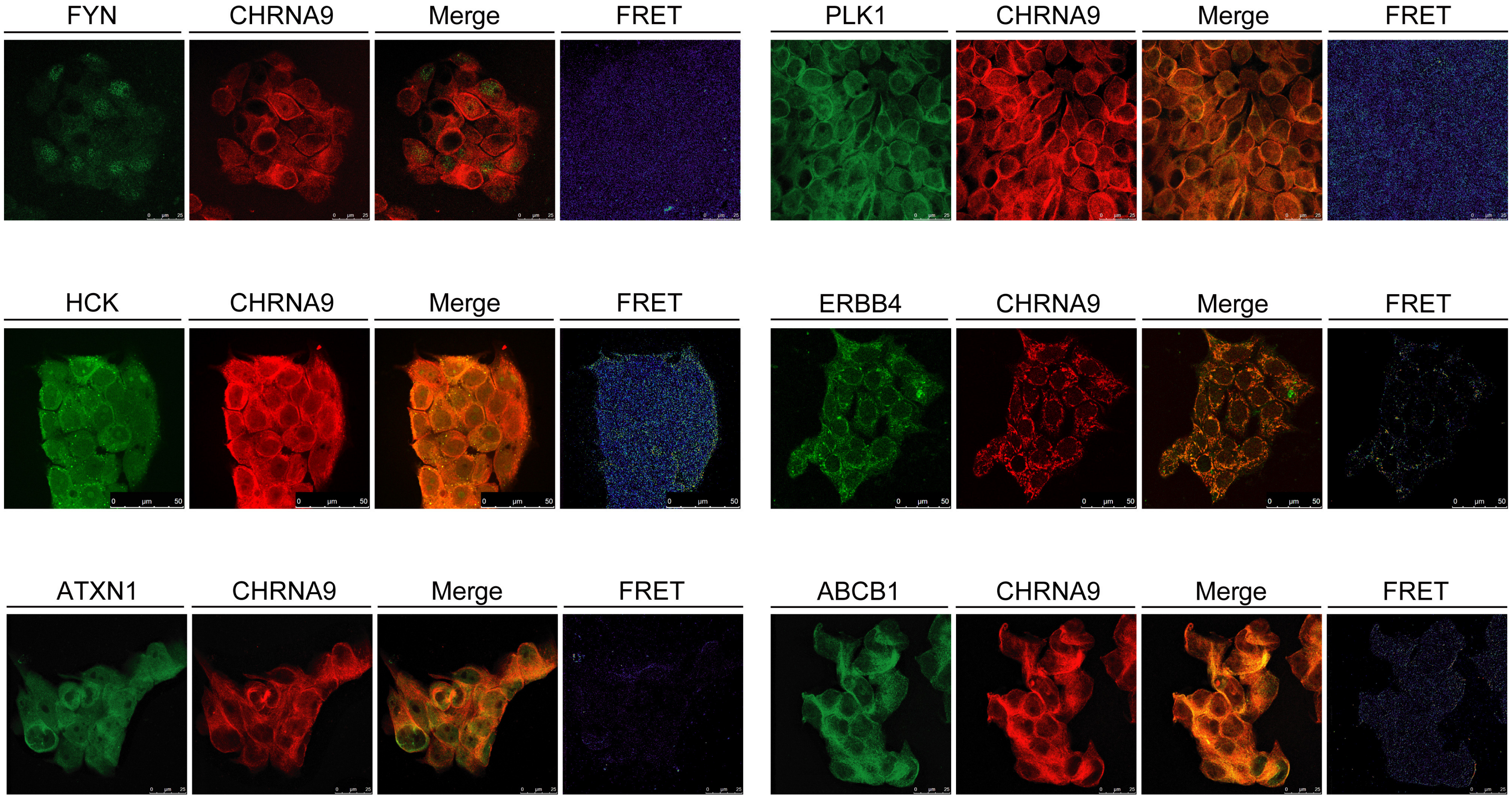
FYN
CHRNA9
Merge
FRET
PLK1
CHRNA9
Merge
FRET
HCK
CHRNA9
Merge
FRET
ERBB4
CHRNA9
Merge
FRET
ATXN1
CHRNA9
Merge
FRET
ABCB1
CHRNA9
Merge
FRET

b

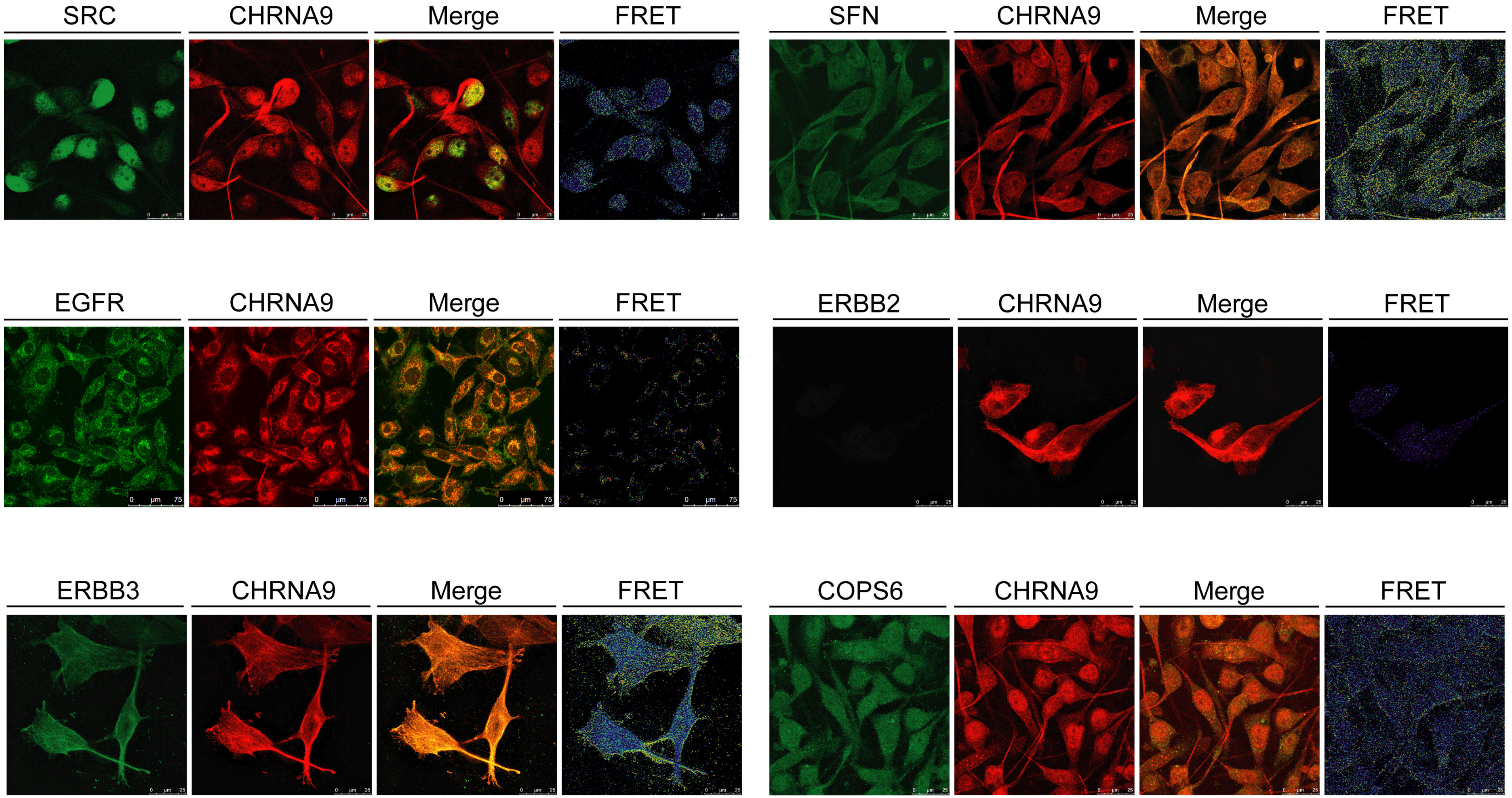
SRC
CHRNA9
Merge
FRET
SFN
CHRNA9
Merge
FRET
EGFR
CHRNA9
Merge
FRET
ERBB2
CHRNA9
Merge
FRET
ERBB3
CHRNA9
Merge
FRET
COPS6
CHRNA9
Merge
FRET

b (continued)

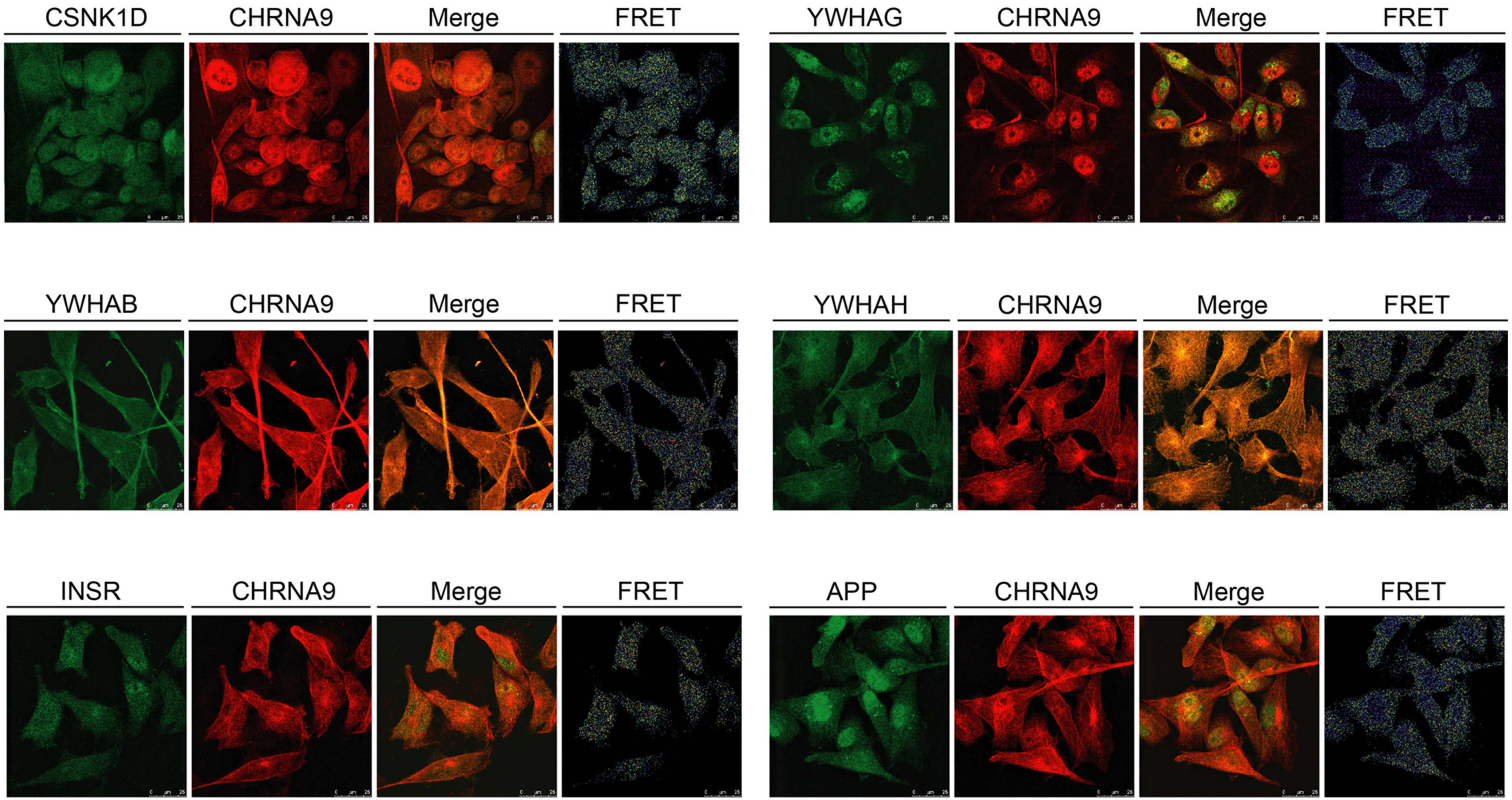
CSNK1D
CHRNA9
Merge
FRET
YWHAG
CHRNA9
Merge
FRET
YWHAB
CHRNA9
Merge
FRET
YWHAH
CHRNA9
Merge
FRET
INSR
CHRNA9
Merge
FRET
APP
CHRNA9
Merge
FRET

b (continued)

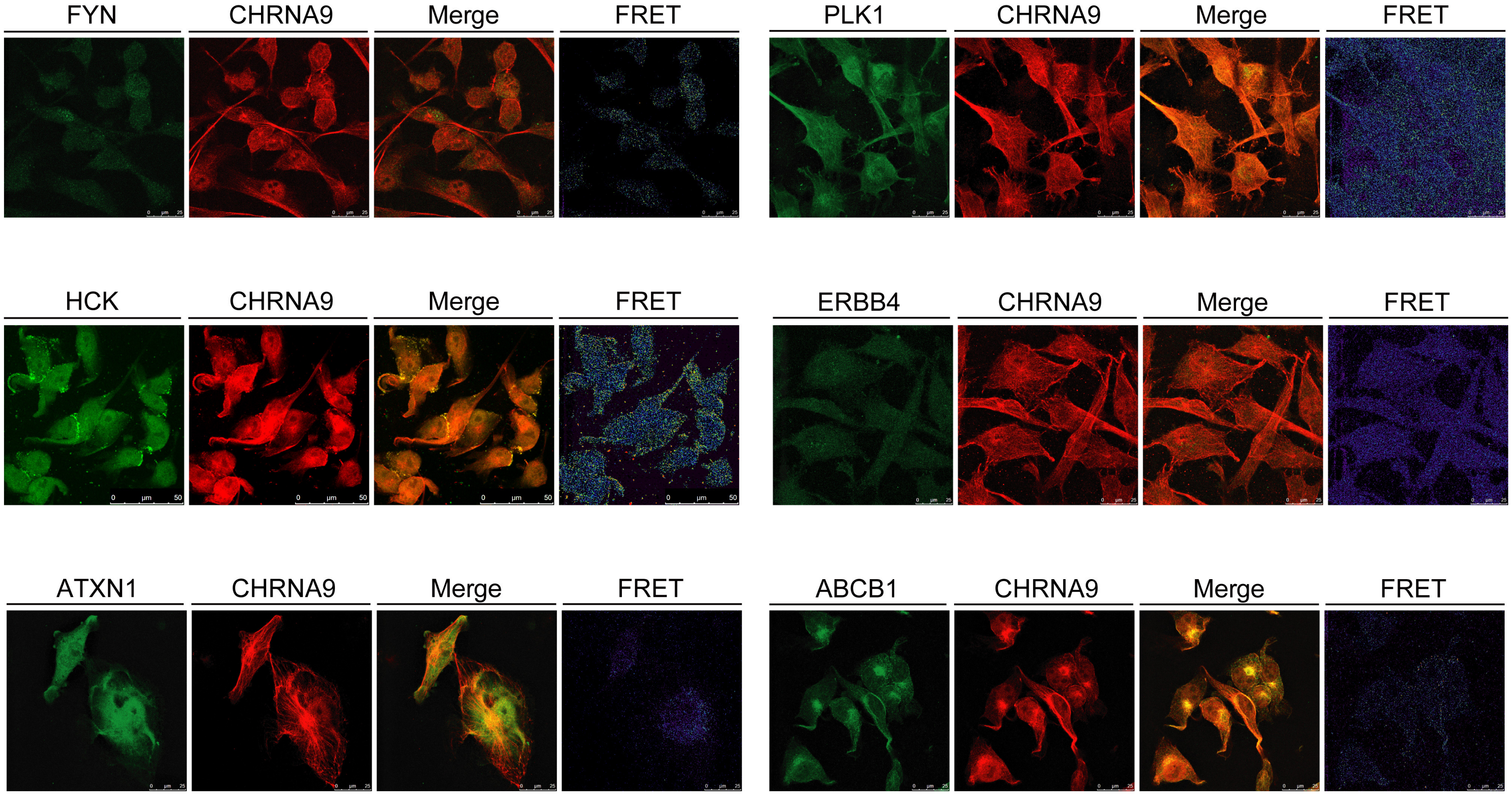
FYN
CHRNA9
Merge
FRET
PLK1
CHRNA9
Merge
FRET
HCK
CHRNA9
Merge
FRET
ERBB4
CHRNA9
Merge
FRET
ATXN1
CHRNA9
Merge
FRET
ABCB1
CHRNA9
Merge
FRET

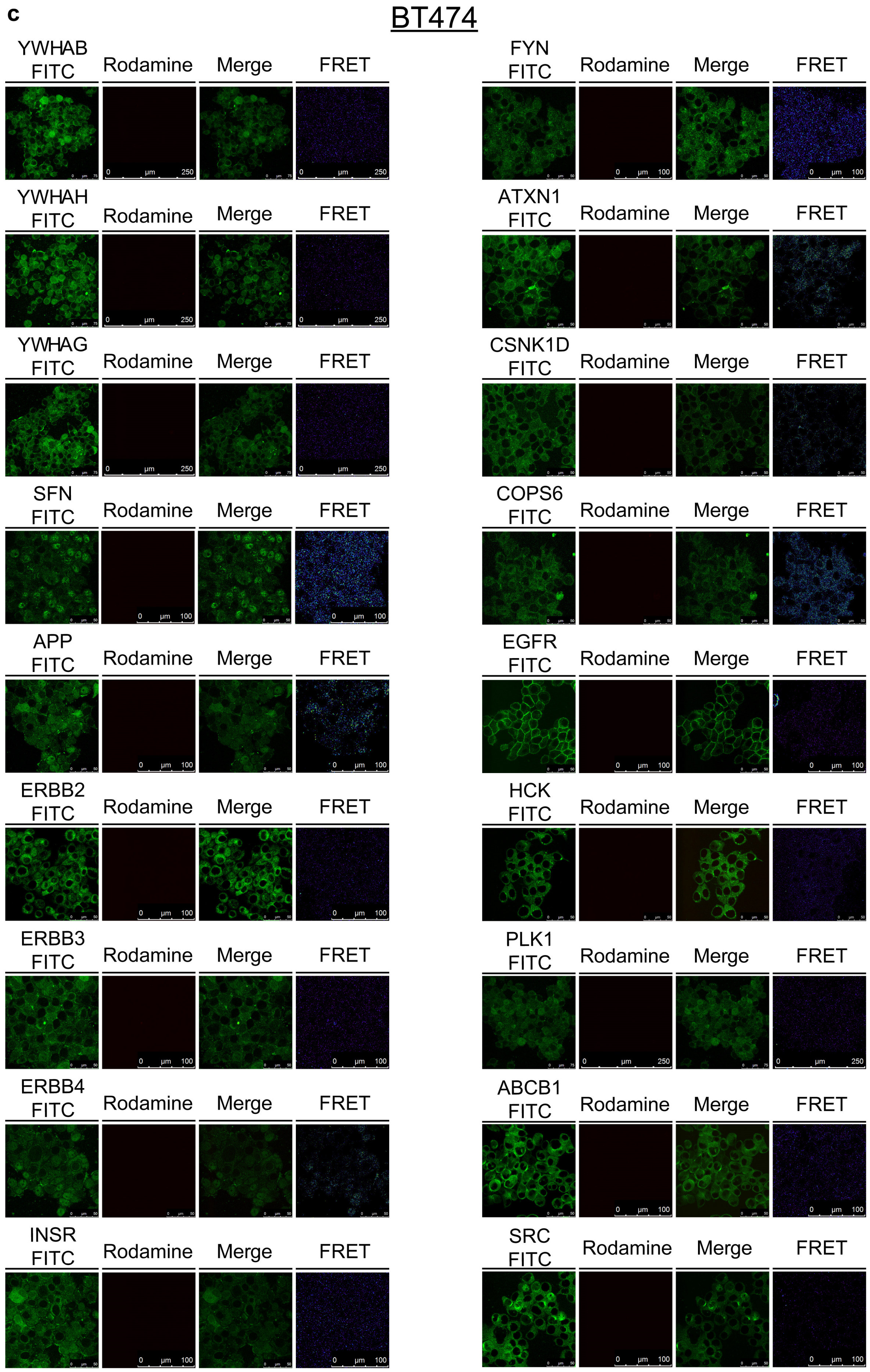
c
BT474
YWHAB FITC
Rodamine
Merge
FRET
YWHAH FITC
YWHAG FITC
SFN FITC
APP FITC
ERBB2 FITC
ERBB3 FITC
ERBB4 FITC
INSR FITC
FYN FITC
ATXN1 FITC
CSNK1D FITC
COPS6 FITC
EGFR FITC
HCK FITC
PLK1 FITC
ABCB1 FITC
SRC FITC

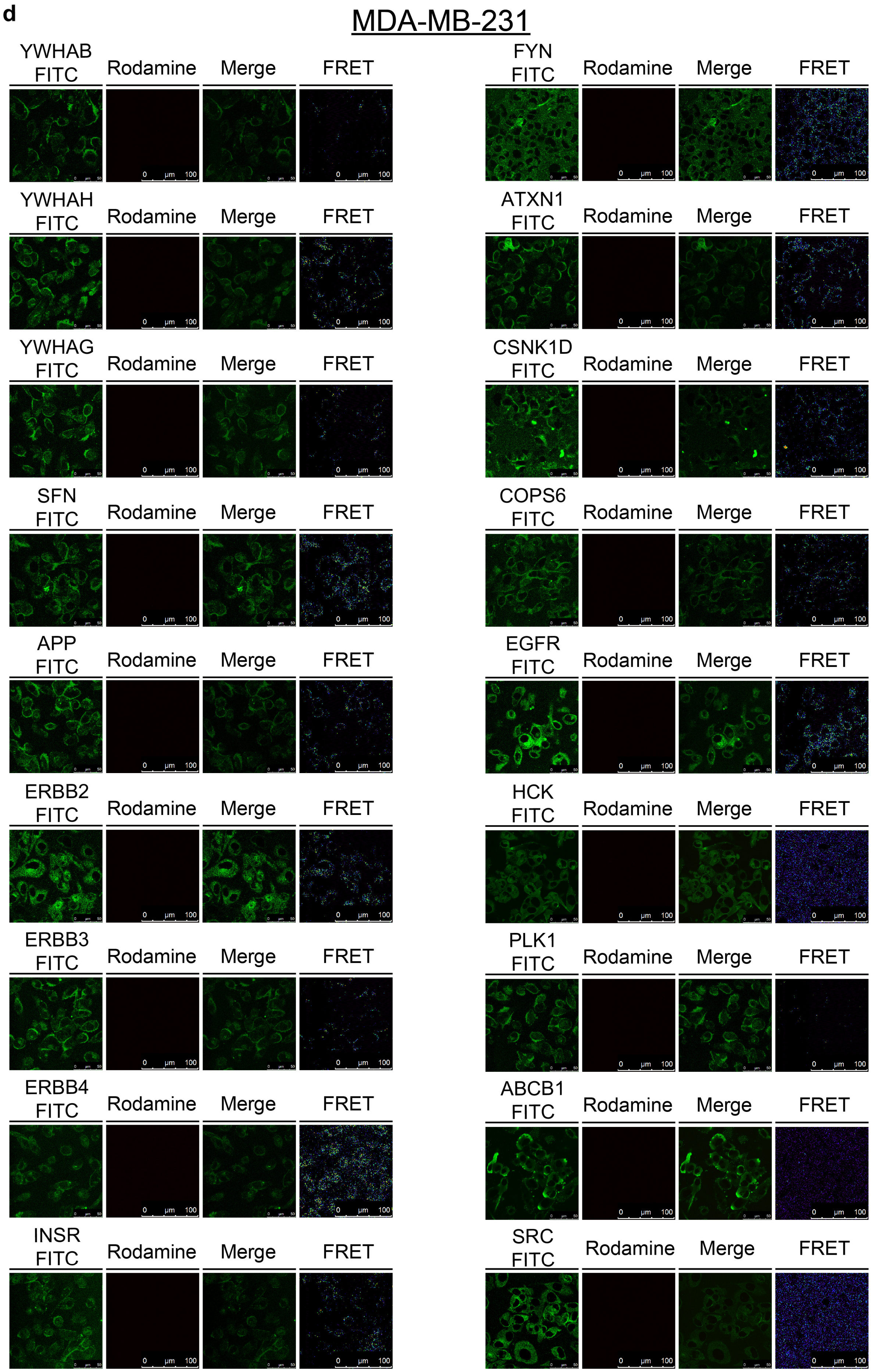
d
MDA-MB-231
YWHAB FITC
Rodamine
Merge
FRET
YWHAH FITC
YWHAG FITC
SFN FITC
APP FITC
ERBB2 FITC
ERBB3 FITC
ERBB4 FITC
INSR FITC
FYN FITC
ATXN1 FITC
CSNK1D FITC
COPS6 FITC
EGFR FITC
HCK FITC
PLK1 FITC
ABCB1 FITC
SRC FITC
0 µm 100

e

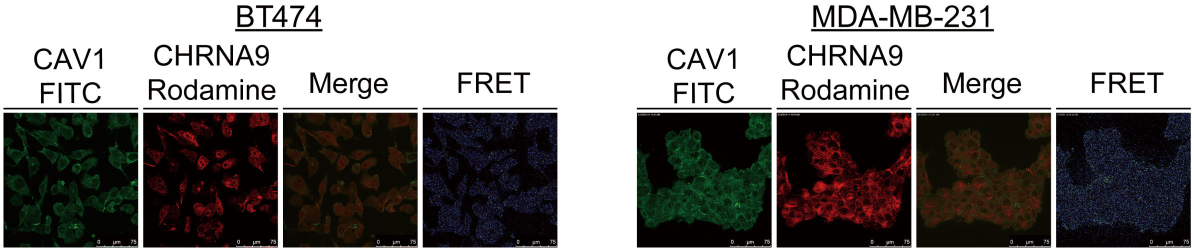

f

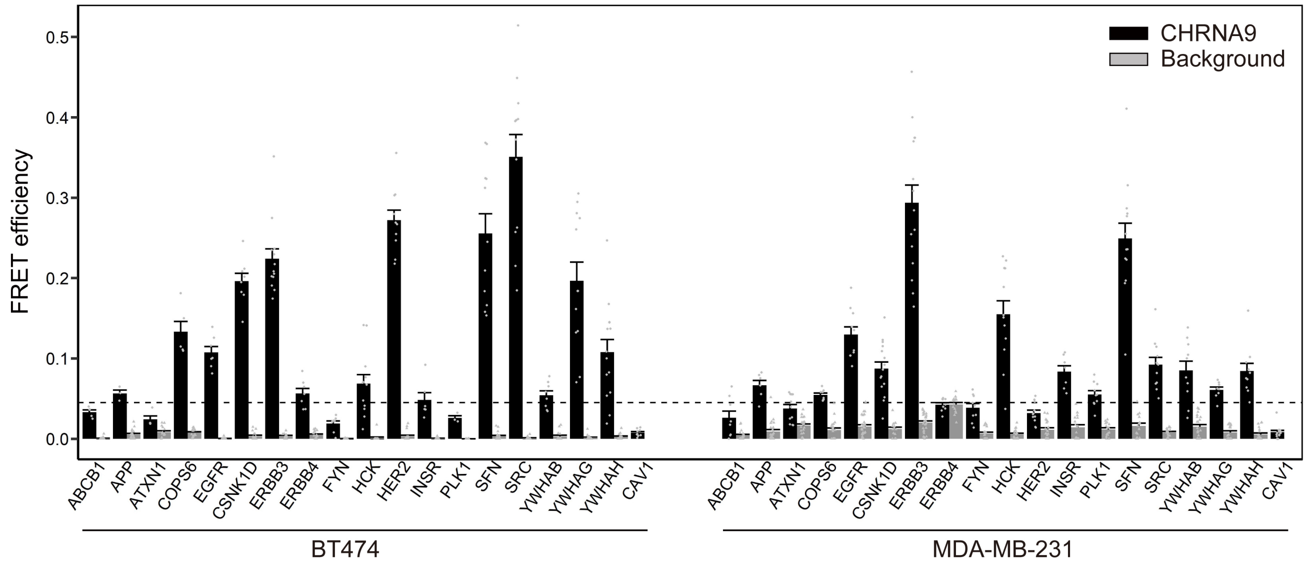

Supplementary Figure 31. Förster resonance energy transfer (FRET) analysis of CHRNA9 and 18 representative binding partners in BT474 and MDA-MB-231 cell lines.

(**a**) BT474 and (**b**) MDA-MB-231 cells were hybridized with CHRNA9 and interacting proteins using secondary rhodamine and fluorescein isothiocyanate (FITC) dyes. The above cells were then examined under a confocal microscope imaging system via FRET module analysis (details in Methods). The photos show interacting protein (green) expression, CHRNA9 (rhodamine conjugation; red) expression, merged images, and FRET efficiency between CHRNA9 and the interacting proteins. The green/yellow/red colors represent the intensity of FRET. Acceptor FRET imaging experiments were performed on a Leica TCS SP5 Confocal Spectral Microscope Imaging System (Leica Microsystems), and the acceptor photobleaching Leica software module was used in the FRET assay. Background control of (**c**) BT474 and (**d**) MDA-MB-231 cells were hybridized with 18 protein antibodies and secondary FITC dye, followed by rhodamine hybridization without the CHRNA9 antibody. The

black/blue colors represent the background signal in the FRET analysis. (**e**) FRET efficiency between CHRNA9 and CAV1 (caveolin-1) in BT474 and MDA-MB-231 cells as a negative control. (**f**) Comparison of FRET efficiencies between CHRNA9 and 18 interacting candidates, their background signals, and the negative control on BT474 and MDA-MB-231 cells. According to the background signals of 18 interacting candidates and the signal between CHRNA9 and CAV1 (negative control) in both BT474 and MDA-MB-231 cells, the maximum FRET efficiency (i.e., ERBB4 in MDA-MB-231) with a standard error was selected as the threshold (0.045; dashed line) to define positive interactions. The error bars indicate the mean ± standard error. Source data are provided as a Source Data file.

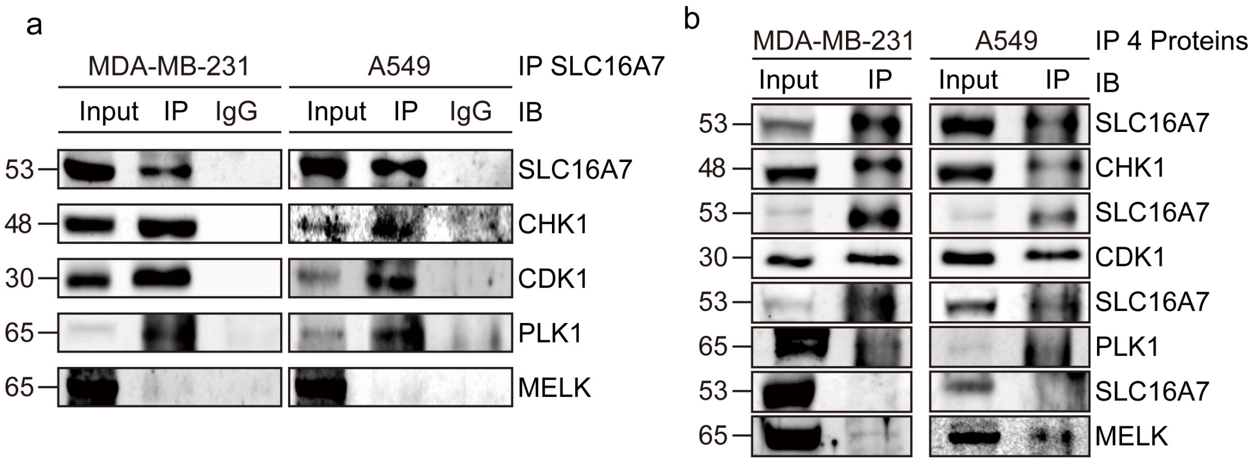

c

IB \ IP	MDA-MB-231 SLC16A7	MDA-MB-231 4 proteins	A549 SLC16A7	A549 4 proteins
SLC16A7	7.66	N/A	9.55	N/A
CHK1	13.34	18.68	25.25	9.93
CDK1	16.21	59.89	26.33	41.27
PLK1	62.57	61.67	57.57	13.96
MELK	1.15	0.87	0.75	1.48

d

Cell line	Assay	Number of validated/tested proteins (n)	Ratio
MDA-MB-231	IP	3/4	75.0%
A549	IP	3/4	75.0%

e

Four selected partner candidates of SLC16A7	MDA-MB-231	A549
	IP	IP
CHK1	+	+
CDK1	+	+
PLK1	+	+
MELK	-	-

Supplementary Figure 32. Co-immunoprecipitation of SLC16A7 and four selected interacting partners.

Cell protein lysates from breast (MDA-MB-231) and lung (A549) cancer cells were collected and incubated with IgG beads and (**a**) SLC16A7 antibody or (**b**) individual candidate protein antibodies overnight. The protein/antibody/bead mixtures were washed and immunoblotted with a light chain-specific secondary antibody, followed by a SLC16A7 antibody or interacting protein antibody hybridization. The IgG immunoprecipitation served as a negative control. Each protein was identified by 20 µg protein input control and its molecular weight. Source data are provided as a Source Data file. (**c**) To define positive interactions, the band intensities of the immunoprecipitated proteins and their input loading controls on blots were measured using ImageJ software and the IP ratio was calculated (*IPR*, %; details in Methods). Similar to the IP analysis of CHRNA9, we used the same

threshold (>3%) to determine positive interactions. A candidate that passes the threshold of both reciprocal IP assays is considered a positive interaction. The negative interactions are colored blue. (**d**) Summary table of the IP results. (**e**) Percentage of interacting proteins that associate with SLC16A7 as determined by the IP assay.

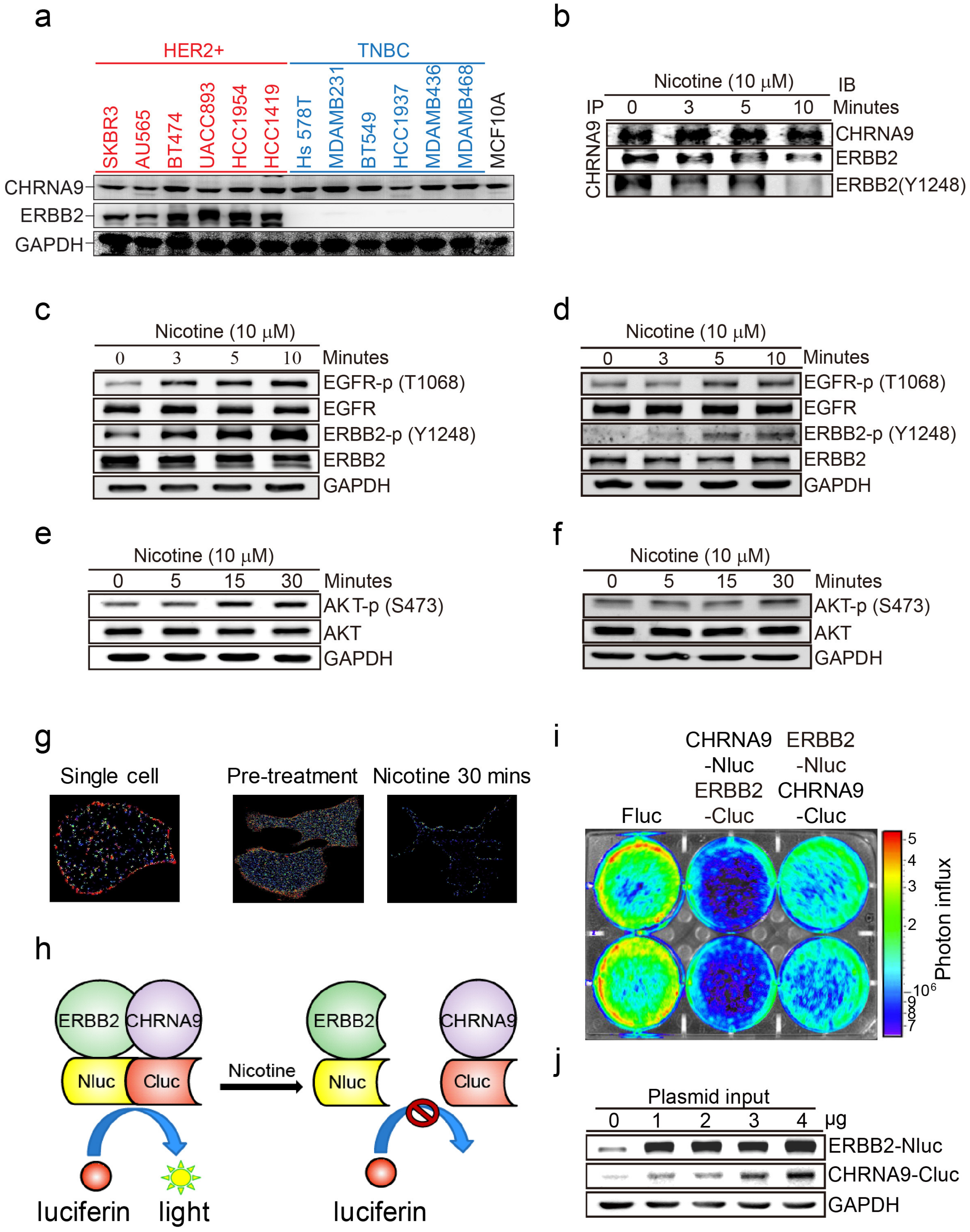

Supplementary Figure 33. Analysis of interaction between CHRNA9 and ERBB2.

(**a**) Protein expression levels of CHRNA9, ERBB2 and GAPDH in one non-malignant breast cell lines (MCF-10A), six HER2-enriched (SKBR3, AU565, BT474, UACC893, HCC1954, and HCC1419) and

six TNBC (Hs578T, MDA-MB-231, BT549, HCC1937, MDA-MB-436, and MDA-MB-468) cancer cell lines. (**b**) Time-dependent IP assay carried out with a CHRNA9 antibody on BT474 cells. ERBB2 and phospho-ERBB2 (Y1248) antibodies were immunoblotted for PPIs after treatment with 10 μM nicotine in a time-dependent manner from 0-10 minutes. Nicotine-induced activation of EGFR and ERBB2 receptors in a time-dependent manner. (**c**) BT474 and (**d**) MDA-MB-231 cells were exposed to 10 μM nicotine from 0-10 minutes and immunoblotted for the phospho-EGFR (T1068) and phospho-ERBB2 (Y1248) proteins. Total-EGFR, total-ERBB2, and GAPDH were used as internal controls. (**e**) BT474 and (**f**) MDA-MB-231 cell lysates treated with nicotine for 0-30 minutes were immunoblotted for phospho-AKT (S473) protein expression. Total-AKT and GAPDH were used as internal controls. (**g**) Plasmids containing ERBB2-YFP and CHRNA9-CFP were co-transfected into MDA-MB-231 cells for the FRET (Förster resonance energy transfer) activity assay. Higher FRET activity is indicated in red, and lower FRET activity is shown in blue. The presented single MDA-MB-231 cell demonstrates FRET activities with or without 10 μM nicotine treatment. (**h**) Schematic representation of the CHRNA9 and ERBB2 split luciferase complementation assay. Interaction between the CHRNA9-Cluc and ERBB2-Nluc proteins bring the luciferase fragments in close enough proximity to measure the activity by luciferin addition. (**i**) Split luciferase complementation assay optimization was carried out using protein expression analysis of the pairs CHRNA9-Nluc/ERBB2-Cluc or CHRNA9-Cluc/ERBB2-Nluc in MDA-MB-231 cells. Luciferase activity was measured by IVIS (non-invasion *in vivo* imaging system), demonstrated in the color bar. In order to find out the optimized interaction paring of both ERBB2 and CHRNA9 fusion proteins with luciferase gene fragments, we tested the luciferase activity on MDA-MB-231 cells with ERBB2/Nluc-CHRNA9/Cluc and ERBB2/Cluc-CHRNA9/Nluc expression. Observation via a non-invasive *in vivo* imaging system (IVIS) showed that the ERBB2/Nluc and CHRNA9/Cluc combinations expressed higher luciferase activity than combinations of the other fusion proteins. (**j**) Overexpression of the CHRNA9-Cluc and ERBB2-Nluc fusion proteins in MDA-MB-231 cells as determined by western blot. Each plasmid was

transfected dose-dependently, and protein expression was measured three days after electroporation. CHRNA9 and ERBB2 antibodies were used to immunoblot for CHRNA9 and ERBB2 fusion protein expression. Expression levels of the fusion proteins ERBB2/Nluc and CHRNA9/Cluc gradually increased in MDA-MB-231 cells following dose-dependent plasmid input.

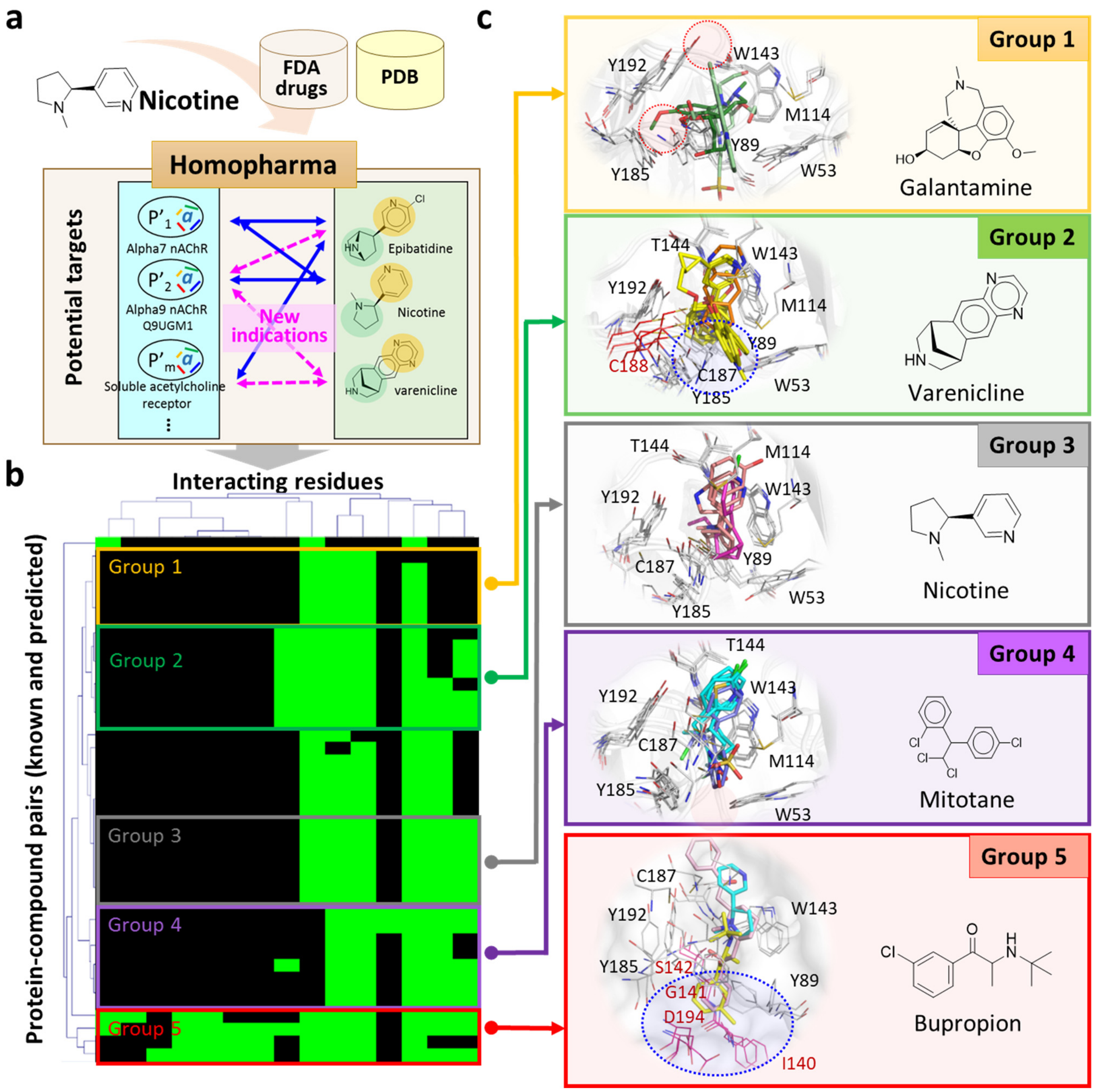

Supplementary Figure 34. Identification of CHRNA9 inhibitors among FDA-approved drugs using Homopharma. (**a**) Potential CHRNA9 inhibitors identified by our previous method, homopharma, for drug repurposing according to the interaction similarity between 1,543 FDA-approved drugs and nicotinic acetylcholine receptors (nAChR) structures (e.g., acetylcholine-binding protein with nicotine; PDB code: 1UW6). (**b**) Five groups of 41 nAChR and compound complexes clustered by their interaction similarity score[57] (black: interaction energy is zero). Group 3 comprises the binding modes of nicotine and nAChRs, and other groups are relative to modes for the other

inhibitors. (**c**) Superimposed structures of five groups and their conserved interacting residues (residue numbering of the PDB code: 1UW6). Group 3 is considered a reference template due to the binding mode of nAChR with the ligand nicotine. In comparison with group 3, complexes in group 1 lose interactions between contacted residues (e.g., T144 and C187) and ligands (e.g., galantamine); complexes in group 2 have additional interactions between residue C188 and target compounds (e.g., varenicline); complexes in group 4 lack Y89 to interact with ligands (e.g., mitotane); and complexes in group 5 comprise the additional residues I140, G141, S142, and D194, involved in interactions with potential inhibitors (e.g., bupropion).

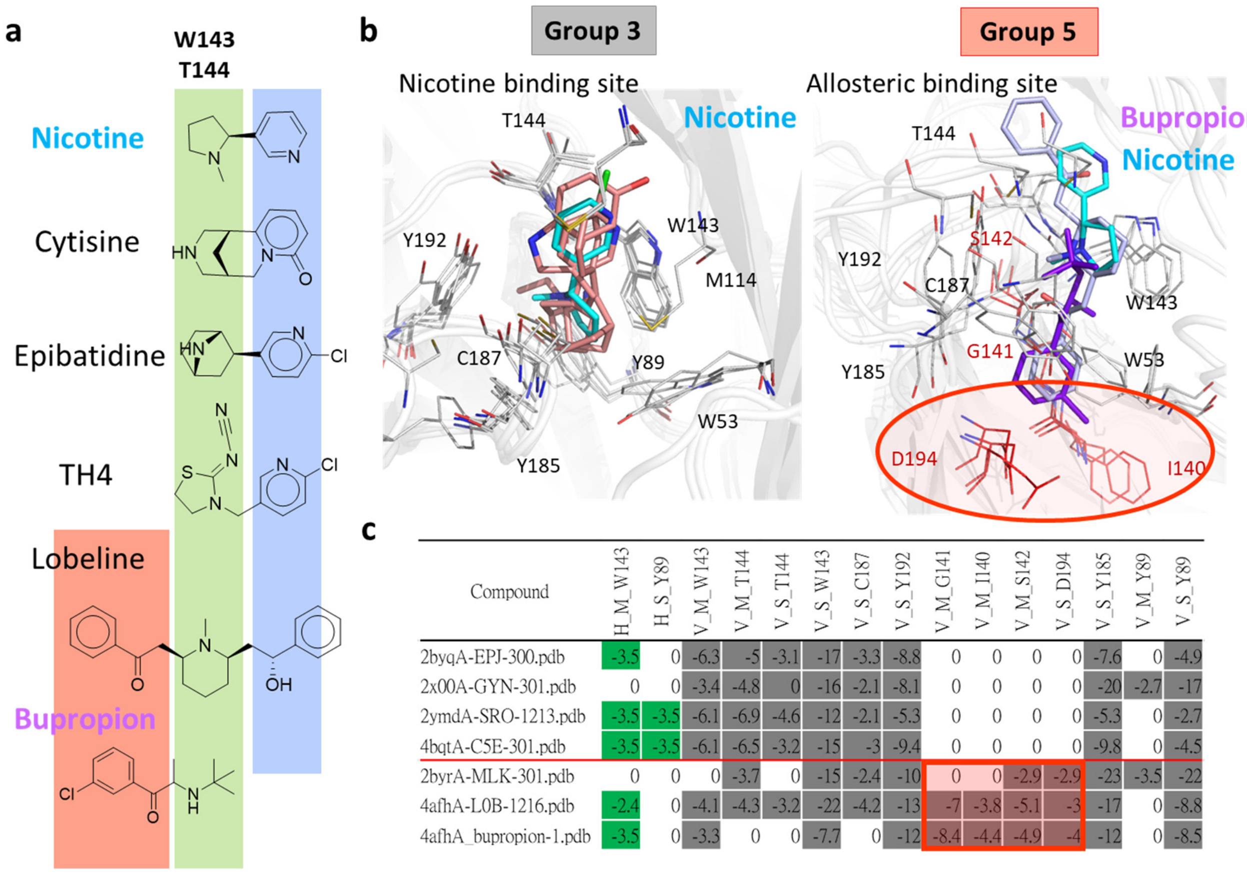

Compound	H_M_W143	H_S_Y89	V_M_W143	V_M_T144	V_S_T144	V_S_W143	V_S_C187	V_S_Y192	V_M_G141	V_M_I140	V_M_S142	V_S_D194	V_S_Y185	V_M_Y89	V_S_Y89
2byqA-EPJ-300.pdb	-3.5	0	-6.3	-5	-3.1	-17	-3.3	-8.8	0	0	0	0	-7.6	0	-4.9
2x00A-GYN-301.pdb	0	0	-3.4	-4.8	0	-16	-2.1	-8.1	0	0	0	0	-20	-2.7	-17
2ymdA-SRO-1213.pdb	-3.5	-3.5	-6.1	-6.9	-4.6	-12	-2.1	-5.3	0	0	0	0	-5.3	0	-2.7
4bqtA-C5E-301.pdb	-3.5	-3.5	-6.1	-6.5	-3.2	-15	-3	-9.4	0	0	0	0	-9.8	0	-4.5
2byrA-MLK-301.pdb	0	0	0	-3.7	0	-15	-2.4	-10	0	0	-2.9	-2.9	-23	-3.5	-22
4afhA-L0B-1216.pdb	-2.4	0	-4.1	-4.3	-3.2	-22	-4.2	-13	-7	-3.8	-5.1	-3	-17	0	-8.8
4afhA_bupropion-1.pdb	-3.5	0	-3.3	0	0	-7.7	0	-12	-8.4	-4.4	-4.9	-4	-12	0	-8.5

Supplementary Figure 35. Comparison of binding modes between potential inhibitors and nAChRs in group 3 (e.g., nicotine in CHRNA9) and group 5 (e.g., bupropion in CHRNA9). (**a**) Compound structures of potential CHRNA9 inhibitors in groups 3 and 5. Compared with nicotine and other inhibitors in group 3, bupropion belongs to group 5 and contains the moiety 1-(3-chlorophenyl)propan-1-one, which can bind an additional subpocket (orange) in CHRNA9. (**b**) Comparison of binding environments for groups 3 and 5. Inhibitors in group 5 not only bind contact residues, such as W143 and Y192 that also share interactions with compounds in group 3, but also comprise a moiety extending to an additional subpocket containing residues I140, G141, S142, and D194 (red circle) as a potential allosteric binding site. (**c**) Interaction profile showing hydrogen bonds and van der Waal forces between docked poses of these compounds in groups 3 and 5.

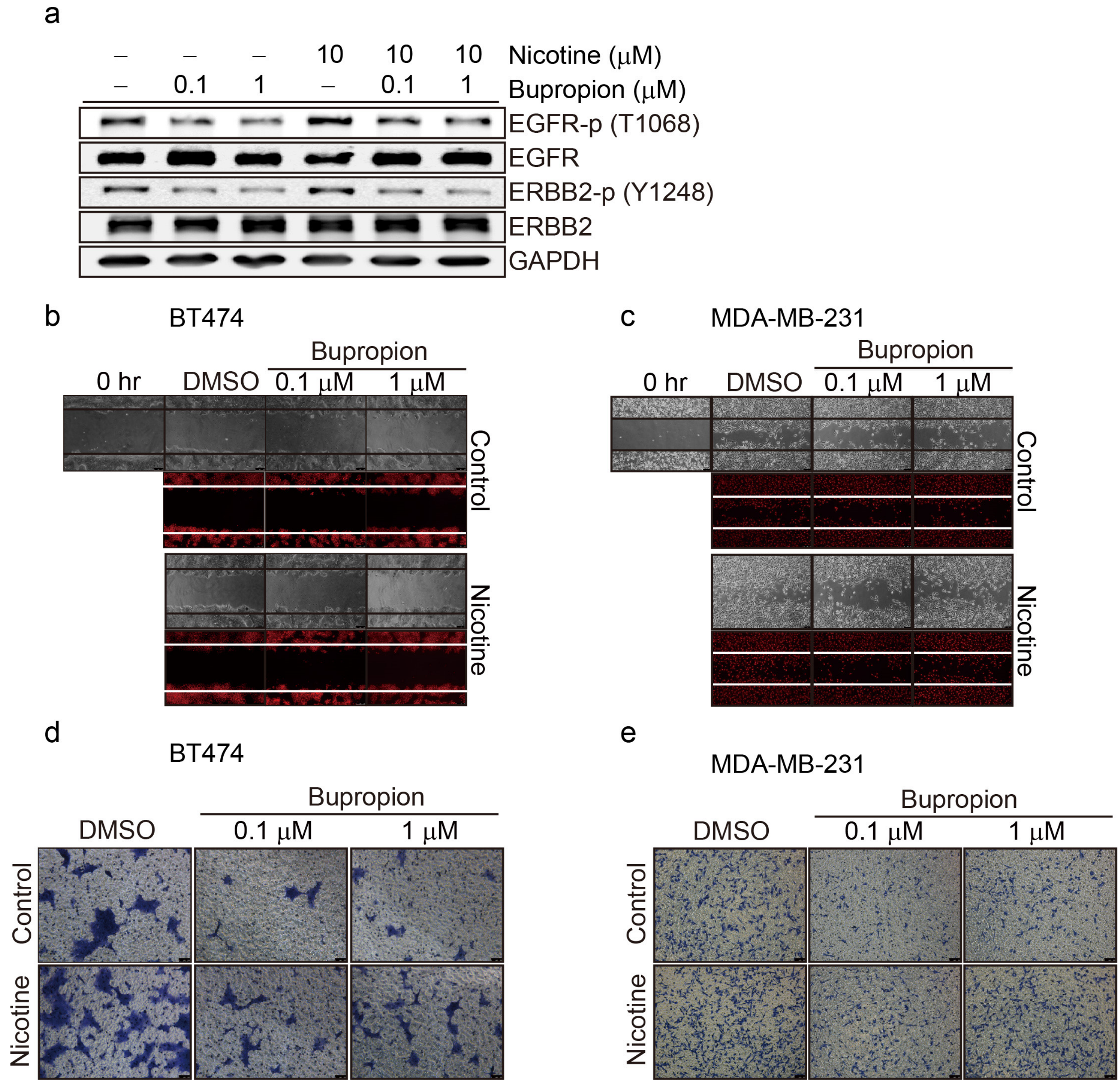

Supplementary Figure 36. Nicotine-induced ERBB2 phosphorylation attenuation and migration/invasion inhibition effects of bupropion on BT474 and/or MDA-MB-231 cells.

(**a**) Bupropion as an inhibitor to attenuate nicotine-induced EGFR and ERBB2 phosphorylation. BT474 cells were treated with 10 μM nicotine with or without 0.1 and 1 μM bupropion, and pretreatment with bupropion could inhibit nicotine-induced ERBB2 (Y1248) activation. GAPDH served as the internal control. Migration inhibition effect of bupropion on (**b**) BT474 and (**c**)MDA-MB-231. A cell migration assay was terminated with bupropion pretreatment at 0.1 and 1 μM with or without 10 μM nicotine. Cell photos were taken 24 hours for BT474 cells and 12 hours for MDA-MB-

231 cells after drug addition. The cells were fixed with formaldehyde and stained with PI (red) for 30 minutes. The statistical analysis is shown in **Figure 7c,d**. Invasion inhibition effect of bupropion on (**d**) BT474 and (**e**) MDA-MB-231. Cells were placed onto matrigel invasion chambers in serum-starved medium, and cells in the lower chambers were cultured using normal medium. Media in both the upper and lower chambers contained 0.1 and 1 μM bupropion, and the cells were either treated or not treated nicotine (10 μM) for 72 hours for BT474 cells and 48 hours for MDA-MB-231 cells. The upper chambers were washed with PBS and fixed with formaldehyde for 30 minutes. Cells in the upper chambers were stained with crystal violet for 2 hours, and photos were acquired under a microscope. The numbers of migrated and invaded cells were calculated by ImageJ software, and the experiments were repeated three times. The statistical analysis is shown in **Figure 7e,f**.

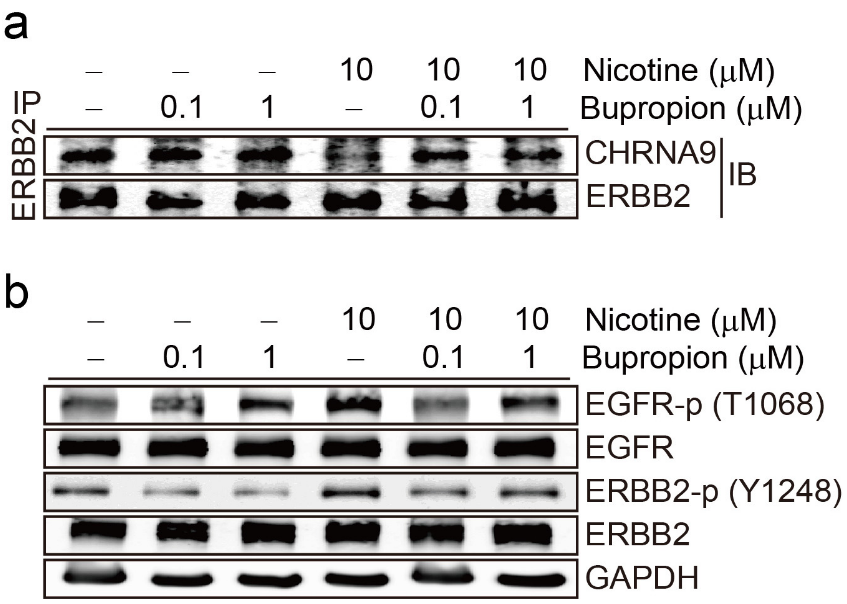

Supplementary Figure 37. Nicotine-induced ERBB2 phosphorylation attenuation and migration/invasion inhibition effects of bupropion on A549 cells.

(a) As an agent, bupropion attenuates nicotine-induced CHRNA9/ERBB2 dissociation, as detected by an IP assay. A549 cells exposed to 10 μM nicotine or a control were subjected to bupropion pretreatment at 0, 0.1 and 1 μM. An ERBB2 antibody was precipitated and immunoblotted for a CHRNA9 antibody, and ERBB2 expression was used as the loading control. When tested *in vitro*, bupropion pretreatment dramatically inhibited the dissociation of the CHRNA9/ERBB2 complex with or without nicotine dose-dependent treatment in lung cancer cells (A549) as determined by IP analysis **(b)** A549 cells were treated with 10 μM nicotine with or without 0.1 and 1 μM bupropion, and pretreatment with bupropion could inhibit nicotine-induced ERBB2 (Y1248) and EGFR (T1068) activation. GAPDH served as the internal control. Bupropion also significantly attenuated nicotine-induced EGFR and ERBB2 phosphorylation in A549 cells, indicating that bupropion may be able to prevent nicotine-induced carcinogenesis in lung cancer.

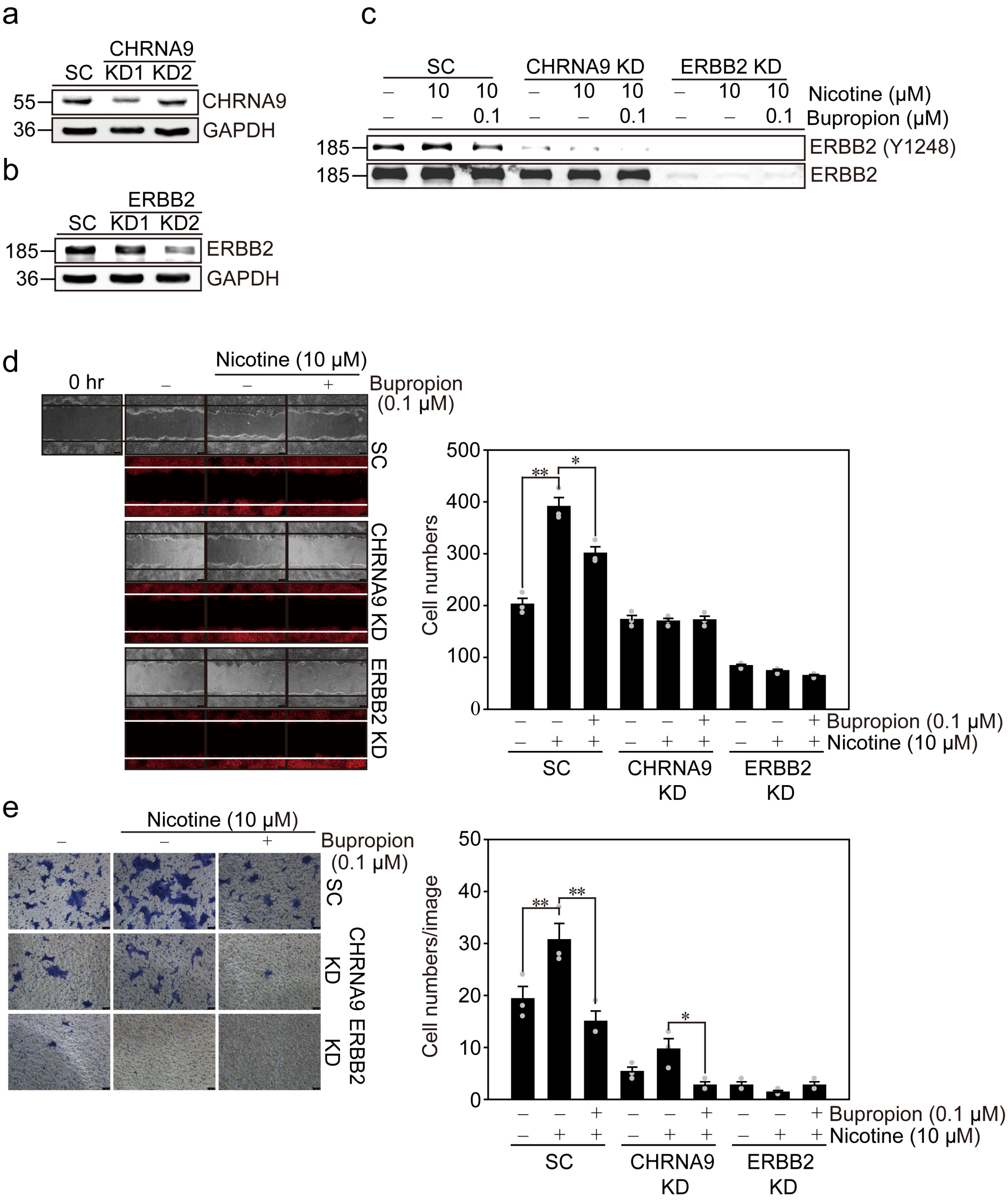

Supplementary Figure 38. Nicotine-induced cancer cell signal/migration/invasion inhibition effects of bupropion on scramble BT474 cells and CHRNA9 (or ERBB2) knockdown BT474 cells.

BT474 cells were transfected with scramble (SC), CHRNA9 KD1, CHRNA9 KD2, ERBB2 KD1 and ERBB2 KD2 CRISPR/Cas9 virus for 48 hours and puromycin-selected for another 48 hours. The cells

were immunoblotted with (**a**) CHRNA9 and (**b**) ERBB2 antibodies to determine the protein knockdown efficiency. GAPDH served as the internal control. (**c**) ERBB2 phosphorylation in SC, CHRNA9 KD1 and ERBB2 KD2 BT474 cells was assessed after bupropion pretreatment at 0.1 μM with or without 10 μM nicotine. Total ERBB2 served as the internal control. The BT474 cells were next assessed for their (**d**) migration and (**e**) invasion abilities at 24 hours and 72 hours after bupropion treatment at 0.1 and 1 μM with or without 10 μM nicotine. The numbers of migrated and invaded cells were calculated using ImageJ software, and the experiments were repeated three times. The error bars indicate the mean ± standard error. Data were analyzed with Student's t-test; all *P* values are two-sided. *P* values less than 0.05 are indicated with an asterisk, and values less than 0.01 are indicated with two asterisks.

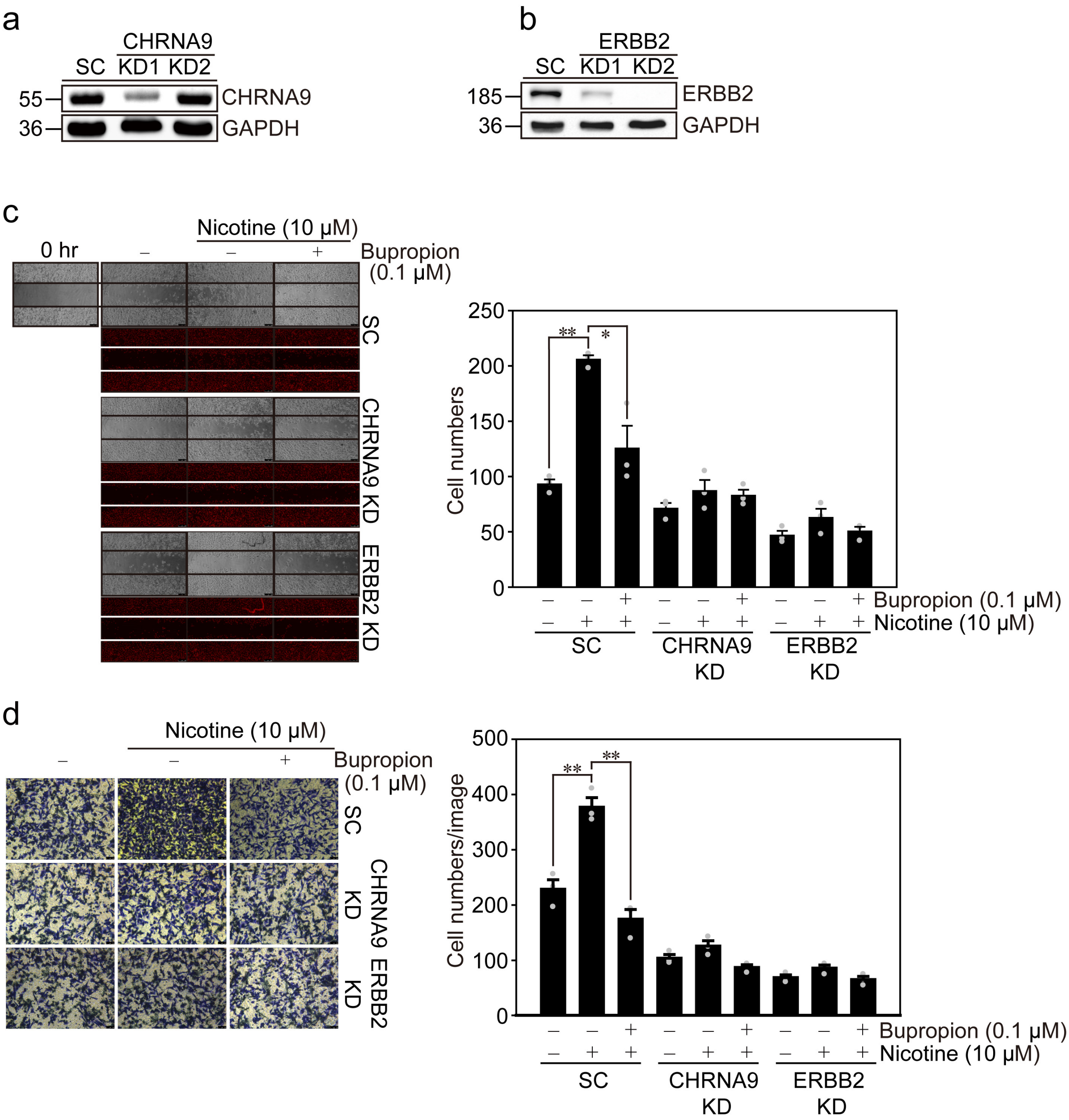

Supplementary Figure 39. Nicotine-induced cancer cell migration/invasion inhibition effects of bupropion on scramble MDA-MB-231 cells and CHRNA9 (or ERBB2) knockdown MDA-MB-231 cells.

MDA-MB-231 cells were transfected with scramble (SC), CHRNA9 KD1, CHRNA9 KD2, ERBB2 KD1 and ERBB2 KD2 CRISPR/Cas9 virus for 48 hours and puromycin-selected for another 48 hours. The cells were immunoblotted with (**a**) CHRNA9 and (**b**) ERBB2 antibodies to determine the protein

knockdown efficiency. GAPDH served as the internal control. The SC, CHRNA9 KD1 and ERBB2 KD2 MDA-MB-231 cells were assessed for their (**c**) migration and (**d**) invasion abilities at 12 hours and 48 hours after bupropion treatment at 0.1 and 1 μM with or without 10 μM nicotine. The numbers of migrated and invaded cells were calculated using ImageJ software, and the experiments were repeated three times. The error bars indicate the mean ± standard error. Data were analyzed with Student's t-test; all *P* values are two-sided. *P* values less than 0.05 are indicated with an asterisk, and values less than 0.01 are indicated with two asterisks.

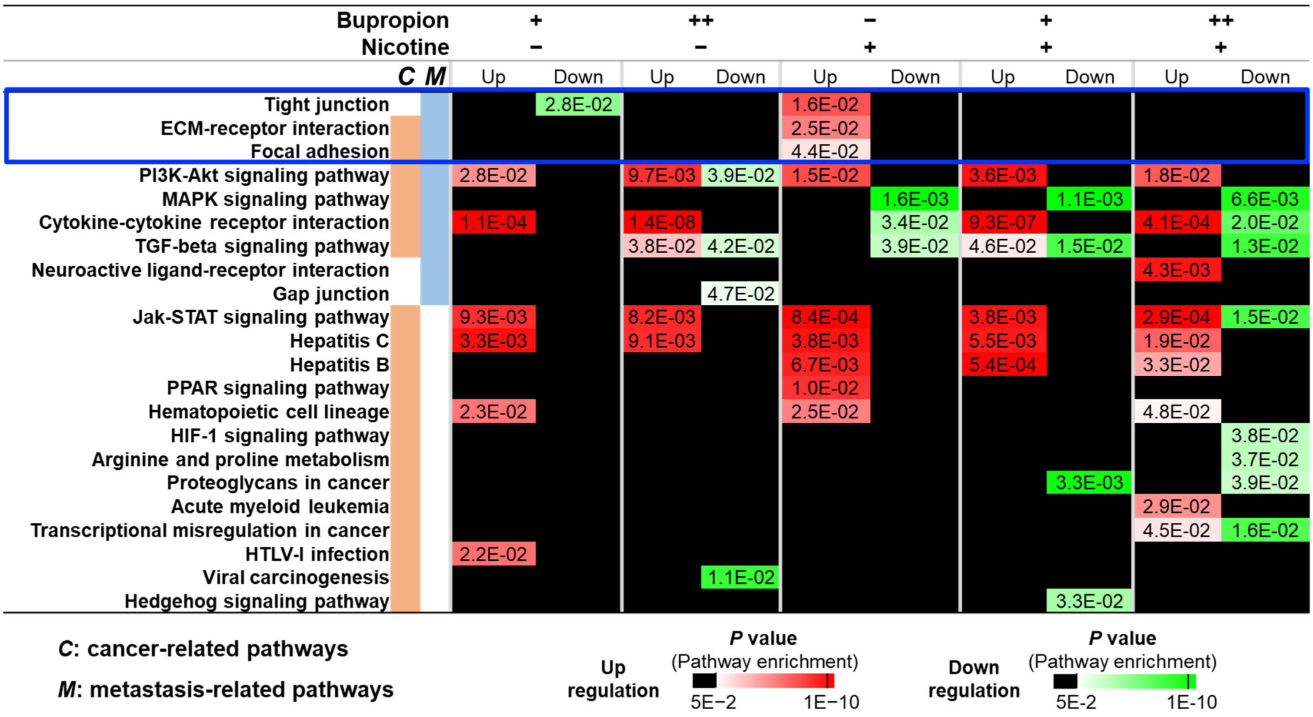

Supplementary Figure 40. Enrichment of significantly up- and down-regulated genes involved in cancer- and metastasis-related pathways in xenograft mice subjected to bupropion and nicotine treatment. Significantly up- or down-regulated genes were defined as those with at least a 2-fold expression change in the mammary tumor tissues of xenograft mice. The control (no treatment) was compared with different treatment combinations of bupropion and nicotine, including the control versus 100 μg kg^{-1} bupropion (+), the control versus 200 μg kg^{-1} bupropion (++), the control versus nicotine exposure (+), the control versus 100 μg kg^{-1} bupropion with nicotine exposure, and the control versus 200 μg kg^{-1} bupropion with nicotine exposure, shown from left to right. Nicotine was added to the drinking water at 10 μg ml^{-1}. Pathway enrichments of each up- (red) and down-regulated (green) gene set were considered statistically significant at *P* values less than 0.05, as determined using a hypergeometric test.

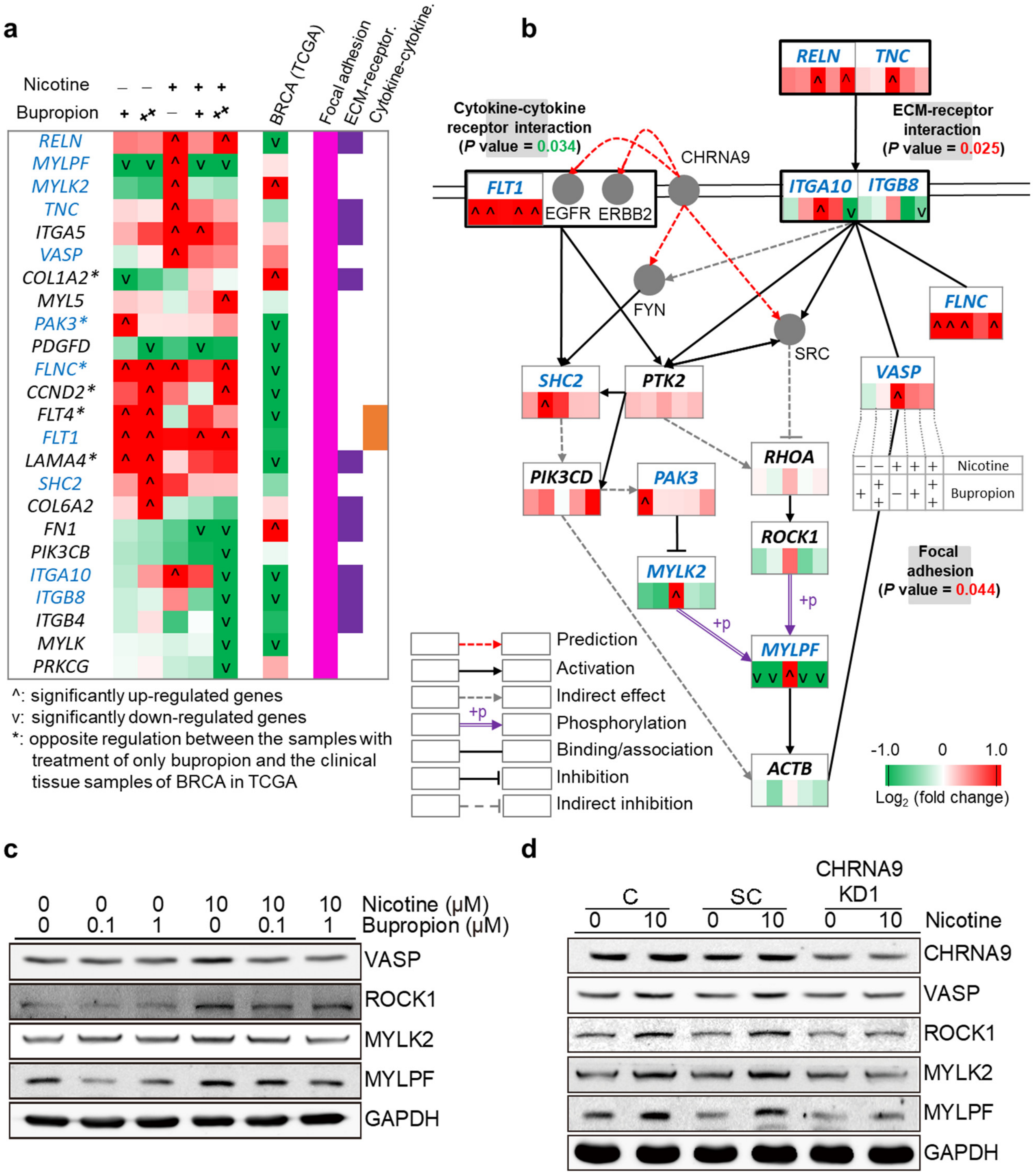

Supplementary Figure 41. Analysis of the mechanisms of anti-metastasis on bupropion. (a) Differential expression profiles of several metastasis-related pathways, including the focal adhesion, ECM-receptor interaction, and cytokine-cytokine receptor interaction pathways, comprising 24 genes with significant up- or down-regulation in at least one test condition for mice with or without bupropion treatment (+ refers to 100 μg kg^{-1}; ++ refers to 200 μg kg^{-1}) and/or nicotine exposure. For each gene,

the fold change values are listed in order as (from left to right) the control (no treatment) versus 100 μg kg^{-1} bupropion, 200 μg kg^{-1} bupropion, nicotine exposure, 100 μg kg^{-1} bupropion with nicotine exposure, and 200 μg kg^{-1} bupropion with nicotine exposure. Nicotine was added to the drinking water at 10 μg ml^{-1}. The fold changes of these genes in TCGA BRCA RNA-seq data are shown. Among these 24 genes, six genes (with an asterisk), including *COL1A2*, *LAMA4*, and *FLT4*, were oppositely regulated between the experimental treatment data with only bupropion and the BRCA clinical tissue sample data from TCGA. The fold change levels of these genes are demonstrated by the color scheme (red: up-regulated; green: down-regulated; white: no change). Pathway enrichment was considered statistically significant at *P* values less than 0.05, as determined using a hypergeometric test. (**b**) Proposed model for regulation of the cell motility and cellular community in the focal adhesion pathway through the inhibition and activation of CHRNA9 by bupropion and nicotine, respectively. Genes with differential expression in at least one test condition are colored blue. (**c**) Bupropion inhibiting the expression of nicotine-induced focal adhesion-related proteins. Serum-starved MDA-MB-231 cells were treated with bupropion dose-dependently with or without nicotine for 24 hours, and bupropion was added 30 minutes prior to nicotine exposure. Proteins, including VASP, ROCK1, MYLK2, and MYLPF, were analyzed by immunoblotting. (**d**) Nicotine blockade by CHRNA9 gene editing in MDA-MB-231 cells mediates the focal adhesion pathway. CRISPR/Cas9 mediates scrambled (SC) and CHRNA9-targeted (CHRNA9 KD1) viruses were introduced into MDA-MB-231 cells for 72 hours and puromycin-selected for 48 hours. Parental control (C), SC, and CHRNA9 KD1 cells were treated with or without nicotine for 24 hours, and focal adhesion-related protein expression was measured via SDS-PAGE. GAPDH served as the internal control.

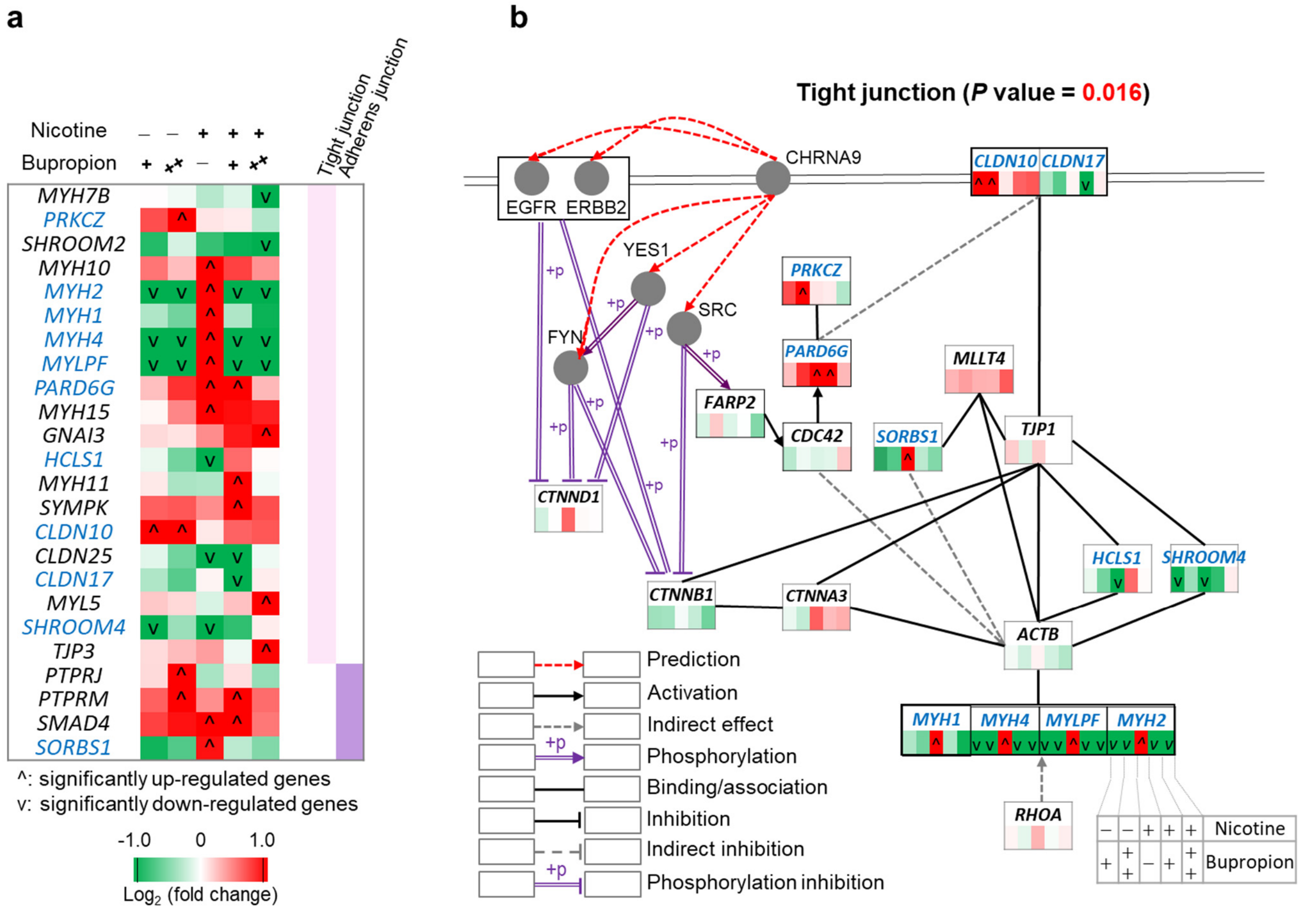

Supplementary Figure 42. Heat map of differentially expressed genes and a proposed model for bupropion as a CHRNA9 inhibitor against nicotine in the tight junction and adherens junction pathways.

(**a**) Differential expression profiles of several metastasis-related pathways, including the tight junction, and adherens junction pathways, containing 24 genes with significant up- or down-regulation in at least one test condition for mice with or without bupropion treatment (+ refers to 100 μg kg^{-1}; ++ refers to 200 μg kg^{-1}) and/or nicotine exposure. The fold change levels of these genes are displayed in the color scheme (red: up-regulated; green: down-regulated; white: no change). (**b**) Proposed model for regulation of the cellular community in the tight junction and adherens junction pathways through the inhibition and activation of CHRNA9 by bupropion and nicotine, respectively. For each gene, the fold change values are listed in order (from left to right) as 100 μg kg^{-1} bupropion, 200 μg kg^{-1} bupropion, nicotine exposure, 100 μg kg^{-1} bupropion with nicotine exposure and 200 μg kg^{-1}

bupropion with nicotine exposure. Nicotine was added to the drinking water at 10 μg ml^{-1}. Pathway enrichment was considered statistically significant at *P* values less than 0.05, as determined using a hypergeometric test.

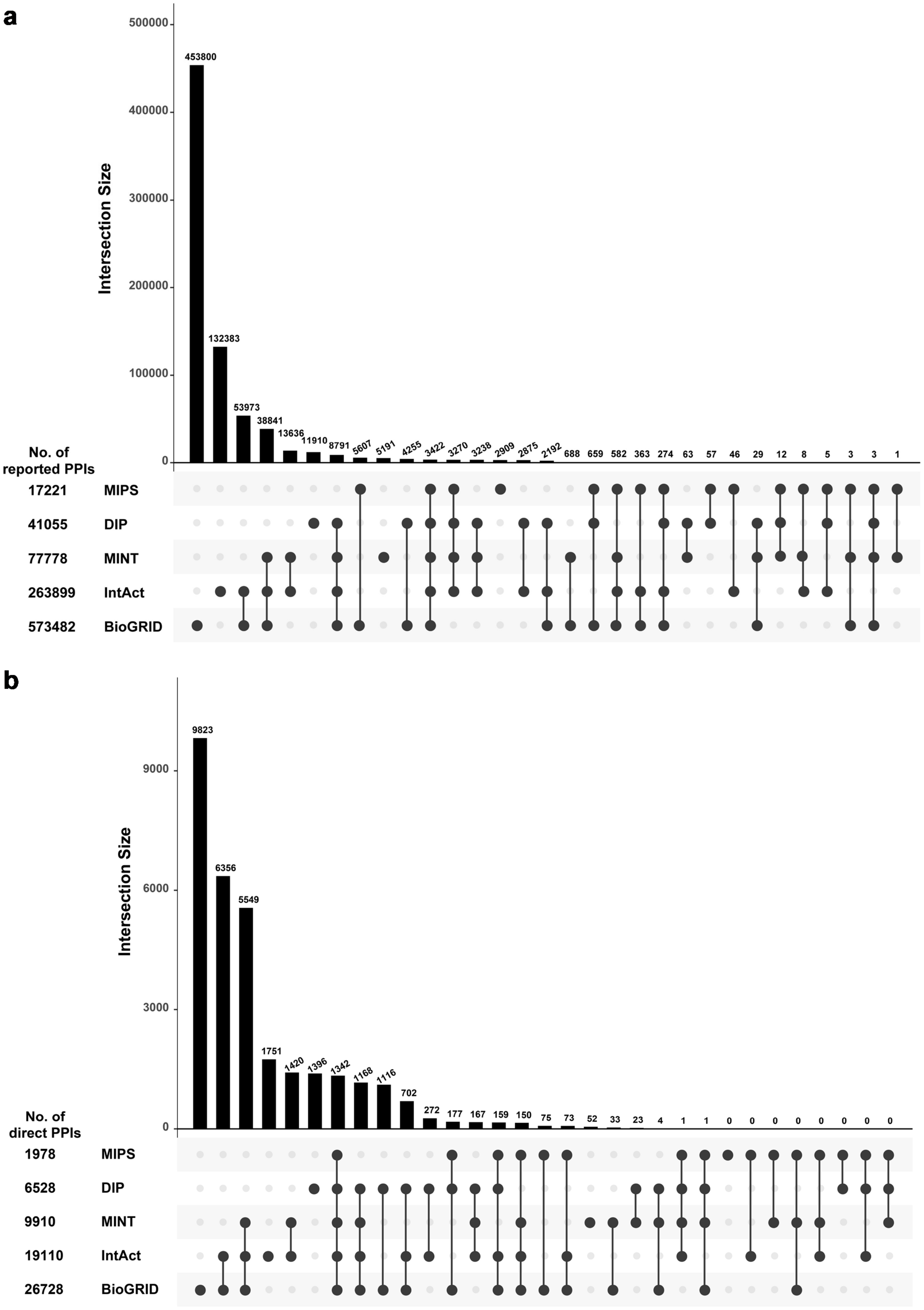
a
Intersection Size
500000
400000
300000
200000
100000
0
453800
132383
53973
38841
13636
11910
8791
5607
5191
4255
3422
3270
3238
2909
2875
2192
688
659
582
363
274
63
57
46
29
12
8
5
3
3
1
No. of reported PPIs
17221 MIPS
41055 DIP
77778 MINT
263899 IntAct
573482 BioGRID
b
Intersection Size
9000
6000
3000
0
9823
6356
5549
1751
1420
1396
1342
1168
1116
702
272
177
167
159
150
75
73
52
33
23
4
1
1
0
0
0
0
0
0
0
0
No. of direct PPIs
1978 MIPS
6528 DIP
9910 MINT
19110 IntAct
26728 BioGRID

Supplementary Figure 43. Matrix layout for all intersections of (a) reported PPI and (b) direct PPI data in five public databases (i.e., BioGRID, IntAct, MINT, DIP, and MIPS), sorted by size (i.e., PPI number).

The dark circles in the matrix indicate the sets that are part of the intersection. In five public PPI databases, most of the reported PPIs (97%, 728,943/749,087) were assembled from the BioGRID and IntAct databases, and only 3,422 (0.5%) PPIs were recorded in all five databases. Similarly, we also observed that 95% (30,339) of 31,810 direct physical interactions were collected from the BioGRID and IntAct databases, and the overlap of all databases was 4.2% (1,342).

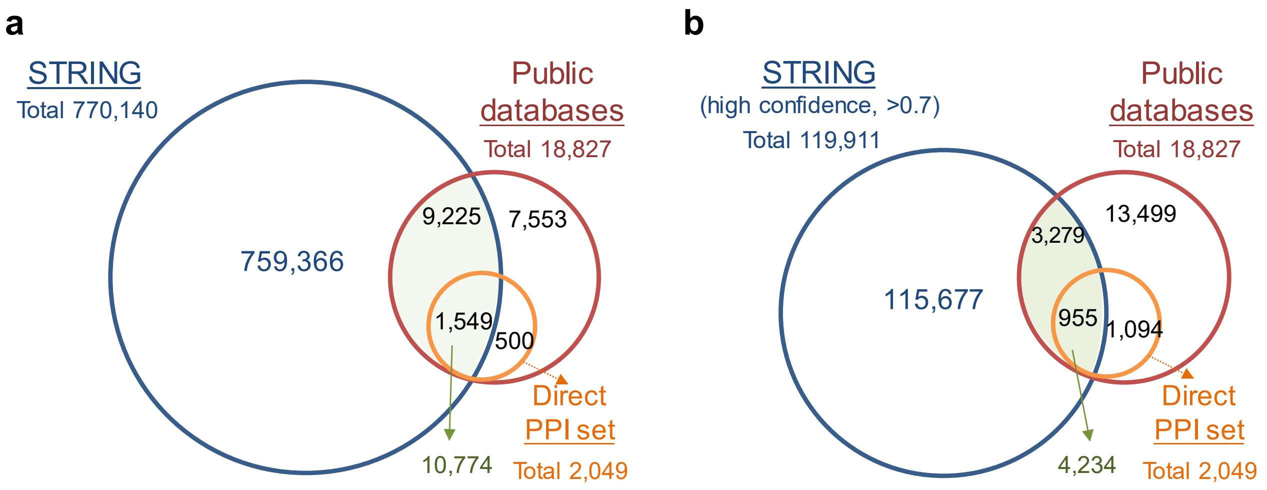

Supplementary Figure 44. The intersections between (a) all PPI and (b) high confidence PPI data in the STRING database (blue circle) and all reported PPI (red circle) and direct PPI (orange circle) data in five reference databases.

STRING human protein network data (9606.protein.links.v10.txt) were downloaded from the STRING database[1] (https://string-db.org/cgi/download.pl). Among 770,140 human MP PPIs in STRING, 119,911 high-confidence PPIs were selected using the suggested threshold (>0.7; https://string-db.org/cgi/help.pl).

Supplementary Table 1. KEGG cancer-related pathways used in our analysis

No.	KEGG cancer-related pathway	No.	KEGG cancer-related pathway
1	hsa00010: Glycolysis / Gluconeogenesis	34	hsa04520: Adherens junction
2	hsa00020: Citrate cycle (TCA cycle)	35	hsa04630: Jak-STAT signaling pathway
3	hsa00030: Pentose phosphate pathway	36	hsa04640: Hematopoietic cell lineage
4	hsa00190: Oxidative phosphorylation	37	hsa04662: B cell receptor signaling pathway
5	hsa00250: Alanine, aspartate and glutamate metabolism	38	hsa04810: Regulation of actin cytoskeleton
6	hsa00260: Glycine, serine and threonine metabolism	39	hsa04916: Melanogenesis
7	hsa00330: Arginine and proline metabolism	40	hsa05160: Hepatitis C
8	hsa00471: D-Glutamine and D-glutamate metabolism	41	hsa05161: Hepatitis B
9	hsa00564: Glycerophospholipid metabolism	42	hsa05166: HTLV-I infection
10	hsa00980: Metabolism of xenobiotics by cytochrome P450	43	hsa05169: Epstein-Barr virus infection
11	hsa03022: Basal transcription factors	44	hsa05200: Pathways in cancer
12	hsa03320: PPAR signaling pathway	45	hsa05202: Transcriptional misregulation in cancer
13	hsa04010: MAPK signaling pathway	46	hsa05203: Viral carcinogenesis
14	hsa04012: ErbB signaling pathway	47	hsa05204: Chemical carcinogenesis
15	hsa04020: Calcium signaling pathway	48	hsa05205: Proteoglycans in cancer
16	hsa04024: cAMP signaling pathway	49	hsa05206: MicroRNAs in cancer
17	hsa04060: Cytokine-cytokine receptor interaction	50	hsa05210: Colorectal cancer
18	hsa04062: Chemokine signaling pathway	51	hsa05211: Renal cell carcinoma
19	hsa04064: NF-kappa B signaling pathway	52	hsa05212: Pancreatic cancer
20	hsa04066: HIF-1 signaling pathway	53	hsa05213: Endometrial cancer
21	hsa04110: Cell cycle	54	hsa05214: Glioma
22	hsa04115: p53 signaling pathway	55	hsa05215: Prostate cancer
23	hsa04120: Ubiquitin mediated proteolysis	56	hsa05216: Thyroid cancer
24	hsa04142: Lysosome	57	hsa05217: Basal cell carcinoma
25	hsa04150: mTOR signaling pathway	58	hsa05218: Melanoma
26	hsa04151: PI3K-Akt signaling pathway	59	hsa05219: Bladder cancer
27	hsa04210: Apoptosis	60	hsa05220: Chronic myeloid leukemia
28	hsa04310: Wnt signaling pathway	61	hsa05221: Acute myeloid leukemia
29	hsa04340: Hedgehog signaling pathway	62	hsa05222: Small cell lung cancer
30	hsa04350: TGF-beta signaling pathway	63	hsa05223: Non-small cell lung cancer
31	hsa04370: VEGF signaling pathway	64	hsa05230: Central carbon metabolism in cancer
32	hsa04510: Focal adhesion	65	hsa05231: Choline metabolism in cancer
33	hsa04512: ECM-receptor interaction		

Supplementary Table 2. Expression datasets of RNA-seq and clinical outcome data in 15 cancer types assembled from TCGA used in our analysis

Cancer types[#]	Abbreviated name	Number of normal samples	Number of tumor samples (Number of patients used in survival analysis)
Bladder urothelial carcinoma	BLCA	19	408 (403)
Breast invasive carcinoma	BRCA	113	1102 (1092)
Cholangiocarcinoma	CHOL	9	36 (36)
Colon adenocarcinoma	COAD	41	287 (282)
Head and neck squamous cell carcinoma	HNSC	44	522 (518)
Kidney chromophobe	KICH	25	66 (64)
Kidney renal clear cell carcinoma	KIRC	72	534 (529)
Kidney renal papillary cell carcinoma	KIRP	32	291 (287)
Liver hepatocellular carcinoma	LIHC	50	374 (369)
Lung adenocarcinoma	LUAD	59	517 (495)
Lung squamous cell carcinoma	LUSC	51	502 (486)
Prostate adenocarcinoma	PRAD	52	498 (497)
Rectum adenocarcinoma	READ	10	95 (93)
Thyroid carcinoma	THCA	59	513 (504)
Uterine corpus endometrial carcinoma	UCEC	24	177 (174)

[#]Extending the first 12 tumor types profiled by the TCGA Pan-Cancer project, we selected a total of 15 cancer types in this study based on the following criteria: (1) for each selected cancer type, the numbers of both the tumor and the corresponding normal samples were at least five (e.g., excluding ovarian carcinoma and lymphoblastic acute myeloid leukemia that have no normal samples); (2) the cancer types from the same tissues or organs complying with criterion 1 were included (e.g., kidney chromophobe, kidney renal clear cell carcinoma, and kidney renal papillary cell carcinoma); (3) glioblastoma multiforme was excluded due to only containing an organ-specific control, that is, the normal tissue data from participants who did not have cancer.

Supplementary Table 3. The feature summary of four PPI predicted approaches

Methods	Features										
	**Quality of reported PPI**	**Sequence similarity**	**Evolutionary analysis**	**Network topology**	**Domains**	**Gene co-expression**	**Text mining**	**Genomic context associations**	**Post-translational modification (PTM)**	**Physicochemical properties**	**Pathway and functional annotations**
SIM	Considering the quality of reported/direct PPIs (S_{qul}) for selecting PPI templates using	1. Sequence similarity (S_{jss}) 2. Normalized ranking of sequence similarity (S_{rank})	Evaluated the evolutionary distance across multiple species (S_{es})	Topology of the PPI network in humans (S_{topo})	Using the topological domain annotations from UniProt to search PPI templates (S_{irs})	N/A	N/A	N/A	N/A	N/A	N/A
Generalized Interolog mapping	User-dependent selection; for example, we used reported PPIs and direct PPIs as the templates	Sequence similarity	N/A	N/A	N/A	N/A	N/A	N/A	N/A	N/A	N/A
STRING	Considering the quality of reported PPIs	N/A	N/A	N/A	N/A	Gene co-expression information was based on gene expression datasets from the GEO	Co-mentioned in PubMed Abstracts	1. Considering neighborhood of gene pairs in the genome 2. Considering gene fusion events 3. Considering gene co-occurence across genomes	N/A	N/A	Protein pairs were recorded in pathway databases
FpClass	N/A	Sequence similarity	Known orthologous interactions in model organisms and paralogous interactions in humans	Topology of the PPI network in humans	Using the domain annotations of human proteins from InterPro and UniProt as the feature sets to compute interaction scores	Gene co-expression information was based on gene expression datasets from the GEO	N/A	N/A	Using the PTMs (e.g., methylation, phosphorylation) of human proteins as the feature sets to compute interaction scores	Structural-chemical features from protein sequence were used to predict the fraction of a protein's residues in disordered regions, and chemical features (charge and isoelectric point)	Using the Gene Ontology (GO) terms (BP, CC and MF) as the feature sets to compute interaction scores

Supplementary Table 4. Comparisons of the performances of four approaches using the standard positive (derived from IntAct, BioGRID, DIP, MIPS, and MINT) and negative (RSS_{BP} < 0.4 or RSS_{CC} < 0.4) sets

Methods (suggested threshold)	Positive set (i.e., reported/direct PPIs)	AUC[a]	Numbers of predicted PPIs[b]	F2 score[c]					
				Overall	Enzymes (89 MPs)	Receptors (1,073 MPs)	Miscellaneous (296 MPs)	Transporters (450 MPs)	Unclassified MPs (686 MPs)
Reported PPI-based SIM ($S_{SIM} \geq 3.6$)	MP reported PPIs	0.924	64,132[d]	0.619	0.711	0.519	0.612	0.539	0.751
direct PPI-based SIM ($S_{SIM} \geq 3.7$)	MP direct PPIs	0.932	14,027[d]	0.530	0.714	0.512	0.364	0.640	0.576
FpClass (probability > 0.47)	MP reported PPIs	0.811	43,417	0.412[e]	0.159	0.483	0.374	0.437	0.334
	MP direct PPIs	0.929		0.534[e]	0.276	0.569	0.298	0.524	0.544
STRING (high confidence with scores > 0.7)	MP reported PPIs	0.824	119,911	0.392[e]	0.218	0.431	0.420	0.413	0.430
	MP direct PPIs	0.921		0.342[e]	0.141	0.303	0.470	0.533	0.459
Reported PPI-based Generalized Interologs (Joint E value < 10^{-70})	MP reported PPIs	0.793	3,157,311[d]	0.076[e]	0.072	0.117	0.032	0.060	0.076
Direct PPI-based Generalized Interologs (Joint E value < 10^{-70})	MP direct PPIs	0.835	480,467[d]	0.060[e]	0.035	0.087	0.022	0.032	0.080

[a] Average area under the receiver operating characteristic curve (AUC).

[b] Numbers of predicted PPIs were counted at the suggested threshold for each method.

[c] F_2 scores were calculated for the predicted PPIs at the suggested threshold for each method using sets of positive and negative cases.

[d] For our SIM and generalized interologs, all reported/direct PPIs of each MP were excluded in advance of the PPI templates being selected to avoid bias in evaluating the predictive power of these methods.

[e] Based on the positive sets of MP reported PPIs and MP direct PPIs, the FpClass method achieved the highest F_2 scores of 0.420 and 0.593 when the probabilities were set to 0.32 and 0.71, respectively; STRING achieved the highest F_2 scores of 0.623 and 0.430 when the scores were set to 0.45 and 0.95, respectively; and the reported PPI- and direct PPI-based generalized interologs achieved the highest F_2 scores of 0.177 and 0.200 when the joint *E* values were set to 10^{-150} and 10^{-140}, respectively.

Supplementary Table 5. Expression data sets of microarray data as validation in 15 cancer types assembled from the GEO database

Cancer types	NCBI GEO Accession Number	Number of normal samples	Number of tumor samples	Platform (GeneChip)	References
BLCA	GSE42089	8	10	HG-U133 Plus 2	Zhou *et al.*[76]
BRCA	GSE61304	4	58	HG-U133 Plus 2	Grinchuk *et al.*[77]
BRCA	GSE22544	4	14	HG-U133 Plus 2	Hawthorn *et al.*[78]
CHOL	GSE45001	10	10	Agilent-028004	Sulpice *et al.*[79]
COAD	GSE23878	24	35	HG-U133 Plus 2	Uddin *et al.*[80]
HNSC	GSE9844	12	26	HG-U133 Plus 2	Ye *et al.*[81]
KICH	GSE26574	8	10	HG-U133 Plus 2	Ooi *et al.*[82]
KIRC	GSE36895	23	29	HG-U133 Plus 2	Peña-Llopis *et al.*[83]
KIRC	GSE15641	23	32	HG-U133A	Jones *et al.*[84]
KIRP	GSE7023	12	35	HG-U133 Plus 2	Furge *et al.*[85]
LIHC	GSE19665	10	10	HG-U133 Plus 2	Deng *et al.*[86]
LIHC	GSE29721	10	10	HG-U133 Plus 2	Stefanska *et al.*[87]
LUAD	GSE19188	15	18	HG-U133 Plus 2	Hou *et al.*[88]
LUAD	GSE31547	20	30	HG-U133A	Girard (unpublished)
LUSC	GSE30219	14	61	HG-U133 Plus 2	Rousseaux *et al.*[89]
LUSC	GSE19188	16	16	HG-U133 Plus 2	Hou *et al.*[88]
PRAD	GSE82132	50	38	HG-U95Av2	Stuart *et al.*[90]
READ	GSE20842	65	65	Agilent-014850	Gaedcke *et al.*[91]
THCA	GSE60542	30	33	HG-U133 Plus 2	Tarabichi *et al.*[92]
UCEC	GSE17025	12	91	HG-U133 Plus 2	Day *et al.*[93]

Supplementary Table 6. The summary table of IP and FRET results on multiple cancer cell lines

18 selected partner candidates of CHRNA9[#]	BT474		MDA-MB-231		A549	RT4	MIA PaCa-2
	IP	FRET	IP	FRET	IP	IP	IP
SRC	+	+	+	+	+	+	+
SFN	+	+	+	+	+	+	+
EGFR	+	+	+	+	+	+	+
ERBB2	+	+	+	-	+	+	+
ERBB3	+	+	+	+	+	+	+
COPS6	+	+	+	+	+	+	+
CSNK1D	+	+	-	+	+	+	-
YWHAG	+	+	+	+	+	+	+
YWHAB	-	+	+	+	-	+	-
YWHAH	+	+	+	+	+	+	+
INSR	+	+	+	+	+	+	+
APP	+	+	+	+	-	+	-
FYN	-	-	-	-	-	+	-
PLK1	+	-	+	+	+	+	-
HCK	+	+	+	+	+	-	+
ERBB4	+	+	-	-	-	-	+
ATXN1	+	-	+	-	+	+	+
ABCB1	-	-	+	-	-	-	-

[#]This table consists of the results of IP data (**Supplementary Fig. 30**) on five (BT474, MDA-MB-231, A549, RT4 and MIA PaCa-2) cancer cells and FRET data (**Supplementary Fig. 31**) on two (BT474 and MDA-MB-231) breast cancer cells. A candidate that passes the threshold (>3%) of both reciprocal IP assays is considered as a positive interaction.

Supplementary Table 7. Antibodies used in our analysis

Antibody	Purchase company	Catalog	Assay[e]
ERBB2	GeneTex	GTX117479	IP[a]
	Cell Signaling Technology	#4290	Western blotting
	Santa Cruz Biotechnology	sc-33684	FRET
SFN	Abcam	ab14123	IP[a] & Western blotting
	R&D system	AF4424	FRET
SRC	Abcam	ab16885	IP[a] & Western blotting
	R&D system	MAB3389	FRET
ERBB4	Abcam	ab32375	IP[a] & Western blotting
	R&D system	MAB1131	FRET
INSR	Cell Signaling Technology	#3025	IP[a] & Western blotting
	Santa Cruz Biotechnology	sc-390130	FRET
EGFR	Cell Signaling Technology	#4267	IP[a]
	Abcam	ab52894	Western blotting
	R&D system	AF231	FRET
FYN	GeneTex	GTX21881	IP[a] & Western blotting
	R&D system	MAB3574	FRET
YWHAB	Abcam	ab32560	IP[a] & Western blotting
	R&D system	AF4724	FRET
GAPDH	Santa Cruz Biotechnology	sc-32233	Western blotting
CHRNA9	Abcam	ab49065	IP[b] & Western blotting
	Thermo Fisher Scientific	PA5-46826	FRET<br>IP[b] & Western blotting
ERBB2 (Y1248)	Cell Signaling Technology	#2247	IP[a]& Western blotting
EGFR (T1068)	Cell Signaling Technology	#3777	Western blotting
VASP	Abcam	ab109321	Western blotting
MYLK2	Thermo Fisher Scientific	PA5-29324	Western blotting
MYLPF	Abcam	ab135404	Western blotting
ROCK1	Cell Signaling Technology	#4035	Western blotting
YWHAG	GeneTex	GTX113298	IP[d] & Western blotting
	R&D system	MAB5700	FRET

ERBB3	Novus	NBP2-32253	IP[a] & Western blotting
	R&D system	AF234	FRET
PLK1	Novus	NB100-547	IP[d] & Western blotting
	R&D system	AF3804	FRET
CSNK1D	Novus	NPB1-21376	IP[d] & Western blotting
	GeneTex	GTX123435	FRET
HCK	Abnova	H00003055-D01P	IP[d] & Western blotting
	GeneTex	GTX32645	FRET
APP	Thermo Fisher Scientific	OMA1-03132	IP[a] & Western blotting
	GeneTex	GTX84875	FRET
YWHAH	Abcam	ab206292	IP[d] & Western blotting
	R&D system	AF4420	FRET
ATXN1	Abcam	ab201037	IP[c] & Western blotting
	GeneTex	GTX80399	FRET
ABCB1	Abcam	ab129450	IP[c] & Western blotting
	GeneTex	GTX42220	FRET
COPS6	Santa Cruz Biotechnology	sc-393023	IP[d] & Western blotting
	GeneTex	GTX115519	FRET
SLC16A7	Abcam	Ab224627	IP[b] & Western blotting
CHK1	Abcam	Ab69536	IP[b] & Western blotting
CDK1	Abcam	Ab18	IP[b] & Western blotting
MELK	Abcam	Ab129373	IP[b] & Western blotting
Mouse Anti-rabbit IgG (Light-Chain Specific-L57A3)	Cell Signaling Technology	#3677	IP
Goat anti-mouse-IgG (H+L)	Santa Cruz Biotechnology	SC-2005	Western blotting
Goat anti-rabbit-IgG (H+L)	Santa Cruz Biotechnology	SC-2004	Western blotting
FITC	Jackson Immuno Research	115-095-003	FRET
Rhodamine Red	Jackson Immuno Research	111-295-003	FRET
Rabbit IgG	Abcam	Ab172730	IP

[a] 200 μg of cell extract was incubated with 1 μg antibody in IP experiment.

[b] 200 μg of cell extract was incubated with 3 μg antibody in IP experiment.

[c] 200 μg of cell extract was incubated with 6 μg antibody in IP experiment.

[d] 400 μg of cell extract was incubated with 12 μg antibody in IP experiment.

[e] For western blot analysis, all primary antibodies were diluted 1000x, whereas all secondary antibodies were diluted 4000x. For FRET analysis, all primary antibodies were diluted 100x, whereas all secondary antibodies were diluted 50x

Supplementary Table 8. Primer sequence used in our analysis

Name	Primer sequence
HBsAg Q-PCR sense	GCTGCTATGCCTCATCT
HBsAg Q-PCR antisense	GAATACAAGTGCAATTTCCGTC
MYD88 Q-PCR sense	CTGCAGAGCAAGGAATGT
MYD88 Q-PCR antisense	CCAAGATTTGGTGCAGGG
TNF Q-PCR sense	ACTCCCAGGTCCTCTTC
TNF Q-PCR antisense	CTCCCAGATAGATGGGCTCATA
TLR4 Q-PCR sense	CTGCATAAAGTATGGTAGAGGT
TLR4 Q-PCR antisense	CGGGAATAAAGTCTCTGTAGTG
ATF4 Q-PCR sense	GGTGTTCTCTGTGGGTC
ATF4 Q-PCR antisense	TCTTCTTCTGGCGGTACCTA
CXCL8 Q-PCR sense	TAGATGTCAGTGCATAAAGACATAC
CXCL8 Q-PCR antisense	AAACTTCTCCACAACCCT
FASLG Q-PCR sense	GAAGGAGCTGGCAGAAC
FASLG Q-PCR antisense	CAGAGGCATGGACCTTGA
CASP8 Q-PCR sense	TTTGACCACGACCTTTGAAGA
CASP8 Q-PCR antisense	ATCAGTGCCATAGATGATGC
FOS Q-PCR sense	CTCAGTGGAACCTGTCAAG
FOS Q-PCR antisense	GCTGCATAGAAGGACCC
CREB3L2 Q-PCR sense	AGCTTCAGACTTTGGTGAT
CREB3L2 Q-PCR antisense	TTGGTGGCAGAAGGATAGG
CHRNA9 KD1 sense	CACCGTCTGAGAGAGCGTAATCTGC
CHRNA9 KD1 antisense	AAACGCAGATTACGCTCTCTCAGAC
CHRNA9 KD2 sense	CACCGTTCTAATGCTCTTCGTCCAG
CHRNA9 KD2 antisense	AAACCTGGACGAAGAGCATTAGAAC
ERBB2 KD1 sense	CACCGCTCCATTGTCTAGCACGGCC
ERBB2 KD1 antisense	AAACGGCCGTGCTAGACAATGGAGC
ERBB2 KD2 sense	CACCGCTCCATTGTCTAGCACGGCC
ERBB2 KD2 antisense	AAACGGCCGTGCTAGACAATGGAGC
SLC16A7 KD1 sense	CACCGTGGAGCCAAGAATATAATGG
SLC16A7 KD1 antisense	AAACCCATTATATTCTTGGCTCCAC
SLC16A7 KD2 sense	CACCGGTGGTTTGATTGGGTCCAAG
SLC16A7 KD2 antisense	AAACCTTGGACCCAATCAAACCACC
Mycoplasma sense	TGCACCATCTGTCACTCTGTTAACCTC
Mycoplasma antisense	GGGAGCAAACAGGATTAGATACCCT

Supplementary References

1. Szklarczyk, D., *et al.* The STRING database in 2017: quality-controlled protein-protein association networks, made broadly accessible. *Nucleic Acids Res* **45**, D362-D368 (2017).
2. Kotlyar, M., *et al.* In silico prediction of physical protein interactions and characterization of interactome orphans. *Nat Methods* **12**, 79-84 (2015).
3. Yu, H.Y., *et al.* Annotation transfer between genomes: Protein-protein interologs and protein-DNA regulogs. *Genome Res* **14**, 1107-1118 (2004).
4. Walhout, A.J.M., *et al.* Protein interaction mapping in C-elegans using proteins involved in vulval development. *Science* **287**, 116-122 (2000).
5. Lo, Y.S., Huang, S.H., Luo, Y.C., Lin, C.Y. & Yang, J.M. Reconstructing genome-wide protein-protein interaction networks using multiple strategies with homologous mapping. *PloS one* **10**, e0116347 (2015).
6. Wu, X.M., Zhu, L., Guo, J., Zhang, D.Y. & Lin, K. Prediction of yeast protein-protein interaction network: insights from the Gene Ontology and annotations. *Nucleic Acids Res* **34**, 2137-2150 (2006).
7. Luo, H., Lin, Y., Gao, F., Zhang, C.T. & Zhang, R. DEG 10, an update of the database of essential genes that includes both protein-coding genes and noncoding genomic elements. *Nucleic Acids Res* **42**, D574-D580 (2014).
8. Meyers, R.M., *et al.* Computational correction of copy number effect improves specificity of CRISPR-Cas9 essentiality screens in cancer cells. *Nat Genet* **49**, 1779-1784 (2017).
9. Babur, O., *et al.* Systematic identification of cancer driving signaling pathways based on mutual exclusivity of genomic alterations. *Genome Biology* **16**(2015).
10. Ciriello, G., Cerami, E., Sander, C. & Schultz, N. Mutual exclusivity analysis identifies oncogenic network modules. *Genome Res* **22**, 398-406 (2012).
11. Gao, J.J., *et al.* Integrative Analysis of Complex Cancer Genomics and Clinical Profiles Using the cBioPortal. *Science Signaling* **6**(2013).
12. Cerami, E., *et al.* The cBio Cancer Genomics Portal: An Open Platform for Exploring Multidimensional Cancer Genomics Data. *Cancer Discov* **2**, 401-404 (2012).
13. Beroukhim, R., *et al.* Assessing the significance of chromosomal aberrations in cancer: Methodology and application to glioma. *P Natl Acad Sci USA* **104**, 20007-20012 (2007).
14. Albert, R., Jeong, H. & Barabasi, A.L. Error and attack tolerance of complex networks. *Nature* **406**, 378-382 (2000).
15. Han, J.D.J., *et al.* Evidence for dynamically organized modularity in the yeast protein-protein interaction network. *Nature* **430**, 88-93 (2004).
16. D'Antonio, M. & Ciccarelli, F.D. Modification of gene duplicability during the evolution of protein interaction network. *Plos Comput Biol* **7**, e1002029 (2011).
17. Bertin, N., *et al.* Confirmation of organized modularity in the yeast interactome. *Plos Biol* **5**, 1206-1210 (2007).
18. Zoller, M. CD44: can a cancer-initiating cell profit from an abundantly expressed molecule? *Nat Rev Cancer* **11**, 254-267 (2011).
19. Hanahan, D. & Weinberg, R.A. Hallmarks of cancer: the next generation. *Cell* **144**, 646-674 (2011).
20. Bhatia, S. & Sklar, C. Second cancers in survivors of childhood cancer. *Nat Rev Cancer* **2**, 124-132 (2002).
21. American Cancer Society, http://www.cancer.org/, 2017.
22. Pinero, J., *et al.* DisGeNET: a comprehensive platform integrating information on human disease-associated genes and variants. *Nucleic Acids Res* **45**, D833-D839 (2017).
23. Alarcon, C.R. & Tavazoie, S.F. Endothelial-cell killing promotes metastasis. *Nature* **536**, 154-155 (2016).

24. Pandey, P., *et al.* Amyloid precursor protein and amyloid precursor-like protein 2 in cancer. *Oncotarget* **7**, 19430-19444 (2016).
25. van Roy, F. Beyond E-cadherin: roles of other cadherin superfamily members in cancer. *Nat Rev Cancer* **14**, 121-134 (2014).
26. Wolf, I., *et al.* Klotho: a tumor suppressor and a modulator of the IGF-1 and FGF pathways in human breast cancer. *Oncogene* **27**, 7094-7105 (2008).
27. Law, V., *et al.* DrugBank 4.0: shedding new light on drug metabolism. *Nucleic Acids Res* **42**, D1091-D1097 (2014).
28. Weigelt, B., Peterse, J.L. & van't Veer, L.J. Breast cancer metastasis: markers and models. *Nat Rev Cancer* **5**, 591-602 (2005).
29. Vonderheide, R.H., *et al.* Clinical activity and immune modulation in cancer patients treated with CP-870,893, a novel CD40 agonist monoclonal antibody. *J Clin Oncol* **25**, 876-883 (2007).
30. Pasquale, E.B. Eph-ephrin bidirectional signaling in physiology and disease. *Cell* **133**, 38-52 (2008).
31. Oricchio, E., *et al.* The Eph-receptor A7 is a soluble tumor suppressor for follicular lymphoma. *Cell* **147**, 554-564 (2011).
32. Morice, P., Leary, A., Creutzberg, C., Abu-Rustum, N. & Darai, E. Endometrial cancer. *Lancet* **387**, 1094-1108 (2016).
33. Lee, C.H., *et al.* Overexpression and activation of the alpha 9-nicotinic receptor during tumorigenesis in human breast epithelial cells. *J Natl Cancer I* **102**, 1322-1335 (2010).
34. Matthay, M.A. & Ware, L.B. Can nicotine treat sepsis? *Nat Med* **10**, 1161-1162 (2004).
35. Sopori, M. Effects of cigarette smoke on the immune system. *Nat Rev Immunol* **2**, 372-377 (2002).
36. Swann, J.B., *et al.* Demonstration of inflammation-induced cancer and cancer immunoediting during primary tumorigenesis. *P Natl Acad Sci USA* **105**, 652-656 (2008).
37. Mantovani, A., Allavena, P., Sica, A. & Balkwill, F. Cancer-related inflammation. *Nature* **454**, 436-444 (2008).
38. Szlosarek, P.W. & Balkwill, F.R. Tumour necrosis factor alpha: a potential target for the therapy of solid tumours. *Lancet Oncol* **4**, 565-573 (2003).
39. Zamani, A.G., *et al.* Evaluation of death pathway genes FAS and FASL polymorphisms in chronic HBV infection. *Int J Immunogenet* **40**, 482-487 (2013).
40. Xu, X.J., Huang, P.X., Yang, B.W., Wang, X.D. & Xia, J.L. Roles of CXCL5 on migration and invasion of liver cancer cells. *J Transl Med* **12**(2014).
41. Zhou, J.M., *et al.* The Role of Chemoattractant Receptors in Shaping the Tumor Microenvironment. *Biomed Res Int* (2014).
42. Strieter, R.M. Chemokines: Not just leukocyte chemoattractants in the promotion of cancer. *Nat Immunol* **2**, 285-286 (2001).
43. Ameri, K., *et al.* Circulating tumour cells demonstrate an altered response to hypoxia and an aggressive phenotype. *Brit J Cancer* **102**, 561-569 (2010).
44. Wouters, B.G. & Koritzinsky, M. Hypoxia signalling through mTOR and the unfolded protein response in cancer. *Nat Rev Cancer* **8**, 851-864 (2008).
45. Hu, J.X., *et al.* Structural basis of G protein-coupled receptor-G protein interactions. *Nat Chem Biol* **6**, 541-548 (2010).
46. Friedl, P. & Wolf, K. Tumour-cell invasion and migration: Diversity and escape mechanisms. *Nat Rev Cancer* **3**, 362-374 (2003).
47. Simpson, K.J., *et al.* Identification of genes that regulate epithelial cell migration using an siRNA screening approach. *Nat Cell Biol* **10**, 1027-1038 (2008).
48. Franzetti, G.A., *et al.* Cell-to-cell heterogeneity of EWSR1-FLI1 activity determines proliferation/migration choices in Ewing sarcoma cells. *Oncogene* **36**, 3505-3514 (2017).

49. Han, G., *et al.* Positive regulation of migration and invasion by vasodilator-stimulated phosphoprotein via Rac1 pathway in human breast cancer cells. *Oncol Rep* **20**, 929-939 (2008).
50. Philippar, U., *et al.* A Mena invasion isoform potentiates EGF-induced carcinoma cell invasion and metastasis. *Dev Cell* **15**, 813-828 (2008).
51. Banerjee, A.G., Bhattacharyya, I. & Vishwanatha, J.K. Identification of genes and molecular pathways involved in the progression of premalignant oral epithelia. *Mol Cancer Ther* **4**, 865-875 (2005).
52. Slamon, D.J., *et al.* Use of chemotherapy plus a monoclonal antibody against HER2 for metastatic breast cancer that overexpresses HER2. *New Engl J Med* **344**, 783-792 (2001).
53. Ross, J.S., *et al.* Targeted therapy in breast cancer - The HER-2/neu gene and protein. *Mol Cell Proteomics* **3**, 379-398 (2004).
54. Yamauchi, H., Stearns, V. & Hayes, D.F. When is a tumor marker ready for prime time? A case study of c-erbB-2 as a predictive factor in breast cancer. *J Clin Oncol* **19**, 2334-2356 (2001).
55. Zimmermann, G.R., Lehar, J. & Keith, C.T. Multi-target therapeutics: when the whole is greater than the sum of the parts. *Drug Discov Today* **12**, 34-42 (2007).
56. Wilhelm, S.M., *et al.* Regorafenib (BAY 73-4506): a new oral multikinase inhibitor of angiogenic, stromal and oncogenic receptor tyrosine kinases with potent preclinical antitumor activity. *Int J Cancer* **129**, 245-255 (2011).
57. Chiu, Y.Y., *et al.* Homopharma: a new concept for exploring the molecular binding mechanisms and drug repurposing. *Bmc Genomics* **15 (Suppl 9)**, S8 (2014).
58. West, K.A., *et al.* Rapid Akt activation by nicotine and a tobacco carcinogen modulates the phenotype of normal human airway epithelial cells. *J Clin Invest* **111**, 81-90 (2003).
59. Schuller, H.M. Is cancer triggered by altered signalling of nicotinic acetylcholine receptors? *Nat Rev Cancer* **9**, 195-205 (2009).
60. Wu, C.H., Lee, C.H. & Ho, Y.S. Nicotinic acetylcholine receptor-based blockade: applications of molecular targets for cancer therapy. *Clin Cancer Res* **17**, 3533-3541 (2011).
61. Ellis, M.J., *et al.* Connecting genomic alterations to cancer biology with proteomics: the NCI Clinical Proteomic Tumor Analysis Consortium. *Cancer Discov* **3**, 1108-1112 (2013).
62. Zhang, B., *et al.* Proteogenomic characterization of human colon and rectal cancer. *Nature* **513**, 382-387 (2014).
63. Quail, D.F. & Joyce, J.A. Microenvironmental regulation of tumor progression and metastasis. *Nat Med* **19**, 1423-1437 (2013).
64. Navin, N., *et al.* Tumour evolution inferred by single-cell sequencing. *Nature* **472**, 90-94 (2011).
65. Shapiro, E., Biezuner, T. & Linnarsson, S. Single-cell sequencing-based technologies will revolutionize whole-organism science. *Nat Rev Genet* **14**, 618-630 (2013).
66. Hermjakob, H., *et al.* The HUPOPSI's Molecular Interaction format - a community standard for the representation of protein interaction data. *Nat Biotechnol* **22**, 177-183 (2004).
67. Almen, M.S., Nordstrom, K.J.V., Fredriksson, R. & Schioth, H.B. Mapping the human membrane proteome: a majority of the human membrane proteins can be classified according to function and evolutionary origin. *Bmc Biol* **7**, 50 (2009).
68. Wang, H.Y., *et al.* beta-amyloid(1-42) binds to alpha 7 nicotinic acetylcholine receptor with high affinity - Implications for Alzheimer's disease pathology. *J Biol Chem* **275**, 5626-5632 (2000).
69. Jaldety, Y., *et al.* Sperm Epidermal Growth Factor Receptor (EGFR) Mediates alpha 7 Acetylcholine Receptor (AChR) Activation to Promote Fertilization. *J Biol Chem* **287**, 22328-22340 (2012).
70. Allen, C.M., Ely, C.M., Juaneza, M.A. & Parsons, S.J. Activation of Fyn tyrosine kinase upon secretagogue stimulation of bovine chromaffin cells. *J Neurosci Res* **44**, 421-429 (1996).

71. Swope, S.L. & Huganir, R.L. Binding of the Nicotinic Acetylcholine-Receptor to Sh2 Domains of Fyn and Fyk Protein-Tyrosine Kinases. *J Biol Chem* **269**, 29817-29824 (1994).
72. Kumar, P. & Meizel, S. Nicotinic acetylcholine receptor subunits and associated proteins in human sperm. *J Biol Chem* **280**, 25928-25935 (2005).
73. Charpantier, E., *et al.* alpha 7 neuronal nicotinic acetylcholine receptors are negatively regulated by tyrosine phosphorylation and Src-family kinases. *J Neurosci* **25**, 9836-9849 (2005).
74. Wang, K., *et al.* Regulation of the neuronal nicotinic acetylcholine receptor by Src family tyrosine kinases. *J Biol Chem* **279**, 8779-8786 (2004).
75. Kanehisa, M. & Goto, S. KEGG: Kyoto Encyclopedia of Genes and Genomes. *Nucleic Acids Res* **28**, 27-30 (2000).
76. Zhou, N., *et al.* The investigational Aurora kinase A inhibitor MLN8237 induces defects in cell viability and cell-cycle progression in malignant bladder cancer cells in vitro and in vivo. *Clin Cancer Res* **19**, 1717-1728 (2013).
77. Grinchuk, O.V., *et al.* Sense-antisense gene-pairs in breast cancer and associated pathological pathways. *Oncotarget* **6**, 42197-42221 (2015).
78. Hawthorn, L., Luce, J., Stein, L. & Rothschild, J. Integration of transcript expression, copy number and LOH analysis of infiltrating ductal carcinoma of the breast. *Bmc Cancer* **10**, 460 (2010).
79. Sulpice, L., *et al.* Molecular profiling of stroma identifies osteopontin as an independent predictor of poor prognosis in intrahepatic cholangiocarcinoma. *Hepatology* **58**, 1992-2000 (2013).
80. Uddin, S., *et al.* Genome-wide expression analysis of Middle Eastern colorectal cancer reveals FOXM1 as a novel target for cancer therapy. *Am J Pathol* **178**, 537-547 (2011).
81. Ye, H., *et al.* Transcriptomic dissection of tongue squamous cell carcinoma. *Bmc Genomics* **9**, 69 (2008).
82. Ooi, A., *et al.* An antioxidant response phenotype shared between hereditary and sporadic type 2 papillary renal cell carcinoma. *Cancer Cell* **20**, 511-523 (2011).
83. Pena-LlopiS, S., *et al.* BAP1 loss defines a new class of renal cell carcinoma. *Nat Genet* **44**, 751-U174 (2012).
84. Jones, J., *et al.* Gene signatures of progression and metastasis in renal cell cancer. *Clin Cancer Res* **11**, 5730-5739 (2005).
85. Furge, K.A., *et al.* Detection of DNA copy number changes and oncogenic signaling abnormalities from gene expression data reveals MYC activation in high-grade papillary renal cell carcinoma. *Cancer Res* **67**, 3171-3176 (2007).
86. Deng, Y.B., *et al.* Identification of genes preferentially methylated in hepatitis C virus-related hepatocellular carcinoma. *Cancer Sci* **101**, 1501-1510 (2010).
87. Stefanska, B., *et al.* Definition of the landscape of promoter DNA hypomethylation in liver cancer. *Cancer Res* **71**, 5891-5903 (2011).
88. Hou, J., *et al.* Gene expression-based classification of non-small cell lung carcinomas and survival prediction. *Plos One* **5**, e10312 (2010).
89. Rousseaux, S., *et al.* Ectopic activation of germline and placental genes identifies aggressive metastasis-prone lung cancers. *Sci Transl Med* **5**, 186ra166 (2013).
90. Stuart, R.O., *et al.* In silico dissection of cell-type-associated patterns of gene expression in prostate cancer. *P Natl Acad Sci USA* **101**, 615-620 (2004).
91. Gaedcke, J., *et al.* Mutated KRAS results in overexpression of DUSP4, a MAP-kinase phosphatase, and SMYD3, a histone methyltransferase, in rectal carcinomas. *Gene Chromosome Canc* **49**, 1024-1034 (2010).

92. Tarabichi, M., *et al.* Revisiting the transcriptional analysis of primary tumours and associated nodal metastases with enhanced biological and statistical controls: application to thyroid cancer. *Br J Cancer* **112**, 1665-1674 (2015).
93. Day, R.S., *et al.* Identifier mapping performance for integrating transcriptomics and proteomics experimental results. *Bmc Bioinformatics* **12**, 213 (2011).
