# Supplementary material for: Membrane protein-regulated networks across human cancers
Source: Nat Commun. 2019 Jul 16;10:3131. doi: 10.1038/s41467-019-10920-8 (PMC6635409; doi:10.1038/s41467-019-10920-8)
Supplement: Supplementary file 3 — Description of Additional Supplementary Files [file 41467_2019_10920_MOESM3_ESM.pdf]

Description of Additional Supplementary Files

File Name: Supplementary Data 1
Description: 2,594 membrane proteins used in our analysis.

File Name: Supplementary Data 2
Description: List of reported PPI-based predicted PPIs and reported PPIs of MPs

File Name: Supplementary Data 3
Description: Profiles of the meta-z-scores for reported PPI-based CaMPNets across 15 cancers.

File Name: Supplementary Data 4
Description: Profiles of the meta-z-scores for direct PPI-based CaMPNets across 15 cancers.

File Name: Supplementary Data 5
Description: List of reported PPI-based MPP communities and reported PPI-based MPP community-regulated pathways with prognostic associations in 15 cancer types.

File Name: Supplementary Data 6
Description: List of direct PPI-based MPP communities and direct PPI-based MPP community-regulated pathways with prognostic associations in 15 cancer types.

File Name: Supplementary Data 7
Description: 31,810 direct PPIs and scores for experimental methods used in our analysis.

File Name: Supplementary Data 8
Description: All plasmid sequences used in this study.
