# Supplementary material for: Membrane protein-regulated networks across human cancers
Source: Nat Commun. 2019 Jul 16;10:3131. doi: 10.1038/s41467-019-10920-8 (PMC6635409; doi:10.1038/s41467-019-10920-8)
Supplement: Supplementary file 11 — Supplementary Data 8 [file 41467_2019_10920_MOESM11_ESM.pdf]

Membrane protein-regulated networks across human cancers

Supplementary Data 8 - All plasmid sequences used in this study

PCDNA5 CHRNA9-CFP

PROMOTER

CHRNA9

Linker

CFP

GACGGATCGGGAGATCTCCCGATCCCCTATGGTGCACTCTCAGTACAATCT
GCTCTGATGCCGCATAGTTAAGCCAGTATCTGCTCCCTGCTTGTGTGTTGGA
GGTCGCTGAGTAGTGCGCGAGCAAAATTTAAGCTACAACAAGGCAAGGCT
TGACCGACAATTGCATGAAGAATCTGCTTAGGGTTAGGCGTTTTGCGCTGC
TTCGCGATGTACGGGCCAGATATACGCGTTGACATTGATTATTGACTAGTTAT
TAATAGTAATCAATTACGGGGTCATTAGTTCATAGCCCATATATGGAGTTCCG
CGTTACATAACTTACGGTAAATGGCCCGCCTGGCTGACCGCCCAACGACCC
CCGCCCATTGACGTCAATAATGACGTATGTTCCCATAGTAACGCCAATAGGG
ACTTTCCATTGACGTCAATGGGTGGAGTATTTACGGTAAACTGCCCACTTGG
CAGTACATCAAGTGTATCATATGCCAAGTACGCCCCCTATTGACGTCAATGA
CGGTAAATGGCCCGCCTGGCATTATGCCCAGTACATGACCTTATGGGACTTT
CCTACTTGGCAGTACATCTACGTATTAGTCATCGCTATTACCATGGTGATGCG
GTTTTGGCAGTACATCAATGGGCGTGGATAGCGGTTTGACTCACGGGGATT
TCCAAGTCTCCACCCCATTGACGTCAATGGGAGTTTGTTTTGGCACCAAAA
TCAACGGGACTTTCCAAAATGTCGTAACAACTCCGCCCCATTGACGCAAAT
GGGCGGTAGGCGTGTACGGTGGGAGGTCTATATAAGCAGAGCTCTCCCTAT
CAGTGATAGAGATCTCCCTATCAGTGATAGAGATCGTCGACGAGCTCGTTTA
GTGAACCGTCAGATCGCCTGGAGACGCCATCCACGCTGTTTTGACCTCCAT
AGAAGACACCGGGACCGATCCAGCCTCCGGACTCTAGCGTTTAAACTTAA
GCTTATGAACTGGTCCCATTCCTGCATCTCCTTTTGCTGGATCTACTTTGCTG
CTTCCAGACTGAGAGCTGCAGAGACGGCAGATGGAAAATATGCTCAGAAG
TTGTTTAATGACCTTTTTGAAGATTATTCTAATGCTCTTCGTCCAGTGGAAG
ATACAGATAAAGTCCTGAATGTGACCCTGCAGATTACGCTCTCTCAGATTAA
GGATATGGATGAAAGAAACCAAATTCTGACTGCTTATTTGTGGATCCGCCA
AATCTGGCACGATGCCTATCTCACGTGGGACCGAGATCAGTACGATGGCCT
AGACTCCATCAGGATCCCCAGTGACCTCGTGTGGAGGCCAGACATCGTCTT

ATATAACAAGGCTGATGATGAATCTTCAGAGCCTGTGAACACCAATGTGGT
CCTGCGGTATGATGGGCTGATCACCTGGGATGCACCGGCCATCACCAAAAG
CTCCTGTGTGGTGGATGTCACCTACTTCCCTTTTGACAACCAGCAGTGCAA
CCTGACTTTTGGTTCCTGGACCTACAATGGCAATCAGGTGGACATATTCAAC
GCCTTGGACAGCGGAGATCTCTCTGACTTCATTGAAGATGTGGAATGGGAG
GTCCATGGCATGCCCGCTGTGAAGAATGTGATCTCCTATGGCTGCTGCTCTG
AGCCTTACCCGGATGTCACATTCACCCTCCTTCTGAAGAGGAGGTCCTCGT
TCTATATCGTCAACCTCCTCATCCCATGCGTCCTCATATCTTTTCTGGCTCCT
CTGAGTTTTTATCTCCCAGCAGCCTCCGGAGAAAAGGTCTCCCTGGGAGTG
ACCATCCTGTTGGCCATGACTGTATTTCAGCTAATGGTGGCAGAAATCATGC
CGGCCTCAGAAAATGTGCCCCTGATAGGTAAATACTACATAGCCACGATGG
CCCTGATCACAGCCTCCACTGCGTTGACCATCATGGTGATGAATATCCACTT
CTGTGGGGCCGAGGCCCGGCCGGTGCCACACTGGGCCAGGGTGGTCATCC
TGAAATACATGTCCAGGGTCTTGTTTGTCTATGATGTGGGTGAAAGCTGCCT
CAGCCCGCACCACAGTAGAGAGCGGGACCACCTCACGAAAGTTTATAGCA
AACTCCCAGAGTCTAACCTGAAAGCAGCCAGGAACAAAGACCTTTCCAGA
AAGAAGGACATGAACAAACGCTTAAAGAACGACCTGGGCTGCCAGGGTA
AGAACCCTCAGGAGGCCGAGAGTTACTGTGCACAGTACAAAGTGCTGACG
AGGAATATTGAGTACATCGCCAAGTGCCTCAAAGACCACAAGGCCACCAG
TTCCAAGGGGAGTGAATGGAAGAAGGTGGCGAAAGTCATAGACCGATTCT
TCATGTGGATTTTTTTCATTATGGTGTTTGTGATGACTATTTTGATCATAGCA
AGAGCGGATGGTACCGAGCTCGGATCCACTAGTCCAGTGTGGTGGAATTCT
GCAGATATCCAGCACAGTGGCGGCCGCTCGAGGATGGCCCTGTCCAACAA
GTTCATCGGCGACGACATGAAGATGACCTACCACATGGACGGCTGCGTGAA
CGGCCACTACTTCACCGTGAAGGGCGAGGGCAGCGGCAAGCCCTACGAGG
GCACCCAGACCTCCACCTTCAAGGTGACCATGGCCAACGGCGGCCCCCTG
GCCTTCTCCTTCGACATCCTGTCCACCGTGTTCATGTACGGCAACCGCTGCT
TCACCGCCTACCCCACCAGCATGCCCGACTACTTCAAGCAGGCCTTCCCCG
ACGGCATGTCCTACGAGAGAACCTTCACCTACGAGGACGGCGGCGTGGCC
ACCGCCAGCTGGGAGATCAGCCTGAAGGGCAACTGCTTCGAGCACAAGTC
CACCTTCCACGGCGTGAACTTCCCCGCCGACGGCCCCGTGATGGCCAAGA
AGACCACCGGCTGGGACCCCTCCTTCGAGAAGATGACCGTGTGCGACGGC
ATCTTGAAGGGCGACGTGACCGCCTTCCTGATGCTGCAGGGCGGCGGCAA
CTACAGATGCCAGTTCCACACCTCCTACAAGACCAAGAAGCCCGTGACCAT
GCCCCCCAACCACGTGGTGGAGCACCGCATCGCCAGAACCGACCTGGACA
AGGGCGGCAACAGCGTGCAGCTGACCGAGCACGCCGTGGCCCACATCACC
TCCGTGGTGCCCTTCTAACTCTAGAGGGCCCGTTTAAACCCGCTGATCAGC
CTCGACTGTGCCTTCTAGTTGCCAGCCATCTGTTGTTTGCCCCTCCCCCGTG

CCTTCCTTGACCCTGGAAGGTGCCACTCCCACTGTCCTTTCCTAATAAAATG
AGGAAATTGCATCGCATTGTCTGAGTAGGTGTCATTCTATTCTGGGGGGTGG
GGTGGGGCAGGACAGCAAGGGGGAGGATTGGGAAGACAATAGCAGGCAT
GCTGGGGATGCGGTGGGCTCTATGGCTTCTGAGGCGGAAAGAACCAGCTG
GGGCTCTAGGGGGTATCCCCACGCGCCCTGTAGCGGCGCATTAAGCGCGGC
GGGTGTGGTGGTTACGCGCAGCGTGACCGCTACACTTGCCAGCGCCCTAGC
GCCCGCTCCTTTCGCTTTCTTCCCTTCCTTTCTCGCCACGTTCGCCGGCTTT
CCCCGTCAAGCTCTAAATCGGGGGCTCCCTTTAGGGTTCCGATTTAGTGCTT
TACGGCACCTCGACCCCAAAAAACTTGATTAGGGTGATGGTTCACGTAGTG
GGCCATCGCCCTGATAGACGGTTTTTCGCCCTTTGACGTTGGAGTCCACGT
TCTTTAATAGTGGACTCTTGTTCCAAACTGGAACAACACTCAACCCTATCTC
GGTCTATTCTTTTGATTTATAAGGGATTTTGCCGATTTCGGCCTATTGGTTAA
AAAATGAGCTGATTTAACAAAAATTTAACGCGAATTAATTCTGTGGAATGTG
TGTCAGTTAGGGTGTGGAAAGTCCCCAGGCTCCCCAGCAGGCAGAAGTAT
GCAAAGCATGCATCTCAATTAGTCAGCAACCAGGTGTGGAAAGTCCCCAG
GCTCCCCAGCAGGCAGAAGTATGCAAAGCATGCATCTCAATTAGTCAGCAA
CCATAGTCCCGCCCCTAACTCCGCCCATCCCGCCCCTAACTCCGCCCAGTTC
CGCCCATTCTCCGCCCCATGGCTGACTAATTTTTTTTATTTATGCAGAGGCCG
AGGCCGCCTCTGCCTCTGAGCTATTCCAGAAGTAGTGAGGAGGCTTTTTTG
GAGGCCTAGGCTTTTGCAAAAAGCTCCCGGGAGCTTGTATATCCATTTTCG
GATCTGATCAGCACGTGATGAAAAAGCCTGAACTCACCGCGACGTCTGTC
GAGAAGTTTCTGATCGAAAAGTTCGACAGCGTCTCCGACCTGATGCAGCTC
TCGGAGGGCGAAGAATCTCGTGCTTTCAGCTTCGATGTAGGAGGGCGTGG
ATATGTCCTGCGGGTAAATAGCTGCGCCGATGGTTTCTACAAAGATCGTTAT
GTTTATCGGCACTTTGCATCGGCCGCGCTCCCGATTCCGGAAGTGCTTGAC
ATTGGGGAATTCAGCGAGAGCCTGACCTATTGCATCTCCCGCCGTGCACAG
GGTGTCACGTTGCAAGACCTGCCTGAAACCGAACTGCCCGCTGTTCTGCA
GCCGGTCGCGGAGGCCATGGATGCGATCGCTGCGGCCGATCTTAGCCAGAC
GAGCGGGTTCGGCCCATTCGGACCGCAAGGAATCGGTCAATACACTACATG
GCGTGATTTCATATGCGCGATTGCTGATCCCCATGTGTATCACTGGCAAACT
GTGATGGACGACACCGTCAGTGCGTCCGTCGCGCAGGCTCTCGATGAGCT
GATGCTTTGGGCCGAGGACTGCCCCGAAGTCCGGCACCTCGTGCACGCGG
ATTTCGGCTCCAACAATGTCCTGACGGACAATGGCCGCATAACAGCGGTCA
TTGACTGGAGCGAGGCGATGTTCGGGGATTCCCAATACGAGGTCGCCAAC
ATCTTCTTCTGGAGGCCGTGGTTGGCTTGTATGGAGCAGCAGACGCGCTAC
TTCGAGCGGAGGCATCCGGAGCTTGCAGGATCGCCGCGGCTCCGGGCGTAT
ATGCTCCGCATTGGTCTTGACCAACTCTATCAGAGCTTGGTTGACGGCAATT
TCGATGATGCAGCTTGGGCGCAGGTCGATGCGACGCAATCGTCCGATCCG

GAGCCGGGACTGTCGGGCGTACACAAATCGCCCGCAGAAGCGCGGCCGTC
TGGACCGATGGCTGTGTAGAAGTACTCGCCGATAGTGGAAACCGACGCCCC
AGCACTCGTCCGAGGGCAAAGGAATAGCACGTGCTACGAGATTTCGATTCC
ACCGCCGCCTTCTATGAAAGGTTGGGCTTCGGAATCGTTTTCCGGGACGCC
GGCTGGATGATCCTCCAGCGCGGGGATCTCATGCTGGAGTTCTTCGCCCAC
CCCAACTTGTTTATTGCAGCTTATAATGGTTACAAATAAAGCAATAGCATCA
CAAATTTCACAAATAAAGCATTTTTTTCACTGCATTCTAGTTGTGGTTTGTC
CAAACTCATCAATGTATCTTATCATGTCTGTATACCGTCGACCTCTAGCTAGA
GCTTGGCGTAATCATGGTCATAGCTGTTTCCTGTGTGAAATTGTTATCCGCT
CACAATTCCACACAACATACGAGCCGGAAGCATAAAGTGTAAAGCCTGGG
GTGCCTAATGAGTGAGCTAACTCACATTAATTGCGTTGCGCTCACTGCCCGC
TTTCCAGTCGGGAAACCTGTCGTGCCAGCTGCATTAATGAATCGGCCAACG
CGCGGGGAGAGGCGGTTTGCGTATTGGGCGCTCTTCCGCTTCCTCGCTCAC
TGACTCGCTGCGCTCGGTCGTTCGGCTGCGGCGAGCGGTATCAGCTCACTC
AAAGGCGGTAATACGGTTATCCACAGAATCAGGGGATAACGCAGGAAAGA
ACATGTGAGCAAAAGGCCAGCAAAAGGCCAGGAACCGTAAAAAGGCCGC
GTTGCTGGCGTTTTTCCATAGGCTCCGCCCCCCTGACGAGCATCACAAAAA
TCGACGCTCAAGTCAGAGGTGGCGAAACCCGACAGGACTATAAAGATACC
AGGCGTTTCCCCCTGGAAGCTCCCTCGTGCGCTCTCCTGTTCCGACCCTGC
CGCTTACCGGATACCTGTCCGCCTTTCTCCCTTCGGGAAGCGTGGCGCTTTC
TCATAGCTCACGCTGTAGGTATCTCAGTTCGGTGTAGGTCGTTCGCTCCAAG
CTGGGCTGTGTGCACGAACCCCCCGTTCAGCCCGACCGCTGCGCCTTATCC
GGTAACTATCGTCTTGAGTCCAACCCGGTAAGACACGACTTATCGCCACTG
GCAGCAGCCACTGGTAACAGGATTAGCAGAGCGAGGTATGTAGGCGGTGC
TACAGAGTTCTTGAAGTGGTGGCCTAACTACGGCTACACTAGAAGAACAGT
ATTTGGTATCTGCGCTCTGCTGAAGCCAGTTACCTTCGGAAAAAGAGTTGG
TAGCTCTTGATCCGGCAAACAAACCACCGCTGGTAGCGGTTGGTTTTTTGT
TTGCAAGCAGCAGATTACGCGCAGAAAAAAAGGATCTCAAGAAGATCCTT
TGATCTTTTCTACGGGGTCTGACGCTCAGTGGAACGAAAACTCACGTTAAG
GGATTTTGGTCATGAGATTATCAAAAAGGATCTTCACCTAGATCCTTTTAAA
TTAAAAATGAAGTTTTAAATCAATCTAAAGTATATATGAGTAAACTTGGTCT
GACAGTTACCAATGCTTAATCAGTGAGGCACCTATCTCAGCGATCTGTCTAT
TTCGTTCATCCATAGTTGCCTGACTCCCCGTCGTGTAGATAACTACGATACG
GGAGGGCTTACCATCTGGCCCCAGTGCTGCAATGATACCGCGAGACCCACG
CTCACCGGCTCCAGATTTATCAGCAATAAACCAGCCAGCCGGAAGGGCCG
AGCGCAGAAGTGGTCCTGCAACTTTATCCGCCTCCATCCAGTCTATTAATTG
TTGCCGGGAAGCTAGAGTAAGTAGTTCGCCAGTTAATAGTTTGCGCAACGT
TGTTGCCATTGCTACAGGCATCGTGGTGTCACGCTCGTCGTTTGGTATGGCT

TCATTCAGCTCCGGTTCCCAACGATCAAGGCGAGTTACATGATCCCCCATGT
TGTGCAAAAAAGCGGTTAGCTCCTTCGGTCCTCCGATCGTTGTCAGAAGTA
AGTTGGCCGCAGTGTTATCACTCATGGTTATGGCAGCACTGCATAATTCTCT
TACTGTCATGCCATCCGTAAGATGCTTTTCTGTGACTGGTGAGTACTCAACC
AAGTCATTCTGAGAATAGTGTATGCGGCGACCGAGTTGCTCTTGCCCGGCG
TCAATACGGGATAATACCGCGCCACATAGCAGAACTTTAAAAGTGCTCATC
ATTGGAAAACGTTCTTCGGGGCGAAAACTCTCAAGGATCTTACCGCTGTTG
AGATCCAGTTCGATGTAACCCACTCGTGCACCCAACTGATCTTCAGCATCTT
TTACTTTCACCAGCGTTTCTGGGTGAGCAAAAACAGGAAGGCAAAATGCC
GCAAAAAAGGGAATAAGGGCGACACGGAAATGTTGAATACTCATACTCTTC
CTTTTTCAATATTATTGAAGCATTTATCAGGGTTATTGTCTCATGAGCGGATA
CATATTTGAATGTATTTAGAAAAATAAACAAATAGGGGTTCCGCGCACATTT
CCCCGAAAAGTGCCACCTGACGTC

PCDNA5 ERBB2-YFP

PROMOTER

ERBB2

Linker

YFP

GACGGATCGGGAGATCTCCCGATCCCCTATGGTGCACTCTCAGTACAATCT
GCTCTGATGCCGCATAGTTAAGCCAGTATCTGCTCCCTGCTTGTGTGTTGGA
GGTCGCTGAGTAGTGCGCGAGCAAAATTTAAGCTACAACAAGGCAAGGCT
TGACCGACAATTGCATGAAGAATCTGCTTAGGGTTAGGCGTTTTGCGCTGC
TTCGCGATGTACGGGCCAGATATACGCGTTGACATTGATTATTGACTAGTTAT
TAATAGTAATCAATTACGGGGTCATTAGTTCATAGCCCATATATGGAGTTCCG
CGTTACATAACTTACGGTAAATGGCCCGCCTGGCTGACCGCCCAACGACCC
CCGCCCATTGACGTCAATAATGACGTATGTTCCCATAGTAACGCCAATAGGG
ACTTTCCATTGACGTCAATGGGTGGAGTATTTACGGTAAACTGCCCACTTGG
CAGTACATCAAGTGTATCATATGCCAAGTACGCCCCCTATTGACGTCAATGA
CGGTAAATGGCCCGCCTGGCATTATGCCCAGTACATGACCTTATGGGACTTT
CCTACTTGGCAGTACATCTACGTATTAGTCATCGCTATTACCATGGTGATGCG
GTTTTGGCAGTACATCAATGGGCGTGGATAGCGGTTTGACTCACGGGGATT
TCCAAGTCTCCACCCCATTGACGTCAATGGGAGTTTGTTTTGGCACCAAAA
TCAACGGGACTTTCCAAAATGTCGTAACAACTCCGCCCCATTGACGCAAAT
GGGCGGTAGGCGTGTACGGTGGGAGGTCTATATAAGCAGAGCTCTCCCTAT
CAGTGATAGAGATCTCCCTATCAGTGATAGAGATCGTCGACGAGCTCGTTTA
GTGAACCGTCAGATCGCCTGGAGACGCCATCCACGCTGTTTTGACCTCCAT
AGAAGACACCGGGACCGATCCAGCCTCCGGACTCTAGCGTTTAAACTTAA
GCTTATGGAGCTGGCGGCCTTGTGCCGCTGGGGGCTCCTCCTCGCCCTCTT
GCCCCCCGGAGCCGCGAGCACCCAAGTGTGCACCGGCACAGACATGAAGC
TGCGGCTCCCTGCCAGTCCCGAGACCCACCTGGACATGCTCCGCCACCTCT
ACCAGGGCTGCCAGGTGGTGCAGGGAAACCTGGAACTCACCTACCTGCCC
ACCAATGCCAGCCTGTCCTTCCTGCAGGATATCCAGGAGGTGCAGGGCTAC
GTGCTCATCGCTCACAACCAAGTGAGGCAGGTCCCACTGCAGAGGCTGCG
GATTGTGCGAGGCACCCAGCTCTTTGAGGACAACTATGCCCTGGCCGTGCT
AGACAATGGAGACCCGCTGAACAATACCACCCCTGTCACAGGGGCCTCCC
CAGGAGGCCTGCGGGAGCTGCAGCTTCGAAGCCTCACAGAGATCTTGAAA
GGAGGGGTCTTGATCCAGCGGAACCCCCAGCTCTGCTACCAGGACACGAT
TTTGTGGAAGGACATCTTCCACAAGAACAACCAGCTGGCTCTCACACTGAT
AGACACCAACCGCTCTCGGGCCTGCCACCCCTGTTCTCCGATGTGTAAGGG
CTCCCGCTGCTGGGGAGAGAGTTCTGAGGATTGTCAGAGCCTGACGCGCA
CTGTCTGTGCCGGTGGCTGTGCCCGCTGCAAGGGGCCACTGCCCACTGAC

TGCTGCCATGAGCAGTGTGCTGCCGGCTGCACGGGCCCCAAGCACTCTGA
CTGCCTGGCCTGCCTCCACTTCAACCACAGTGGCATCTGTGAGCTGCACTG
CCCAGCCCTGGTCACCTACAACACAGACACGTTTGAGTCCATGCCCAATCC
CGAGGGCCGGTATACATTCGGCGCCAGCTGTGTGACTGCCTGTCCCTACAA
CTACCTTTCTACGGACGTGGGATCCTGCACCCTCGTCTGCCCCCTGCACAA
CCAAGAGGTGACAGCAGAGGATGGAACACAGCGGTGTGAGAAGTGCAGC
AAGCCCTGTGCCCGAGTGTGCTATGGTCTGGGCATGGAGCACTTGCGAGA
GGTGAGGGCAGTTACCAGTGCCAATATCCAGGAGTTTGCTGGCTGCAAGA
AGATCTTTGGGAGCCTGGCATTTCTGCCGGAGAGCTTTGATGGGGACCCAG
CCTCCAACACTGCCCCGCTCCAGCCAGAGCAGCTCCAAGTGTTTGAGACT
CTGGAAGAGATCACAGGTTACCTATACATCTCAGCATGGCCGGACAGCCTG
CCTGACCTCAGCGTCTTCCAGAACCTGCAAGTAATCCGGGGACGAATTCTG
CACAATGGCGCCTACTCGCTGACCCTGCAAGGGCTGGGCATCAGCTGGCTG
GGGCTGCGCTCACTGAGGGAACTGGGCAGTGGACTGGCCCTCATCCACCA
TAACACCCACCTCTGCTTCGTGCACACGGTGCCCTGGGACCAGCTCTTTCG
GAACCCGCACCAAGCTCTGCTCCACACTGCCAACCGGCCAGAGGACGAGT
GTGTGGGCGAGGGCCTGGCCTGCCACCAGCTGTGCGCCCGAGGGCACTGC
TGGGGTCCAGGGCCCACCCAGTGTGTCAACTGCAGCCAGTTCCTTCGGGG
CCAGGAGTGCGTGGAGGAATGCCGAGTACTGCAGGGGCTCCCCAGGGAGT
ATGTGAATGCCAGGCACTGTTTGCCGTGCCACCCTGAGTGTCAGCCCCAGA
ATGGCTCAGTGACCTGTTTTGGACCGGAGGCTGACCAGTGTGTGGCCTGTG
CCCACTATAAGGACCCTCCCTTCTGCGTGGCCCGCTGCCCCAGCGGTGTGA
AACCTGACCTCTCCTACATGCCCATCTGGAAGTTTCCAGATGAGGAGGGCG
CATGCCAGCCTTGCCCCATCAACTGCACCCACTCCTGTGTGGACCTGGATG
ACAAGGGCTGCCCCGCCGAGCAGAGAGCCAGCCCTCTGACGTCCATCATC
TCTGCGGTGGTTGGCATTCTGCTGGTCGTGGTCTTGGGGGTGGTCTTTGGG
ATCCTCATCAAGCGACGGCAGCAGAAGATCCGGAAGTACACGATGCGGAG
ACTGCTGCAGGAAACGGAGCTGGTGGAGCCGCTGACACCTAGCGGAGCG
ATGCCCAACCAGGCGCAGATGCGGATCCTGAAAGAGACGGAGCTGAGGAA
GGTGAAGGTGCTTGGATCTGGCGCTTTTGGCACAGTCTACAAGGGCATCTG
GATCCCTGATGGGGAGAATGTGAAAATTCCAGTGGCCATCAAAGTGTTGAG
GGAAAACACATCCCCCAAAGCCAACAAAGAAATCTTAGACGAAGCATACG
TGATGGCTGGTGTGGGCTCCCCATATGTCTCCCGCCTTCTGGGCATCTGCCT
GACATCCACGGTGCAGCTGGTGACACAGCTTATGCCCTATGGCTGCCTCTT
AGACCATGTCCGGGAAAACCGCGGACGCCTGGGCTCCCAGGACCTGCTGA
ACTGGTGTATGCAGATTGCCAAGGGGATGAGCTACCTGGAGGATGTGCGGC
TCGTACACAGGGACTTGGCCGCTCGGAACGTGCTGGTCAAGAGTCCCAAC
CATGTCAAAATTACAGACTTCGGGCTGGCTCGGCTGCTGGACATTGACGAG

ACAGAGTACCATGCAGATGGGGGCAAGGTGCCCATCAAGTGGATGGCGCT
GGAGTCCATTCTCCGCCGGCGGTTCACCCACCAGAGTGATGTGTGGAGTTA
TGGTGTGACTGTGTGGGAGCTGATGACTTTTGGGGCCAAACCTTACGATGG
GATCCCAGCCCGGGAGATCCCTGACCTGCTGGAAAAGGGGGAGCGGCTGC
CCCAGCCCCCCATCTGCACCATTGATGTCTACATGATCATGGTCAAATGTTG
GATGATTGACTCTGAATGTCGGCCAAGATTCCGGGAGTTGGTGTCTGAATT
CTCCCGCATGGCCAGGGACCCCCAGCGCTTTGTGGTCATCCAGAATGAGGA
CTTGGGCCCAGCCAGTCCCTTGGACAGCACCTTCTACCGCTCACTGCTGGA
GGACGATGACATGGGGGACCTGGTGGATGCTGAGGAGTATCTGGTACCCCA
GCAGGGCTTCTTCTGTCCAGACCCTGCCCCGGGCGCTGGGGGCATGGTCC
ACCACAGGCACCGCAGCTCATCTACCAGGAGTGGCGGTGGGGACCTGACA
CTAGGGCTGGAGCCCTCTGAAGAGGAGGCCCCCAGGTCTCCACTGGCACC
CTCCGAAGGGGCTGGCTCCGATGTATTTGATGGTGACCTGGGAATGGGGGC
AGCCAAGGGGCTGCAAAGCCTCCCCACACATGACCCCAGCCCTCTACAGC
GGTACAGTGAGGACCCCACAGTACCCCTGCCCTCTGAGACTGATGGCTACG
TTGCCCCCCTGACCTGCAGCCCCCAGCCTGAATATGTGAACCAGCCAGATG
TTCGGCCCCAGCCCCCTTCGCCCCGAGAGGGCCCTCTGCCTGCTGCCCGAC
CTGCTGGTGCCACTCTGGAAAGGCCCAAGACTCTCTCCCCAGGGAAGAAT
GGGGTCGTCAAAGACGTTTTTGCCTTTGGGGGTGCCGTGGAGAACCCCGA
GTACTTGACACCCCAGGGAGGAGCTGCCCCTCAGCCCCACCCTCCTCCTGC
CTTCAGCCCAGCCTTCGACAACCTCTATTACTGGGACCAGGACCCACCAGA
GCGGGGGGCTCCACCCAGCACCTTCAAAGGGACACCTACGGCAGAGAACC
CAGAGTACCTGGGTCTGGACGTGCCAGTGGCAGCAGCAGGCGGCCGCTCG
AGGATGGCCCACAGCAAGCACGGCCTGAAGGAGGAGATGACCATGAAGTA
CCACATGGAGGGCTGCGTGAACGGCCACAAGTTCGTGATCACCGGCGAGG
GCATCGGCTACCCCTTCAAGGGCAAGCAGACCATCAACCTGTGCGTGATCG
AGGGCGGCCCCCTGCCCTTCAGCGAGGACATCCTGAGCGCCGGCTTCAAG
TACGGCGACCGGATCTTCACCGAGTACCCCCAGGACATCGTGGACTACTTC
AAGAACAGCTGCCCCGCCGGCTACACCTGGGGCCGGAGCTTCCTGTTCGA
GGACGGCGCCGTGTGCATCTGTAACGTGGACATCACCGTGAGCGTGAAGG
AGAACTGCATCTACCACAAGAGCATCTTCAACGGCGTGAACTTCCCCGCCG
ACGGCCCCGTGATGAAGAAGATGACCACCAACTGGGAGGCCAGCTGCGAG
AAGATCATGCCCGTGCCTAAGCAGGGCATCCTGAAGGGCGACGTGAGCAT
GTACCTGCTGCTGAAGGACGGCGGCCGGTACCGGTGCCAGTTCGACACCG
TGTACAAGGCCAAGAGCGTGCCCAGCAAGATGCCCGAGTGGCACTTCATC
CAGCACAAGCTGCTGCGGGAGGACCGGAGCGACGCCAAGAACCAGAAGT
GGCAGCTGACCGAGCACGCCATCGCCTTCCCCAGCGCCCTGGCCTAAGTCT
AGAGGGCCCGTTTAAACCCGCTGATCAGCCTCGACTGTGCCTTCTAGTTGC

CAGCCATCTGTTGTTTGCCCCTCCCCCGTGCCTTCCTTGACCCTGGAAGGT
GCCACTCCCACTGTCCTTTCCTAATAAAATGAGGAAATTGCATCGCATTGTC
TGAGTAGGTGTCATTCTATTCTGGGGGGTGGGGTGGGGCAGGACAGCAAG
GGGGAGGATTGGGAAGACAATAGCAGGCATGCTGGGGATGCGGTGGGCTC
TATGGCTTCTGAGGCGGAAAGAACCAGCTGGGGCTCTAGGGGGTATCCCCA
CGCGCCCTGTAGCGGCGCATTAAGCGCGGCGGGTGTGGTGGTTACGCGCA
GCGTGACCGCTACACTTGCCAGCGCCCTAGCGCCCGCTCCTTTCGCTTTCT
TCCCTTCCTTTCTCGCCACGTTCGCCGGCTTTCCCCGTCAAGCTCTAAATCG
GGGGCTCCCTTTAGGGTTCCGATTTAGTGCTTTACGGCACCTCGACCCCAA
AAAACTTGATTAGGGTGATGGTTCACGTAGTGGGCCATCGCCCTGATAGAC
GGTTTTTCGCCCTTTGACGTTGGAGTCCACGTTCTTTAATAGTGGACTCTTG
TTCCAAACTGGAACAACACTCAACCCTATCTCGGTCTATTCTTTTGATTTAT
AAGGGATTTTGCCGATTTCGGCCTATTGGTTAAAAAATGAGCTGATTTAACA
AAAATTTAACGCGAATTAATTCTGTGGAATGTGTGTCAGTTAGGGTGTGGA
AAGTCCCCAGGCTCCCCAGCAGGCAGAAGTATGCAAAGCATGCATCTCAAT
TAGTCAGCAACCAGGTGTGGAAAGTCCCCAGGCTCCCCAGCAGGCAGAAG
TATGCAAAGCATGCATCTCAATTAGTCAGCAACCATAGTCCCGCCCCTAACT
CCGCCCATCCCGCCCCTAACTCCGCCCAGTTCCGCCCATTCTCCGCCCCATG
GCTGACTAATTTTTTTTATTTATGCAGAGGCCGAGGCCGCCTCTGCCTCTGA
GCTATTCCAGAAGTAGTGAGGAGGCTTTTTTGGAGGCCTAGGCTTTTGCAA
AAAGCTCCCGGGAGCTTGTATATCCATTTTCGGATCTGATCAGCACGTGATG
AAAAAGCCTGAACTCACCGCGACGTCTGTCGAGAAGTTTCTGATCGAAAA
GTTCGACAGCGTCTCCGACCTGATGCAGCTCTCGGAGGGCGAAGAATCTC
GTGCTTTCAGCTTCGATGTAGGAGGGCGTGGATATGTCCTGCGGGTAAATA
GCTGCGCCGATGGTTTCTACAAAGATCGTTATGTTTATCGGCACTTTGCATC
GGCCGCGCTCCCGATTCCGGAAGTGCTTGACATTGGGGAATTCAGCGAGA
GCCTGACCTATTGCATCTCCCGCCGTGCACAGGGTGTCACGTTGCAAGACC
TGCCTGAAACCGAACTGCCCGCTGTTCTGCAGCCGGTCGCGGAGGCCATG
GATGCGATCGCTGCGGCCGATCTTAGCCAGACGAGCGGGTTCGGCCCATTC
GGACCGCAAGGAATCGGTCAATACACTACATGGCGTGATTTCATATGCGCG
ATTGCTGATCCCCATGTGTATCACTGGCAAACTGTGATGGACGACACCGTC
AGTGCGTCCGTCGCGCAGGCTCTCGATGAGCTGATGCTTTGGGCCGAGGA
CTGCCCCGAAGTCCGGCACCTCGTGCACGCGGATTTCGGCTCCAACAATGT
CCTGACGGACAATGGCCGCATAACAGCGGTCATTGACTGGAGCGAGGCGA
TGTTCGGGGATTCCCAATACGAGGTCGCCAACATCTTCTTCTGGAGGCCGT
GGTTGGCTTGTATGGAGCAGCAGACGCGCTACTTCGAGCGGAGGCATCCG
GAGCTTGCAGGATCGCCGCGGCTCCGGGCGTATATGCTCCGCATTGGTCTT
GACCAACTCTATCAGAGCTTGGTTGACGGCAATTTCGATGATGCAGCTTGG

GCGCAGGGTCGATGCGACGCAATCGTCCGATCCGGAGCCGGGACTGTCGG
GCGTACACAAATCGCCCGCAGAAGCGCGGCCGTCTGGACCGATGGCTGTG
TAGAAGTACTCGCCGATAGTGGAAACCGACGCCCCAGCACTCGTCCGAGG
GCAAAGGAATAGCACGTGCTACGAGATTTCGATTCCACCGCCGCCTTCTAT
GAAAGGTTGGGCTTCGGAATCGTTTTCCGGGACGCCGGCTGGATGATCCTC
CAGCGCGGGGATCTCATGCTGGAGTTCTTCGCCCACCCCAACTTGTTTATTG
CAGCTTATAATGGTTACAAATAAAGCAATAGCATCACAAATTTCACAAATAA
AGCATTTTTTTCACTGCATTCTAGTTGTGGTTTGTCCAAACTCATCAATGTAT
CTTATCATGTCTGTATACCGTCGACCTCTAGCTAGAGCTTGGCGTAATCATG
GTCATAGCTGTTTCCTGTGTGAAATTGTTATCCGCTCACAATTCCACACAAC
ATACGAGCCGGAAGCATAAAGTGTAAAGCCTGGGGTGCCTAATGAGTGAG
CTAACTCACATTAATTGCGTTGCGCTCACTGCCCGCTTTCCAGTCGGGAAA
CCTGTCGTGCCAGCTGCATTAATGAATCGGCCAACGCGCGGGGAGAGGCG
GTTTGCGTATTGGGCGCTCTTCCGCTTCCTCGCTCACTGACTCGCTGCGCTC
GGTCGTTCGGCTGCGGCGAGCGGTATCAGCTCACTCAAAGGCGGTAATACG
GTTATCCACAGAATCAGGGGATAACGCAGGAAAGAACATGTGAGCAAAAG
GCCAGCAAAAGGCCAGGAACCGTAAAAAGGCCGCGTTGCTGGCGTTTTTC
CATAGGCTCCGCCCCCCTGACGAGCATCACAAAAATCGACGCTCAAGTCAG
AGGTGGCGAAACCCGACAGGACTATAAAGATACCAGGCGTTTCCCCCTGG
AAGCTCCCTCGTGCGCTCTCCTGTTCCGACCCTGCCGCTTACCGGATACCT
GTCCGCCTTTCTCCCTTCGGGAAGCGTGGCGCTTTCTCATAGCTCACGCTGT
AGGTATCTCAGTTCGGTGTAGGTCGTTCGCTCCAAGCTGGGCTGTGTGCAC
GAACCCCCCGTTCAGCCCGACCGCTGCGCCTTATCCGGTAACTATCGTCTTG
AGTCCAACCCGGTAAGACACGACTTATCGCCACTGGCAGCAGCCACTGGT
AACAGGATTAGCAGAGCGAGGTATGTAGGCGGTGCTACAGAGTTCTTGAA
GTGGTGGCCTAACTACGGCTACACTAGAAGAACAGTATTTGGTATCTGCGC
TCTGCTGAAGCCAGTTACCTTCGGAAAAAGAGTTGGTAGCTCTTGATCCGG
CAAACAAACCACCGCTGGTAGCGGTTGGTTTTTTTGTTTGCAAGCAGCAGAT
TACGCGCAGAAAAAAAGGATCTCAAGAAGATCCTTTGATCTTTTCTACGGG
GTCTGACGCTCAGTGGAACGAAAACTCACGTTAAGGGATTTTGGTCATGAG
ATTATCAAAAAGGATCTTCACCTAGATCCTTTTAAATTAAAAATGAAGTTTT
AAATCAATCTAAAGTATATATGAGTAAACTTGGTCTGACAGTTACCAATGCT
TAATCAGTGAGGCACCTATCTCAGCGATCTGTCTATTTCGTTCATCCATAGTT
GCCTGACTCCCCGTCGTGTAGATAACTACGATACGGGAGGGCTTACCATCT
GGCCCCAGTGCTGCAATGATACCGCGAGACCCACGCTCACCGGCTCCAGAT
TTATCAGCAATAAACCAGCCAGCCGGAAGGGCCGAGCGCAGAAGTGGTCC
TGCAACTTTATCCGCCTCCATCCAGTCTATTAATTGTTGCCGGGAAGCTAGA
GTAAGTAGTTCGCCAGTTAATAGTTTGCGCAACGTTGTTGCCATTGCTACAG

GCATCGTGGTGTCACGCTCGTCGTTTGGTATGGCTTCATTCAGCTCCGGTTC
CCAACGATCAAGGCGAGTTACATGATCCCCCATGTTGTGCAAAAAAGCGGT
TAGCTCCTTCGGTCCTCCGATCGTTGTCAGAAGTAAGTTGGCCGCAGTGTT
ATCACTCATGGTTATGGCAGCACTGCATAATTCTCTTACTGTCATGCCATCCG
TAAGATGCTTTTCTGTGACTGGTGAGTACTCAACCAAGTCATTCTGAGAATA
GTGTATGCGGCGACCGAGTTGCTCTTGCCCGGCGTCAATACGGGATAATAC
CGCGCCACATAGCAGAACTTTAAAAGTGCTCATCATTGGAAAACGTTCTTC
GGGGCGAAAACTCTCAAGGATCTTACCGCTGTTGAGATCCAGTTCGATGTA
ACCCACTCGTGCACCCAACTGATCTTCAGCATCTTTTACTTTCACCAGCGTT
TCTGGGTGAGCAAAAACAGGAAGGCAAAATGCCGCAAAAAAGGGAATAA
GGGCGACACGGAAATGTTGAATACTCATACTCTTCCTTTTTCAATATTATTG
AAGCATTTATCAGGGTTATTGTCTCATGAGCGGATACATATTTGAATGTATTT
AGAAAAATAAACAAATAGGGGTTCCGCGCACATTTCCCCGAAAAGTGCCA
CCTGACGTC

PCDNA3.1 CHRNA9-Cluc

PROMOTER

Cluc

Linker

CHRNA9

GACGGATCGGGAGATCTCCCGATCCCCTATGGTGCACTCTCAGTACAATCT
GCTCTGATGCCGCATAGTTAAGCCAGTATCTGCTCCCTGCTTGTGTGTTGGA
GGTCGCTGAGTAGTGCGCGAGCAAAATTTAAGCTACAACAAGGCAAGGCT
TGACCGACAATTGCATGAAGAATCTGCTTAGGGTTAGGCGTTTTGCGCTGC
TTCGCGATGTACGGGCCAGATATACGCGTTGACATTGATTATTGACTAGTTAT
TAATAGTAATCAATTACGGGGTCATTAGTTCATAGCCCATATATGGAGTTCCG
CGTTACATAACTTACGGTAAATGGCCCGCCTGGCTGACCGCCCAACGACCC
CCGCCCATTGACGTCAATAATGACGTATGTTCCCATAGTAACGCCAATAGGG
ACTTTCCATTGACGTCAATGGGTGGAGTATTTACGGTAAACTGCCCACTTGG
CAGTACATCAAGTGTATCATATGCCAAGTACGCCCCCTATTGACGTCAATGA
CGGTAAATGGCCCGCCTGGCATTATGCCCAGTACATGACCTTATGGGACTTT
CCTACTTGGCAGTACATCTACGTATTAGTCATCGCTATTACCATGGTGATGCG
GTTTTGGCAGTACATCAATGGGCGTGGATAGCGGTTTGACTCACGGGGATT
TCCAAGTCTCCACCCCATTGACGTCAATGGGAGTTTGTTTTGGCACCAAAA
TCAACGGGACTTTCCAAAATGTCGTAACAACTCCGCCCCATTGACGCAAAT
GGGCGGTAGGCGTGTACGGTGGGAGGTCTATATAAGCAGAGCTCTCTGGCT
AACTAGAGAACCCACTGCTTACTGGCTTATCGAAATTAATACGACTCACTAT
AGGGAGACCCAAGCTGGCTAGCATGAGCGGCTACGTTAACAACCCCGAGG
CTACAAACGCTCTCATCGACAAGGACGGCTGGCTGCACAGCGGCGACATC
GCCTACTGGGACGAGGACGAGCACTTCTTCATCGTGGACCGGCTGAAGAG
CCTGATCAAATACAAGGGCTACCAGGTAGCCCCAGCCGAACTGGAGAGCA
TCCTGCTGCAACACCCCAACATCTTCGACGCCGGGGTCGCCGGCCTGCCCG
ACGACGATGCCGGCGAGCTGCCCGCCGCAGTCGTCGTGCTGGAACACGGT
AAAACCATGACCGAGAAGGAGATCGTGGACTATGTGGCCAGCCAGGTTAC
AACCGCCAAGAAGCTGCGCGGTGGTGTTGTGTTCGTGGACGAGGTGCCTA
AAGGACTGACCGGCAAGTTGGACGCCCGCAAGATCCGCGAGATTCTCATT
AAGGCCAAGAAGGGCGGCAAGATCGCCGTGGCAAGCTTGGTACCGAGCTC
GGATCCACTAGTCCAGTGTGGTGGAATTCTGCAGATATCCAGCACAGTGGC
GGCCGCTCGAGCCATGAACTGGTCCCATTCCTGCATCTCCTTTTGCTGGATC
TACTTTGCTGCTTCCAGACTGAGAGCTGCAGAGACGGCAGATGGAAAATAT
GCTCAGAAGTTGTTTAATGACCTTTTTGAAGATTATTCTAATGCTCTTCGTCC
AGTGGAAGATACAGATAAAGTCCTGAATGTGACCCTGCAGATTACGCTCTC
TCAGATTAAGGATATGGATGAAAGAAACCAAATTCTGACTGCTTATTTGTGG

ATCCGCCAAATCTGGCACGATGCCTATCTCACGTGGGACCGAGATCAGTAC
GATGGCCTAGACTCCATCAGGATCCCCAGTGACCTCGTGTGGAGGCCAGAC
ATCGTCTTATATAACAAGGCTGATGATGAATCTTCAGAGCCTGTGAACACCA
ATGTGGTCCTGCGGTATGATGGGCTGATCACCTGGGATGCACCGGCCATCA
CCAAAAGCTCCTGTGTGGTGGATGTCACCTACTTCCCTTTTGACAACCAGC
AGTGCAACCTGACTTTTGGTTCCTGGACCTACAATGGCAATCAGGTGGACA
TATTCAACGCCTTGGACAGCGGAGATCTCTCTGACTTCATTGAAGATGTGG
AATGGGAGGTCCATGGCATGCCCGCTGTGAAGAATGTGATCTCCTATGGCT
GCTGCTCTGAGCCTTACCCGGATGTCACATTCACCCTCCTTCTGAAGAGGA
GGTCCTCGTTCTATATCGTCAACCTCCTCATCCCATGCGTCCTCATATCTTTT
CTGGCTCCTCTGAGTTTTTATCTCCCAGCAGCCTCCGGAGAAAAGGTCTCC
CTGGGAGTGACCATCCTGTTGGCCATGACTGTATTTCAGCTAATGGTGGCA
GAAATCATGCCGGCCTCAGAAAATGTGCCCCTGATAGGTAAATACTACATAG
CCACGATGGCCCTGATCACAGCCTCCACTGCGTTGACCATCATGGTGATGA
ATATCCACTTCTGTGGGGCCGAGGCCCGGCCGGTGCCACACTGGGCCAGG
GTGGTCATCCTGAAATACATGTCCAGGGTCTTGTTTGTCTATGATGTGGGTG
AAAGCTGCCTCAGCCCGCACCACAGTAGAGAGCGGGACCACCTCACGAAA
GTTTATAGCAAACTCCCAGAGTCTAACCTGAAAGCAGCCAGGAACAAAGA
CCTTTCCAGAAAGAAGGACATGAACAAACGCTTAAAGAACGACCTGGGCT
GCCAGGGTAAGAACCCTCAGGAGGCCGAGAGTTACTGTGCACAGTACAAA
GTGCTGACGAGGAATATTGAGTACATCGCCAAGTGCCTCAAAGACCACAA
GGCCACCAGTTCCAAGGGGAGTGAATGGAAGAAGGTGGCGAAAGTCATAG
ACCGATTCTTCATGTGGATTTTTTTCATTATGGTGTTTGTGATGACTATTTTG
ATCATAGCAAGAGCGGATTAGTCTAGAGGGCCCGTTTAAACCCGCTGATCA
GCCTCGACTGTGCCTTCTAGTTGCCAGCCATCTGTTGTTTGCCCCTCCCCCG
TGCCTTCCTTGACCCTGGAAGGTGCCACTCCCACTGTCCTTTCCTAATAAA
ATGAGGAAATTGCATCGCATTGTCTGAGTAGGTGTCATTCTATTCTGGGGGG
TGGGGTGGGGCAGGACAGCAAGGGGGAGGATTGGGAAGACAATAGCAGG
CATGCTGGGGATGCGGTGGGCTCTATGGCTTCTGAGGCGGAAAGAACCAG
CTGGGGCTCTAGGGGGTATCCCCACGCGCCCTGTAGCGGCGCATTAAGCGC
GGCGGGTGTGGTGGTTACGCGCAGCGTGACCGCTACACTTGCCAGCGCCC
TAGCGCCCGCTCCTTTCGCTTTCTTCCCTTCCTTTCTCGCCACGTTCGCCGG
CTTTCCCCGTCAAGCTCTAAATCGGGGGCTCCCTTTAGGGTTCCGATTTAGT
GCTTTACGGCACCTCGACCCCAAAAAACTTGATTAGGGTGATGGTTCACGT
AGTGGGCCATCGCCCTGATAGACGGTTTTTCGCCCTTTGACGTTGGAGTCC
ACGTTCTTTAATAGTGGACTCTTGTTCCAAACTGGAACAACACTCAACCCT
ATCTCGGTCTATTCTTTTGATTTATAAGGGATTTTGCCGATTTCGGCCTATTG
GTTAAAAAATGAGCTGATTTAACAAAAATTTAACGCGAATTAATTCTGTGG

AATGTGTGTCAGTTAGGGTGTGGAAAGTCCCCAGGCTCCCCAGCAGGCAG
AAGTATGCAAAGCATGCATCTCAATTAGTCAGCAACCAGGTGTGGAAAGTC
CCCAGGCTCCCCAGCAGGCAGAAGTATGCAAAGCATGCATCTCAATTAGTC
AGCAACCATAGTCCCGCCCCTAACTCCGCCCATCCCGCCCCTAACTCCGCC
CAGTTCCGCCCATTCTCCGCCCCATGGCTGACTAATTTTTTTTATTTATGCAG
AGGCCGAGGCCGCCTCTGCCTCTGAGCTATTCCAGAAGTAGTGAGGAGGC
TTTTTTGGAGGCCTAGGCTTTTGCAAAAAGCTCCCGGGAGCTTGTATATCCA
TTTTCGGATCTGATCAAGAGACAGGATGAGGATCGTTTCGCATGATTGAAC
AAGATGGATTGCACGCAGGTTCTCCGGCCGCTTGGGTGGAGAGGCTATTCG
GCTATGACTGGGCACAACAGACAATCGGCTGCTCTGATGCCGCCGTGTTCC
GGCTGTCAGCGCAGGGGCGCCCGGTTCTTTTTGTCAAGACCGACCTGTCC
GGTGCCCTGAATGAACTGCAGGACGAGGCAGCGCGGCTATCGTGGCTGGC
CACGACGGGCGTTCCTTGCGCAGCTGTGCTCGACGTTGTCACTGAAGCGG
GAAGGGACTGGCTGCTATTGGGCGAAGTGCCGGGGCAGGATCTCCTGTCAT
CTCACCTTGCTCCTGCCGAGAAAGTATCCATCATGGCTGATGCAATGCGGC
GGCTGCATACGCTTGATCCGGCTACCTGCCCATTCGACCACCAAGCGAAAC
ATCGCATCGAGCGAGCACGTACTCGGATGGAAGCCGGTCTTGTCGATCAGG
ATGATCTGGACGAAGAGCATCAGGGGCTCGCGCCAGCCGAACTGTTCGCC
AGGCTCAAGGCGCGCATGCCCGACGGCGAGGATCTCGTCGTGACCCATGG
CGATGCCTGCTTGCCGAATATCATGGTGGAAAATGGCCGCTTTTCTGGATTC
ATCGACTGTGGCCGGCTGGGTGTGGCGGACCGCTATCAGGACATAGCGTTG
GCTACCCGTGATATTGCTGAAGAGCTTGGCGGCGAATGGGCTGACCGCTTC
CTCGTGCTTTACGGTATCGCCGCTCCCGATTCGCAGCGCATCGCCTTCTATC
GCCTTCTTGACGAGTTCTTCTGAGCGGGACTCTGGGGTTCGAAATGACCGA
CCAAGCGACGCCCAACCTGCCATCACGAGATTTCGATTCCACCGCCGCCTT
CTATGAAAGGTTGGGCTTCGGAATCGTTTTCCGGGACGCCGGCTGGATGAT
CCTCCAGCGCGGGGATCTCATGCTGGAGTTCTTCGCCCACCCCAACTTGTT
TATTGCAGCTTATAATGGTTACAAATAAAGCAATAGCATCACAAATTTCACA
AATAAAGCATTTTTTTCACTGCATTCTAGTTGTGGTTTGTCCAAACTCATCA
ATGTATCTTATCATGTCTGTATACCGTCGACCTCTAGCTAGAGCTTGGCGTAA
TCATGGTCATAGCTGTTTCCTGTGTGAAATTGTTATCCGCTCACAATTCCAC
ACAACATACGAGCCGGAAGCATAAAGTGTAAAGCCTGGGGTGCCTAATGA
GTGAGCTAACTCACATTAATTGCGTTGCGCTCACTGCCCGCTTTCCAGTCG
GGAAACCTGTCGTGCCAGCTGCATTAATGAATCGGCCAACGCGCGGGGAG
AGGCGGTTTGCGTATTGGGCGCTCTTCCGCTTCCTCGCTCACTGACTCGCT
GCGCTCGGTCGTTCGGCTGCGGCGAGCGGTATCAGCTCACTCAAAGGCGG
TAATACGGTTATCCACAGAATCAGGGGATAACGCAGGAAAGAACATGTGAG
CAAAAGGCCAGCAAAAGGCCAGGAACCGTAAAAAGGCCGCGTTGCTGGC

GTTTTTCCATAGGCTCCGCCCCCCTGACGAGCATCACAAAAATCGACGCTC
AAGTCAGAGGTGGCGAAACCCGACAGGACTATAAAGATACCAGGCGTTTC
CCCCTGGAAGCTCCCTCGTGCGCTCTCCTGTTCCGACCCTGCCGCTTACCG
GATACCTGTCCGCCTTTCTCCCTTCGGGAAGCGTGGCGCTTTCTCATAGCTC
ACGCTGTAGGTATCTCAGTTCGGTGTAGGTCGTTCGCTCCAAGCTGGGCTG
TGTGCACGAACCCCCCGTTCAGCCCGACCGCTGCGCCTTATCCGGTAACTA
TCGTCTTGAGTCCAACCCGGTAAGACACGACTTATCGCCACTGGCAGCAGC
CACTGGTAACAGGATTAGCAGAGCGAGGTATGTAGGCGGTGCTACAGAGTT
CTTGAAGTGGTGGCCTAACTACGGCTACACTAGAAGAACAGTATTTGGTAT
CTGCGCTCTGCTGAAGCCAGTTACCTTCGGAAAAAGAGTTGGTAGCTCTTG
ATCCGGCAAACAAACCACCGCTGGTAGCGGTTTTTTTGTTTGCAAGCAGCA
GATTACGCGCAGAAAAAAAGGATCTCAAGAAGATCCTTTGATCTTTTCTAC
GGGGTCTGACGCTCAGTGGAACGAAAACTCACGTTAAGGGATTTTGGTCAT
GAGATTATCAAAAAGGATCTTCACCTAGATCCTTTTAAATTAAAAATGAAGT
TTTAAATCAATCTAAAGTATATATGAGTAAACTTGGTCTGACAGTTACCAAT
GCTTAATCAGTGAGGCACCTATCTCAGCGATCTGTCTATTTCGTTCATCCATA
GTTGCCTGACTCCCCGTCGTGTAGATAACTACGATACGGGAGGGCTTACCAT
CTGGCCCCAGTGCTGCAATGATACCGCGAGACCCACGCTCACCGGCTCCAG
ATTTATCAGCAATAAACCAGCCAGCCGGAAGGGCCGAGCGCAGAAGTGGT
CCTGCAACTTTATCCGCCTCCATCCAGTCTATTAATTGTTGCCGGGAAGCTA
GAGTAAGTAGTTCGCCAGTTAATAGTTTGCGCAACGTTGTTGCCATTGCTAC
AGGCATCGTGGTGTCACGCTCGTCGTTTGGTATGGCTTCATTCAGCTCCGGT
TCCCAACGATCAAGGCGAGTTACATGATCCCCCATGTTGTGCAAAAAAGCG
GTTAGCTCCTTCGGTCCTCCGATCGTTGTCAGAAGTAAGTTGGCCGCAGTG
TTATCACTCATGGTTATGGCAGCACTGCATAATTCTCTTACTGTCATGCCATC
CGTAAGATGCTTTTCTGTGACTGGTGAGTACTCAACCAAGTCATTCTGAGA
ATAGTGTATGCGGCGACCGAGTTGCTCTTGCCCGGCGTCAATACGGGATAAT
ACCGCGCCACATAGCAGAACTTTAAAAGTGCTCATCATTGGAAAACGTTCT
TCGGGGCGAAAACTCTCAAGGATCTTACCGCTGTTGAGATCCAGTTCGATG
TAACCCACTCGTGCACCCAACTGATCTTCAGCATCTTTTACTTTCACCAGCG
TTTCTGGGTGAGCAAAAACAGGAAGGCAAAATGCCGCAAAAAAGGGAAT
AAGGGCGACACGGAAATGTTGAATACTCATACTCTTCCTTTTTCAATATTAT
TGAAGCATTTATCAGGGTTATTGTCTCATGAGCGGATACATATTTGAATGTAT
TTAGAAAAATAAACAAATAGGGGTTCCGCGCACATTTCCCCGAAAAGTGCC
ACCTGACGTC

PCDNA3.1 CHRNA9-Nluc

PROMOTER

Nluc

Linker

CHRNA9

GACGGATCGGGAGATCTCCCGATCCCCTATGGTGCACTCTCAGTACAATCT
GCTCTGATGCCGCATAGTTAAGCCAGTATCTGCTCCCTGCTTGTGTGTTGGA
GGTCGCTGAGTAGTGCGCGAGCAAAATTTAAGCTACAACAAGGCAAGGCT
TGACCGACAATTGCATGAAGAATCTGCTTAGGGTTAGGCGTTTTGCGCTGC
TTCGCGATGTACGGGCCAGATATACGCGTTGACATTGATTATTGACTAGTTAT
TAATAGTAATCAATTACGGGGTCATTAGTTCATAGCCCATATATGGAGTTCCG
CGTTACATAACTTACGGTAAATGGCCCGCCTGGCTGACCGCCCAACGACCC
CCGCCCATTGACGTCAATAATGACGTATGTTCCCATAGTAACGCCAATAGGG
ACTTTCCATTGACGTCAATGGGTGGAGTATTTACGGTAAACTGCCCACTTGG
CAGTACATCAAGTGTATCATATGCCAAGTACGCCCCCTATTGACGTCAATGA
CGGTAAATGGCCCGCCTGGCATTATGCCCAGTACATGACCTTATGGGACTTT
CCTACTTGGCAGTACATCTACGTATTAGTCATCGCTATTACCATGGTGATGCG
GTTTTGGCAGTACATCAATGGGCGTGGATAGCGGTTTGACTCACGGGGATT
TCCAAGTCTCCACCCCATTGACGTCAATGGGAGTTTGTTTTGGCACCAAAA
TCAACGGGACTTTCCAAAATGTCGTAACAACTCCGCCCCATTGACGCAAAT
GGGCGGTAGGCGTGTACGGTGGGAGGTCTATATAAGCAGAGCTCTCTGGCT
AACTAGAGAACCCACTGCTTACTGGCTTATCGAAATTAATACGACTCACTAT
AGGGAGACCCAAGCTGGCTAGCATGGAAGATGCCAAAAACATTAAGAAGG
GCCCAGCGCCATTCTACCCACTCGAAGACGGGACCGCCGGCGAGCAGCTG
CACAAAGCCATGAAGCGCTACGCCCTGGTGCCCGGCACCATCGCCTTTACC
GACGCACATATCGAGGTGGACATTACCTACGCCGAGTACTTCGAGATGAGC
GTTCGGCTGGCAGAAGCTATGAAGCGCTATGGGCTGAATACAAACCATCGG
ATCGTGGTGTGCAGCGAGAATAGCTTGCAGTTCTTCATGCCCGTGTTGGGT
GCCCTGTTCATCGGTGTGGCTGTGGCCCCAGCTAACGACATCTACAACGAG
CGCGAGCTGCTGAACAGCATGGGCATCAGCCAGCCCACCGTCGTATTCGTG
AGCAAGAAAGGGCTGCAAAAGATCCTCAACGTGCAAAAGAAGCTACCGAT
CATACAAAAGATCATCATCATGGATAGCAAGACCGACTACCAGGGCTTCCA
AAGCATGTACACCTTCGTGACTTCCCATTTGCCACCCGGCTTCAACGAGTA
CGACTTCGTGCCCGAGAGCTTCGACCGGGACAAAACCATCGCCCTGATCAT
GAACAGTAGTGGCAGTACCGGATTGCCCAAGGGCGTAGCCCTACCGCACC
GCACCGCTTGTGTCCGATTCAGTCATGCCCGCGACCCCATCTTCGGCAACC
AGATCATCCCCGACACCGCTATCCTCAGCGTGGTGCCATTTCACCACGGCTT
CGGCATGTTCACCACGCTGGGCTACTTGATCTGCGGCTTTCGGGTCGTGCT

CATGTACCGCTTCGAGGAGGAGCTATTCTTGCGCAGCTTGCAAGACTATAA
GATTCAATCTGCCCTGCTGGTGCCCACACTATTTAGCTTCTTCGCTAAGAGC
ACTCTCATCGACAAGTACGACCTAAGCAACTTGCACGAGATCGCCAGCGG
CGGGGCGCCGCTCAGCAAGGAGGTAGGTGAGGCCGTGGCCAAACGCTTCC
ACCTACCAGGCATCCGCCAGGGCTACGGCCTGACAGAAACAACCAGCGCC
ATTCTGATCACCCCCGAAGGGGACGACAAGCCTGGCGCAGTAGGCAAGGT
GGTGCCCTTCTTCGAGGCTAAGGTGGTGGACTTGGACACCGGTAAGACAC
TGGGTGTGAACCAGCGCGGCGAGCTGTGCGTCCGTGGCCCCATGATCATG
AGCGGCTACGTTAACAACCCCGAGGCTACAAACGCTCTCATCGACAAGGA
CGGCGGAAGCTTGGTACCGAGCTCGGATCCACTAGTCCAGTGTGGTGGAAT
TCTGCAGATATCCAGCACAGTGGCGGCCGCTCGAGCCATGAACTGGTCCCA
TTCCTGCATCTCCTTTTGCTGGATCTACTTTGCTGCTTCCAGACTGAGAGCT
GCAGAGACGGCAGATGGAAAATATGCTCAGAAGTTGTTTAATGACCTTTTT
GAAGATTATTCTAATGCTCTTCGTCCAGTGGAAGATACAGATAAAGTCCTGA
ATGTGACCCTGCAGATTACGCTCTCTCAGATTAAGGATATGGATGAAAGAA
ACCAAATTCTGACTGCTTATTTGTGGATCCGCCAAATCTGGCACGATGCCTA
TCTCACGTGGGACCGAGATCAGTACGATGGCCTAGACTCCATCAGGATCCC
CAGTGACCTCGTGTGGAGGCCAGACATCGTCTTATATAACAAGGCTGATGA
TGAATCTTCAGAGCCTGTGAACACCAATGTGGTCCTGCGGTATGATGGGCT
GATCACCTGGGATGCACCGGCCATCACCAAAAGCTCCTGTGTGGTGGATGT
CACCTACTTCCCTTTTGACAACCAGCAGTGCAACCTGACTTTTGGTTCCTG
GACCTACAATGGCAATCAGGTGGACATATTCAACGCCTTGGACAGCGGAGA
TCTCTCTGACTTCATTGAAGATGTGGAATGGGAGGTCCATGGCATGCCCGC
TGTGAAGAATGTGATCTCCTATGGCTGCTGCTCTGAGCCTTACCCGGATGTC
ACATTCACCCTCCTTCTGAAGAGGAGGTCCTCGTTCTATATCGTCAACCTCC
TCATCCCATGCGTCCTCATATCTTTTCTGGCTCCTCTGAGTTTTTATCTCCCA
GCAGCCTCCGGAGAAAAGGTCTCCCTGGGAGTGACCATCCTGTTGGCCAT
GACTGTATTTCAGCTAATGGTGGCAGAAATCATGCCGGCCTCAGAAAATGT
GCCCCTGATAGGTAAATACTACATAGCCACGATGGCCCTGATCACAGCCTCC
ACTGCGTTGACCATCATGGTGATGAATATCCACTTCTGTGGGGCCGAGGCC
CGGCCGGTGCCACACTGGGCCAGGGTGGTCATCCTGAAATACATGTCCAGG
GTCTTGTTTGTCTATGATGTGGGTGAAAGCTGCCTCAGCCCGCACCACAGT
AGAGAGCGGGACCACCTCACGAAAGTTTATAGCAAACTCCCAGAGTCTAA
CCTGAAAGCAGCCAGGAACAAAGACCTTTCCAGAAAGAAGGACATGAAC
AAACGCTTAAAGAACGACCTGGGCTGCCAGGGTAAGAACCCTCAGGAGGC
CGAGAGTTACTGTGCACAGTACAAAGTGCTGACGAGGAATATTGAGTACAT
CGCCAAGTGCCTCAAAGACCACAAGGCCACCAGTTCCAAGGGGAGTGAAT
GGAAGAAGGTGGCGAAAGTCATAGACCGATTCTTCATGTGGATTTTTTCA

TTATGGTGTTTGTGATGACTATTTTGATCATAGCAAGAGCGGATTAGTCTAG
AGGGCCCGTTTAAACCCGCTGATCAGCCTCGACTGTGCCTTCTAGTTGCCA
GCCATCTGTTGTTTGCCCCTCCCCCGTGCCTTCCTTGACCCTGGAAGGTGC
CACTCCCACTGTCCTTTCCTAATAAAATGAGGAAATTGCATCGCATTGTCTG
AGTAGGTGTCATTCTATTCTGGGGGGTGGGGTGGGGCAGGACAGCAAGGG
GGAGGATTGGGAAGACAATAGCAGGCATGCTGGGGATGCGGTGGGCTCTA
TGGCTTCTGAGGCGGAAAGAACCAGCTGGGGCTCTAGGGGGTATCCCCAC
GCGCCCTGTAGCGGCGCATTAAGCGCGGCGGGTGTGGTGGTTACGCGCAG
CGTGACCGCTACACTTGCCAGCGCCCTAGCGCCCGCTCCTTTCGCTTTCTTC
CCTTCCTTTCTCGCCACGTTCGCCGGCTTTCCCCGTCAAGCTCTAAATCGGG
GGCTCCCTTTAGGGTTCCGATTTAGTGCTTTACGGCACCTCGACCCCAAAA
AACTTGATTAGGGTGATGGTTCACGTAGTGGGCCATCGCCCTGATAGACGG
TTTTTCGCCCTTTGACGTTGGAGTCCACGTTCTTTAATAGTGGACTCTTGTT
CCAAACTGGAACAACACTCAACCCTATCTCGGTCTATTCTTTTGATTTATAA
GGGATTTTGCCGATTTCGGCCTATTGGTTAAAAAATGAGCTGATTTAACAAA
AATTTAACGCGAATTAATTCTGTGGAATGTGTGTCAGTTAGGGTGTGGAAA
GTCCCCAGGCTCCCCAGCAGGCAGAAGTATGCAAAGCATGCATCTCAATTA
GTCAGCAACCAGGTGTGGAAAGTCCCCAGGCTCCCCAGCAGGCAGAAGTA
TGCAAAGCATGCATCTCAATTAGTCAGCAACCATAGTCCCGCCCCTAACTCC
GCCCATCCCGCCCCTAACTCCGCCCAGTTCCGCCCATTCTCCGCCCCATGGC
TGACTAATTTTTTTTATTTATGCAGAGGCCGAGGCCGCCTCTGCCTCTGAGC
TATTCCAGAAGTAGTGAGGAGGCTTTTTTGGAGGCCTAGGCTTTTGCAAAA
AGCTCCCGGGAGCTTGTATATCCATTTTCGGATCTGATCAAGAGACAGGATG
AGGATCGTTTCGCATGATTGAACAAGATGGATTGCACGCAGGTTCTCCGGC
CGCTTGGGTGGAGAGGCTATTCGGCTATGACTGGGCACAACAGACAATCG
GCTGCTCTGATGCCGCCGTGTTCCGGCTGTCAGCGCAGGGGCGCCCGGTTC
TTTTTGTCAAGACCGACCTGTCCGGTGCCCTGAATGAACTGCAGGACGAG
GCAGCGCGGCTATCGTGGCTGGCCACGACGGGCGTTCCTTGCGCAGCTGT
GCTCGACGTTGTCACTGAAGCGGGAAGGGACTGGCTGCTATTGGGCGAAG
TGCCGGGGCAGGATCTCCTGTCATCTCACCTTGCTCCTGCCGAGAAAGTAT
CCATCATGGCTGATGCAATGCGGCGGCTGCATACGCTTGATCCGGCTACCTG
CCCATTCGACCACCAAGCGAAACATCGCATCGAGCGAGCACGTACTCGGAT
GGAAGCCGGTCTTGTCGATCAGGATGATCTGGACGAAGAGCATCAGGGGC
TCGCGCCAGCCGAACTGTTCGCCAGGCTCAAGGCGCGCATGCCCGACGGC
GAGGATCTCGTCGTGACCCATGGCGATGCCTGCTTGCCGAATATCATGGTG
GAAAATGGCCGCTTTTCTGGATTCATCGACTGTGGCCGGCTGGGTGTGGCG
GACCGCTATCAGGACATAGCGTTGGCTACCCGTGATATTGCTGAAGAGCTT
GGCGGCGAATGGGCTGACCGCTTCCTCGTGCTTTACGGTATCGCCGCTCCC

GATTCGCAGCGCATCGCCTTCTATCGCCTTCTTGACGAGTTCTTCTGAGCGG
GACTCTGGGGTTCGAAATGACCGACCAAGCGACGCCCAACCTGCCATCAC
GAGATTTCGATTCCACCGCCGCCTTCTATGAAAGGTTGGGCTTCGGAATCG
TTTTCCGGGACGCCGGCTGGATGATCCTCCAGCGCGGGGATCTCATGCTGG
AGTTCTTCGCCCACCCCAACTTGTTTATTGCAGCTTATAATGGTTACAAATA
AAGCAATAGCATCACAAATTTCACAAATAAAGCATTTTTTTCACTGCATTCT
AGTTGTGGTTTGTCCAAACTCATCAATGTATCTTATCATGTCTGTATACCGTC
GACCTCTAGCTAGAGCTTGGCGTAATCATGGTCATAGCTGTTTCCTGTGTGA
AATTGTTATCCGCTCACAATTCCACACAACATACGAGCCGGAAGCATAAAG
TGTAAAGCCTGGGGTGCCTAATGAGTGAGCTAACTCACATTAATTGCGTTG
CGCTCACTGCCCGCTTTCCAGTCGGGAAACCTGTCGTGCCAGCTGCATTAA
TGAATCGGCCAACGCGCGGGGAGAGGCGGTTTGCGTATTGGGCGCTCTTCC
GCTTCCTCGCTCACTGACTCGCTGCGCTCGGTCGTTCGGCTGCGGCGAGCG
GTATCAGCTCACTCAAAGGCGGTAATACGGTTATCCACAGAATCAGGGGAT
AACGCAGGAAAGAACATGTGAGCAAAAGGCCAGCAAAAGGCCAGGAACC
GTAAAAAGGCCGCGTTGCTGGCGTTTTTCCATAGGCTCCGCCCCCCTGACG
AGCATCACAAAAATCGACGCTCAAGTCAGAGGTGGCGAAACCCGACAGG
ACTATAAAGATACCAGGCGTTTCCCCCTGGAAGCTCCCTCGTGCGCTCTCCT
GTTCCGACCCTGCCGCTTACCGGATACCTGTCCGCCTTTCTCCCTTCGGGAA
GCGTGGCGCTTTCTCATAGCTCACGCTGTAGGTATCTCAGTTCGGTGTAGGT
CGTTCGCTCCAAGCTGGGCTGTGTGCACGAACCCCCCGTTCAGCCCGACC
GCTGCGCCTTATCCGGTAACTATCGTCTTGAGTCCAACCCGGTAAGACACG
ACTTATCGCCACTGGCAGCAGCCACTGGTAACAGGATTAGCAGAGCGAGG
TATGTAGGCGGTGCTACAGAGTTCTTGAAGTGGTGGCCTAACTACGGCTAC
ACTAGAAGAACAGTATTTGGTATCTGCGCTCTGCTGAAGCCAGTTACCTTC
GGAAAAAGAGTTGGTAGCTCTTGATCCGGCAAACAAACCACCGCTGGTAG
CGGTTTTTTTGTTTGCAAGCAGCAGATTACGCGCAGAAAAAAAGGATCTCA
AGAAGATCCTTTGATCTTTTCTACGGGGTCTGACGCTCAGTGGAACGAAAA
CTCACGTTAAGGGATTTTGGTCATGAGATTATCAAAAAGGATCTTCACCTAG
ATCCTTTTAAATTAAAAATGAAGTTTTAAATCAATCTAAAGTATATATGAGTA
AACTTGGTCTGACAGTTACCAATGCTTAATCAGTGAGGCACCTATCTCAGC
GATCTGTCTATTTCGTTCATCCATAGTTGCCTGACTCCCCGTCGTGTAGATAA
CTACGATACGGGAGGGCTTACCATCTGGCCCCAGTGCTGCAATGATACCGC
GAGACCCACGCTCACCGGCTCCAGATTTATCAGCAATAAACCAGCCAGCCG
GAAGGGCCGAGCGCAGAAGTGGTCCTGCAACTTTATCCGCCTCCATCCAGT
CTATTAATTGTTGCCGGGAAGCTAGAGTAAGTAGTTCGCCAGTTAATAGTTT
GCGCAACGTTGTTGCCATTGCTACAGGCATCGTGGTGTCACGCTCGTCGTT
TGGTATGGCTTCATTCAGCTCCGGTTCCCAACGATCAAGGCGAGTTACATG

ATCCCCCATGTTGTGCAAAAAAGCGGTTAGCTCCTTCGGTCCTCCGATCGTT
GTCAGAAGTAAGTTGGCCGCAGTGTTATCACTCATGGTTATGGCAGCACTG
CATAATTCTCTTACTGTCATGCCATCCGTAAGATGCTTTTCTGTGACTGGTGA
GTACTCAACCAAGTCATTCTGAGAATAGTGTATGCGGCGACCGAGTTGCTC
TTGCCCGGCGTCAATACGGGATAATACCGCGCCACATAGCAGAACTTTAAA
AGTGCTCATCATTGGAAAACGTTCTTCGGGGCGAAAACTCTCAAGGATCTT
ACCGCTGTTGAGATCCAGTTCGATGTAACCCACTCGTGCACCCAACTGATC
TTCAGCATCTTTTACTTTCACCAGCGTTTCTGGGTGAGCAAAAACAGGAAG
GCAAAATGCCGCAAAAAAGGGAATAAGGGCGACACGGAAATGTTGAATAC
TCATACTCTTCCTTTTTCAATATTATTGAAGCATTTATCAGGGTTATTGTCTCA
TGAGCGGATACATATTTGAATGTATTTAGAAAAATAAACAAATAGGGGTTCC
GCGCACATTTCCCCGAAAAGTGCCACCTGACGTC

PCDNA3.1 ERBB2-Cluc

PROMOTER

Cluc

Linker

ERBB2

GACGGATCGGGAGATCTCCCGATCCCCTATGGTGCACTCTCAGTACAATCT
GCTCTGATGCCGCATAGTTAAGCCAGTATCTGCTCCCTGCTTGTGTGTTGGA
GGTCGCTGAGTAGTGCGCGAGCAAAATTTAAGCTACAACAAGGCAAGGCT
TGACCGACAATTGCATGAAGAATCTGCTTAGGGTTAGGCGTTTTGCGCTGC
TTCGCGATGTACGGGCCAGATATACGCGTTGACATTGATTATTGACTAGTTAT
TAATAGTAATCAATTACGGGGTCATTAGTTCATAGCCCATATATGGAGTTCCG
CGTTACATAACTTACGGTAAATGGCCCGCCTGGCTGACCGCCCAACGACCC
CCGCCCATTGACGTCAATAATGACGTATGTTCCCATAGTAACGCCAATAGGG
ACTTTCCATTGACGTCAATGGGTGGAGTATTTACGGTAAACTGCCCACTTGG
CAGTACATCAAGTGTATCATATGCCAAGTACGCCCCCTATTGACGTCAATGA
CGGTAAATGGCCCGCCTGGCATTATGCCCAGTACATGACCTTATGGGACTTT
CCTACTTGGCAGTACATCTACGTATTAGTCATCGCTATTACCATGGTGATGCG
GTTTTGGCAGTACATCAATGGGCGTGGATAGCGGTTTGACTCACGGGGATT
TCCAAGTCTCCACCCCATTGACGTCAATGGGAGTTTGTTTTGGCACCAAAA
TCAACGGGACTTTCCAAAATGTCGTAACAACTCCGCCCCATTGACGCAAAT
GGGCGGTAGGCGTGTACGGTGGGAGGTCTATATAAGCAGAGCTCTCTGGCT
AACTAGAGAACCCACTGCTTACTGGCTTATCGAAATTAATACGACTCACTAT
AGGGAGACCCAAGCTGGCTAGCATGAGCGGCTACGTTAACAACCCCGAGG
CTACAAACGCTCTCATCGACAAGGACGGCTGGCTGCACAGCGGCGACATC
GCCTACTGGGACGAGGACGAGCACTTCTTCATCGTGGACCGGCTGAAGAG
CCTGATCAAATACAAGGGCTACCAGGTAGCCCCAGCCGAACTGGAGAGCA
TCCTGCTGCAACACCCCAACATCTTCGACGCCGGGGTCGCCGGCCTGCCCG
ACGACGATGCCGGCGAGCTGCCCGCCGCAGTCGTCGTGCTGGAACACGGT
AAAACCATGACCGAGAAGGAGATCGTGGACTATGTGGCCAGCCAGGTTAC
AACCGCCAAGAAGCTGCGCGGTGGTGTTGTGTTCGTGGACGAGGTGCCTA
AAGGACTGACCGGCAAGTTGGACGCCCGCAAGATCCGCGAGATTCTCATT
AAGGCCAAGAAGGGCGGCAAGATCGCCGTGGCAAGCTTGGTACCGAGCTC
GGATCCACTAGTCCAGTGTGGTGGAATTCTGCAGATATCCAGCACAGTGGC
GGCCGCTCGAGCCATGGAGCTGGCGGCCTTGTGCCGCTGGGGGCTCCTCC
TCGCCCTCTTGCCCCCCGGAGCCGCGAGCACCCAAGTGTGCACCGGCACA
GACATGAAGCTGCGGCTCCCTGCCAGTCCCGAGACCCACCTGGACATGCT
CCGCCACCTCTACCAGGGCTGCCAGGTGGTGCAGGGAAACCTGGAACTCA

CCTACCTGCCCACCAATGCCAGCCTGTCCTTCCTGCAGGATATCCAGGAGG
TGCAGGGCTACGTGCTCATCGCTCACAACCAAGTGAGGCAGGTCCCACTG
CAGAGGCTGCGGATTGTGCGAGGCACCCAGCTCTTTGAGGACAACTATGC
CCTGGCCGTGCTAGACAATGGAGACCCGCTGAACAATACCACCCCTGTCAC
AGGGGCCTCCCCAGGAGGCCTGCGGGAGCTGCAGCTTCGAAGCCTCACAG
AGATCTTGAAAGGAGGGGTCTTGATCCAGCGGAACCCCCAGCTCTGCTACC
AGGACACGATTTTGTGGAAGGACATCTTCCACAAGAACAACCAGCTGGCT
CTCACACTGATAGACACCAACCGCTCTCGGGCCTGCCACCCCTGTTCTCCG
ATGTGTAAGGGCTCCCGCTGCTGGGGAGAGAGTTCTGAGGATTGTCAGAG
CCTGACGCGCACTGTCTGTGCCGGTGGCTGTGCCCGCTGCAAGGGGCCAC
TGCCCACTGACTGCTGCCATGAGCAGTGTGCTGCCGGCTGCACGGGCCCC
AAGCACTCTGACTGCCTGGCCTGCCTCCACTTCAACCACAGTGGCATCTGT
GAGCTGCACTGCCCAGCCCTGGTCACCTACAACACAGACACGTTTGAGTC
CATGCCCAATCCCGAGGGCCGGTATACATTCGGCGCCAGCTGTGTGACTGC
CTGTCCCTACAACTACCTTTCTACGGACGTGGGATCCTGCACCCTCGTCTGC
CCCCTGCACAACCAAGAGGTGACAGCAGAGGATGGAACACAGCGGTGTG
AGAAGTGCAGCAAGCCCTGTGCCCGAGTGTGCTATGGTCTGGGCATGGAG
CACTTGCGAGAGGTGAGGGCAGTTACCAGTGCCAATATCCAGGAGTTTGCT
GGCTGCAAGAAGATCTTTGGGAGCCTGGCATTTCTGCCGGAGAGCTTTGAT
GGGGACCCAGCCTCCAACACTGCCCCGCTCCAGCCAGAGCAGCTCCAAGT
GTTTGAGACTCTGGAAGAGATCACAGGTTACCTATACATCTCAGCATGGCC
GGACAGCCTGCCTGACCTCAGCGTCTTCCAGAACCTGCAAGTAATCCGGG
GACGAATTCTGCACAATGGCGCCTACTCGCTGACCCTGCAAGGGCTGGGC
ATCAGCTGGCTGGGGCTGCGCTCACTGAGGGAACTGGGCAGTGGACTGGC
CCTCATCCACCATAACACCCACCTCTGCTTCGTGCACACGGTGCCCTGGGA
CCAGCTCTTTCGGAACCCGCACCAAGCTCTGCTCCACACTGCCAACCGGC
CAGAGGACGAGTGTGTGGGCGAGGGCCTGGCCTGCCACCAGCTGTGCGCC
CGAGGGCACTGCTGGGGTCCAGGGCCCACCCAGTGTGTCAACTGCAGCCA
GTTCCTTCGGGGCCAGGAGTGCGTGGAGGAATGCCGAGTACTGCAGGGGC
TCCCCAGGGAGTATGTGAATGCCAGGCACTGTTTGCCGTGCCACCCTGAGT
GTCAGCCCCAGAATGGCTCAGTGACCTGTTTTGGACCGGAGGCTGACCAG
TGTGTGGCCTGTGCCCACTATAAGGACCCTCCCTTCTGCGTGGCCCGCTGC
CCCAGCGGTGTGAAACCTGACCTCTCCTACATGCCCATCTGGAAGTTTCCA
GATGAGGAGGGCGCATGCCAGCCTTGCCCCATCAACTGCACCCACTCCTGT
GTGGACCTGGATGACAAGGGCTGCCCCGCCGAGCAGAGAGCCAGCCCTCT
GACGTCCATCATCTCTGCGGTGGTTGGCATTCTGCTGGTCGTGGTCTTGGG
GGTGGTCTTTGGGATCCTCATCAAGCGACGGCAGCAGAAGATCCGGAAGT
ACACGATGCGGAGACTGCTGCAGGAAACGGAGCTGGTGGAGCCGCTGAC

ACCTAGCGGAGCGATGCCCAACCAGGCGCAGATGCGGATCCTGAAAGAGA
CGGAGCTGAGGAAGGTGAAGGTGCTTGGATCTGGCGCTTTTGGCACAGTC
TACAAGGGCATCTGGATCCCTGATGGGGAGAATGTGAAAATTCCAGTGGCC
ATCAAAGTGTTGAGGGAAAACACATCCCCCAAAGCCAACAAAGAAATCTT
AGACGAAGCATACGTGATGGCTGGTGTGGGCTCCCCATATGTCTCCCGCCT
TCTGGGCATCTGCCTGACATCCACGGTGCAGCTGGTGACACAGCTTATGCC
CTATGGCTGCCTCTTAGACCATGTCCGGGAAAACCGCGGACGCCTGGGCTC
CCAGGACCTGCTGAACTGGTGTATGCAGATTGCCAAGGGGATGAGCTACCT
GGAGGATGTGCGGCTCGTACACAGGGACTTGGCCGCTCGGAACGTGCTGG
TCAAGAGTCCCAACCATGTCAAAATTACAGACTTCGGGCTGGCTCGGCTGC
TGGACATTGACGAGACAGAGTACCATGCAGATGGGGGCAAGGTGCCCATC
AAGTGGATGGCGCTGGAGTCCATTCTCCGCCGGCGGTTCACCCACCAGAG
TGATGTGTGGAGTTATGGTGTGACTGTGTGGGAGCTGATGACTTTTGGGGC
CAAACCTTACGATGGGATCCCAGCCCGGGAGATCCCTGACCTGCTGGAAA
AGGGGGAGCGGCTGCCCCAGCCCCCCATCTGCACCATTGATGTCTACATGA
TCATGGTCAAATGTTGGATGATTGACTCTGAATGTCGGCCAAGATTCCGGG
AGTTGGTGTCTGAATTCTCCCGCATGGCCAGGGACCCCCAGCGCTTTGTGG
TCATCCAGAATGAGGACTTGGGCCCAGCCAGTCCCTTGGACAGCACCTTCT
ACCGCTCACTGCTGGAGGACGATGACATGGGGGACCTGGTGGATGCTGAG
GAGTATCTGGTACCCCAGCAGGGCTTCTTCTGTCCAGACCCTGCCCCGGGC
GCTGGGGGCATGGTCCACCACAGGCACCGCAGCTCATCTACCAGGAGTGG
CGGTGGGGACCTGACACTAGGGCTGGAGCCCTCTGAAGAGGAGGCCCCCA
GGTCTCCACTGGCACCCTCCGAAGGGGCTGGCTCCGATGTATTTGATGGTG
ACCTGGGAATGGGGGCAGCCAAGGGGCTGCAAAGCCTCCCCACACATGAC
CCCAGCCCTCTACAGCGGTACAGTGAGGACCCCACAGTACCCCTGCCCTCT
GAGACTGATGGCTACGTTGCCCCCCTGACCTGCAGCCCCCAGCCTGAATAT
GTGAACCAGCCAGATGTTCGGCCCCAGCCCCCTTCGCCCCGAGAGGGCCC
TCTGCCTGCTGCCCGACCTGCTGGTGCCACTCTGGAAAGGCCCAAGACTCT
CTCCCCAGGGAAGAATGGGGTCGTCAAAGACGTTTTTGCCTTTGGGGGTG
CCGTGGAGAACCCCGAGTACTTGACACCCCAGGGAGGAGCTGCCCCTCAG
CCCCACCCTCCTCCTGCCTTCAGCCCAGCCTTCGACAACCTCTATTACTGGG
ACCAGGACCCACCAGAGCGGGGGGCTCCACCCAGCACCTTCAAAGGGAC
ACCTACGGCAGAGAACCCAGAGTACCTGGGTCTGGACGTGCCAGTGTGAT
CTAGAGGGCCCGTTTAAACCCGCTGATCAGCCTCGACTGTGCCTTCTAGTT
GCCAGCCATCTGTTGTTTGCCCCTCCCCCGTGCCTTCCTTGACCCTGGAAG
GTGCCACTCCCACTGTCCTTTCCTAATAAAATGAGGAAATTGCATCGCATTG
TCTGAGTAGGTGTCATTCTATTCTGGGGGGTGGGGTGGGGCAGGACAGCA
AGGGGGAGGATTGGGAAGACAATAGCAGGCATGCTGGGGATGCGGTGGGC

TCTATGGCTTCTGAGGCGGAAAGAACCAGCTGGGGCTCTAGGGGGTATCCC
CACGCGCCCTGTAGCGGCGCATTAAGCGCGGCGGGTGTGGTGGTTACGCG
CAGCGTGACCGCTACACTTGCCAGCGCCCTAGCGCCCGCTCCTTTCGCTTT
CTTCCCTTCCTTTCTCGCCACGTTCGCCGGCTTTCCCCGTCAAGCTCTAAAT
CGGGGGCTCCCTTTAGGGTTCCGATTTAGTGCTTTACGGCACCTCGACCCC
AAAAAACTTGATTAGGGTGATGGTTCACGTAGTGGGCCATCGCCCTGATAG
ACGGTTTTTCGCCCTTTGACGTTGGAGTCCACGTTCTTTAATAGTGGACTCT
TGTTCCAAACTGGAACAACACTCAACCCTATCTCGGTCTATTCTTTTGATTT
ATAAGGGATTTTGCCGATTTCGGCCTATTGGTTAAAAAATGAGCTGATTTAA
CAAAAATTTAACGCGAATTAATTCTGTGGAATGTGTGTCAGTTAGGGTGTG
GAAAGTCCCCAGGCTCCCCAGCAGGCAGAAGTATGCAAAGCATGCATCTC
AATTAGTCAGCAACCAGGTGTGGAAAGTCCCCAGGCTCCCCAGCAGGCAG
AAGTATGCAAAGCATGCATCTCAATTAGTCAGCAACCATAGTCCCGCCCCTA
ACTCCGCCCATCCCGCCCCTAACTCCGCCCAGTTCCGCCCATTCTCCGCCCC
ATGGCTGACTAATTTTTTTTATTTATGCAGAGGCCGAGGCCGCCTCTGCCTC
TGAGCTATTCCAGAAGTAGTGAGGAGGCTTTTTTGGAGGCCTAGGCTTTTG
CAAAAAGCTCCCGGGAGCTTGTATATCCATTTTCGGATCTGATCAAGAGAC
AGGATGAGGATCGTTTCGCATGATTGAACAAGATGGATTGCACGCAGGTTC
TCCGGCCGCTTGGGTGGAGAGGCTATTCGGCTATGACTGGGCACAACAGA
CAATCGGCTGCTCTGATGCCGCCGTGTTCCGGCTGTCAGCGCAGGGGCGCC
CGGTTCTTTTTGTCAAGACCGACCTGTCCGGTGCCCTGAATGAACTGCAGG
ACGAGGCAGCGCGGCTATCGTGGCTGGCCACGACGGGCGTTCCTTGCGCA
GCTGTGCTCGACGTTGTCACTGAAGCGGGAAGGGACTGGCTGCTATTGGG
CGAAGTGCCGGGGCAGGATCTCCTGTCATCTCACCTTGCTCCTGCCGAGAA
AGTATCCATCATGGCTGATGCAATGCGGCGGCTGCATACGCTTGATCCGGCT
ACCTGCCCATTCGACCACCAAGCGAAACATCGCATCGAGCGAGCACGTACT
CGGATGGAAGCCGGTCTTGTCGATCAGGATGATCTGGACGAAGAGCATCA
GGGGCTCGCGCCAGCCGAACTGTTCGCCAGGCTCAAGGCGCGCATGCCCG
ACGGCGAGGATCTCGTCGTGACCCATGGCGATGCCTGCTTGCCGAATATCA
TGGTGGAAAATGGCCGCTTTTCTGGATTCATCGACTGTGGCCGGCTGGGTG
TGGCGGACCGCTATCAGGACATAGCGTTGGCTACCCGTGATATTGCTGAAG
AGCTTGGCGGCGAATGGGCTGACCGCTTCCTCGTGCTTTACGGTATCGCCG
CTCCCGATTCGCAGCGCATCGCCTTCTATCGCCTTCTTGACGAGTTCTTCTG
AGCGGGACTCTGGGGTTCGAAATGACCGACCAAGCGACGCCCAACCTGCC
ATCACGAGATTTCGATTCCACCGCCGCCTTCTATGAAAGGTTGGGCTTCGG
AATCGTTTTCCGGGACGCCGGCTGGATGATCCTCCAGCGCGGGGATCTCAT
GCTGGAGTTCTTCGCCCACCCCAACTTGTTTATTGCAGCTTATAATGGTTAC
AAATAAAGCAATAGCATCACAAATTTCACAAATAAAGCATTTTTTTCACTGC

ATTCTAGTTGTGGTTTGTCCAAACTCATCAATGTATCTTATCATGTCTGTATA
CCGTCGACCTCTAGCTAGAGCTTGGCGTAATCATGGTCATAGCTGTTTCCTG
TGTGAAATTGTTATCCGCTCACAATTCCACACAACATACGAGCCGGAAGCA
TAAAGTGTAAAGCCTGGGGTGCCTAATGAGTGAGCTAACTCACATTAATTG
CGTTGCGCTCACTGCCCGCTTTCCAGTCGGGAAACCTGTCGTGCCAGCTGC
ATTAATGAATCGGCCAACGCGCGGGGAGAGGCGGTTTGCGTATTGGGCGCT
CTTCCGCTTCCTCGCTCACTGACTCGCTGCGCTCGGTCGTTCGGCTGCGGC
GAGCGGTATCAGCTCACTCAAAGGCGGTAATACGGTTATCCACAGAATCAG
GGGATAACGCAGGAAAGAACATGTGAGCAAAAGGCCAGCAAAAGGCCAG
GAACCGTAAAAAGGCCGCGTTGCTGGCGTTTTTCCATAGGCTCCGCCCCCC
TGACGAGCATCACAAAAATCGACGCTCAAGTCAGAGGTGGCGAAACCCGA
CAGGACTATAAAGATACCAGGCGTTTCCCCCTGGAAGCTCCCTCGTGCGCT
CTCCTGTTCCGACCCTGCCGCTTACCGGATACCTGTCCGCCTTTCTCCCTTC
GGGAAGCGTGGCGCTTTCTCATAGCTCACGCTGTAGGTATCTCAGTTCGGT
GTAGGTCGTTCGCTCCAAGCTGGGCTGTGTGCACGAACCCCCCGTTCAGCC
CGACCGCTGCGCCTTATCCGGTAACTATCGTCTTGAGTCCAACCCGGTAAG
ACACGACTTATCGCCACTGGCAGCAGCCACTGGTAACAGGATTAGCAGAG
CGAGGTATGTAGGCGGTGCTACAGAGTTCTTGAAGTGGTGGCCTAACTACG
GCTACACTAGAAGAACAGTATTTGGTATCTGCGCTCTGCTGAAGCCAGTTA
CCTTCGGAAAAAGAGTTGGTAGCTCTTGATCCGGCAAACAAACCACCGCT
GGTAGCGGTTTTTTTGTTTGCAAGCAGCAGATTACGCGCAGAAAAAAAGG
ATCTCAAGAAGATCCTTTGATCTTTTCTACGGGGTCTGACGCTCAGTGGAA
CGAAAACTCACGTTAAGGGATTTTGGTCATGAGATTATCAAAAAGGATCTT
CACCTAGATCCTTTTAAATTAAAAATGAAGTTTTAAATCAATCTAAAGTATAT
ATGAGTAAACTTGGTCTGACAGTTACCAATGCTTAATCAGTGAGGCACCTAT
CTCAGCGATCTGTCTATTTCGTTCATCCATAGTTGCCTGACTCCCCGTCGTG
TAGATAACTACGATACGGGAGGGCTTACCATCTGGCCCCAGTGCTGCAATG
ATACCGCGAGACCCACGCTCACCGGCTCCAGATTTATCAGCAATAAACCAG
CCAGCCGGAAGGGCCGAGCGCAGAAGTGGTCCTGCAACTTTATCCGCCTC
CATCCAGTCTATTAATTGTTGCCGGGAAGCTAGAGTAAGTAGTTCGCCAGTT
AATAGTTTGCGCAACGTTGTTGCCATTGCTACAGGCATCGTGGTGTCACGC
TCGTCGTTTGGTATGGCTTCATTCAGCTCCGGTTCCCAACGATCAAGGCGA
GTTACATGATCCCCCATGTTGTGCAAAAAAGCGGTTAGCTCCTTCGGTCCTC
CGATCGTTGTCAGAAGTAAGTTGGCCGCAGTGTTATCACTCATGGTTATGGC
AGCACTGCATAATTCTCTTACTGTCATGCCATCCGTAAGATGCTTTTCTGTG
ACTGGTGAGTACTCAACCAAGTCATTCTGAGAATAGTGTATGCGGCGACCG
AGTTGCTCTTGCCCGGCGTCAATACGGGATAATACCGCGCCACATAGCAGA
ACTTTAAAAGTGCTCATCATTGGAAAACGTTCTTCGGGGCGAAAACTCTCA

AGGATCTTACCGCTGTTGAGATCCAGTTCGATGTAACCCACTCGTGCACCC
AACTGATCTTCAGCATCTTTTACTTTCACCAGCGTTTCTGGGTGAGCAAAA
ACAGGAAGGCAAAATGCCGCAAAAAAGGGAATAAGGGCGACACGGAAAT
GTTGAATACTCATACTCTTCCTTTTTCAATATTATTGAAGCATTTATCAGGGT
TATTGTCTCATGAGCGGATACATATTTGAATGTATTTAGAAAAATAAACAAAT
AGGGGTTCCGCGCACATTTCCCCGAAAAGTGCCACCTGACGTC

PCDNA3.1 ERBB2-Nluc

PROMOTER

Nluc

Linker

ERBB2

GACGGATCGGGAGATCTCCCGATCCCCTATGGTGCACTCTCAGTACAATCT
GCTCTGATGCCGCATAGTTAAGCCAGTATCTGCTCCCTGCTTGTGTGTTGGA
GGTCGCTGAGTAGTGCGCGAGCAAAATTTAAGCTACAACAAGGCAAGGCT
TGACCGACAATTGCATGAAGAATCTGCTTAGGGTTAGGCGTTTTGCGCTGC
TTCGCGATGTACGGGCCAGATATACGCGTTGACATTGATTATTGACTAGTTAT
TAATAGTAATCAATTACGGGGTCATTAGTTCATAGCCCATATATGGAGTTCCG
CGTTACATAACTTACGGTAAATGGCCCGCCTGGCTGACCGCCCAACGACCC
CCGCCCATTGACGTCAATAATGACGTATGTTCCCATAGTAACGCCAATAGGG
ACTTTCCATTGACGTCAATGGGTGGAGTATTTACGGTAAACTGCCCACTTGG
CAGTACATCAAGTGTATCATATGCCAAGTACGCCCCCTATTGACGTCAATGA
CGGTAAATGGCCCGCCTGGCATTATGCCCAGTACATGACCTTATGGGACTTT
CCTACTTGGCAGTACATCTACGTATTAGTCATCGCTATTACCATGGTGATGCG
GTTTTGGCAGTACATCAATGGGCGTGGATAGCGGTTTGACTCACGGGGATT
TCCAAGTCTCCACCCCATTGACGTCAATGGGAGTTTGTTTTGGCACCAAAA
TCAACGGGACTTTCCAAAATGTCGTAACAACTCCGCCCCATTGACGCAAAT
GGGCGGTAGGCGTGTACGGTGGGAGGTCTATATAAGCAGAGCTCTCTGGCT
AACTAGAGAACCCACTGCTTACTGGCTTATCGAAATTAATACGACTCACTAT
AGGGAGACCCAAGCTGGCTAGCATGGAAGATGCCAAAAACATTAAGAAGG
GCCCAGCGCCATTCTACCCACTCGAAGACGGGACCGCCGGCGAGCAGCTG
CACAAAGCCATGAAGCGCTACGCCCTGGTGCCCGGCACCATCGCCTTTACC
GACGCACATATCGAGGTGGACATTACCTACGCCGAGTACTTCGAGATGAGC
GTTCGGCTGGCAGAAGCTATGAAGCGCTATGGGCTGAATACAAACCATCGG
ATCGTGGTGTGCAGCGAGAATAGCTTGCAGTTCTTCATGCCCGTGTTGGGT
GCCCTGTTCATCGGTGTGGCTGTGGCCCCAGCTAACGACATCTACAACGAG
CGCGAGCTGCTGAACAGCATGGGCATCAGCCAGCCCACCGTCGTATTCGTG
AGCAAGAAAGGGCTGCAAAAGATCCTCAACGTGCAAAAGAAGCTACCGAT
CATACAAAAGATCATCATCATGGATAGCAAGACCGACTACCAGGGCTTCCA
AAGCATGTACACCTTCGTGACTTCCCATTTGCCACCCGGCTTCAACGAGTA
CGACTTCGTGCCCGAGAGCTTCGACCGGGACAAAACCATCGCCCTGATCAT
GAACAGTAGTGGCAGTACCGGATTGCCCAAGGGCGTAGCCCTACCGCACC
GCACCGCTTGTGTCCGATTCAGTCATGCCCGCGACCCCATCTTCGGCAACC
AGATCATCCCCGACACCGCTATCCTCAGCGTGGTGCCATTTCACCACGGCTT
CGGCATGTTCACCACGCTGGGCTACTTGATCTGCGGCTTTCGGGTCGTGCT

CATGTACCGCTTCGAGGAGGAGCTATTCTTGCGCAGCTTGCAAGACTATAA
GATTCAATCTGCCCTGCTGGTGCCCACACTATTTAGCTTCTTCGCTAAGAGC
ACTCTCATCGACAAGTACGACCTAAGCAACTTGCACGAGATCGCCAGCGG
CGGGGCGCCGCTCAGCAAGGAGGTAGGTGAGGCCGTGGCCAAACGCTTCC
ACCTACCAGGCATCCGCCAGGGCTACGGCCTGACAGAAACAACCAGCGCC
ATTCTGATCACCCCCGAAGGGGACGACAAGCCTGGCGCAGTAGGCAAGGT
GGTGCCCTTCTTCGAGGCTAAGGTGGTGGACTTGGACACCGGTAAGACAC
TGGGTGTGAACCAGCGCGGCGAGCTGTGCGTCCGTGGCCCCATGATCATG
AGCGGCTACGTTAACAACCCCGAGGCTACAAACGCTCTCATCGACAAGGA
CGGCGGAAGCTTGGTACCGAGCTCGGATCCACTAGTCCAGTGTGGTGGAAT
TCTGCAGATATCCAGCACAGTGGCGGCCGCTCGAGCCATGGAGCTGGCGG
CCTTGTGCCGCTGGGGGCTCCTCCTCGCCCTCTTGCCCCCCGGAGCCGCGA
GCACCCAAGTGTGCACCGGCACAGACATGAAGCTGCGGCTCCCTGCCAGT
CCCGAGACCCACCTGGACATGCTCCGCCACCTCTACCAGGGCTGCCAGGT
GGTGCAGGGAAACCTGGAACTCACCTACCTGCCCACCAATGCCAGCCTGT
CCTTCCTGCAGGATATCCAGGAGGTGCAGGGCTACGTGCTCATCGCTCACA
ACCAAGTGAGGCAGGTCCCACTGCAGAGGCTGCGGATTGTGCGAGGCACC
CAGCTCTTTGAGGACAACTATGCCCTGGCCGTGCTAGACAATGGAGACCCG
CTGAACAATACCACCCCTGTCACAGGGGCCTCCCCAGGAGGCCTGCGGGA
GCTGCAGCTTCGAAGCCTCACAGAGATCTTGAAAGGAGGGGTCTTGATCC
AGCGGAACCCCCAGCTCTGCTACCAGGACACGATTTTGTGGAAGGACATCT
TCCACAAGAACAACCAGCTGGCTCTCACACTGATAGACACCAACCGCTCT
CGGGCCTGCCACCCCTGTTCTCCGATGTGTAAGGGCTCCCGCTGCTGGGGA
GAGAGTTCTGAGGATTGTCAGAGCCTGACGCGCACTGTCTGTGCCGGTGG
CTGTGCCCGCTGCAAGGGGCCACTGCCCACTGACTGCTGCCATGAGCAGT
GTGCTGCCGGCTGCACGGGCCCCAAGCACTCTGACTGCCTGGCCTGCCTC
CACTTCAACCACAGTGGCATCTGTGAGCTGCACTGCCCAGCCCTGGTCACC
TACAACACAGACACGTTTGAGTCCATGCCCAATCCCGAGGGCCGGTATACA
TTCGGCGCCAGCTGTGTGACTGCCTGTCCCTACAACTACCTTTCTACGGAC
GTGGGATCCTGCACCCTCGTCTGCCCCCTGCACAACCAAGAGGTGACAGC
AGAGGATGGAACACAGCGGTGTGAGAAGTGCAGCAAGCCCTGTGCCCGA
GTGTGCTATGGTCTGGGCATGGAGCACTTGCGAGAGGTGAGGGCAGTTAC
CAGTGCCAATATCCAGGAGTTTGCTGGCTGCAAGAAGATCTTTGGGAGCCT
GGCATTTCTGCCGGAGAGCTTTGATGGGGACCCAGCCTCCAACACTGCCCC
GCTCCAGCCAGAGCAGCTCCAAGTGTTTGAGACTCTGGAAGAGATCACAG
GTTACCTATACATCTCAGCATGGCCGGACAGCCTGCCTGACCTCAGCGTCTT
CCAGAACCTGCAAGTAATCCGGGGACGAATTCTGCACAATGGCGCCTACTC
GCTGACCCTGCAAGGGCTGGGCATCAGCTGGCTGGGGCTGCGCTCACTGA

GGGAACTGGGCAGTGGACTGGCCCTCATCCACCATAACACCCACCTCTGCT
TCGTGCACACGGTGCCCTGGGACCAGCTCTTTCGGAACCCGCACCAAGCT
CTGCTCCACACTGCCAACCGGCCAGAGGACGAGTGTGTGGGCGAGGGCCT
GGCCTGCCACCAGCTGTGCGCCCGAGGGCACTGCTGGGGTCCAGGGCCCA
CCCAGTGTGTCAACTGCAGCCAGTTCCTTCGGGGCCAGGAGTGCGTGGAG
GAATGCCGAGTACTGCAGGGGCTCCCCAGGGAGTATGTGAATGCCAGGCA
CTGTTTGCCGTGCCACCCTGAGTGTCAGCCCCAGAATGGCTCAGTGACCTG
TTTTGGACCGGAGGCTGACCAGTGTGTGGCCTGTGCCCACTATAAGGACCC
TCCCTTCTGCGTGGCCCGCTGCCCCAGCGGTGTGAAACCTGACCTCTCCTA
CATGCCCATCTGGAAGTTTCCAGATGAGGAGGGCGCATGCCAGCCTTGCCC
CATCAACTGCACCCACTCCTGTGTGGACCTGGATGACAAGGGCTGCCCCGC
CGAGCAGAGAGCCAGCCCTCTGACGTCCATCATCTCTGCGGTGGTTGGCAT
TCTGCTGGTCGTGGTCTTGGGGGTGGTCTTTGGGATCCTCATCAAGCGACG
GCAGCAGAAGATCCGGAAGTACACGATGCGGAGACTGCTGCAGGAAACG
GAGCTGGTGGAGCCGCTGACACCTAGCGGAGCGATGCCCAACCAGGCGCA
GATGCGGATCCTGAAAGAGACGGAGCTGAGGAAGGTGAAGGTGCTTGGAT
CTGGCGCTTTTGGCACAGTCTACAAGGGCATCTGGATCCCTGATGGGGAGA
ATGTGAAAATTCCAGTGGCCATCAAAGTGTTGAGGGAAAACACATCCCCC
AAAGCCAACAAAGAAATCTTAGACGAAGCATACGTGATGGCTGGTGTGGG
CTCCCCATATGTCTCCCGCCTTCTGGGCATCTGCCTGACATCCACGGTGCAG
CTGGTGACACAGCTTATGCCCTATGGCTGCCTCTTAGACCATGTCCGGGAA
AACCGCGGACGCCTGGGCTCCCAGGACCTGCTGAACTGGTGTATGCAGATT
GCCAAGGGGATGAGCTACCTGGAGGATGTGCGGCTCGTACACAGGGACTT
GGCCGCTCGGAACGTGCTGGTCAAGAGTCCCAACCATGTCAAAATTACAG
ACTTCGGGCTGGCTCGGCTGCTGGACATTGACGAGACAGAGTACCATGCA
GATGGGGGCAAGGTGCCCATCAAGTGGATGGCGCTGGAGTCCATTCTCCGC
CGGCGGTTCACCCACCAGAGTGATGTGTGGAGTTATGGTGTGACTGTGTGG
GAGCTGATGACTTTTGGGGCCAAACCTTACGATGGGATCCCAGCCCGGGAG
ATCCCTGACCTGCTGGAAAAGGGGGAGCGGCTGCCCCAGCCCCCCATCTG
CACCATTGATGTCTACATGATCATGGTCAAATGTTGGATGATTGACTCTGAA
TGTCGGCCAAGATTCCGGGAGTTGGTGTCTGAATTCTCCCGCATGGCCAGG
GACCCCCAGCGCTTTGTGGTCATCCAGAATGAGGACTTGGGCCCAGCCAG
TCCCTTGGACAGCACCTTCTACCGCTCACTGCTGGAGGACGATGACATGGG
GGACCTGGTGGATGCTGAGGAGTATCTGGTACCCCAGCAGGGCTTCTTCTG
TCCAGACCCTGCCCCGGGCGCTGGGGGCATGGTCCACCACAGGCACCGCA
GCTCATCTACCAGGAGTGGCGGTGGGGACCTGACACTAGGGCTGGAGCCC
TCTGAAGAGGAGGCCCCCAGGTCTCCACTGGCACCCTCCGAAGGGGCTGG
CTCCGATGTATTTGATGGTGACCTGGGAATGGGGGCAGCCAAGGGGCTGCA

AAGCCTCCCCACACATGACCCCAGCCCTCTACAGCGGTACAGTGAGGACC
CCACAGTACCCCTGCCCTCTGAGACTGATGGCTACGTTGCCCCCCTGACCT
GCAGCCCCCAGCCTGAATATGTGAACCAGCCAGATGTTCGGCCCCAGCCCC
CTTCGCCCCGAGAGGGCCCTCTGCCTGCTGCCCGACCTGCTGGTGCCACTC
TGGAAAGGCCCAAGACTCTCTCCCCAGGGAAGAATGGGGTCGTCAAAGAC
GTTTTTGCCTTTGGGGGTGCCGTGGAGAACCCCGAGTACTTGACACCCCAG
GGAGGAGCTGCCCCTCAGCCCCACCCTCCTCCTGCCTTCAGCCCAGCCTTC
GACAACCTCTATTACTGGGACCAGGACCCACCAGAGCGGGGGGCTCCACC
CAGCACCTTCAAAGGGACACCTACGGCAGAGAACCCAGAGTACCTGGGTC
TGGACGTGCCAGTGTGATCTAGAGGGCCCGTTTAAACCCGCTGATCAGCCT
CGACTGTGCCTTCTAGTTGCCAGCCATCTGTTGTTTGCCCCTCCCCCGTGCC
TTCCTTGACCCTGGAAGGTGCCACTCCCACTGTCCTTTCCTAATAAAATGA
GGAAATTGCATCGCATTGTCTGAGTAGGTGTCATTCTATTCTGGGGGGTGGG
GTGGGGCAGGACAGCAAGGGGGAGGATTGGGAAGACAATAGCAGGCATG
CTGGGGATGCGGTGGGCTCTATGGCTTCTGAGGCGGAAAGAACCAGCTGG
GGCTCTAGGGGGTATCCCCACGCGCCCTGTAGCGGCGCATTAAGCGCGGCG
GGTGTGGTGGTTACGCGCAGCGTGACCGCTACACTTGCCAGCGCCCTAGCG
CCCGCTCCTTTCGCTTTCTTCCCTTCCTTTCTCGCCACGTTCGCCGGCTTTC
CCCGTCAAGCTCTAAATCGGGGGCTCCCTTTAGGGTTCCGATTTAGTGCTTT
ACGGCACCTCGACCCCAAAAAACTTGATTAGGGTGATGGTTCACGTAGTGG
GCCATCGCCCTGATAGACGGTTTTTCGCCCTTTGACGTTGGAGTCCACGTTC
TTTAATAGTGGACTCTTGTTCCAAACTGGAACAACACTCAACCCTATCTCG
GTCTATTCTTTTGATTTATAAGGGATTTTGCCGATTTCGGCCTATTGGTTAAA
AAATGAGCTGATTTAACAAAAATTTAACGCGAATTAATTCTGTGGAATGTGT
GTCAGTTAGGGTGTGGAAAGTCCCCAGGCTCCCCAGCAGGCAGAAGTATG
CAAAGCATGCATCTCAATTAGTCAGCAACCAGGTGTGGAAAGTCCCCAGG
CTCCCCAGCAGGCAGAAGTATGCAAAGCATGCATCTCAATTAGTCAGCAAC
CATAGTCCCGCCCCTAACTCCGCCCATCCCGCCCCTAACTCCGCCCAGTTCC
GCCCATTCTCCGCCCCATGGCTGACTAATTTTTTTTATTTATGCAGAGGCCG
AGGCCGCCTCTGCCTCTGAGCTATTCCAGAAGTAGTGAGGAGGCTTTTTTG
GAGGCCTAGGCTTTTGCAAAAAGCTCCCGGGAGCTTGTATATCCATTTTCG
GATCTGATCAAGAGACAGGATGAGGATCGTTTCGCATGATTGAACAAGATG
GATTGCACGCAGGTTCTCCGGCCGCTTGGGTGGAGAGGCTATTCGGCTATG
ACTGGGCACAACAGACAATCGGCTGCTCTGATGCCGCCGTGTTCCGGCTGT
CAGCGCAGGGGCGCCCGGTTCTTTTTGTCAAGACCGACCTGTCCGGTGCC
CTGAATGAACTGCAGGACGAGGCAGCGCGGCTATCGTGGCTGGCCACGAC
GGGCGTTCCTTGCGCAGCTGTGCTCGACGTTGTCACTGAAGCGGGAAGGG
ACTGGCTGCTATTGGGCGAAGTGCCGGGGCAGGATCTCCTGTCATCTCACC

TTGCTCCTGCCGAGAAAGTATCCATCATGGCTGATGCAATGCGGCGGCTGC
ATACGCTTGATCCGGCTACCTGCCCATTCGACCACCAAGCGAAACATCGCA
TCGAGCGAGCACGTACTCGGATGGAAGCCGGTCTTGTCGATCAGGATGATC
TGGACGAAGAGCATCAGGGGCTCGCGCCAGCCGAACTGTTCGCCAGGCTC
AAGGCGCGCATGCCCGACGGCGAGGATCTCGTCGTGACCCATGGCGATGC
CTGCTTGCCGAATATCATGGTGGAAAATGGCCGCTTTTCTGGATTCATCGAC
TGTGGCCGGCTGGGTGTGGCGGACCGCTATCAGGACATAGCGTTGGCTACC
CGTGATATTGCTGAAGAGCTTGGCGGCGAATGGGCTGACCGCTTCCTCGTG
CTTTACGGTATCGCCGCTCCCGATTCGCAGCGCATCGCCTTCTATCGCCTTC
TTGACGAGTTCTTCTGAGCGGGACTCTGGGGTTCGAAATGACCGACCAAG
CGACGCCCAACCTGCCATCACGAGATTTCGATTCCACCGCCGCCTTCTATG
AAAGGTTGGGCTTCGGAATCGTTTTCCGGGACGCCGGCTGGATGATCCTCC
AGCGCGGGGATCTCATGCTGGAGTTCTTCGCCCACCCCAACTTGTTTATTGC
AGCTTATAATGGTTACAAATAAAGCAATAGCATCACAAATTTCACAAATAAA
GCATTTTTTTCACTGCATTCTAGTTGTGGTTTGTCCAAACTCATCAATGTATC
TTATCATGTCTGTATACCGTCGACCTCTAGCTAGAGCTTGGCGTAATCATGGT
CATAGCTGTTTCCTGTGTGAAATTGTTATCCGCTCACAATTCCACACAACAT
ACGAGCCGGAAGCATAAAGTGTAAAGCCTGGGGTGCCTAATGAGTGAGCT
AACTCACATTAATTGCGTTGCGCTCACTGCCCGCTTTCCAGTCGGGAAACC
TGTCGTGCCAGCTGCATTAATGAATCGGCCAACGCGCGGGGAGAGGCGGT
TTGCGTATTGGGCGCTCTTCCGCTTCCTCGCTCACTGACTCGCTGCGCTCGG
TCGTTCGGCTGCGGCGAGCGGTATCAGCTCACTCAAAGGCGGTAATACGGT
TATCCACAGAATCAGGGGATAACGCAGGAAAGAACATGTGAGCAAAAGGC
CAGCAAAAGGCCAGGAACCGTAAAAAGGCCGCGTTGCTGGCGTTTTTCCA
TAGGCTCCGCCCCCCTGACGAGCATCACAAAAATCGACGCTCAAGTCAGA
GGTGGCGAAACCCGACAGGACTATAAAGATACCAGGCGTTTCCCCCTGGA
AGCTCCCTCGTGCGCTCTCCTGTTCCGACCCTGCCGCTTACCGGATACCTGT
CCGCCTTTCTCCCTTCGGGAAGCGTGGCGCTTTCTCATAGCTCACGCTGTA
GGTATCTCAGTTCGGTGTAGGTCGTTCGCTCCAAGCTGGGCTGTGTGCACG
AACCCCCCGTTCAGCCCGACCGCTGCGCCTTATCCGGTAACTATCGTCTTGA
GTCCAACCCGGTAAGACACGACTTATCGCCACTGGCAGCAGCCACTGGTA
ACAGGATTAGCAGAGCGAGGTATGTAGGCGGTGCTACAGAGTTCTTGAAGT
GGTGGCCTAACTACGGCTACACTAGAAGAACAGTATTTGGTATCTGCGCTC
TGCTGAAGCCAGTTACCTTCGGAAAAAGAGTTGGTAGCTCTTGATCCGGCA
AACAAACCACCGCTGGTAGCGGTTTTTTTGTTTGCAAGCAGCAGATTACGC
GCAGAAAAAAAGGATCTCAAGAAGATCCTTTGATCTTTTCTACGGGGTCTG
ACGCTCAGTGGAACGAAAACTCACGTTAAGGGATTTTGGTCATGAGATTAT
CAAAAAGGATCTTCACCTAGATCCTTTTAAATTAAAAATGAAGTTTTAAATC

AATCTAAAGTATATATGAGTAAACTTGGTCTGACAGTTACCAATGCTTAATC
AGTGAGGCACCTATCTCAGCGATCTGTCTATTTCGTTCATCCATAGTTGCCT
GACTCCCCGTCGTGTAGATAACTACGATACGGGAGGGCTTACCATCTGGCC
CCAGTGCTGCAATGATACCGCGAGACCCACGCTCACCGGCTCCAGATTTAT
CAGCAATAAACCAGCCAGCCGGAAGGGCCGAGCGCAGAAGTGGTCCTGC
AACTTTATCCGCCTCCATCCAGTCTATTAATTGTTGCCGGGAAGCTAGAGTA
AGTAGTTCGCCAGTTAATAGTTTGCGCAACGTTGTTGCCATTGCTACAGGC
ATCGTGGTGTCACGCTCGTCGTTTGGTATGGCTTCATTCAGCTCCGGTTCCC
AACGATCAAGGCGAGTTACATGATCCCCCATGTTGTGCAAAAAAGCGGTTA
GCTCCTTCGGTCCTCCGATCGTTGTCAGAAGTAAGTTGGCCGCAGTGTTAT
CACTCATGGTTATGGCAGCACTGCATAATTCTCTTACTGTCATGCCATCCGTA
AGATGCTTTTCTGTGACTGGTGAGTACTCAACCAAGTCATTCTGAGAATAG
TGTATGCGGCGACCGAGTTGCTCTTGCCCGGCGTCAATACGGGATAATACC
GCGCCACATAGCAGAACTTTAAAAGTGCTCATCATTGGAAAACGTTCTTCG
GGGCGAAAACTCTCAAGGATCTTACCGCTGTTGAGATCCAGTTCGATGTAA
CCCACTCGTGCACCCAACTGATCTTCAGCATCTTTTACTTTCACCAGCGTTT
CTGGGTGAGCAAAAACAGGAAGGCAAAATGCCGCAAAAAAGGGAATAAG
GGCGACACGGAAATGTTGAATACTCATACTCTTCCTTTTTCAATATTATTGA
AGCATTTATCAGGGTTATTGTCTCATGAGCGGATACATATTTGAATGTATTTA
GAAAAATAAACAAATAGGGGTTCCGCGCACATTTCCCCGAAAAGTGCCAC
CTGACGTC

PcDNA3.1 Luc

PROMOTER

Luc

GACGGATCGGGAGATCTCCCGATCCCCTATGGTGCACTCTCAGTACAATCT
GCTCTGATGCCGCATAGTTAAGCCAGTATCTGCTCCCTGCTTGTGTGTTGGA
GGTCGCTGAGTAGTGCGCGAGCAAAATTTAAGCTACAACAAGGCAAGGCT
TGACCGACAATTGCATGAAGAATCTGCTTAGGGTTAGGCGTTTTGCGCTGC
TTCGCGATGTACGGGCCAGATATACGCGTTGACATTGATTATTGACTAGTTAT
TAATAGTAATCAATTACGGGGTCATTAGTTCATAGCCCATATATGGAGTTCCG
CGTTACATAACTTACGGTAAATGGCCCGCCTGGCTGACCGCCCAACGACCC
CCGCCCATTGACGTCAATAATGACGTATGTTCCCATAGTAACGCCAATAGGG
ACTTTCCATTGACGTCAATGGGTGGAGTATTTACGGTAAACTGCCCACTTGG
CAGTACATCAAGTGTATCATATGCCAAGTACGCCCCCTATTGACGTCAATGA
CGGTAAATGGCCCGCCTGGCATTATGCCCAGTACATGACCTTATGGGACTTT
CCTACTTGGCAGTACATCTACGTATTAGTCATCGCTATTACCATGGTGATGCG
GTTTTGGCAGTACATCAATGGGCGTGGATAGCGGTTTGACTCACGGGGATT
TCCAAGTCTCCACCCCATTGACGTCAATGGGAGTTTGTTTTGGCACCAAAA
TCAACGGGACTTTCCAAAATGTCGTAACAACTCCGCCCCATTGACGCAAAT
GGGCGGTAGGCGTGTACGGTGGGAGGTCTATATAAGCAGAGCTCTCTGGCT
AACTAGAGAACCCACTGCTTACTGGCTTATCGAAATTAATACGACTCACTAT
AGGGAGACCCAAGCTGGCTAGCATGGAAGATGCCAAAAACATTAAGAAGG
GCCCAGCGCCATTCTACCCACTCGAAGACGGGACCGCCGGCGAGCAGCTG
CACAAAGCCATGAAGCGCTACGCCCTGGTGCCCGGCACCATCGCCTTTACC
GACGCACATATCGAGGTGGACATTACCTACGCCGAGTACTTCGAGATGAGC
GTTCGGCTGGCAGAAGCTATGAAGCGCTATGGGCTGAATACAAACCATCGG
ATCGTGGTGTGCAGCGAGAATAGCTTGCAGTTCTTCATGCCCGTGTTGGGT
GCCCTGTTCATCGGTGTGGCTGTGGCCCCAGCTAACGACATCTACAACGAG
CGCGAGCTGCTGAACAGCATGGGCATCAGCCAGCCCACCGTCGTATTCGTG
AGCAAGAAAGGGCTGCAAAAGATCCTCAACGTGCAAAAGAAGCTACCGAT
CATACAAAAGATCATCATCATGGATAGCAAGACCGACTACCAGGGCTTCCA
AAGCATGTACACCTTCGTGACTTCCCATTTGCCACCCGGCTTCAACGAGTA
CGACTTCGTGCCCGAGAGCTTCGACCGGGACAAAACCATCGCCCTGATCAT
GAACAGTAGTGGCAGTACCGGATTGCCCAAGGGCGTAGCCCTACCGCACC
GCACCGCTTGTGTCCGATTCAGTCATGCCCGCGACCCCATCTTCGGCAACC
AGATCATCCCCGACACCGCTATCCTCAGCGTGGTGCCATTTCACCACGGCTT
CGGCATGTTCACCACGCTGGGCTACTTGATCTGCGGCTTTCGGGTCGTGCT
CATGTACCGCTTCGAGGAGGAGCTATTCTTGCGCAGCTTGCAAGACTATAA
GATTCAATCTGCCCTGCTGGTGCCCACACTATTTAGCTTCTTCGCTAAGAGC

ACTCTCATCGACAAGTACGACCTAAGCAACTTGCACGAGATCGCCAGCGG
CGGGGCGCCGCTCAGCAAGGAGGTAGGTGAGGCCGTGGCCAAACGCTTCC
ACCTACCAGGCATCCGCCAGGGCTACGGCCTGACAGAAACAACCAGCGCC
ATTCTGATCACCCCCGAAGGGGACGACAAGCCTGGCGCAGTAGGCAAGGT
GGTGCCCTTCTTCGAGGCTAAGGTGGTGGACTTGGACACCGGTAAGACAC
TGGGTGTGAACCAGCGCGGCGAGCTGTGCGTCCGTGGCCCCATGATCATG
AGCGGCTACGTTAACAACCCCGAGGCTACAAACGCTCTCATCGACAAGGA
CGGCTGGCTGCACAGCGGCGACATCGCCTACTGGGACGAGGACGAGCACT
TCTTCATCGTGGACCGGCTGAAGAGCCTGATCAAATACAAGGGCTACCAGG
TAGCCCCAGCCGAACTGGAGAGCATCCTGCTGCAACACCCCAACATCTTCG
ACGCCGGGGTCGCCGGCCTGCCCGACGACGATGCCGGCGAGCTGCCCGCC
GCAGTCGTCGTGCTGGAACACGGTAAAACCATGACCGAGAAGGAGATCGT
GGACTATGTGGCCAGCCAGGTTACAACCGCCAAGAAGCTGCGCGGTGGTG
TTGTGTTCGTGGACGAGGTGCCTAAAGGACTGACCGGCAAGTTGGACGCC
CGCAAGATCCGCGAGATTCTCATTAAGGCCAAGAAGGGCGGCAAGATCGC
CGTGGCAAGCTTGGTACCGAGCTCGGATCCACTAGTCCAGTGTGGTGGAAT
TCTGCAGATATCCAGCACAGTGGCGGCCGCTCGAGTCTAGAGGGCCCGTTT
AAACCCGCTGATCAGCCTCGACTGTGCCTTCTAGTTGCCAGCCATCTGTTG
TTTGCCCCTCCCCCGTGCCTTCCTTGACCCTGGAAGGTGCCACTCCCACTG
TCCTTTCCTAATAAAATGAGGAAATTGCATCGCATTGTCTGAGTAGGTGTCA
TTCTATTCTGGGGGGTGGGGTGGGGCAGGACAGCAAGGGGGAGGATTGGG
AAGACAATAGCAGGCATGCTGGGGATGCGGTGGGCTCTATGGCTTCTGAGG
CGGAAAGAACCAGCTGGGGCTCTAGGGGGTATCCCCACGCGCCCTGTAGC
GGCGCATTAAGCGCGGCGGGTGTGGTGGTTACGCGCAGCGTGACCGCTAC
ACTTGCCAGCGCCCTAGCGCCCGCTCCTTTCGCTTTCTTCCCTTCCTTTCTC
GCCACGTTCGCCGGCTTTCCCCGTCAAGCTCTAAATCGGGGGCTCCCTTTA
GGGTTCCGATTTAGTGCTTTACGGCACCTCGACCCCAAAAAACTTGATTAG
GGTGATGGTTCACGTAGTGGGCCATCGCCCTGATAGACGGTTTTTCGCCCTT
TGACGTTGGAGTCCACGTTCTTTAATAGTGGACTCTTGTTCCAAACTGGAA
CAACACTCAACCCTATCTCGGTCTATTCTTTTGATTTATAAGGGATTTTGCCG
ATTTCGGCCTATTGGTTAAAAAATGAGCTGATTTAACAAAAATTTAACGCGA
ATTAATTCTGTGGAATGTGTGTCAGTTAGGGTGTGGAAAGTCCCCAGGCTC
CCCAGCAGGCAGAAGTATGCAAAGCATGCATCTCAATTAGTCAGCAACCAG
GTGTGGAAAGTCCCCAGGCTCCCCAGCAGGCAGAAGTATGCAAAGCATGC
ATCTCAATTAGTCAGCAACCATAGTCCCGCCCCTAACTCCGCCCATCCCGCC
CCTAACTCCGCCCAGTTCCGCCCATTCTCCGCCCCATGGCTGACTAATTTTT
TTTATTTATGCAGAGGCCGAGGCCGCCTCTGCCTCTGAGCTATTCCAGAAGT
AGTGAGGAGGCTTTTTTGGAGGCCTAGGCTTTTGCAAAAAGCTCCCGGGA

GCTTGTATATCCATTTTCGGATCTGATCAAGAGACAGGATGAGGATCGTTTC
GCATGATTGAACAAGATGGATTGCACGCAGGTTCTCCGGCCGCTTGGGTGG
AGAGGCTATTCGGCTATGACTGGGCACAACAGACAATCGGCTGCTCTGATG
CCGCCGTGTTCCGGCTGTCAGCGCAGGGGCGCCCGGTTCTTTTTGTCAAGA
CCGACCTGTCCGGTGCCCTGAATGAACTGCAGGACGAGGCAGCGCGGCTA
TCGTGGCTGGCCACGACGGGCGTTCCTTGCGCAGCTGTGCTCGACGTTGTC
ACTGAAGCGGGAAGGGACTGGCTGCTATTGGGCGAAGTGCCGGGGCAGG
ATCTCCTGTCATCTCACCTTGCTCCTGCCGAGAAAGTATCCATCATGGCTGA
TGCAATGCGGCGGCTGCATACGCTTGATCCGGCTACCTGCCCATTCGACCA
CCAAGCGAAACATCGCATCGAGCGAGCACGTACTCGGATGGAAGCCGGTC
TTGTCGATCAGGATGATCTGGACGAAGAGCATCAGGGGCTCGCGCCAGCC
GAACTGTTCGCCAGGCTCAAGGCGCGCATGCCCGACGGCGAGGATCTCGT
CGTGACCCATGGCGATGCCTGCTTGCCGAATATCATGGTGGAAAATGGCCG
CTTTTCTGGATTCATCGACTGTGGCCGGCTGGGTGTGGCGGACCGCTATCA
GGACATAGCGTTGGCTACCCGTGATATTGCTGAAGAGCTTGGCGGCGAATG
GGCTGACCGCTTCCTCGTGCTTTACGGTATCGCCGCTCCCGATTCGCAGCG
CATCGCCTTCTATCGCCTTCTTGACGAGTTCTTCTGAGCGGGACTCTGGGGT
TCGAAATGACCGACCAAGCGACGCCCAACCTGCCATCACGAGATTTCGATT
CCACCGCCGCCTTCTATGAAAGGTTGGGCTTCGGAATCGTTTTCCGGGACG
CCGGCTGGATGATCCTCCAGCGCGGGGATCTCATGCTGGAGTTCTTCGCCC
ACCCCAACTTGTTTATTGCAGCTTATAATGGTTACAAATAAAGCAATAGCAT
CACAAATTTCACAAATAAAGCATTTTTTTCACTGCATTCTAGTTGTGGTTTG
TCCAAACTCATCAATGTATCTTATCATGTCTGTATACCGTCGACCTCTAGCTA
GAGCTTGGCGTAATCATGGTCATAGCTGTTTCCTGTGTGAAATTGTTATCCG
CTCACAATTCCACACAACATACGAGCCGGAAGCATAAAGTGTAAAGCCTGG
GGTGCCTAATGAGTGAGCTAACTCACATTAATTGCGTTGCGCTCACTGCCC
GCTTTCCAGTCGGGAAACCTGTCGTGCCAGCTGCATTAATGAATCGGCCAA
CGCGCGGGGAGAGGCGGTTTGCGTATTGGGCGCTCTTCCGCTTCCTCGCTC
ACTGACTCGCTGCGCTCGGTCGTTCGGCTGCGGCGAGCGGTATCAGCTCAC
TCAAAGGCGGTAATACGGTTATCCACAGAATCAGGGGATAACGCAGGAAA
GAACATGTGAGCAAAAGGCCAGCAAAAGGCCAGGAACCGTAAAAAGGCC
GCGTTGCTGGCGTTTTTCCATAGGCTCCGCCCCCCTGACGAGCATCACAAA
AATCGACGCTCAAGTCAGAGGTGGCGAAACCCGACAGGACTATAAAGATA
CCAGGCGTTTCCCCCTGGAAGCTCCCTCGTGCGCTCTCCTGTTCCGACCCT
GCCGCTTACCGGATACCTGTCCGCCTTTCTCCCTTCGGGAAGCGTGGCGCT
TTCTCATAGCTCACGCTGTAGGTATCTCAGTTCGGTGTAGGTCGTTCGCTCC
AAGCTGGGCTGTGTGCACGAACCCCCCGTTCAGCCCGACCGCTGCGCCTT
ATCCGGTAACTATCGTCTTGAGTCCAACCCGGTAAGACACGACTTATCGCC

ACTGGCAGCAGCCACTGGTAACAGGATTAGCAGAGCGAGGTATGTAGGCG
GTGCTACAGAGTTCTTGAAGTGGTGGCCTAACTACGGCTACACTAGAAGAA
CAGTATTTGGTATCTGCGCTCTGCTGAAGCCAGTTACCTTCGGAAAAAGAG
TTGGTAGCTCTTGATCCGGCAAACAAACCACCGCTGGTAGCGGTTTTTTTG
TTTGCAAGCAGCAGATTACGCGCAGAAAAAAAGGATCTCAAGAAGATCCT
TTGATCTTTTCTACGGGGTCTGACGCTCAGTGGAACGAAAACTCACGTTAA
GGGATTTTGGTCATGAGATTATCAAAAAGGATCTTCACCTAGATCCTTTTAA
ATTAAAAATGAAGTTTTAAATCAATCTAAAGTATATATGAGTAAACTTGGTCT
GACAGTTACCAATGCTTAATCAGTGAGGCACCTATCTCAGCGATCTGTCTAT
TTCGTTCATCCATAGTTGCCTGACTCCCCGTCGTGTAGATAACTACGATACG
GGAGGGCTTACCATCTGGCCCCAGTGCTGCAATGATACCGCGAGACCCACG
CTCACCGGCTCCAGATTTATCAGCAATAAACCAGCCAGCCGGAAGGGCCG
AGCGCAGAAGTGGTCCTGCAACTTTATCCGCCTCCATCCAGTCTATTAATTG
TTGCCGGGAAGCTAGAGTAAGTAGTTCGCCAGTTAATAGTTTGCGCAACGT
TGTTGCCATTGCTACAGGCATCGTGGTGTCACGCTCGTCGTTTGGTATGGCT
TCATTCAGCTCCGGTTCCCAACGATCAAGGCGAGTTACATGATCCCCCATGT
TGTGCAAAAAAGCGGTTAGCTCCTTCGGTCCTCCGATCGTTGTCAGAAGTA
AGTTGGCCGCAGTGTTATCACTCATGGTTATGGCAGCACTGCATAATTCTCT
TACTGTCATGCCATCCGTAAGATGCTTTTCTGTGACTGGTGAGTACTCAACC
AAGTCATTCTGAGAATAGTGTATGCGGCGACCGAGTTGCTCTTGCCCGGCG
TCAATACGGGATAATACCGCGCCACATAGCAGAACTTTAAAAGTGCTCATC
ATTGGAAAACGTTCTTCGGGGCGAAAACTCTCAAGGATCTTACCGCTGTTG
AGATCCAGTTCGATGTAACCCACTCGTGCACCCAACTGATCTTCAGCATCTT
TTACTTTCACCAGCGTTTCTGGGTGAGCAAAAACAGGAAGGCAAAATGCC
GCAAAAAAGGGAATAAGGGCGACACGGAAATGTTGAATACTCATACTCTTC
CTTTTTCAATATTATTGAAGCATTTATCAGGGTTATTGTCTCATGAGCGGATA
CATATTTGAATGTATTTAGAAAAATAAACAAATAGGGGTTCCGCGCACATTT
CCCCGAAAAGTGCCACCTGACGTC
