# Supplementary material for: Membrane protein-regulated networks across human cancers
Source: Nat Commun. 2019 Jul 16;10:3131. doi: 10.1038/s41467-019-10920-8 (PMC6635409; doi:10.1038/s41467-019-10920-8)
Supplement: Supplementary file 12 — Reporting Summary [file 41467_2019_10920_MOESM12_ESM.pdf]

nature research

Corresponding author(s): Yuan-Soon Ho and Jinn-Moon Yang
Last updated by author(s): Jun 4, 2019

Reporting Summary

Nature Research wishes to improve the reproducibility of the work that we publish. This form provides structure for consistency and transparency in reporting. For further information on Nature Research policies, see Authors & Referees and the Editorial Policy Checklist.

Statistics

For all statistical analyses, confirm that the following items are present in the figure legend, table legend, main text, or Methods section.

n/a	Confirmed	
☐	☒	The exact sample size (*n*) for each experimental group/condition, given as a discrete number and unit of measurement
☐	☒	A statement on whether measurements were taken from distinct samples or whether the same sample was measured repeatedly
☐	☒	The statistical test(s) used AND whether they are one- or two-sided<br>*Only common tests should be described solely by name; describe more complex techniques in the Methods section.*
☐	☒	A description of all covariates tested
☐	☒	A description of any assumptions or corrections, such as tests of normality and adjustment for multiple comparisons
☐	☒	A full description of the statistical parameters including central tendency (e.g. means) or other basic estimates (e.g. regression coefficient) AND variation (e.g. standard deviation) or associated estimates of uncertainty (e.g. confidence intervals)
☐	☒	For null hypothesis testing, the test statistic (e.g. *F*, *t*, *r*) with confidence intervals, effect sizes, degrees of freedom and *P* value noted<br>*Give P values as exact values whenever suitable.*
☒	☐	For Bayesian analysis, information on the choice of priors and Markov chain Monte Carlo settings
☐	☒	For hierarchical and complex designs, identification of the appropriate level for tests and full reporting of outcomes
☐	☒	Estimates of effect sizes (e.g. Cohen's *d*, Pearson's *r*), indicating how they were calculated

Our web collection on statistics for biologists contains articles on many of the points above.

Software and code

Policy information about availability of computer code

Data collection	TCGA-assembler (Version 2.0.0)<br>RStudio (R Version 3.2.1)<br>The custom codes of SIM and CaMPNets are provided in http://campnets.life.nctu.edu.tw/download.php.
Data analysis	Microsoft office 2016<br>RStudio (R Version 3.2.1)<br>R 'limma' package (v. 3.24.15)<br>R 'affy' package (v. 1.46.1)<br>R 'survival' package (v. 2.37.2)<br>Bioconductor (v. 3.1)<br>IBM SPSS Statistics (Version 22)<br>SigmaPlot (Version 10)<br>iGEMDOCK<br>3D-BLAST<br>DALI<br>ImageJ<br>Roche LightCycler (Version 4)<br>Living Image software (Version 4.0)<br>Leica TCS SP5 Confocal Spectral Microscope Imaging System

For manuscripts utilizing custom algorithms or software that are central to the research but not yet described in published literature, software must be made available to editors/reviewers. We strongly encourage code deposition in a community repository (e.g. GitHub). See the Nature Research guidelines for submitting code & software for further information.

Data

Policy information about availability of data

All manuscripts must include a data availability statement. This statement should provide the following information, where applicable:

- Accession codes, unique identifiers, or web links for publicly available datasets
- A list of figures that have associated raw data
- A description of any restrictions on data availability

Please see 'Data availability' statement.
The data generated in this study are available on the website of CaMPNets (http://campnets.life.nctu.edu.tw).
The accession number for microarray data reported in this paper is GEO: GSE105445.
The data that support the findings of this study are included in this published article and its Supplementary Information files. All other relevant data are available from the corresponding author upon reasonable request.

Field-specific reporting

Please select the one below that is the best fit for your research. If you are not sure, read the appropriate sections before making your selection.

☒ Life sciences ☐ Behavioural & social sciences ☐ Ecological, evolutionary & environmental sciences

For a reference copy of the document with all sections, see nature.com/documents/nr-reporting-summary-flat.pdf

Life sciences study design

All studies must disclose on these points even when the disclosure is negative.

Sample size	The experiments were repeated at least three times. The description can be found in Figure 7c-g, Supplementary Figure 28d-f, Supplementary Figure 36b-e, Supplementary Figure 38d, e, and Supplementary Figure 39c, d.<br>For animal studies, total thirty mice in the orthotropic xenograft model were randomized into six groups (n = 5 per group).
Data exclusions	No bioluminescent imaging data (animal) was excluded from the analysis. Please see 'Bioluminescent imaging' subsection.
Replication	Experiments were carried out in biological and/or technical replicates as indicated in the results part (text and figure legends). The reproducibility of the experimental findings was verified by performing additional independent experiments (at least two) or by having several technical replicates (as described in the figure legends). All attempts at replication were confirmed to be successful.
Randomization	The xenograft mice were randomized into six groups (n = 5 per group). Please see 'Orthotropic xenograft model' subsection. For simulation experiments, we randomly generated 1,000 trials for each test. Please see 'Cancer membrane protein-regulated networks (CaMPNets)' subsection, 'Quantification of tumor homogeneity in CaMPNets across human cancers' subsection, and Supplementary Note 4.<br>For animal studies, all mice were randomized into six groups (n = 5 per group) and i.p. injected with PBS, 100 or 200 μg/kg bupropion three times per week, and with or without nicotine treatment (10 μg/ml) via their drinking water.
Blinding	N/A.

Reporting for specific materials, systems and methods

We require information from authors about some types of materials, experimental systems and methods used in many studies. Here, indicate whether each material, system or method listed is relevant to your study. If you are not sure if a list item applies to your research, read the appropriate section before selecting a response.

Materials & experimental systems

n/a	Involved in the study	
☐	☒	Antibodies
☐	☒	Eukaryotic cell lines
☒	☐	Palaeontology
☐	☒	Animals and other organisms
☐	☒	Human research participants
☒	☐	Clinical data

Methods

n/a	Involved in the study	
☒	☐	ChIP-seq
☒	☐	Flow cytometry
☒	☐	MRI-based neuroimaging

Antibodies

Antibodies used	The detailed information of all antibodies used in our present study has been provided in the "Supplementary Table 7".
Validation	All antibodies used were of commercial origin and have been validated by the respective manufacturers.

Eukaryotic cell lines

Policy information about cell lines

Cell line source(s)	All the cell lines were purchased from American Type Culture Collection (ATCC, Manassas, VA, USA), except that RT4 and MIA PaCa-2 cell lines were purchased from The Bioresource Collection and Research Center (BCRC, Hsinchu, Taiwan).
Authentication	Yes, all the cell lines were purchased from American Type Culture Collection (ATCC, Manassas, VA, USA), except that RT4 and MIA PaCa-2 cell lines were purchased from The Bioresource Collection and Research Center (BCRC, Hsinchu, Taiwan). Additionally, the STR genotyping has been performed on MDA-MB-231 cell line by The Food Industry Research and Development Institute (Hsinchu, Taiwan). The STR genotyping has also been performed on BT474, SKBR3, AU565, Hs578T, and MCF-10A cell lines by the Mission Biotech (Taipei, Taiwan). The A549 and Hep3B cell lines are under STR genotyping examination.
Mycoplasma contamination	Please see 'Cell culture and patient samples' subsection.
Commonly misidentified lines (See ICLAC register)	N/A.

Animals and other organisms

Policy information about studies involving animals; ARRIVE guidelines recommended for reporting animal research

Laboratory animals	For split luciferase complementation assay, six-week-old female BALB/c nude (CAnN.Cg-Foxn1nu/Crl) mice were purchased from the National Science Council Animal Center of Taipei and housed in micro-isolator cages at the Laboratory Animal Center of Taipei Medical University (Taipei, Taiwan). For metastasis observation, SCID mice (NOD.CB17/Icr-Prkdcscid/NcrCrl, female, 4 weeks old) purchased from the National Science Council Animal Center of Taipei and housed at the Laboratory Animal Center (LAC) in the National Defense Medical Center (NDMC, Taipei, Taiwan).
Wild animals	N/A.
Field-collected samples	N/A.
Ethics oversight	The animal experiments were approved with number LAC-201-0177 and IACUC-15-327.

Note that full information on the approval of the study protocol must also be provided in the manuscript.

Human research participants

Policy information about studies involving human research participants

Population characteristics	All human breast cancer samples were obtained from anonymous donors at Taipei Medical University Hospital, Taipei, according to a protocol approved by the Institutional Review Board (N201612082).
Recruitment	All types of breast cancer patient were recruited in the human clinical trial, whereas TNBC and HER2 breast cancer patients were selected for CHRNA9/ERBB2 FRET efficiency measurement.
Ethics oversight	All human breast cancer samples were obtained from anonymous donors at Taipei Medical University Hospital, Taipei, according to a protocol approved by the Institutional Review Board (N201612082).

Note that full information on the approval of the study protocol must also be provided in the manuscript.
